# Affinity and Selectivity of Protein–Ligand Recognition: A Minor Chemical Modification Changes Carbonic Anhydrase Binding Profile

**DOI:** 10.1021/acs.jmedchem.5c01421

**Published:** 2025-08-13

**Authors:** Audrius Zakšauskas, Vaida Paketurytė-Latvė, Alberta Janku̅naitė, Edita Čapkauskaitė, Yann Becart, Alexey Smirnov, Klára Pospíšilová, Janis Leitans, Jiří Brynda, Andris Kazaks, Lina Baranauskienė, Elena Manakova, Saulius Gražulis, Visvaldas Kairys, Kaspars Tars, Pavlína Řezáčová, Daumantas Matulis

**Affiliations:** † Department of Biothermodynamics and Drug Design, Institute of Biotechnology, Life Sciences Center, 54694 Vilnius University, Saulėtekio al. 7, Vilnius LT-10257, Lithuania; ‡ 89220 Institute of Organic Chemistry and Biochemistry of the Czech Academy of Sciences, Flemingovo n. 2, Prague 6 16610, Czech Republic; § 382968 Latvian Biomedical Research and Study Centre, Ratsupites 1 k-1, Riga LV-1067, Latvia; ∥ Department of Protein−DNA Interactions, Institute of Biotechnology, Life Sciences Center, 54694 Vilnius University, Saulėtekio al. 7, Vilnius LT-10257, Lithuania; ⊥ Department of Bioinformatics, Institute of Biotechnology, Life Sciences Center, 54694 Vilnius University, Saulėtekio 7, Vilnius LT-10257, Lithuania; 6 Sector of Crystallography and Cheminformatics, Institute of Biotechnology, Life Sciences Center, 54694 Vilnius University, Saulėtekio al. 7, Vilnius LT-10257, Lithuania

## Abstract

Discovery of small-molecule drugs relies on their strong binding affinity compared to nontarget proteins, thus possessing selectivity. Minor chemical structure changes usually exhibit little change in the compound efficacy, with rare exceptions. We developed a series of nearly 50 *ortho*-substituted benzenesulfonamides and experimentally measured their interactions with the 12 catalytically active human carbonic anhydrase (CA) isozymes. Inhibitors were designed using seven different substituent groups, including 4-*sulfanyl*-substituted 3-sulfamoyl benzoates and benzamides, 4-*sulfinyl*-substituted 3-sulfamoyl benzoates and benzamides, 4-*sulfonyl*-substituted 3-sulfamoyl benzoates and benzamides, and 4-amino-substituted benzamides. The oxidation state of sulfur at the *ortho* position significantly influenced the compound’s affinity for CAIX, a target for anticancer drugs, demonstrating affinities hundreds of thousands of times stronger than related compounds. Coupled with X-ray crystal structures and molecular docking, the relationship between structure and thermodynamics offers insights into how small changes in the structure lead to significant changes in affinity for drug design.

## Graphical abstract

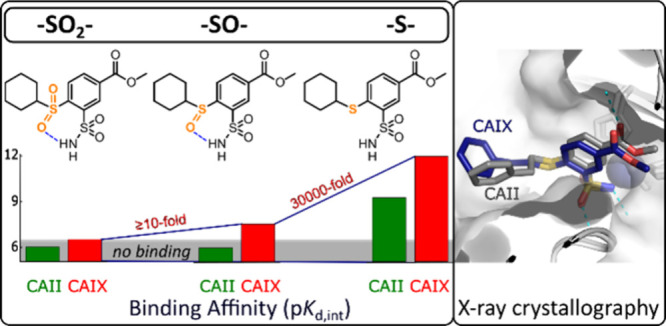

## Introduction

A detailed understanding of protein–ligand recognition is an essential goal in small-molecule drug discovery.^1^ Any drug-like chemical compound should bind the disease-related target protein with sufficiently high affinity. Furthermore, the compound must bind with high selectivity and thus not interact strongly with other nontarget proteins whose inhibition or binding could cause undesired side effects.^2^ Rational design of such compounds is complex because of the limited understanding of the underlying energies of binding and how a compound recognizes and binds to the target protein.

As a model system, we study sulfonamide compound binding to human carbonic anhydrases (CA), zinc-containing enzymes.^3^ Humans have 12 catalytically active CA isozymes (EC 4.2.1.1).^4–6^ The enzyme catalyzes the reversible hydration of carbon dioxide and has many essential physiological functions. Since these enzymes function in pH and electrolyte homeostasis and regulation, many drugs target CA isozymes to treat diseases like glaucoma, edema, obesity, epilepsy, infertility, and cancer.^7–9^ Primary sulfonamides are the most investigated CA inhibitors.^10–12^ Their amino group binds directly to the catalytic zinc in the active site by forming a coordination bond and inhibits the activity of all CA isozymes. However, the binding affinity may be low or high and depends on small details of each compound arrangement on the protein surface, possible steric hindrances, or attraction due to hydrogen bonds or hydrophobic interactions.^13^

The 12 catalytically active human CA isozymes have nearly identical beta-sheet folds. Their active sites are highly similar in shape, but several amino acids in the active site vary among the isozymes.^14–16^ Because the differences in the active site amino acid composition are small, it is difficult to design compounds that would bind one isozyme with high affinity while all other isozymes with low affinity, thus leading toward high selectivity for only one isozyme. The active site of CAs is funnel-shaped and has hydrophobic and hydrophilic sides for some isozymes. Differences in a few amino acids determine the selectivity of inhibitors for particular isozymes. Introducing various scaffolds on the aromatic sulfonamide ring targets unique residues in the active site.^17^ In most studies, the tails are relatively distant from the sulfonamide group, resulting in weak interactions with peripheral amino acids. The affinity profile depended mostly on the substituents located close to the sulfonamide headgroup.^18–20^ This is also dependent on the flexibility of the substituents, which help adjust to the protein shape.

Depending on the variation of the substituents while investigating doubly substituted compounds,^20–22^ several high-affinity compounds exhibiting picomolar *K*_d_ were obtained for CAVII, CAIX, CAXII, and CAXIV. A significant achievement was a *LJ15-12* compound that exhibited 0.08 pM intrinsic *K*_d_ for CAIX.^20^ In this study, we investigate the functional groups in the *ortho* position by varying 13 substituents from small methyl to bulky adamantyl. Sulfinyl and amino substituents were also synthesized to examine the influence of the linking atoms. Interestingly, the substitution of only one atom, an addition of an oxygen atom in the *ortho* position, decreased compound affinity for nontarget isozymes by a million-fold, significantly improving the selectivity, which is one of the main goals in small-molecule drug discovery.

## Results

### Organic Synthesis of Designed Compounds

The synthesis of 2,5-disubstituted benzenesulfonamides was performed starting from 4-chloro-3-sulfamoylbenzoic acid **1** (Scheme 1). Methyl ester **2** was obtained from 4-chloro-3-sulfamoylbenzoic acid **1** by reflux in methanol in the presence of a catalytic amount of sulfuric acid. Amide **3** was synthesized by refluxing acid **1** with thionyl chloride in toluene and subsequent treatment, the resulting anhydride with an appropriate amine, using as the base excess of amine according to the procedure reported in ref 21.

**Scheme 1:**
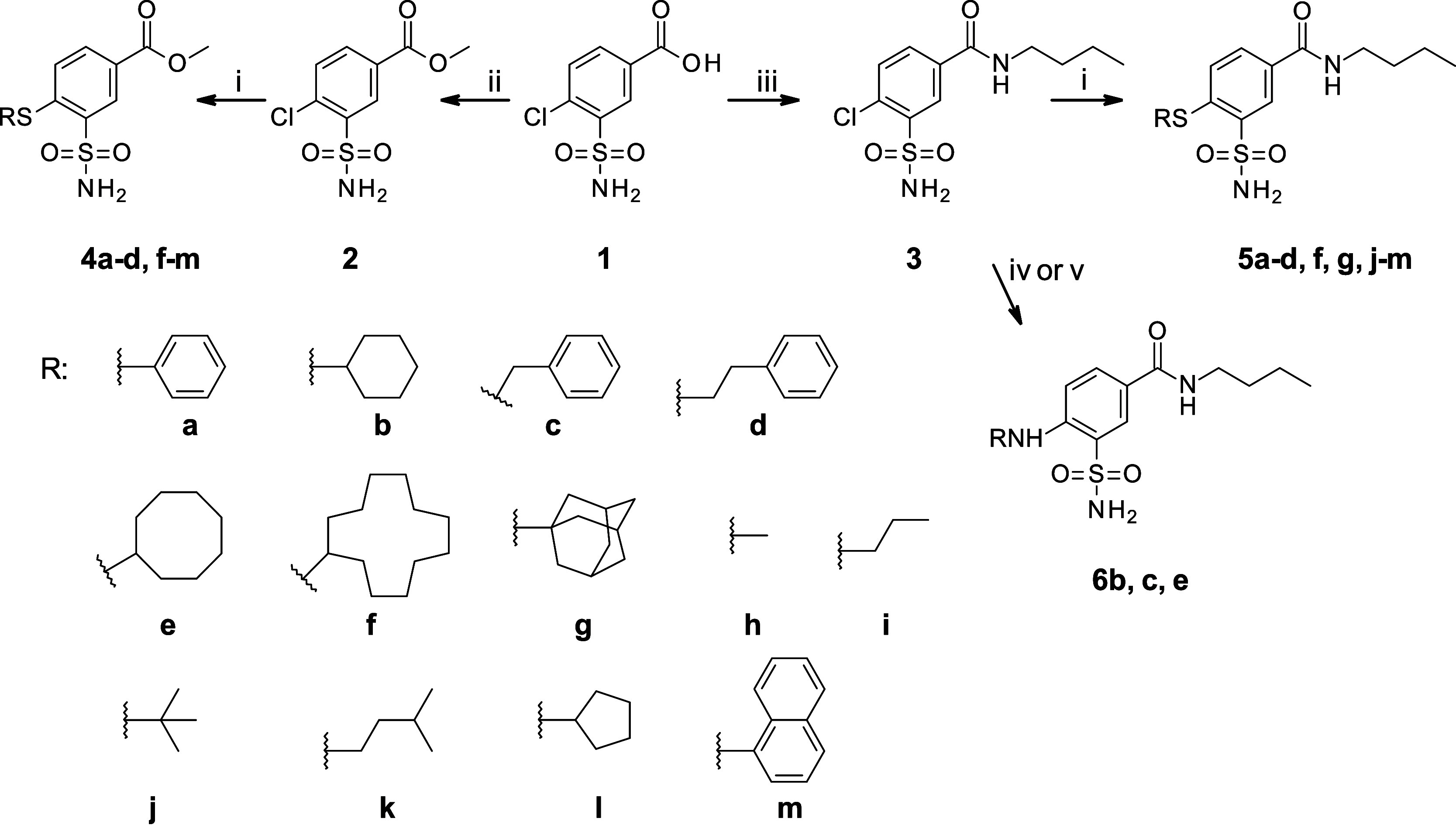
Synthesis of Methyl 4-Substituted-3-Sulfamoylbenzoates **4a–d, f–m**, 4-Substituted 3-Sulfamoylbenzamides **5a–d, f, g, j–m**, and 4-Amino-Substituted Benzamides **6b, c, e** ^a^

a Reagents and conditions: (i) RSH, K_2_CO_3_, DMF, 80 °C; (ii) H_2_SO_4_, MeOH, Δ; (iii) (a) SOCl_2_, toluene, Δ, (b) RNH_2_, THF, 0 °C; (iv) RNH_2_, 130 °C; (v) RNH_2_, TEA, toluene, Δ.

The synthesis of 4-substituted sulfamoylbenzoic acid derivatives **4a–d, f–m** was achieved by aromatic nucleophilic substitution of the chlorine substituent with various thiols under an inert atmosphere in dimethylformamide using potassium carbonate as the base (Scheme 1). All thiols were commercially available except cyclododecylthiol, which was synthesized by subjecting cyclododecanone to reaction with 1,2-ethanedithiol and subsequent reduction of intermediate dithiolane with *n*-butyllithium.^23^

The 4-substituted 3-sulfamoylbenzamides **5a**–**d**, **f**, **g**, **j–m** were synthesized using the same reaction conditions as 4-sulfanyl-substituted esters **4a–d, f–m**. Substitution of the chlorine group with amines required harsher reaction conditions than thiols. Therefore, 4-amino-substituted benzamides **6b** and **c** were synthesized by heating amide **3** over the appropriate amine at 130 °C. Compound **6e** was synthesized by boiling amide **3** in toluene with 2 equiv of cyclooctylamine and 2 equiv of triethylamine.

The oxidation of esters **4a–d, h, i, k–m** and benzamides **5a**, **b**, **k–m** to the sulfinyl and sulfonyl compounds was performed using in situ generated peracetic acid (Scheme 2). The reaction was carried out at room temperature and produced 4-sulfinyl compounds **7a, b, h, i, k–m**, **8a, b, k–m**, and heating the reaction mixture at 70 °C yielded the corresponding 4-sulfonyl compounds **9a–d, h, i, l, m, 10b,** and **k–m**.

**Scheme 2:**
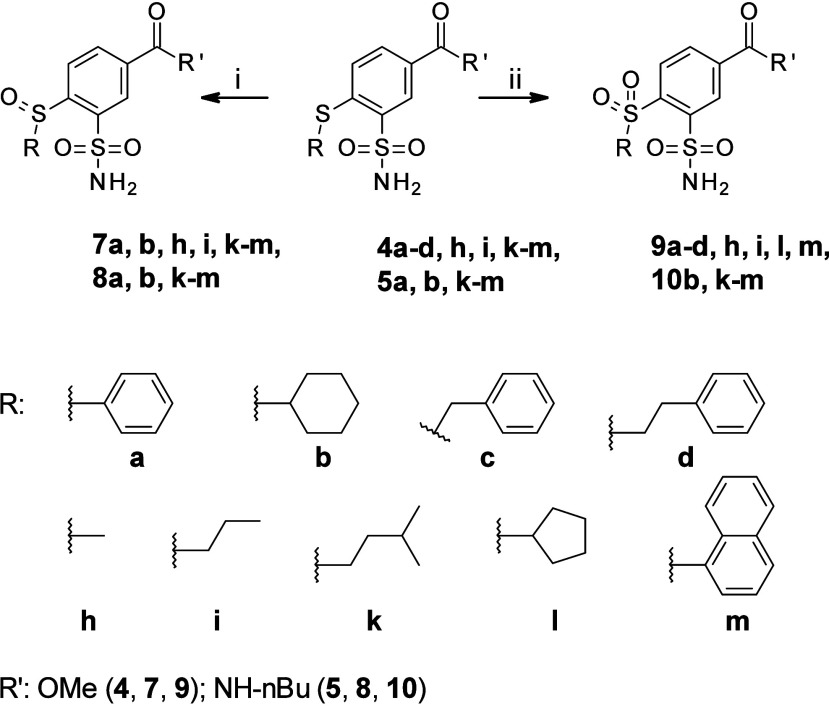
Synthesis of Methyl 4-Sulfinyl-Substituted-3-Sulfamoyl Benzoates **7a, b, h, i, k–m**, *N*-Butyl-4-sulfinyl-Substituted-3-Sulfamoylbenzamides **8a, b, k–m**, Methyl 4-Sulfonyl-Substituted-3-Sulfamoylbenzoates **9a-d, h, i, l, m,** and *N*-Butyl-4-sulfonyl-Substituted-3-Sulfamoylbenzamides **10b, k–m** ^a^

a Reagents and conditions: (i) 30% H_2_O_2_, AcOH, r. t.; (ii) 30% H_2_O_2_, AcOH, 70 °C.

### Compound Binding to CA Isozymes

All synthesized compounds were divided into 7 groups: **4­(a–d, f–m)**, **5­(a–d, f, g, j–m)**, **6­(b, c, e)**, **7­(a, b, h, i, k–m)**, **8­(a, k–m)**, **9­(a–d, h, i, l, m)**, and **10­(b, k–m)** (Figure 1). Compounds that started with numbers **4**, **7**, and **9** were 4-sulfanyl-, 4-sulfinyl, and 4-sulfonyl- substituted esters, respectively. Compounds starting with the numbers **5**, **8**, and **10** were 4-sulfanyl-, 4-sulfinyl, and 4-sulfonyl- substituted benzamides analogous to the previous series. Compounds starting with the number **6** were 4-amino-substituted benzamides. Various linear, branched, and cyclic aliphatic and aromatic substituents at the *ortho* position were tested to assess whether size, flexibility, and hydrophobicity affect affinity. Compound affinities for the 12 catalytically active human CA isozymes are listed in Table 1.

**Figure 1:**
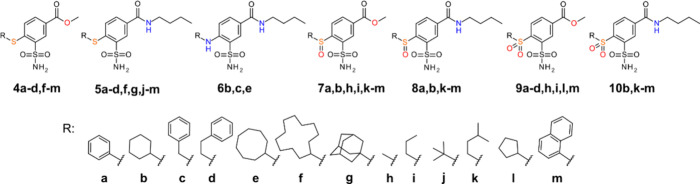
Chemical structures of the compounds synthesized and investigated in this study. Compounds **1**, **2**, and **3** are the starting compounds of the synthesis shown in Scheme 1.

**Table 1:**
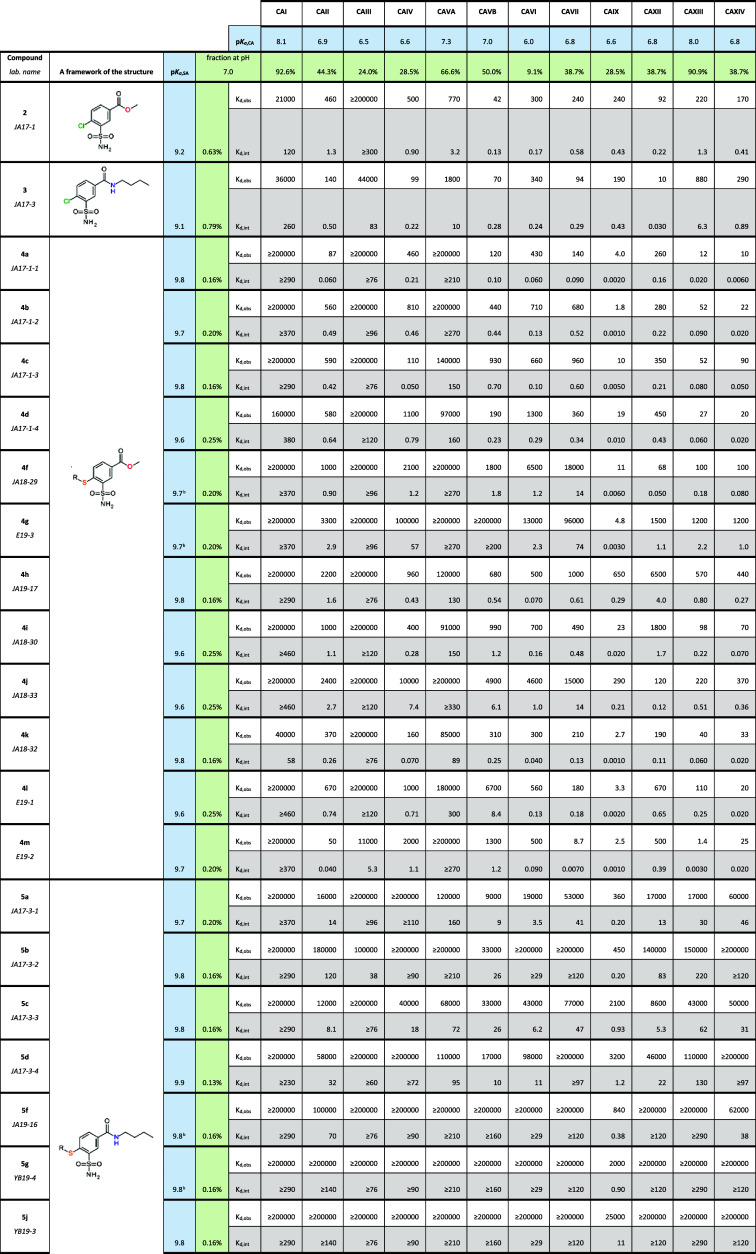

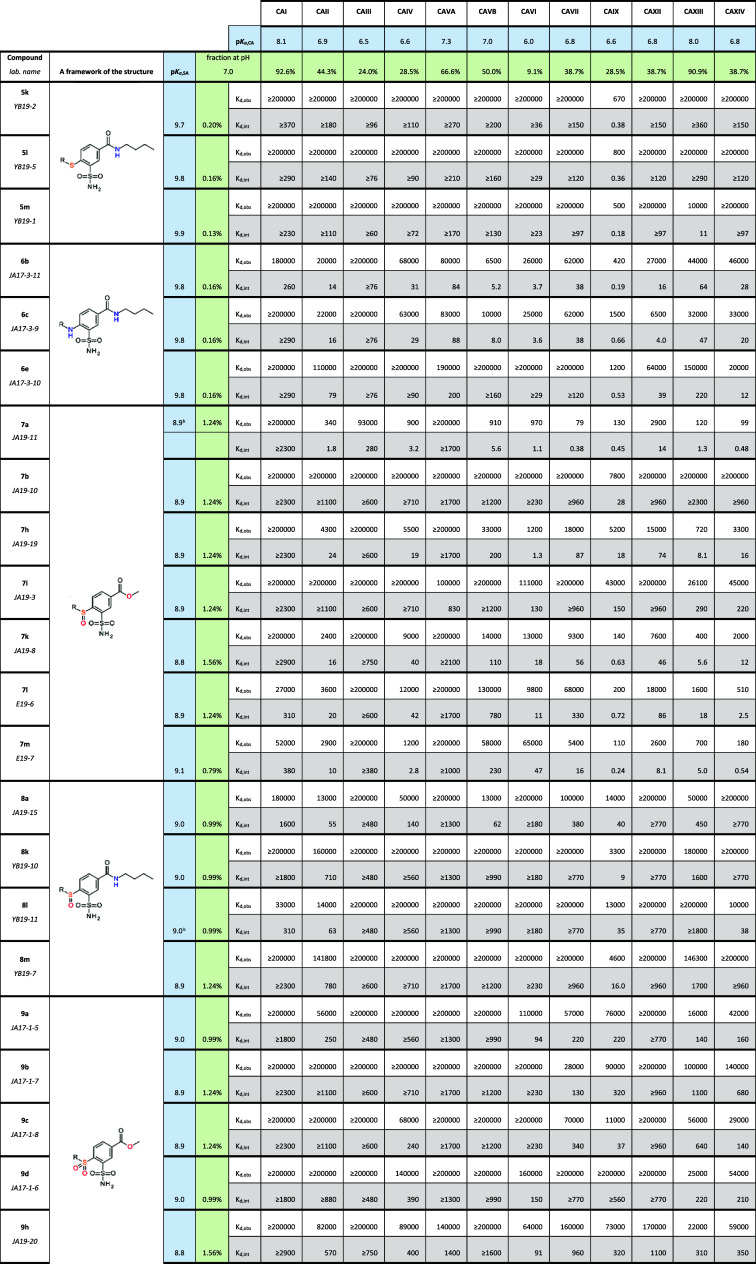

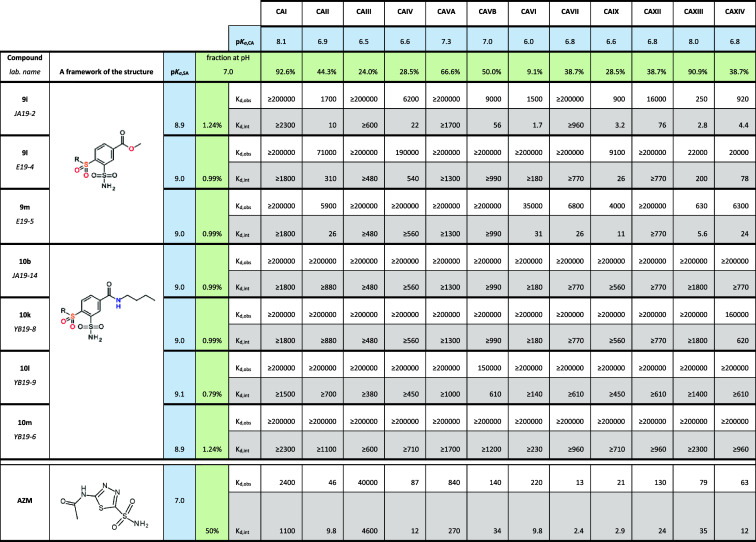
*Observed* and *Intrinsic* (*K*_d,obs_ and *K*_d,int_) Dissociation Constants (in nM Units) of Investigated Compounds to All Catalytically Active Human CAs at 37 °C Obtained by Fluorescent Thermal Shift Assay (FTSA)^a^

a Observed values were determined using 50 mM sodium phosphate buffer at pH 7.0 containing 100 mM sodium chloride, 50 μM ANS dye, and 2% (v/v) DMSO. *K*_d,int_s were calculated according to eq 1 (see the Experimental Section). Values with a “≥” sign show that they are at the detection limit of ≥200 000 nM of *K*_d,obs_ according to the highest used ligand concentration. The intrinsic value limits vary for different CAs and compounds due to differences in p*K*_a_. p*K*_a,SA_ – p*K*_a_ of the sulfonamide group; p*K*_a,CA_ – p*K*_a_ value of water molecule bound to Zn­(II) in the active site of CA; AZM, acetazolamide (a standard inhibitor).

b Not determined due to solubility issues or low intensity of the spectrum curves. The p*K*_a,SA_ value was assigned based on similarities in chemical structure.

As one of the important findings in this manuscript, Figure 2 arranges the compounds in the order of their affinity for CAIX and compared to undesired target CAII. A high affinity for CAIX is desired, because CAIX is implicated in various types of cancer.^24^ However, CAII is abundant in erythrocytes, thus an off-target for anticancer inhibitors.

**Figure 2:**
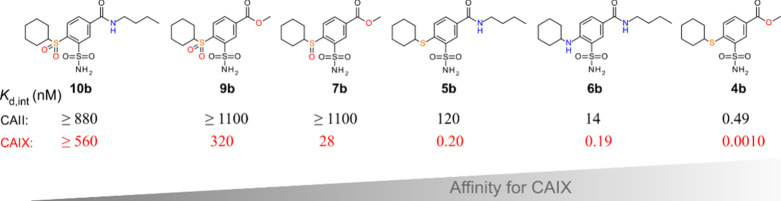
Compounds are arranged in the order of increasing affinity for CAIX. Compound **4b** had the highest affinity and selectivity for CAIX. The intrinsic dissociation constants (in nM units) are compared for CAII and CAIX, while values for the remaining CAs are listed in Table 1.

Compound affinities for human CA isozymes were analyzed using fluorescence-based thermal shift assay (FTSA) and the enzymatic activity stopped-flow-based inhibition assay (SFA) (Figure 3). The FTSA determined the observed dissociation constants for all compounds with all 12 CA isozymes (Table 1 and Supplementary Figure S1). From these experimentally determined *K*_d,obs_, the *intrinsic* dissociation constants *K*_d,int_ were calculated, and the results are primarily focused on them. Figure 3B,C shows two compounds with strong and weak affinity for CAII and CAIX by FTSA. A single oxygen atom strongly diminished affinity for both isozymes. The stopped-flow assay of CO_2_ hydration enzymatic activity inhibition confirmed that the compounds inhibited CAIX and CAII (Figure 3D,E). Inhibition *K*_i_ values are presented in Table 2 and Figure S2. The FTSA is a convenient technique covering a significantly wider *K*_d_ range than the SFA.^25^

**Figure 3:**
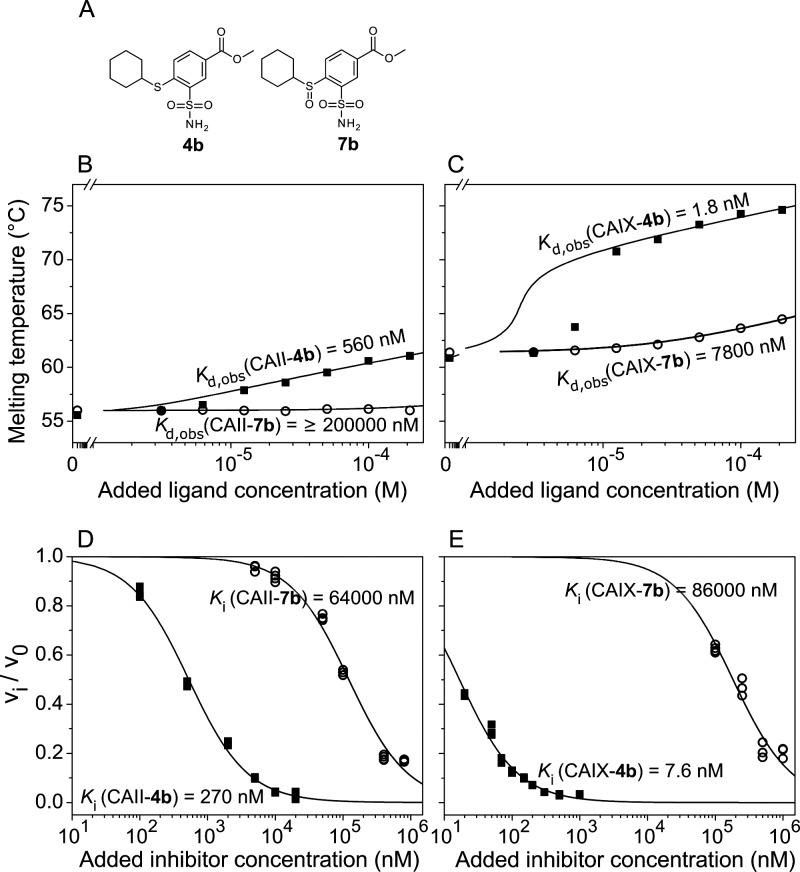
Compound affinity was determined by two assays in this study. (A) Chemical structures of two compounds whose binding data are shown below. (B) and (C) Fluorescent thermal shift assay data (FTSA) of compounds **4b** (closed squares) and **7b** (open circles) binding to CAII and CAIX, respectively. (D) and (E) Stopped-flow carbon dioxide hydration assay (SFA) data of compounds **4b** (closed squares) and **7b** (open circles) inhibition of CAII and CAIX, respectively. The dissociation constants (*K*_d_) or inhibition constants (*K*_i_) are given next to the corresponding curves. It is important to note that the experimental conditions of the methods were slightly different: FTSA, pH 7.0, 37 °C, while for SFA, pH 7.5, 25 °C.

**Table 2:** Inhibition Constants and IC_50_ (in nM Units) of CAII and CAIX with Compounds Obtained by Stopped-Flow Carbon Dioxide Hydration Assay (SFA) at 25 °C^a^

	*K*_i_ (nM)		*IC*_50_ (nM)	
compound	CAII	CAIX	CAII	CAIX
**4a**	25 ± 1.3	5.4 ± 0.5	47 ± 2.6	12 ± 0.5
**4b**	270 ± 20	7.6 ± 0.5	530 ± 40	16 ± 1.3
**4c**	210 ± 30	37 ± 3.5	400 ± 50	79 ± 7
**4d**	120 ± 10	51 ± 6	230 ± 20	110 ± 12
**4h**	9200 ± 500	1300 ± 200	18,000 ± 1000	2700 ± 400
**7a**	63 ± 6	360 ± 30	120 ± 12	760 ± 60
**7b**	64,000 ± 7500	86,000 ± 16,000	100,000 ± 10,000	200,000 ± 30,000
**7h**	3400 ± 200	7600 ± 700	6500 ± 300	16,000 ± 1600
**9a**	34,000 ± 4000	3700 ± 600	65,000 ± 7000	7800 ± 1300

a Experiments were performed using 20 mM HEPES Na at pH 7.5, 20 mM Na_2_SO_4_, and 0.2 mM Phenol Red.

The experimental conditions slightly differed between FTSA and SFA, and it is therefore not appropriate to directly compare the *K*_d,obs_ and *K*_i_. However, the measured affinities were similar in both techniques. Further analysis is based on FTSA data due to its wider limits in identifying strong binders. Note that the enzyme concentration limits the SFA’s ability to determine high-affinity binders. For example, if we use a 10 nM concentration of a CA isozyme, the lowest IC_50_ is 5 nM, half of the protein concentration. Any compound with an *IC*_50_ stronger than 5 nM would exhibit a dosing curve that appears as an *IC*_50_ of 5 nM. Thus, compounds that possess single-digit nM or picomolar *K*_d_, cannot be distinguished by SFA.

Sulfonamide binding of CA is a pH-dependent reaction.^6,26,27^ The water molecule bound to the CA zinc ion is replaced by the deprotonated form of sulfonamide upon binding.^28,29^ The protonation forms required for the interaction exist at different pHs: the CA-Zn­(II)-H_2_O has the largest fraction at acidic pH and the deprotonated sulfonamide has the largest fraction at alkaline pH. Therefore, the measured affinity is always lower than the intrinsic affinity. The intrinsic parameters are calculated (see equations in the Experimental section) by knowing the p*K*_a_ of the CA zinc-bound water and the p*K*_a_ of the sulfonamide group (Table 1 and Figure S3). Experimental data of the sulfonamide group p*K*_a_ determination for compound **4b** are shown in Figure 4. Figure S3 shows the graphs of p*K*_a_ determination for the remaining compounds.

**Figure 4:**
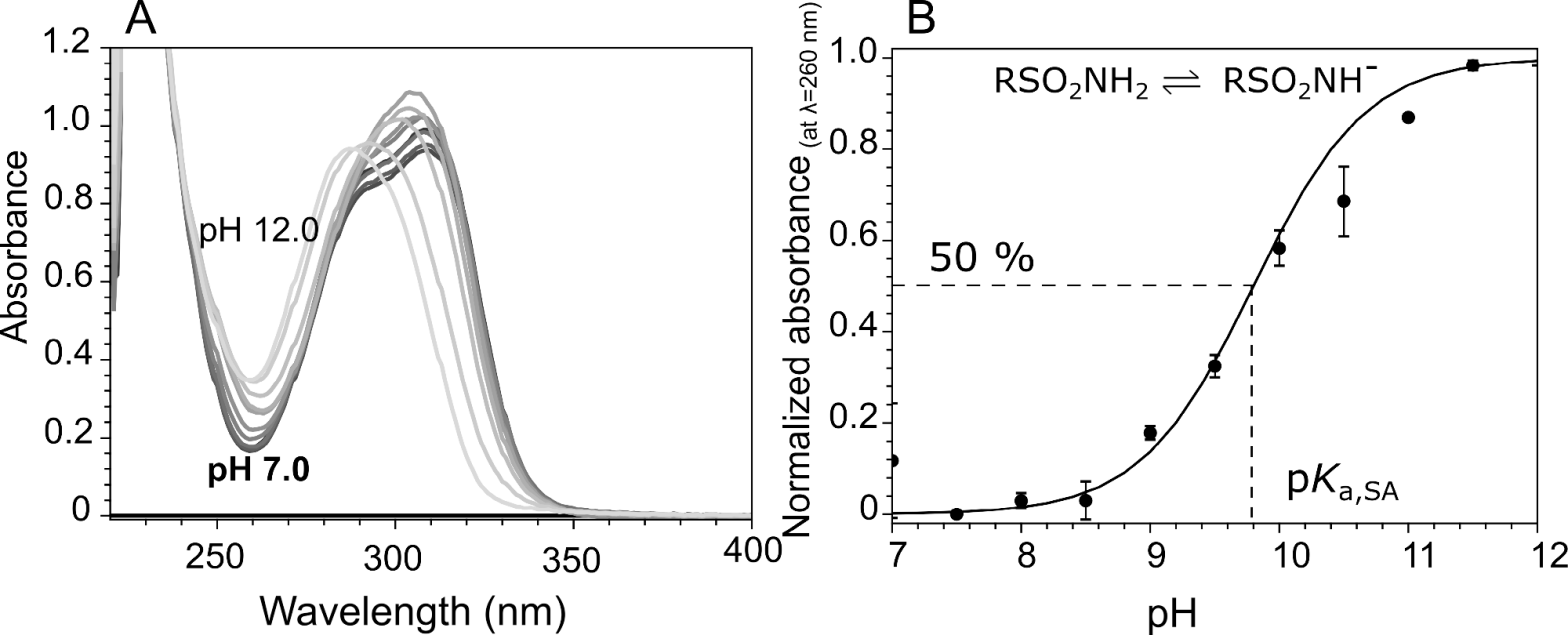
Spectrophotometric determination of the sulfonamide group deprotonation p*K*_a_ for compound **4b**. (A) Absorption spectra of compound **4b** in solutions at various pH at intervals of 0.5 pH units at 37 °C. (B) Normalized absorbance at 260 nm was plotted as a function of pH, and the p*K*_a_ value was determined as a midpoint of the curve. The data points are the mean points of two repeats ((A) shows one repeat for simplicity), with standard deviations. The p*K*_a_ value of compound **4b** was 9.74 ± 0.13 (±1.3%) with a confidence interval [9.62–9.87] of 95%.

Intrinsic affinities are especially important in rational drug design. It is not rare when a stronger affinity is observed not because of the formed bonds but because of the substitutions that lower the p*K*_a_ of the sulfonamide group and thus increase the fraction of the ready-to-bind form.^30^ Intrinsic parameters are used to avoid misleading conclusions when comparing the affinity of compounds for CAs.

#### Esters vs Benzamides

4-sulfanyl-substituted esters **4a**–**d**, **f–m** were the largest (12 compounds) studied group of compounds with the same framework. Compounds of this group, even almost independently of the substituent, interacted strongly and selectively with several CAs. *K*_d,int_ of **4b** for CAIX was 0.0010 nM and it was the strongest interaction measured in this study, CAXIII—0.090 nM and CAXIV—0.020 nM, with others interacting much weaker. The size of the substituent was critical in this interaction. CAIX has a larger active site than the rest of the CAs.^31^ It was interesting that compound **4g** (*adamantyl*), which has a similar affinity for CAIX (*K*_d,int_ = 0.0030 nM) to most of the compounds in this group, is a few hundred times more selective for CAIX, whereas, for example, **4b** (*cyclohexyl*) is only a few tens of times more selective for CAIX and can be considered a strong binder to several undesired CAs. On the other hand, the 4-methylsulfanyl- substituted compound **4h** is no longer selective. Thus, the hydrophobic interaction made a significant contribution to affinity for CAIX, and selectivity was mainly obtained by the size of the substituent when the binding to CAIX was optimal and binding to other CAs was limited by steric interference. Analogous benzamides were much weaker binders of CAs. Most benzamides exhibited no interaction with CAs. Nevertheless, all compounds in this series bound to CAIX and were, in most cases, at least several dozen times more selective for it than for other CAs.

#### Sulfanyl vs Sulfinyl vs Sulfonyl Compounds

Different forms of sulfur oxidation led to drastically different affinities for CAs. A higher degree of oxidation in these compounds weakened the affinity. However, in our opinion, it was not the oxidation itself that had the main influence, but rather the conformation of the compound. The affinity of all sulfinyl- compounds with all CAs was significantly weaker than analogous sulfanyl- compounds. The decrease in affinity varied depending on the CA isozyme and the substituents. Therefore, no generalized observations could be made. For example, compound **7a** did not bind to CAI and CAVA, but the weak affinity was determined for CAIII, with all other CAs the interactions were similar and did not exceed more than 10-fold in most cases and there was no selectivity for CAIX. Compounds **7b**, **7h**, and **7i** did not bind or bind weakly and nonselectively to all CAs. Except for **7b**, it is bound only to CAIX with 28 nM. The *K*_d,int_ of compounds **7k**, **7l**, and **7m** were 0.63, 0.72, and 0.24 nM, respectively. Also, there was a similar affinity for CAXIII and CAXIV and weaker for the other CAs. From compounds **5­(a–d, f, g, j–m)** to **8­(a, k–m)** decreased affinity for all CAs.

Switching to sulfonyl compounds reduced the affinity and abolished the selectivity for CAIX. **9b** only bound to CAVII, CAIX, CAXIII, and CAXIV with *K*_d,int_ 130, 320, 1100, and 680 nM, respectively. The most strongly interacting compound in this series was **9i**, e.g., *K*_d,int_ of CAVI—1.7 nM, CAIX—3.2 nM but also bound to other CAs quite similarly. Meanwhile, compounds **10­(b, k–m)** did not bind to any isozymes, only a couple of measurements showed a weak interaction.

#### 4-Sulfanyl- vs 4-Amino-Substituted

Comparing **5b** vs. **6b** and **5c** vs. **6c**, in most cases, the dissociation constants differed only a few times, and the constants for CAIX did not exceed the margin of error. The S or N atom in the same position of the compound almost did not change the affinity for all isozymes. Compound **6e** (cyclooctyl-substituted) selectively interacted with CAIX, *K*_d,int_ of CAIX—0.53 nM, CAXII—39 nM, and CAXIV—12 nM. It did not bind to other CAs. Most likely the selectivity was due to the size of the active site pocket.

### X-ray Crystal Structures of Compound Binding to CAII and CAXIII

Nine crystal structures were determined by X-ray crystallography, the complexes of CAII with compounds **2**, **3**, **4c**, **4d, 4h**, CAIX with **4d** and **5b**, and CAXIII with **4c** and **4d**. Table 3 lists the data collection and refinement statistics. Figure 5 shows the electron density maps of these compounds in the active site of CAs. Two molecules of compound **4h** were identified in the active site of CAII, one conventionally formed a coordination bond between the sulfonamide group and zinc, and the other was independently located near the periphery of the active site. The localization of both separated molecules relative to the same zinc is shown in Figure 5E,F. The main highlights of the identified protein–ligand interactions are described below and illustrated in Figure 6.

**Table 3:** X-ray Diffraction Data Collection and Refinement Statistics

isozyme—ligand	CAII—**2**	CAII—**3**	CAII—**4c**	CAII—**4d**	CAII—**4h**	CAIX—**4d**	CAIX—**5b**	CAXIII–**4c**	CAXIII–**4d**
PDB ID	9FPT	9FPU	9FPQ	9FPR	9FPS	9R8X	9R8Y	9FPV	9FPW
Data Collection Statistics									
space group	P12_1_1	P12_1_1	P12_1_1	P12_1_1	P12_1_1	H3	H3	*P*2_1_2_1_2_1_	*P*2_1_2_1_2_1_
unit-cell parameters (*a*, *b*, *c* (Å); α, β, γ (°))	*a* = 42.1, *b* = 40.9, *c* = 71.7, α = γ = 90, β = 104.0	*a* = 42.0, *b* = 41.1, *c* = 71.9, α = γ = 90, β = 104.2	*a* = 42.3, *b* = 41.3, *c* = 72.2, α = γ = 90, β = 104.2	*a* = 42.3, *b* = 41.3, *c* = 72.1, α = γ = 90, β = 104.5	*a* = 42.3, *b* = 41.2, *c* = 72.0, α = γ = 90, β = 104.4	*a* = *b* = 152.5, *c* = 172.4, α = β = 90, γ = 120	*a* = *b* = 151.9, *c* = 173.7, α = β = 90, γ = 120	*a* = 55.1, *b* = 58.0, *c* = 160.2, α = β = γ = 90	*a* = 55.5, *b* = 58.4, *c* = 160.7, α = β = γ = 90
resolution range (Å)	69.6–1.2	40.7–1.1	70.0–1.5	40.0–1.5	39.9–1.4	47.95–2.0	47.8–1.95	80.1–1.7	40.2–1.9
unique reflections number	75321	87594	38824	41447	47284	101012	108927	57402	42102
*R*_merge_, overall (outer shell)	0.04 (0.38)	0.05 (0.31)	0.05 (0.25)	0.05 (0.39)	0.04 (0.38)	0.08 (1.18)	0.21 (1.30)	0.09 (0.32)	0.12 (0.31)
I/σ overall (outer shell)	16.9 (3.5)	13.7 (3.8)	15.9 (4.9)	19.6 (4.6)	24.2 (4.7)	21.1 (2.3)	7.1 (1.5)	16.8 (7.4)	13.0 (7.6)
multiplicity overall (outer shell)	6.8 (6.7)	6.6 (6.0)	6.6 (6.4)	7.0 (6.9)	7.0 (6.8)	10.4 (10.5)	10.4 (9.9)	13.2 (13.1)	13.1 (13.4)
completeness (%) overall (outer shell)	96.7 (94.0)	96.0 (91.3)	99.6 (99.2)	98.7 (97.7)	97.6 (94.9)	100.0 (100.0)	100.0 (100.0)	100 (100)	100 (100)
Wilson B-factor	12.8	11.4	17.8	15.5	17.0	28.0	22.4	16.9	22.5
Refinement Statistics									
*R*_work_	0.14	0.15	0.17	0.14	0.14	0.17	0.21	0.16	0.19
*R*_free_	0.18	0.17	0.20	0.21	0.19	0.21	0.25	0.19	0.23
RMSD bond lengths (Å)/ angles (°)	0.02/2.13	0.02/2.26	0.01/1.80	0.01/2.04	0.01/2.02	0.01/1.81	0.01/1.73	0.01/1.90	0.01/1.57
average B factors (Å^2^): all atoms/inhibitors	22.7/17.2	19.6/16.9	21.6/21.9	20.1/15.7	21.8/21.7	31.9/50.4	30.7/39.4	21.6/32.5	25.9/35.6
Ramachandran statistics (%): favored/allowed/outliers	97/3/ 0	97/3/ 0	97/3/ 0	96/4/ 0	96/4/ 0	96/4/ 0.11	94/5/ 0.32	chain A: 98/2/ 0 chain B: 97/3/ 0	chain A: 97/3/ 0 chain B: 97/3/ 0

**Figure 5:**
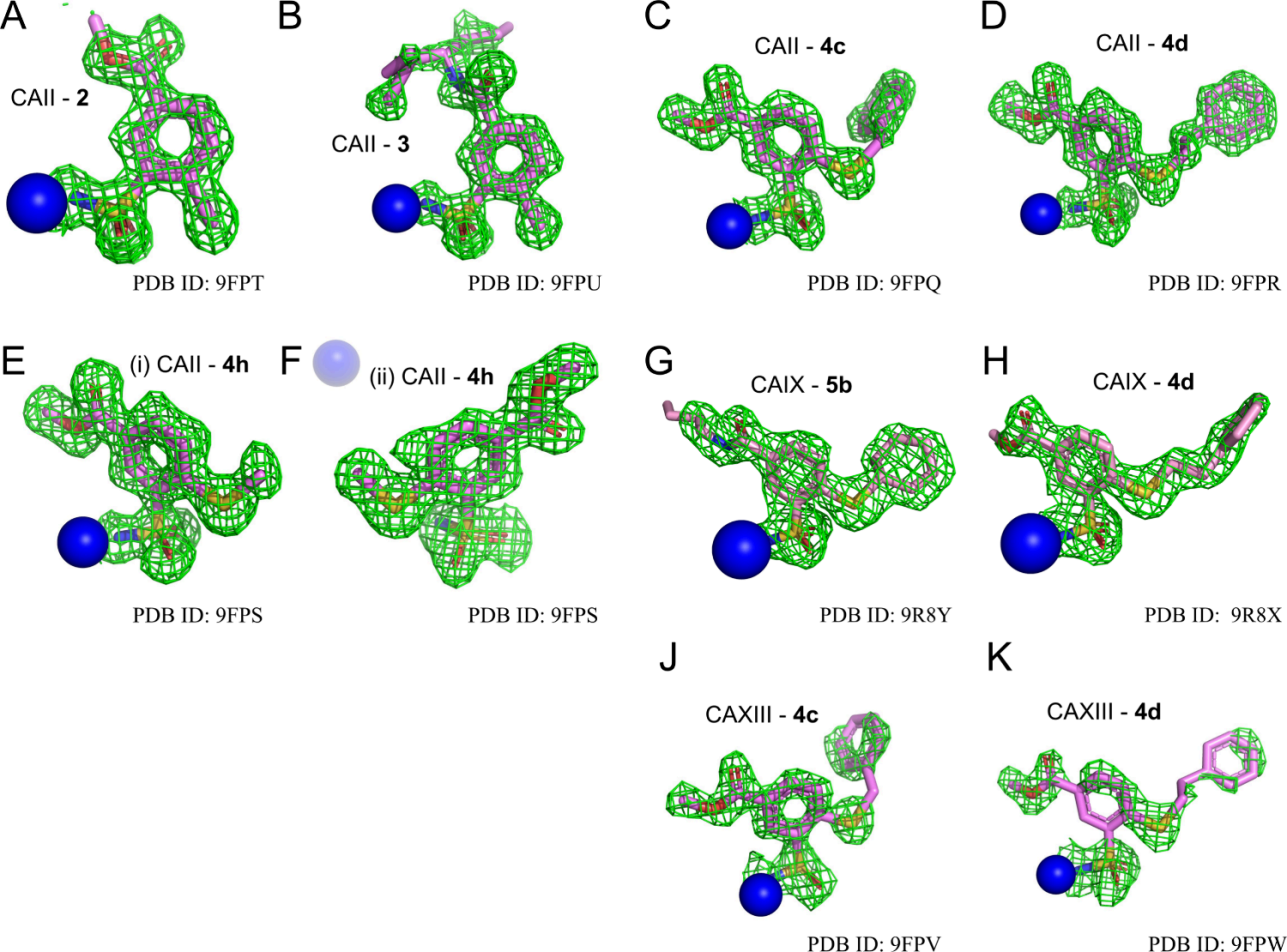
|*F*(*o*) – *F*(*c*)| omit maps at 3σ in green for the investigated ligands in the active site of CAs. (A) CAII—**2** (PDB ID: 9FPT), (B) CAII—**3** (PDB ID: 9FPU), (C) CAII—**4c** (PDB ID: 9FPQ), (D) CAII—**4d** (PDB ID: 9FPR), (E) and (F) CAII—**4h** (PDB ID: 9FPS; two ligand molecules were identified, one bound directly to Zn in a conventional position, while the second was seen located nearby toward the edge of the active site), (G) CAIX—**5b** (PDB ID: 9R8Y), (H) CAIX—**4d** (PDB ID: 9R8X), **(J)** CAXIII—**4c** (PDB ID: 9FPV), **(K)** CAXIII—**4d** (PDB ID: 9FPW). Omit maps were taken from a refinement run of the final model without the ligand. Zinc is shown in blue.

**Figure 6:**
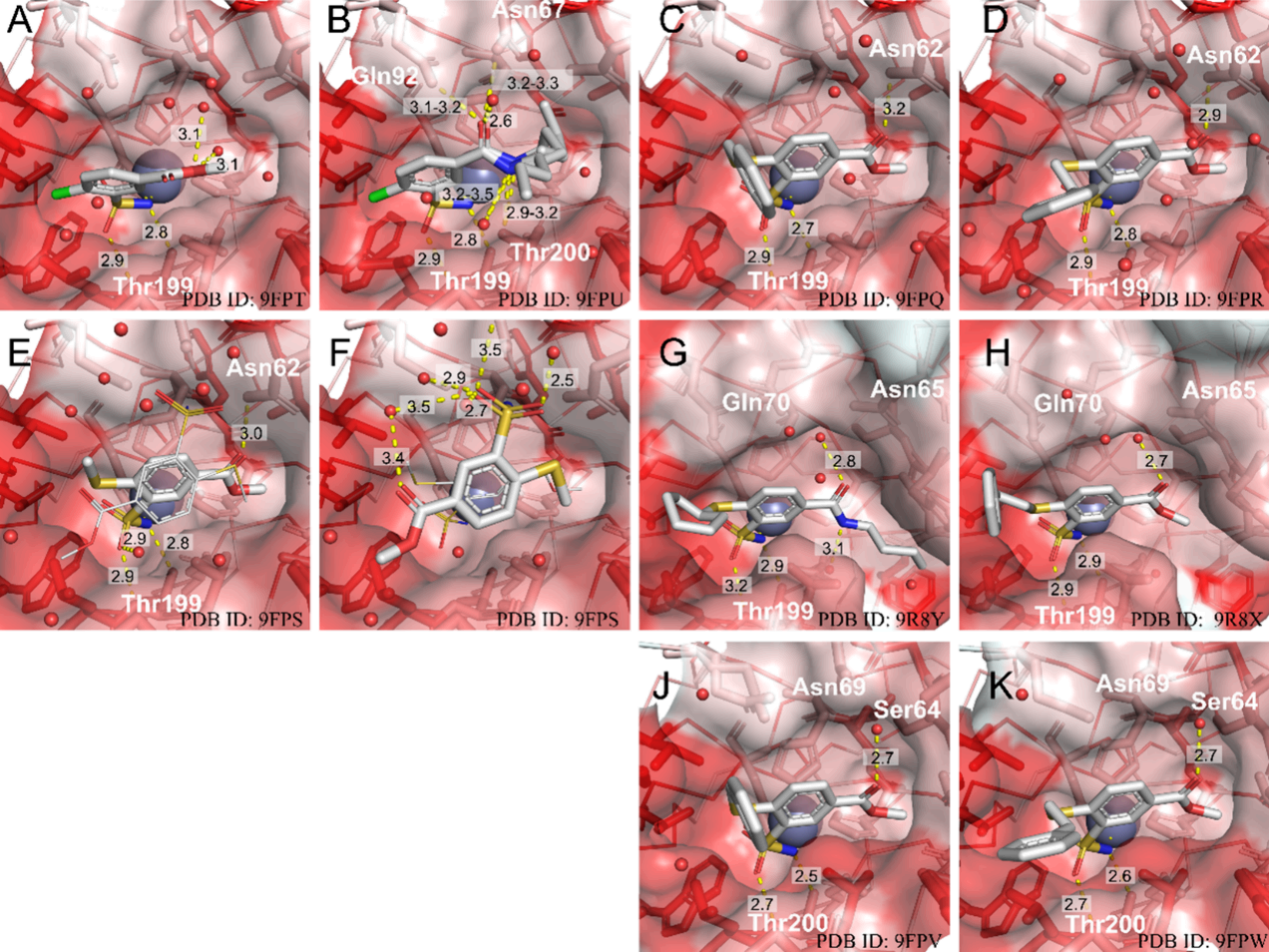
X-ray crystal structures of (A) CAII—**2** (PDB ID: 9FPT), (B) CAII—**3** (PDB ID: 9FPU), (C) CAII—**4c** (PDB ID: 9FPQ), (D) CAII—**4d** (PDB ID: 9FPR), (E) and (F) CAII—**4h** (PDB ID: 9FPS; two ligand molecules were identified, one bound directly (E) to Zn in a conventional position, while the second is seen located nearby toward the edge (F) of the active site), (G) CAIX—**5b** (PDB ID: 9R8Y), (H) CAIX—**4d** (PDB ID: 9R8X), **(J)** CAXIII—**4c** (PDB ID: 9FPV), **(K)** CAXIII—**4d** (PDB ID: 9FPW). The yellow dashed line represents the hydrogen bond; the distances are given in angstroms. The amino acids directly involved in hydrogen bond formation are labeled. Amino acids are colored according to hydrophobicity:^32^ the most intense red color represents the most hydrophobic amino acids.

#### Structures of **2** vs **3** Bound to CAII

Starting compounds **2** and **3** used in the synthesis differed by one substitution at the *meta* position relative to the sulfonamide group, ester vs. amide. Figure 6A,B shows the position of these compounds in the active site of CAII. Both compounds retained the same position of the sulfonamide group and the chlorine atom in the active site of CAII. The amide substituent formed multiple hydrogen bonds with the amino acid side groups, while the ester was stabilized by a network of hydrogen bonds through water molecules. Presumably, a different network of hydrogen bonds pulled the entire molecule slightly, so a partially rotated benzene ring was observed in the crystal structure. No significant conformational changes were observed in the amino acid chains of the active site of CAII. However, the amide substituent of the compound was not in one fixed position. Instead, 3 alternative conformations of the substituent were identified in the structure. However, it should be noted that this substituent has a poor electron density, likely due to its high flexibility. Consequently, its exact arrangement cannot be determined.

#### Structures of CAII with Chlorine-Substituted (**2**, **3**) vs Sulfanyl-Substituted (**4c**, **4d**, and **4h**)

The position of *ortho-sulfanyl* and chlorine-substituted compounds in the CAII active site differed significantly. The entire sulfanyl-substituted molecule was shifted to avoid steric interference but maintained a similar distance between the sulfonamide group’s nitrogen and the enzyme’s zinc compared to the chlorine-substituted compound. Meanwhile, the studied *ortho*-substituted compounds occupied similar positions, and the ester groups of all three compounds formed a hydrogen bond with Asn62. Notably, the electron density of all inhibitor molecules is well-defined.

#### Structures of CAII vs CAIX and CAXIII with Bound Sulfanyl-Substituted (**4c** and **4d** or **5b**)

Figure 6 shows the interactions between CAII with compounds **4c** and **4d** (Figure 6C,D), CAIX with **4d** (Figure 6H), and CAXIII with compounds **4c** and **4d** (Figure 6J,K). One of the main differences was in the formed hydrogen bonds. The oxygen of the ester group of the compound formed a direct hydrogen bond with Asn62 in the active site of CAII. Meanwhile, a hydrogen bond is formed in the active site of CAIX and CAXIII through a water molecule with Gln70 and Asn65 or Ser64 and Asn69, respectively. Asn62 in CAII, Asn65 in CAIX, and Asn69 in CAXIII differ only by the numbering but correspond to the same position.

Incidentally, compound **5b** CAIX also forms the same hydrogen bond through a water molecule, and the entire molecule adopts a similar conformation. Notably, the electron density of the 17 amino acids at the N-terminus of both X-ray crystal structures of CAIX is not defined as expected. These amino acids fold in their 3D structure to form the active site of the protein. This side of the active site is called the hydrophilic part. This suggests that the bound ligand pushes these amino acids to fit fully into the active site. When comparing these structures (PDB ID: 9R8X and 9R8Y) with those existing in the PDB (e.g., PDB ID: 3iai), the clash between ligand and Tyr7 is seen without changing their arrangement.

It is important to emphasize that both **4c** and **4d** have very poor electron density in the CAXIII active center, so the arrangement of the compound should be evaluated more cautiously. On the other hand, a comparison of the structures shows that compound **4d** is more similarly located in the active sites of CAII and CAXIII, while in CAIX, it is shifted toward the hydrophilic side, which, as mentioned, is not fully visible in the X-ray structure.

#### Atypical Binding Position

The structure 9FPS was unique because two ligand molecules were identified as bound in the active site. One molecule was bound classically and formed a coordination bond with the zinc ion. The second formed a hydrophobic interaction with the first molecule, and hydrogen bonds with water molecules located toward the edge of the active site. This could be a crystallographic artifact or a secondary interaction with the protein.^33^ A previous publication^2^ identified a similar case with CAIX (PDB ID: 6QUT) (Figure 7). In that case, the molecules interacted in the active site and among themselves between the chains in an asymmetric unit. This case with CAII was different because the two chains had no interaction between ligand molecules. The active sites of symmetric chains were not oriented face-to-face. The atoms of the second nonclassical ligand were further than 5Å from the amino acids of the symmetric chain.

**Figure 7:**
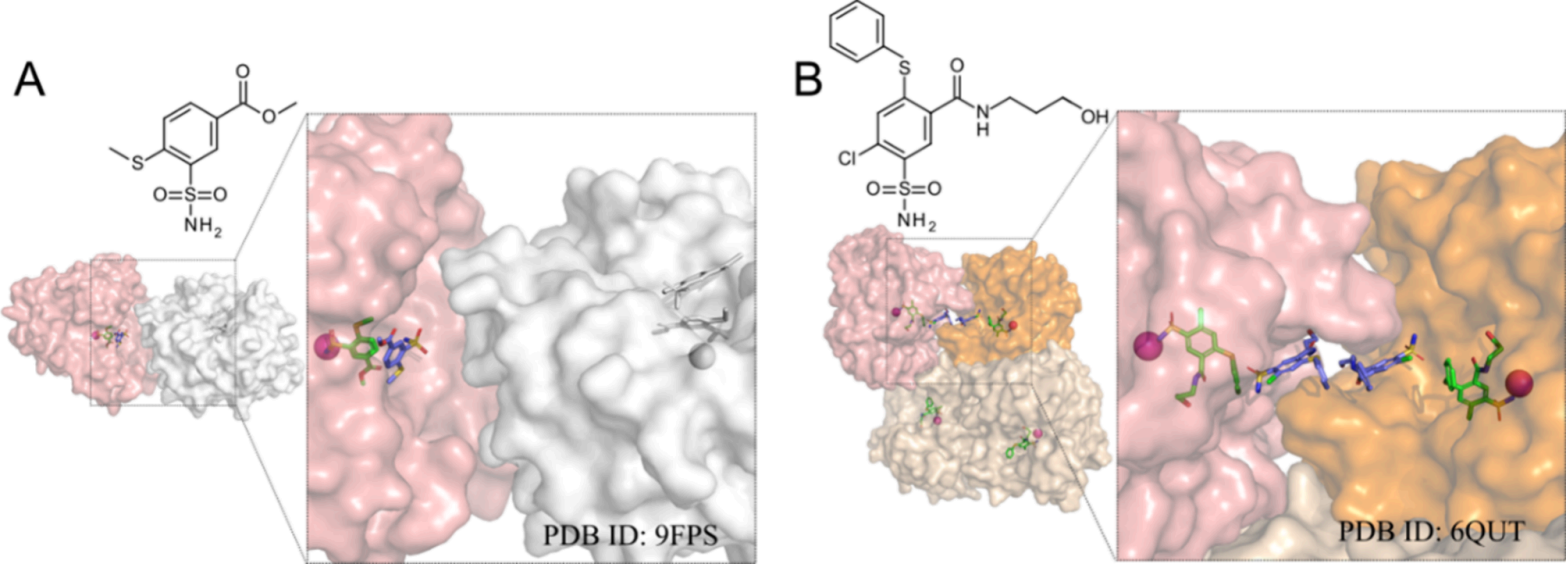
Unusual ligand positions in crystal structures of CAII (PDB ID: 9FPS) and CAIX (PDB ID: 6QUT, published previously^2^). The chemical structures of the ligands are shown above the crystal structures. The crystal structures represent a view of the monomers and a zoomed-in view of the active site. Zn­(II) is shown as a pink sphere. Bound to Zn­(II) inhibitor molecules are green and others are blue. (A) Monomer of CAII is shown in surface mode and colored salmon. The symmetric chain is colored white. (B) In the case of CAIX, only the asymmetric unit is represented. Six inhibitor molecules are bound to 4 CA IX chains. Chain C (orange) and chain D (salmon) have two inhibitor molecules, and chains A and B (beige) have one molecule. In this case, the interaction of ligands between separate chains is visible.

### Molecular Docking

To understand the differences in the binding affinities of series **4**, **7**, and **9** ligands, they were docked into CAII, except for **4e** and **4f**, containing large, flexible rings that are challenging to dock. Series **4** ligands were also docked into CAIX and CAXIII, to compare with crystal structures and thus assess the docking accuracy. To reduce bias, different receptors (PDB IDs – CAII: 3HS4; CAIX: 6G9U, chain A; CAXIII: 4KNN, chain A) were chosen instead of the new X-ray structures presented in this paper.

To mimic the donor–acceptor bond between the zinc ion and the sulfonamide nitrogen of the ligand, we employed constrained docking using the Smina program.^34^ The constraint forced the sulfonamide nitrogen to maintain its original position in the X-ray structure. The generated poses were afterward rescored using the Vinardo scoring function.^35^ Vina^36^ and GNINA^37^ Machine-learning-based scoring functions were found to be inferior to Vinardo for this system, and therefore only the latter was employed for further analysis. The pose with the best rescored affinity that matched the X-ray conformation of the benzenesulfonamide moiety was then chosen as the representative best docked structure (in all cases, for ligand **4** series, it was ranked 1 by Vinardo, except for **4j**, where the correct benzenesulfonamide conformation was ranked 3). The best docked poses for CAII are shown in Figure 8A. The Pearson correlation coefficient *R* with the experimental *intrinsic* binding affinities to CAII for the best docked poses was 0.87. Figure 8B plots the corresponding computed and experimental binding affinities for CAII and CAIX (values listed in Table S1 in the Supporting Information). Notably, for CAII, the scoring function correctly predicts the best binder, **4m**, and the four worst binders (**4g**–**j**). The panel also shows the correspondence between the experimental and predicted binding affinities for CAIX, with *R* = 0.66.

**Figure 8:**
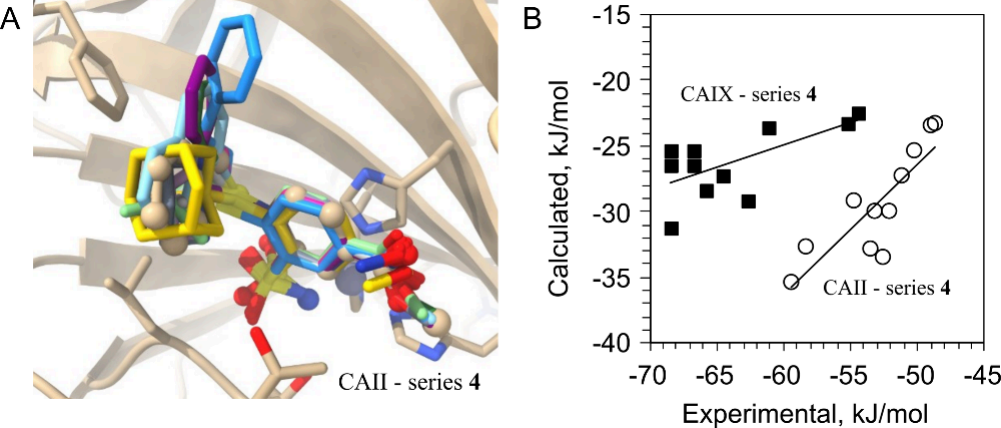
(A) Docked series **4** ligands (see main text for details) superposed onto the X-ray structure of **4c** (PDB ID: 9FPQ; rendered using ball-and-stick representation) in a complex with CAII. Note that hydrophobic substituents stack against the Phe131 side chain. (B) Computed ligand binding affinities to CAII (circles) and CAIX (squares) using Vinardo scoring function plotted against the experimental binding affinities—intrinsic Gibbs energy changes. The Pearson correlations R of the plotted points are 0.87 for CAII and 0.66 for CAIX. The large difference between the computed and experimental binding affinities is because the Vinardo scoring function does not take into account the interaction with zinc. However, it can be assumed to be approximately equal for all ligands.

#### Validation of the Docking Protocol via Comparison with the X-ray Structures

The proposed docking and scoring protocol was further validated by comparing the predicted docked poses of some of the ligands with their conformations in the newly reported X-ray structures. Figure S4A–C shows the ranked poses of ligands **4c**, **4d**, and **4h** compared against their X-ray conformations in the complex with CAII. The heavy atom Root Mean Square Deviation (RMSD) between the docked and X-ray conformation is 2.03, 3.13, and 0.46 Å, respectively. For the first two ligands, however, the rank 2 conformations are close to the X-ray conformation (**4c**: 0.61 Å; **4d**: 0.67 Å, see Figure S4A,B).

To further validate the chosen docking protocol, **4d** and **5b** were docked into CAIX (PDB ID: 6G9U, chain A). The best-scored poses after reranking using Vinardo are shown in Figure S5. The corresponding heavy atom RMSDs for **4d** and **5b** are 2.18 and 0.92 Å, respectively, and despite the RMSD for **4d** being above 2 Å, the binding mode is captured by the docking reasonably well, especially since the receptor used for docking was originally bound to a ligand from a chemically different series.

Compounds **4c** and **4d** were also docked into the CAXIII receptor (PDB ID: 4KNN, chain A). Similarly to CAIX, the docking protocol picked the docked pose with the approximately correct binding mode (with RMSDs equal to 1.90 and 0.95 Å, respectively) (Figure S6).

#### Docking of Series **7** and **9** Compounds

Since series **4** compounds using the docking protocol described above can reproduce the scaffold rather well and generally seem to stack the hydrophobic substituents reasonably, we applied a similar procedure by docking series **9** ligands into CAII as a model protein, in hopes that it will help to explain a difference between the binding affinities of series **4** and **9**. Docking using the same protocol led to comparable binding affinities between the two series (not shown). However, experiments indicate that **9** binds worse by several orders of magnitude, and rescored constrained pose affinities for this series exhibit practically no correlation with the experiment (*R* ≅ 0.12). A careful examination of the docking results for series **9** revealed the source of the poor binding affinities and the poor performance of the docking score. Figure 9A displays the best ranking pose of **9a**. While the phenyl substituent of **9a** stacks well with Phe131 side chain, the two sulfonyl moieties exhibit an apparent clash against each other.

**Figure 9:**
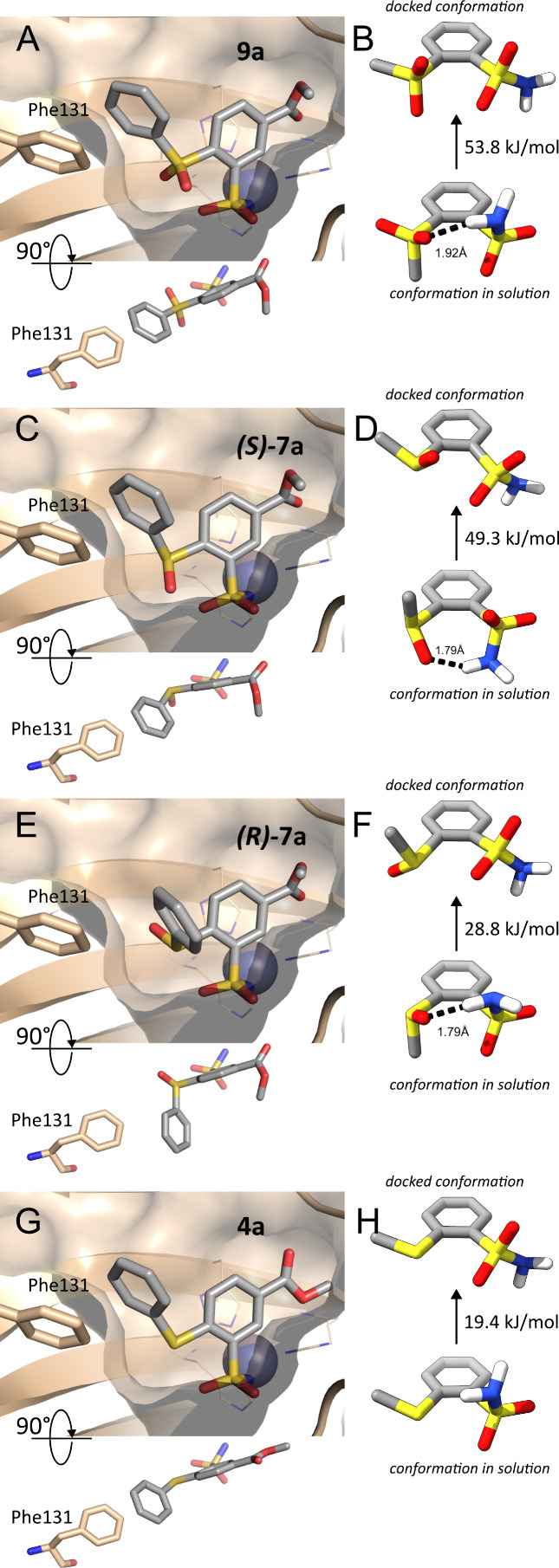
Best ranking docked pose of **9a**, (S)-**7a**, (R)-**7a**, and **4a** in the active site of CAII as a model protein. (A) Best-ranking docked pose of **9a**. While the phenyl substituent stacks well with the Phe131 side chain, the two SO_2_ moieties presumably clash. (B) Two conformations of 2-(methylsulfonyl)­benzenesulfonamide: Top: conformation with two benzene-to-S bond dihedrals constrained so that it is similar to the docked conformation in (A); bottom: lowest energy conformation with an intramolecular H-bond that is likely to be found in solution. The energy difference between the two rotamers computed using density functional theory is 53.8 kJ/mol. The fact that the used scoring function does not take into account this difference explains why the calculated affinities (not shown) do not match the experimental values. (C), (E) Best-ranked docked conformations of *(S)-* and *(R)-* **7a**, correspondingly. (D), (F) Top: the conformations 2-(methylsulfinyl)­benzenesulfonamide, matching the docked geometries in (C) and (E). Bottom: The lowest energy conformer with an intramolecular H-bond, presumably existing in solution. The docked *(R)-* enantiomer is much more stable due to the lack of clashes present in the docked *(S)-* enantiomer. (G) Best-ranking docked pose of **4a**. (H) Two conformations of 2-methylsulfanylbenzenesulfonamide: Top: the optimized with constraints conformation matching the docked conformation; bottom: The lowest energy conformer. Compared with the sulfinyl- and sulfonyl-forms, the docked conformation for the sulfanyl analog is the most stable, which is also reflected in the binding affinities.

To further explore this clash, we ran simulations of a simple compound 2-(methylsulfonyl)­benzenesulfonamide containing sulfonamide and methylsulfonyl groups at the *ortho*-positions on a benzene ring. The lowest energy conformer for this compound was generated using CREST software^38^ using semiempirical GFN2-xTB wave function,^39^ and afterward reoptimized in the implicit solvent using the Density Functional Theory (DFT) approach with the GAMESS-US program.^40^ In the lowest energy conformer, sulfonamide nitrogen forms a hydrogen bond with the sulfonyl oxygen at the *ortho* position (Figure 9B, bottom). The approximate conformational energy of the docked conformation was also computed using DFT, keeping frozen the sulfonamide S–N and methylsulfonyl S–C bond torsional angles for the benzene ring, and optimizing the rest of the 2-methylsulfonylbenzenesulfonamide molecule (Figure 9B, top). The freezing of the select torsions was necessary because the DFT calculation lacks the protein environment that keeps them at certain values seen in docking. The energy difference between the lowest energy and the docked conformers in Figure 9B was 53.8 kJ/mol. It became clear that the used scoring function did not report a correct binding energy of the conformation in Figure 9A because the Vinardo scoring function neither takes into account the change of the ligand conformation when going from the solution into the receptor, nor the ligand intramolecular energy (in fact, none of the several built-in scoring functions in Smina do). This means that in the conformation shown in Figure 9A the 2-methylsulfonyl group must rotate to avoid the rather severe clash with the sulfonamide oxygens (Figure 9B, top), and by doing so the hydrophobic substituent rotates away from Phe131, potentially yielding other clashes with the protein and leading to the poor binding energies.

Series **7** compounds are more complex to investigate because two enantiomers of the S atom of sulfinyl exist (Figure 9C,D). We will explore the behavior of sulfinyl-containing series **7** using compound **7a** as an example. One of its docked enantiomers, *(S)*-**7a** forms sulfonyl-sulfinyl clash (Figure 9C and the top left part of Figure 9D) similar to what we found for **9a**. The docked *(R)*-**7a** enantiomer is about 1.7 times more stable compared to the *(S)-* enantiomer because of the lack of the clash (Figure 9D,F). Calculations also show that in the absence of the protein, the preferred conformation **7a** (of either chirality) has an intramolecular H-bond (Figure 9D,F, bottom).

For comparison, we also used DFT calculations of the 2-methylsulfanylbenzenesulfonamide molecule to estimate the stability of the docked **4a** (Figure 9G), which does not seem to exhibit clashes. Comparison of Figure 9B,D,F,H shows that the docked conformer **4a** is ∼1.5 times more stable than the most stable docked conformer of **7a** and nearly 3 times more stable than docked **9a**, corresponding to the experimentally determined binding affinities that are best in series **4**, followed by **7** and **9**. Interestingly, calculations suggest that one reason for the relative stability of docked **4** is the lack of the intramolecular hydrogen bond in solution (Figure 9H, bottom), and this observation could be potentially used in designing better binders for carbonic anhydrases, or even other receptors.

## Discussion

Recognition of the pockets on the protein surface by small-molecule ligands is still rather poorly understood, and it is not possible to accurately calculate the thermodynamic binding parameters.^41^ Primary sulfonamide compounds are known to inhibit CA isozymes by binding to the catalytic Zn­(II). Chemical variation of the remaining molecule often has a limited influence on binding affinity and selectivity. However, some chemical changes made near the sulfonamide group have a significant impact and may change the behavior from strong binder to completely undetectable binding. To investigate this phenomenon, we synthesized *ortho*-substituted benzenesulfonamides, determined their binding affinity to all catalytically active isoforms of human carbonic anhydrases, obtained crystal structures with CAII, CAIX, and CAXIII, and performed molecular docking. Different oxidation forms of the linker at the *ortho* position were used: −S–, −SO–, and −SO_2_–. Compounds with −SO– or −SO_2_– linker were much weaker binders to any CA isozyme than compounds with the −S– linker. Several reasons could cause this. First, a substituent with a higher oxidation state exhibits a stronger electron-withdrawing effect, thus lowering the p*K*_a_ of the amine of the sulfonamide group and causing stronger binding. Second, the linker oxygen atoms may prevent the easier rotation of the ligand molecule bonds and prevent conformational changes needed for the ligand to adapt to the protein surface. Third, the oxygen atoms take additional space, and the steric hindrance could be an essential factor in diminishing the binding affinity.

To test the electron-withdrawing effect, the p*K*_a_ values of compounds were determined experimentally. The p*K*_a_ of compounds containing −S– linker was almost an entire pH unit higher than compounds with linkers −SO– or −SO_2_–. Sulfonamides bind to CA in their negatively charged deprotonated form.^42^ Therefore, the lowering of sulfonamide p*K*_a_ increases the fraction of the binding-ready deprotonated sulfonamide and thus increases the observed affinity. This effect is simply the effect of compound availability in the proper form. To eliminate this misleading increase in affinity, we subtract the fraction effects and calculate the *intrinsic* affinity. The intrinsic dissociation constants of all compounds are provided next to the observed values in the table. In all cases, the intrinsic affinities are higher than the observed ones. The intrinsic values show the ‘real’ affinities between the binding components in the binding-ready protonation state. These values should be used in drug design to explain the structure–function relationship of the compound effects and not the experimentally observed ones. However, the experimentally observed affinity values show the affinities observed by any experimental technique, and the values are biologically relevant. They should be used to calculate the bound fractions and drug effect at particular conditions.

Furthermore, the compounds with a higher oxidation state (**7a, b, h, i, k–m** and **9a–d, h, i, l, m**) did not bind or bind with lower affinity than compounds **4a–d, f–m**. Even the compounds **7h** and **9h** with the smallest methyl substituent did not match the sulfanyl compound **4h** in affinity. The main reason for this was the allowed conformations of the compounds, which were calculated using quantum Density Functional Theory (DFT). The oxygen atoms limited both the flexibility of the molecule in finding the optimal position and acted as a steric hindrance. The present study shows that the *ortho* modifications had a more significant effect than the *para* variations.^21^

The highest affinity for CAIX in this study was exhibited by compound **4b** (methyl 4-cyclohexylsulfanyl-3-sulfamoylbenzoate). Previously we have designed similar compounds bearing 2-chloro or 2-bromo substituents that exhibited slightly higher affinity for CAIX.^20^ Here, we have intentionally omitted the halogen atoms to synthesize the *ortho*-substituted compounds more easily and explore the binding without halogens. It was also easier to synthesize compounds with an amide substituent in the *meta* position. The length of these amide-substituted compounds was of limited importance in the previous study but had a significant influence on this study, likely due to reasons of steric hindrance.

Sulfonamide compounds usually bind to CA isozymes by forming a coordination bond between the Zn­(II) and sulfonamide amino groups. This bond significantly increases the affinity, but is not necessary for binding to occur.^43^ Removal of the metal or change of the metal with another demonstrated that the coordination bond contribution is additive and metal-dependent. Strongly binding compounds like brinzolamide to CAII also bind to the Zn-free apoCAII. The energy contribution of the coordination bond could be determined by using a metal-exchange approach.

In the crystal structure of compound **4h** bound to CAII, two well-resolved compound molecules were bound in the active site. The first was bound in a conventional way forming a coordination bond with the Zn­(II), but the second was bound to the protein residues without forming a coordination bond with the Zn­(II). This second compound molecule did not bind solely due to crystal-forming effects because it did not bind to the second protein molecule. Therefore, the binding of the second molecule is likely not an artifact. However, the presence of the second molecule in the crystal structure does not mean that it is bound as strongly, nor that we can measure its binding affinity experimentally. Binding assays showed that the stoichiometry here was 1:1, and the affinity of the second molecule was likely weak compared to the first one. Compound concentration in the crystallization experiments was relatively high, millimolar, thus the second molecule could be seen in the crystal structure even if the *K*_d_ was in the millimolar range and therefore not interfering in any binding assays. This also means that the second molecule is biologically irrelevant and would not play an essential role in drug design.

## Conclusions

The strategy to acidify the p*K*_a_ of *ortho* sulfanyl-substituted benzenesulfonamides by oxidation, with the goal of increased affinity to CA, yielded an unexpected drop of affinity in the order of hundreds of thousands of times. Small changes in chemical structures influenced the flexibility of the molecule’s substituents and steric restrictions on interactions with proteins. Furthermore, some minor changes led to the discovery of novel CAIX inhibitors with high affinity and selectivity.

## Experimental Section

### Organic Synthesis

All starting materials and reagents were commercial products used without further purification. Melting points of the compounds were determined in open capillaries on a Thermo Scientific 9100 Series and are uncorrected. ^1^H and ^13^C NMR spectra (Figure S7) were recorded on a (400 and 100 MHz, respectively) spectrometer in DMSO-*d*_6_ using residual DMSO signals (2.52 and 40.21 ppm for ^1^H and ^13^C NMR spectra, respectively) as the internal standard. TLC was performed with silica gel 60 F254 aluminum plates (Merck) and visualized with UV light. Column chromatography used silica gel 60 (0.040–0.063 mm, Merck). High-resolution mass spectra (HRMS) were recorded by an Agilent TOF 6230 equipped with an Agilent Infinity 1260 HPLC system (Agilent Technologies). HPLC verified the purity of final compounds to be >95% (Figure S8) using the Agilent Infinity 1260 instrument with a ZORBAX Eclipse Plus C18 Rapid Resolution 4.6 × 100 mm 3.5 μm column, ZORBAX Eclipse Plus-C18 4.6 × 12.5 mm 5.0 μm analytical guard column, eluents A – 20 mM ammonium acetate (pH = 6.9 of unadjusted solution) and B – 100% MeOH was used. HPLC gradient elution with a flow rate of 0.80 mL/min was used, B gradient: 0–10 min 55–95%, 10–14 min 95%. UV detection was recorded at 254 nm. Figure S9 contains ESI-MS spectra of representative compounds.

#### Methyl 4-Chloro-3-sulfamoyl-benzoate (**2**)

4-chloro-3-sulfamoylbenzoic acid **1** (1.78 g, 7.55 mmol, Sigma-Aldrich) was refluxed in MeOH (30 mL) with concentrated H_2_SO_4_ (0.3 mL) for 8 h. The reaction mixture was concentrated under reduced pressure. Residue was filtered, washed with H_2_O and crystallized from H_2_O:MeOH­(4:1). Yield: 1.70 g, 91%, mp 129–130 °C (Literature^44^ mp 124–125 °C).

^**1**^ **H NMR** (400 MHz, DMSO-*d*_6_) δ ppm: 3.91 (s, 3H, C**H**_**3**_O), 7.80 (d, *J* = 8.4 Hz, 1H, Ar**H**), 7.84 (s, 2H, SO_2_N**H**_**2**_), 8.14 (dd, *J* = 8.4 Hz, *J* = 2.1 Hz, 1H, Ar**H**), 8.52 (d, *J* = 2.1 Hz, 1H, Ar**H**).

#### *N*-Butyl-4-chloro-3-sulfamoyl-benzamide (**3**)

The mixture of 4-chloro-3-sulfamoylbenzoic acid **1** (500 mg, 2.12 mmol), SOCl_2_ (0.616 mL, 8.48 mmol), and one drop DMF in toluene (6.0 mL) was refluxed for four h. Excess SOCl_2_ and toluene were removed by distillation under reduced pressure. The crude acid chloride was dissolved in THF (20 mL) and added dropwise to a solution of *N*-butylamine (0.591 mL, 6.0 mmol) in THF (20 mL) at 0 °C and allowed stirring for one h. The mixture was warmed to room temperature and stirred for another 4 h. THF was removed under reduced pressure. The crude product was crystallized from H_2_O. Yield: 492 mg, 80%, mp 172–173 °C (lit.^45^ mp 171–172 °C).

^**1**^ **H NMR** (400 MHz, DMSO-*d*_6_) δ ppm: 0.92 (t, *J* = 7.3 Hz, 3H, C**H**_**3**_CH_2_), 1.37 (sextet, *J* = 7.3 Hz, 2H, C**H**_**2**_CH_3_), 1.54 (quint, *J* = 7.3 Hz, 2H, CH_2_ **CH**_**2**_CH_2_), 3.29 (q, *J* = 6.8 Hz, 2H, C**H**_**2**_NH), 7.72 (s, 2H, SO_2_N**H**_**2**_), 7.77 (d, *J* = 8.2 Hz, 1H, Ar**H**), 8.04 (dd, *J* = 8.2 Hz, *J* = 2.0 Hz, 1H, Ar**H**), 8.45 (d, *J* = 2.0 Hz, 1H, Ar**H**), 8.77 (t, *J* = 5.5 Hz, 1H, N**H**).

#### General Procedure for the syntheses of **4a–d, f, g, i–m**

The mixture of methyl 4-chloro-3-sulfamoylbenzoate **2** (285 mg, 1,14 mmol), DMF (4.0 mL), appropriate thiol (1.25 mmol), and K_2_CO_3_ (630 mg, 4,56 mmol) was heated at 80 °C for 4–6 h in an inert atmosphere (argon). The mixture was cooled to room temperature, and 10 mL of H_2_O was added. The product was extracted with EtOAc (3 × 8 mL). The organic layer was washed with H_2_O, dried over anhydrous MgSO_4_, filtered, and concentrated under reduced pressure.

#### Methyl 4-Phenylsulfanyl-3-sulfamoyl-benzoate (**4a**)

The product was purified by flash chromatography on silica gel (CHCl_3_:EtOAc, 6:1). Yield: 303 mg, 82%, mp 155–156 °C. (lit.^46^ mp 154–157).

^**1**^ **H NMR** (400 MHz, DMSO-*d*_6_) δ ppm: 3.86 (s, 3H, C**H**_**3**_O), 7.00 (d, *J* = 8.4 Hz, 1H, Ar**H**), 7.55–7.62 (m, 5H, Ar**H**), 7.74 (s, 2H, SO_2_N**H**_**2**_), 7.93 (dd, *J* = 8.4 Hz, *J* = 1.9 Hz, 1H, Ar**H**), 8.46 (t, *J* = 1.9 Hz, 1H, Ar**H**).

^**13**^ **C NMR** (100 MHz, DMSO-*d*_6_) δ ppm: 52.9, 126.8, 129.0, 129.2, 130.5, 130.8, 131.0, 132.6, 135.5, 140.8, 144.2, 165.4.

HRMS calcd for C_14_H_13_NO_4_S_2_ [(M+H)^+^]: 324.0359, found: 324.0354.

#### Methyl 4-Cyclohexylsulfanyl-3-sulfamoyl-benzoate (**4b**)

The product was purified by flash chromatography on silica gel (CHCl_3_:EtOAc, 7:1). Yield: 315 mg, 84%, mp 119–120 °C.

^**1**^ **H NMR** (400 MHz, DMSO-*d*_6_) δ ppm: 1.26–1.99 (m, 10H, C**H** cyclohexyl), 3.61 (m, 1H, C**H**S), 3.88 (s, 3H, C**H**_**3**_O), 7.47 (s, 2H, SO_2_N**H**_**2**_), 7.74 (d, *J* = 8.4 Hz, 1H, Ar**H**), 8.04 (dd, *J* = 8.3 Hz, *J* = 1.9 Hz, 1H, Ar**H**), 8.43 (d, *J* = 1.9 Hz, 1H, Ar**H**).

^**13**^ **C NMR** (100 MHz, DMSO-*d*_6_) δ ppm: 25.7, 25.8, 32.5, 44.4, 52.9, 100.0, 125.9, 129.1, 132.3, 141.7, 142.8, 165.6.

HRMS calcd for C_14_H_19_NO_4_S_2_ [(M+H)^+^]: 330.0828, found: 330.0833.

#### Methyl 4-Benzylsulfanyl-3-sulfamoyl-benzoate (**4c**)

The product was purified by flash chromatography on silica gel (CHCl_3_:EtOAc, 6:1). Yield: 211 mg, 55%, mp 135–136 °C.

^**1**^ **H NMR** (400 MHz, DMSO-*d*_6_) δ ppm: 3.88 (s, 3H, C**H**_**3**_O), 4.43 (s, 2H, C**H**_**2**_S), 7.29–7.37 (m, 3H, Ar**H**), 7.51–7.53 (m, 2H, Ar**H**), 7.58 (s, 2H, SO_2_N**H**_**2**_), 7.74 (d, *J* = 8.3 Hz, 1H, Ar**H**), 8.02 (dt, *J* = 8.3 Hz, *J* = 1.7 Hz, 1H, Ar**H**), 8.42 (t, *J* = 2.2 Hz, 1H, Ar**H**).

^**13**^ **C NMR** (100 MHz, DMSO-*d*_6_) δ ppm: 36.4, 52.9, 125.9, 127.7, 128.0, 128.9, 129.0, 129.8, 132.2, 136.0, 140.7, 143.7, 165.6.

HRMS calcd for C_15_H_15_NO_4_S_2_ [(M+H)^+^]: 338.0515, found: 338.0517.

#### Methyl 4-(2-Phenylethylsulfanyl)-3-sulfamoyl-benzoate (**4d**)

The product was purified by flash chromatography on silica gel (CHCl_3_:EtOAc, 5:1). Yield: 332 mg, 83%, mp 146–147 °C.

^**1**^ **H NMR** (400 MHz, DMSO-*d*_6_) δ ppm: 2.99 (t, *J* = 7.5 Hz, 2H, C**H**_**2**_Ph), 3.41 (t, *J* = 7.5 Hz, 2H, C**H**_**2**_S), 3.88 (s, 3H, C**H**_**3**_O), 7.23–7.34 (m, 5H, Ar**H**), 7.51 (s, 2H, SO_2_N**H**_**2**_), 7.73 (d, *J* = 8.4 Hz, 1H, Ar**H**), 8.05 (dd, *J* = 8.4 Hz, *J* = 1.8 Hz, 1H, Ar**H**), 8.43 (d, *J* = 1.8 Hz, 1H, Ar**H**).

^**13**^ **C NMR** (100 MHz, DMSO-*d*_6_) δ ppm: 33.4, 34.3, 52.9, 125.8, 126.9, 127.8, 128.8, 128.9, 129.1, 132.4, 140.2, 141.,0, 143.6, 165.6.

HRMS calcd for C_16_H_17_NO_4_S_2_ [(M+H)^+^]: 352.0672, found: 352.0675.

#### Methyl 4-Cyclododecylsulfanyl-3-sulfamoyl-benzoate (**4f**)

The product was purified by flash chromatography on silica gel (CHCl_3_:EtOAc, 10:1). Yield: 203 mg, 43%, mp 167–168 °C.

^**1**^ **H NMR** (400 MHz, DMSO-*d*_6_) δ ppm: 1.32–1.59 (m, 20H, C**H** cyclododecyl), 1.76 (m, 2H, C**H** cyclododecyl), 3.66 (m, 1H, C**H**S), 3.88 (s, 3H, C**H**_**3**_O), 7.47 (s, 2H, SO_2_N**H**_**2**_), 7.70 (d, *J* = 8.4 Hz, 1H, Ar**H**), 8.07 (dd, *J* = 8.4 Hz, *J* = 2.0 Hz, 1H, Ar**H**), 8.44 (d, *J* = 2.0 Hz, 1H, Ar**H**).

^**13**^ **C NMR** (100 MHz, DMSO-*d*_6_) δ ppm: 22.0, 23.2, 23.3, 24.0, 24.2, 29.2, 43.5, 52.9, 125.9, 129.0, 129.2, 132.3, 142.0, 143.2, 165.6.

HRMS calcd for C_20_H_31_NO_4_S_2_ [(M+H)^+^]: 414.1767, found: 414.1773.

#### Methyl 4-(1-Adamantylsulfanyl)-3-sulfamoyl-benzoate (**4g**)

The product was purified by flash chromatography on silica gel (CHCl_3_:EtOAc, 10:1). Yield: 173 mg, 40%, mp 189–190 °C.

^**1**^ **H NMR** (400 MHz, DMSO-*d*_6_) δ ppm: 1.63 (br s, 6H, C**H** adamantanyl), 1.98 (br s, 6H, C**H** adamantanyl), 2.00 (br s, 3H, C**H** adamantanyl), 3.90 (s, 3H, OC**H**_**3**_), 7.40 (s, 2H, SO_2_N**H**_**2**_), 7.87 (d, *J* = 8.0 Hz, 1H, Ar**H**), 8.08 (dd, *J* = 8.0 Hz, *J* = 2.0 Hz, 1H, Ar**H**), 8.53 (d, *J* = 2.0 Hz, 1H, Ar**H**).

^**13**^ **C NMR** (100 MHz, DMSO-*d*_6_) δ ppm: 30.1, 35.9, 43.8, 52.2, 53.0, 128.8, 128.9, 131.8, 137.7, 137.9, 147.0, 165.5.

HRMS calcd for C_18_H_23_NO_4_S_2_ [(M+H)^+^]: 382.1141, found: 382.1144.

#### Methyl 4-Propylsulfanyl-3-sulfamoyl-benzoate (**4i**)

The product was purified by flash chromatography on silica gel (CHCl_3_:EtOAc, 5:1). Yield: 202 mg, 61%, mp 111–112 °C.

^**1**^ **H NMR** (400 MHz, DMSO-*d*_6_) δ ppm: 1.05 (t, *J* = 7.3 Hz, 3H, C**H**_**3**_CH_2_), 1.71 (quint, *J* = 7.3 Hz, 2H, CH_3_C**H**_**2**_), 3.11 (t, *J* = 7.3 Hz, 2H, C**H**_**2**_S), 3.88 (s, 3H, C**H**_**3**_O), 7.54 (s, 2H, SO_2_N**H**_**2**_), 7.65 (d, *J* = 8.3 Hz, 1H, Ar**H**), 8.04 (dd, *J* = 8.3 Hz, *J* = 1.9 Hz, 1H, Ar**H**), 8.43 (d, *J* = 1.9 Hz, 1H, Ar**H**).

^**13**^ **C NMR** (100 MHz, DMSO-*d*_6_) δ ppm: 13.8, 21.7, 33.9, 52.8, 125.6, 127.5, 128.9, 132.3, 140.9, 144.1, 165.6.

HRMS calcd for C_11_H_15_NO_4_S_2_ [(M+H)^+^]: 290.0515, found: 290.0519.

#### Methyl 4-*tert*-Butylsulfanyl-3-sulfamoyl-benzoate (**4j**)

The product was purified by flash chromatography on silica gel (CHCl_3_:EtOAc, 5:1). Yield: 214 mg, 62%, mp 124–125 °C.

^**1**^ **H NMR** (400 MHz, DMSO-*d*_6_) δ ppm: 1.43 (s, 9H, C**H**_**3**_), 3.90 (s, 3H, C**H**_**3**_O), 7.43 (s, 2H, SO_2_N**H**_**2**_), 7.91 (d, *J* = 8.2 Hz, 1H, Ar**H**), 8.11 (dd, *J* = 8.2 Hz, *J* = 2.0 Hz, 1H, Ar**H**), 8.52 (d, *J* = 2.0 Hz, 1H, Ar**H**).

^**13**^ **C NMR** (100 MHz, DMSO-*d*_6_) δ ppm: 31.6, 49.6, 53.0, 128.4, 128.9, 132.0, 136.2, 140.0, 146.1, 165.5.

HRMS calcd for C_12_H_17_NO_4_S_2_ [(M+H)^+^]: 304.0672, found: 304.0671.

#### Methyl 4-Isopentylsulfanyl-3-sulfamoyl-benzoate (**4k**)

The product was purified by flash chromatography on silica gel (CHCl_3_:EtOAc, 5:1). Yield: 265 mg, 74%, mp 92–93 °C.

^**1**^ **H NMR** (400 MHz, DMSO-*d*_6_) δ ppm: 0.94 (d, *J* = 6.6 Hz, 6H, C**H**_**3**_), 1.57 (td, *J* = 7.8 Hz, *J* = 6.8 Hz, 2H, C**H**_**2**_CH), 1.76 (m, 1H, C**H**), 3.11 (t, *J* = 7.8 Hz, 2H, C**H**_**2**_S), 3.88 (s, 3H, C**H**_**3**_O), 7.52 (s, 2H, SO_2_N**H**_**2**_), 7.67 (d, *J* = 8.4 Hz, Ar**H**), 8.05 (dd, *J* = 8.4 Hz, *J* = 2.0 Hz, 1H, Ar**H**), 8.42 (d, *J* = 2.0 Hz, 1H, Ar**H**).

^**13**^ **C NMR** (100 MHz, DMSO-*d*_6_) δ ppm: 22.6, 27.5, 30.2, 36.9, 52.8, 125.6, 127.5, 128.9, 132.3, 140.9, 144.1, 165.6.

HRMS calcd for C_13_H_19_NO_4_S_2_ [(M+H)^+^]: 318.0828, found: 318.0833.

#### Methyl 4-Cyclopentylsulfanyl-3-sulfamoyl-benzoate (**4l**)

The product was purified by flash chromatography on silica gel (CHCl_3_:EtOAc, 8:1). Yield: 259 mg, 72%, mp 106–107 °C.

^**1**^ **H NMR** (400 MHz, DMSO-*d*_6_) δ ppm: 1.55–1.65 (m, 4H, C**H** cyclopentyl), 1.73–1.78 (m, 2H, C**H** cyclopentyl), 2.18–2.23 (m, 2H, C**H** cyclopentyl), 3.84–3.92 (m, 4H, C**H**_**3**_O and C**H**S), 7.50 (s, 2H, SO_2_N**H**_**2**_), 7.72 (d, *J* = 8.4 Hz, 1H, Ar**H**), 8,.04 (dd, *J* = 8.4 Hz, *J* = 2.0 Hz, 1H, Ar**H**), 8.42 (d, *J* = 2.0 Hz, 1H, Ar**H**).

^**13**^ **C NMR** (100 MHz, DMSO-*d*_6_) δ ppm: 25.1, 33.3, 43.9, 52.8, 125.5, 128.4, 128.9, 132.3, 140.8, 144.7, 165.6.

HRMS calcd for C_13_H_17_NO_4_S_2_ [(M+H)^+^]: 316.0672, found: 316.0677.

#### Methyl 4-(1-Naphthylsulfanyl)-3-sulfamoyl-benzoate (**4m**)

The product was purified by flash chromatography on silica gel (CHCl_3_:EtOAc, 8:1). Yield: 281 mg, 66%, mp 187–188 °C.

^**1**^ **H NMR** (400 MHz, DMSO-*d*_6_) δ ppm: 3.83 (s, 3H, C**H**_**3**_O), 6.64 (d, *J* = 8.4 Hz, 1H, Ar**H**), 7.54–7.64 (m, 2H, Ar**H**), 7.69 (m, 1H, Ar**H**), 7.74 (dd, *J* = 8.4 Hz, *J* = 2.0 Hz, 1H, Ar**H**), 7.93 (s, 2H, SO_2_N**H**_**2**_), 8.04–8.15 (m, 3H, Ar**H**), 8.22 (d, *J* = 8.3 Hz, 1H, Ar**H**), 8.49 (d, *J* = 2.,0 Hz, 1H, Ar**H**).

^**13**^ **C NMR** (100 MHz, DMSO-*d*_6_) δ ppm: 52.9, 125.6, 126.7, 126.9, 127.3, 127.5, 128.4, 128.5, 129.1, 129.6, 132.2, 132.5, 133.8, 134.8, 136.8, 140.4, 143.9, 165.4.

HRMS calcd for C_18_H_15_NO_4_S_2_ [(M+H)^+^]: 374.0515, found: 374.0511.

#### Methyl 4-Methylsulfanyl-3-sulfamoyl-benzoate (**4h**)

The mixture of methyl 4-chloro-3-sulfamoylbenzoate **2** (308 mg, 1,23 mmol), DMSO (4.0 mL), sodium methanethiolate (259 mg, 3.69 mmol), and K_2_CO_3_ (509 mg, 3.69 mmol) was heated at 80 °C temperature for 12 h in inert atmosphere­(argon). The mixture was cooled to room temperature and 20 mL brine was added. The product was extracted with EtOAc (3 × 10 mL). The organic layer was washed with H_2_O, dried over anhydrous MgSO_4_, filtered, and concentrated under reduced pressure. The product was crystallized from H_2_O:MeOH­(4:1). Yield: 203 mg, 64%, mp 157–158 °C.

^**1**^ **H NMR** (400 MHz, DMSO-*d*_6_) δ ppm: 2.59 (s, 3H, C**H**_**3**_S), 3.89 (s, 3H, C**H**_**3**_O), 7.54 (s, 2H, SO_2_N**H**_**2**_), 7.61 (d, *J* = 8.3 Hz, 1H, Ar**H**), 8.07 (dd, *J* = 8.3 Hz, *J* = 1.4 Hz, 1H, Ar**H**), 8.42 (d, *J* = 1.4 Hz, 1H, Ar**H**).

^**13**^ **C NMR** (100 MHz, DMSO-*d*_6_) δ ppm: 15.5, 52.9, 125.4, 126.6, 128.8, 132.4, 140.4, 145.1, 165.7.

HRMS calcd for C_9_H_11_NO_4_S_2_ [(M+H)^+^]: 262.0202, found: 262.0199.

#### General Procedure for the Syntheses of **5a–d, f–g, j–m**

The mixture of *N*-butyl-4-chloro-3-sulfamoyl-benzamide **3** (111 mg, 0.381 mmol), DMF (4.0 mL), appropriate thiol (0.572 mmol), and K_2_CO_3_ (210 mg, 1.52 mmol) was heated at 80 °C temperature for 12 h in an inert atmosphere­(argon). The mixture was cooled to room temperature and 20 mL H_2_O was added. The product was extracted with EtOAc (3 × 8 mL). The organic layer was washed with H_2_O, dried over anhydrous MgSO_4_, filtered, and concentrated under reduced pressure.

#### *N*-Butyl-4-phenylsulfanyl-3-sulfamoyl-benzamide (**5a**)

The product was purified by flash chromatography on silica gel (CHCl_3_:EtOAc, 1:1). Yield: 71 mg, 51%, mp 109–110 °C.

^**1**^ **H NMR** (400 MHz, DMSO-*d*_6_) δ ppm: 0.90 (t, *J* = 7.3 Hz, 3H, C**H**_**3**_), 1.33 (sext, *J* = 7.3 Hz, 2H, CH_2_C**H**_**2**_CH_3_), 1.52 (quint, *J* = 7.3 Hz, 2H, CH_2_C**H**_**2**_CH_2_), 3.27 (dt, *J* = 6.9 Hz, *J* = 5.7 Hz, 2H, C**H**_**2**_NH), 6.99 (d, *J* = 8.3 Hz, 1H, Ar**H**), 7.51–7.58 (m, 5H, Ar**H**), 7.60 (s, 2H, SO_2_N**H**_**2**_), 7.82 (dd, *J* = 8.3 Hz, *J* = 2.0 Hz, 1H, Ar**H**), 8.38 (d, *J* = 2.0 Hz, 1H, Ar**H**), 8.59 (t, *J* = 5.7 Hz, 1H, N**H**).

^**13**^ **C NMR** (100 MHz, DMSO-*d*_6_) δ ppm: 14.2, 20.1, 31.6, 39.4, 127.5, 129.6, 129.9, 130.5, 130.6, 132.3, 132.4, 134.9, 140.7, 141.2, 164.9.

HRMS calcd for C_17_H_20_N_2_O_3_S_2_ [(M+H)^+^]: 365.0988, found: 365.0985.

#### *N*-Butyl-4-cyclohexylsulfanyl-3-sulfamoyl-benzamide (**5b**)

The product was purified by flash chromatography on silica gel (CHCl_3_:EtOAc, 1:1). Yield: 70 mg, 50%, mp 114–115 °C.

^**1**^ **H NMR** (400 MHz, DMSO-*d*_6_) δ ppm: 0.92 (t, *J* = 7.3 Hz, 3H, C**H**_**3**_), 1.23–1.63 (m, 10H, C**H** cyclohexyl and butyl), 1.72 (m, 2H, C**H** cyclohexyl), 1.90–1.96 (m, 2H, C**H** cyclohexyl), 3.29 (q, *J* = 6.6 Hz, 2H, C**H**_**2**_NH), 3.55–3.60 (m, 1H, C**H**S), 7.34 (s, 2H, SO_2_N**H**_**2**_), 7.70 (d, *J* = 8.2 Hz, 1H, Ar**H**), 7.96 (dd, *J* = 8.2 Hz, *J* = 1.2 Hz, 1H, Ar**H**), 8.36 (d, *J* = 1.2 Hz, 1H, Ar**H**), 8.64 (t, *J* = 5.3 Hz, 1H, N**H**).

^**13**^ **C NMR** (100 MHz, DMSO-*d*_6_) δ ppm: 14.2, 20.1, 25.7, 25.8, 31.6, 32.5, 39.4, 44.8, 127.6, 129.7, 130.3, 131.6, 139.0, 142.1, 165.1

HRMS calcd for C_17_H_26_N_2_O_3_S_2_ [(M+H)^+^]: 371.1458, found: 371.1463.

#### 4-Benzylsulfanyl-*N*-butyl-3-sulfamoyl-benzamide (**5c**)

The product was purified by flash chromatography on silica gel (CHCl_3_:EtOAc, 1:1). Yield: 65 mg, 45%, mp 185–186 °C.

^**1**^ **H NMR** (400 MHz, DMSO-*d*_6_) δ ppm: 0.91 (t, *J* = 7.2 Hz, 3H, C**H**_**3**_), 1.36 (sext, *J* = 7.2 Hz, 2H, C**H**_**2**_CH_3_), 1.53 (quint, *J* = 7.2 Hz, 2H, CH_2_ **CH**_**2**_CH_2_), 3.28 (q, *J* = 6.9 Hz, 2H, C**H**_**2**_NH), 4.40 (s, 2H, C**H**_**2**_S), 7.29 (t, *J* = 7.1 Hz, 1H, Ar**H**), 7.36 (t, *J* = 7.1 Hz, 2H, Ar**H**), 7.44 (s, 2H, SO_2_N**H**_**2**_), 7.52 (d, *J* = 7.1 Hz, 2H, Ar**H**), 7.66 (d, *J* = 8.3 Hz, 1H, Ar**H**), 7.94 (dd, *J* = 8.3 Hz, *J* = 1.9 Hz, 1H, Ar**H**), 8.36 (d, *J* = 1.9 Hz, 1H, Ar**H**), 8.62 (t, *J* = 5.6 Hz, 1H, N**H**).

^**13**^ **C NMR** (100 MHz, DMSO-*d*_6_) δ ppm: 14.2, 20.1, 31.6, 36.5, 39.4, 127.6, 127.7, 127.8, 128.9, 129.7, 130.2, 131.2, 136.5, 140.2, 140.7, 165.0.

HRMS calcd for C_18_H_22_N_2_O_3_S_2_ [(M+H)^+^]: 379.1145, found: 379.1140.

#### *N*-Butyl-4-(2-phenylethylsulfanyl)-3-sulfamoyl-benzamide (**5d**)

The product was purified by flash chromatography on silica gel (CHCl_3_:EtOAc, 1:1). Yield: 95 mg, 63%, mp 116–117 °C.

^**1**^ **H NMR** (400 MHz, DMSO-*d*_6_) δ ppm: 0.92 (t, *J* = 7.3 Hz, 3H, C**H**_**3**_), 1.37 (sext, *J* = 7.3 Hz, 2H, C**H**_**2**_CH_3_), 1.55 (quint, *J* = 7.3 Hz, 2H, CH_2_C**H**_**2**_CH_2_), 2.98 (t, *J* = 7.3 Hz, 2H, C**H**_**2**_Ar), 3.28 (q, *J* = 6.6 Hz, 2H, C**H**_**2**_NH), 3.34–3.38 (m, 2H, C**H**_**2**_S), 7.25–7.27 (m, 1H, Ar**H**), 7.31–7.33 (m, 4H, Ar**H**), 7.35 (s, 2H, SO_2_N**H**_**2**_), 7.69 (d, *J* = 8.3 Hz, 1H, Ar**H**), 7.99 (d, *J* = 8.3 Hz, 1H, Ar**H**), 8.38 (s, 1H, Ar**H**), 8.65 (t, *J* = 5.4 Hz, 1H, N**H**).

^**13**^ **C NMR** (100 MHz, DMSO-*d*_6_) δ ppm: 14.2, 20.1, 31.6, 33.7, 34.4, 39.4, 126.9, 127.5, 127.9, 128.9, 129.0, 130.4, 131.3, 140.1, 140.3, 141.2, 165.1.

HRMS calcd for C_19_H_24_N_2_O_3_S_2_ [(M+H)^+^]: 393.1301, found: 393.1297.

#### *N*-Butyl-4-cyclododecylsulfanyl-3-sulfamoyl-benzamide (**5f**)

The product was purified by flash chromatography on silica gel (CHCl_3_:EtOAc, 3:1). Yield: 76 mg, 44%, mp 166–168 °C.

^**1**^ **H NMR** (400 MHz, DMSO-*d*_6_) δ ppm: 0.93 (t, *J* = 7.3 Hz, 3H, C**H**_**3**_), 1.24–1.59 (m, 24H, C**H**_**2**_C**H**_**2**_CH_3_ and C**H** cyclododecyl), 1.72–1.75 (m, 2H, C**H** cyclododecyl), 3.29 (dt, *J* = 6.8 Hz, *J* = 5.6 Hz, 2H, C**H**_**2**_NH), 3.68 (s, 1H, C**H**S), 7.33 (s, 2H, SO_2_N**H**_**2**_), 7.66 (d, *J* = 8.3 Hz, 1H, Ar**H**), 7.99 (dd, *J* = 8.3 Hz, *J* = 2.0 Hz, 1H, Ar**H**), 8.37 (d, *J* = 2.0 Hz, 1H, Ar**H**), 8.64 (t, *J* = 5.6 Hz, 1H, N**H**).

^**13**^ **C NMR** (100 MHz, DMSO-*d*_6_) δ ppm: 14.2, 20.1, 22.1, 23.4, 23.8, 23.9, 24.2, 29.4, 31.6, 39.4, 43.7, 127.6, 129.5, 130.3, 131.6, 139.7, 142.3, 165.1.

HRMS calcd for C_23_H_38_N_2_O_3_S_2_ [(M+H)^+^]: 455.2397, found: 455.2399.

#### 4-(1-Adamantylsulfanyl)-*N*-butyl-3-sulfamoyl-benzamide (**5g**)

The product was purified by flash chromatography on silica gel (CHCl_3_:EtOAc, 1:1). Yield: 64 mg, 40%, mp 187–189 °C.

^**1**^ **H NMR** (400 MHz, DMSO-*d*_6_) δ ppm: 0.91 (t, *J* = 6.8 Hz, 3H, C**H**_**3**_), 1.34 (sext, *J* = 7.2 Hz, 2H, C**H**_**2**_CH_3_), 1.52 (quint, *J* = 7.2 Hz, 2H, NHCH_2_C**H**_**2**_), 1.63 (br s, 6H, C**H** adamantanyl), 1.95 (br s, 6H, C**H** adamantanyl), 2.01 (br s, 3H, C**H** adamantanyl), 3.28 (q, *J* = 6.8 Hz, 2H, NHC**H**_**2**_), 7.28 (s, 2H, SO_2_N**H**_**2**_), 7.78 (d, *J* = 8.0 Hz, 1H, Ar**H**), 7.98 (dd, *J* = 8.0 Hz, *J* = 2.0 Hz, 1H, Ar**H**), 8.44 (d, *J* = 2.0 Hz, 1H, Ar**H**), 8.71 (t, *J* = 5.6 Hz, 1H, N**H**).

^**13**^ **C NMR** (100 MHz, DMSO-*d*_6_) δ ppm: 14.2, 20.1, 30.1, 31.6, 36.0, 39.5, 43.9, 51.8, 127.4, 129.8, 134.0, 134.5, 138.2, 147.3, 165.2.

HRMS calcd for C_21_H_30_N_2_O_3_S_2_ [(M+H)^+^]: 423.1771, found: 423.1777.

#### *N*-Butyl-4-*tert*-butylsulfanyl-3-sulfamoyl-benzamide (**5j**)

The product was purified by flash chromatography on silica gel (CHCl_3_:EtOAc, 1:1). Yield: 55 mg, 42%, mp 150–152 °C.

^**1**^ **H NMR** (400 MHz, DMSO-*d*_6_) δ ppm: 0.91 (t, *J* = 7.6 Hz, 3H, C**H**_**3**_), 1.30–1.37 (m, 2H, CH_2_C**H**_**2**_CH_3_), 1.41 (s, 9H, C­(C**H**_**3**_)_3_), 1.52 (quint, *J* = 7.2 Hz, 2H, NHCH_2_C**H**_**2**_), 3.28 (q, *J* = 6.0 Hz, 2H, NHC**H**_**2**_), 7.31 (s, 2H, SO_2_N**H**_**2**_), 7.82 (d, *J* = 7.6 Hz, 1H, Ar**H**), 7.99 (dd, *J* = 8.0 Hz, *J* = 1.6 Hz, 1H, Ar**H**), 8.44 (d, *J* = 2.0 Hz, 1H, Ar**H**), 8.71 (t, *J* = 5.8 Hz, 1H, N**H**).

^**13**^ **C NMR** (100 MHz, DMSO-*d*_6_) δ ppm: 14.2, 20.1, 31.6, 31.7, 39.5, 49.4, 127.5, 130.1, 134.3, 136.2, 137.0, 146.7, 165.2.

HRMS calcd for C_15_H_24_N_2_O_3_S_2_ [(M+H)^+^]: 345.1301, found: 345.1295.

#### *N*-Butyl-4-isopentylsulfanyl-3-sulfamoyl-benzamide (**5k**)

The product was purified by flash chromatography on silica gel (CHCl_3_:EtOAc, 1:1). Yield: 98 mg, 72%, mp 130–132 °C.

^**1**^ **H NMR** (400 MHz, DMSO-*d*_6_) δ ppm: 0.89–0.94 (m, 9H, C**H**_**3**_), 1.33 (sext, *J* = 7.2 Hz, 2H, CH_2_C**H**_**2**_CH_3_), 1.48–1.57 (m, 4H, NHCH_2_C**H**_**2**_ and SCH_2_C**H**_**2**_), 1.75 (hept, *J* = 7.6 Hz, 1H, C**H**(CH_3_)_2_), 3.09 (t, *J* = 7.8 Hz, 2H, SC**H**_**2**_), 3.30 (q, *J* = 6.4 Hz, 2H, NHC**H**_**2**_), 7.38 (s, 2H, SO_2_N**H**_**2**_), 7.61 (d, *J* = 8.4 Hz, 1H, Ar**H**), 7.97 (dd, *J* = 8.3 Hz, *J* = 1.6 Hz, 1H, Ar**H**), 8.36 (d, *J* = 1.7 Hz, 1H, Ar**H**), 8.62 (t, *J* = 5.3 Hz, 1H, N**H**).

^**13**^ **C NMR** (100 MHz, DMSO-*d*_6_) δ ppm: 14.2, 20.1, 22.6, 27.5, 30.4, 31.7, 37.2, 39.4, 127.6­(2C), 130.4, 131.1, 140.6, 141.0, 165.1.

HRMS calcd for C_16_H_26_N_2_O_3_S_2_ [(M+H)^+^]: 359.1458, found: 359.1464.

#### *N*-Butyl-4-cyclopentylsulfanyl-3-sulfamoyl-benzamide (**5l**)

The product was purified by flash chromatography on silica gel (CHCl_3_:EtOAc, 1:1). Yield: 84 mg, 62%, mp 122–124 °C.

^**1**^ **H NMR** (400 MHz, DMSO-*d*_6_) δ ppm: 0.91 (t, *J* = 7.2 Hz, 3H, C**H**_**3**_), 1.33 (sext, *J* = 7.2 Hz, 2H, CH_2_C**H**_**2**_CH_3_), 1.48–1.67 (m, 6H, NHCH_2_C**H**_**2**_ and C**H** cyclopentyl), 1.71–1.81 (m, 2H, C**H** cyclopentyl), 2.12–2.21 (m, 2H, C**H** cyclopentyl), 3.28 (q, *J* = 6.4 Hz, 2H, NHC**H**_**2**_), 3.92 (quint, *J* = 6.0 Hz, 1H, SC**H**), 7.36 (s, 2H, SO_2_N**H**_**2**_), 7.66 (d, *J* = 8.4 Hz, 1H, Ar**H**), 7.96 (d, *J* = 7.6 Hz, 1H, Ar**H**), 8.36 (s, 1H, Ar**H**), 8.61 (t, *J* = 5.2 Hz, 1H, N**H**).

^**13**^ **C NMR** (100 MHz, DMSO-*d*_6_) δ ppm: 14.2, 20.1, 25.1, 31.7, 33.3, 39.4, 44.2, 127.6, 128.5, 130.3, 131.0, 141.0, 141.2, 165.1.

HRMS calcd for C_16_H_24_N_2_O_3_S_2_ [(M+H)^+^]: 357.1301, found: 357.1307.

#### *N*-Butyl-4-(1-naphthylsulfanyl)-3-sulfamoyl-benzamide (**5m**)

The product was purified by flash chromatography on silica gel (CHCl_3_:EtOAc, 1:1). Yield: 96 mg, 61%, mp 216–218 °C.

^**1**^ **H NMR** (400 MHz, DMSO-*d*_6_) δ ppm: (DMSO–D_6_): 0.86 (t, *J* = 7.6 Hz, 3H, C**H**_**3**_), 1.28 (sext, *J* = 7.6 Hz, 2H, CH_2_C**H**_**2**_CH_3_), 1.45 (quint, *J* = 7.2 Hz, 2H, NHCH_2_C**H**_**2**_), 3.21 (q, *J* = 6.8 Hz, 2H, NHC**H**_**2**_), 6.57 (d, *J* = 8.4 Hz, 1H, Ar**H**), 7.53–7.63 (m, 3H, Ar**H** and, naphthyl-**H**), 7.68 (t, *J* = 8.0 Hz, 1H, naphthyl-**H**), 7.83 (s, 2H, SO_2_N**H**_**2**_), 8.03 (d, *J* = 6.8 Hz, 1H, naphthyl-**H**), 8.08 (d, *J* = 8.0 Hz, 1H, naphthyl-**H**), 8.15 (d, *J* = 8.4 Hz, 1H, naphthyl-**H**), 8.19 (d, *J* = 8.4 Hz, 1H, naphthyl-**H**), 8.41 (d, *J* = 2.0 Hz, 1H, Ar**H**), 8.50 (t, *J* = 5.6 Hz, 1H, N**H**).

^**13**^ **C NMR** (100 MHz, DMSO-*d*_6_) δ ppm: 14.1, 20.0, 31.6, 39.4, 125.8, 126.9, 127.4, 127.7, 128.1, 128.2­(2C), 129.5, 130.4, 131.8, 132.1, 133.7, 134.8, 136.5, 140.4, 140.7, 165.1.

HRMS calcd for C_21_H_22_N_2_O_3_S_2_ [(M+H)^+^]: 415.1145, found: 415.1151.

#### General Procedure for the Syntheses of **6b, c, e**

The mixture of *N*-butyl-4-chloro-3-sulfamoyl-benzamide **3** (100 mg, 0.344 mmol) and appropriate amine (2.0 mL) was heated at 130 °C for 24 h in an inert atmosphere. The mixture was cooled to room temperature and 2 N HCl­(aq) (5 mL) was added. The product was extracted with EtOAc (3 × 5 mL). The organic layer was washed with H_2_O, dried over anhydrous MgSO_4_, filtered, and concentrated under reduced pressure.

#### *N*-Butyl-4-(cyclohexylamino)-3-sulfamoyl-benzamide (**6b**)

The product was purified by flash chromatography on silica gel (CHCl_3_:EtOAc, 1:1). Yield: 64 mg, 53%, mp 89–91 °C.

^**1**^ **H NMR** (400 MHz, DMSO-*d*_6_) δ ppm: 0.91 (t, *J* = 7.3 Hz, 3H, C**H**_**3**_), 1.23–1.60 (m, 10H, C**H**_**2**_C**H**_**2**_CH_3_ and C**H** cyclohexyl), 1.66–1.69 (m, 2H, C**H** cyclohexyl), 1.90–1.93 (m, 2H, C**H** cyclohexyl), 3.25 (dt, *J* = 6.8 Hz, *J* = 5.7 Hz, 2H, C**H**_**2**_NH), 3.49–3.52 (m, 1H, C**H**NH), 6,17 (d, *J* = 7.6 Hz, 1H, N**H**-cyclohexyl), 6.84 (d, *J* = 8.8 Hz, 1H, Ar**H**), 7.43 (s, 2H, SO_2_N**H**_**2**_), 7.85 (dd, *J* = 8.8 Hz, *J* = 2.1 Hz, 1H, Ar**H**), 8.21 (d, *J* = 2.1 Hz, 1H, Ar**H**), 8.25 (t, *J* = 5.7 Hz, 1H, N**H**).

^**13**^ **C NMR** (100 MHz, DMSO-*d*_6_) δ ppm: 14.2, 20.1, 24.4, 25.8, 31.9, 32.3, 39.2, 50.5, 111.8, 120.9, 124.5, 129.2, 132.3, 146.3, 165.4.

HRMS calcd for C_17_H_27_N_3_O_3_S [(M+H)^+^]: 354.1846, found: 354.1840.

#### 4-(Benzylamino)-*N*-butyl-3-sulfamoyl-benzamide (**6c**)

The product was purified by flash chromatography on silica gel (CHCl_3_:EtOAc, 1:1). Yield: 40 mg, 32%, mp 132–133 °C.

^**1**^ **H NMR** (400 MHz, DMSO-*d*_6_) δ ppm: 0.88 (t, *J* = 7.2 Hz, 3H, C**H**_**3**_), 1.35 (sext, *J* = 7.2 Hz, 2H, C**H**_**2**_CH_3_), 1.49 (quint, *J* = 7.2 Hz, 2H, CH_2_C**H**_**2**_CH_2_), 3.23 (dt, *J* = 6.8 Hz, *J* = 5.9 Hz, 2H, C**H**_**2**_NH), 4.55 (d, *J* = 5.8 Hz, 2H, PhC**H**_**2**_NH), 6.69 (d, *J* = 8.8 Hz, 1H, Ar**H**), 6.82 (t, *J* = 5.8 Hz, 1H, N**H**-benzyl), 7.23–7.39 (m, 5H, Ar**H**), 7.49 (s, 2H, SO_2_N**H**_**2**_), 7.77 (dd, *J* = 8.8 Hz, *J* = 2.0 Hz, 1H, Ar**H**), 8.23 (m, 2H, Ar**H** and N**H**).

^**13**^ **C NMR** (100 MHz, DMSO-*d*_6_) δ ppm: 14.2, 20.1, 31.8, 39.2, 46.5, 111.9, 121.5, 125.1, 127.4, 128.9, 128.9, 132.1, 139.2, 146.8, 165.4.

HRMS calcd for C_18_H_23_N_3_O_3_S [(M+H)^+^]: 362.1533, found: 362.1539.

#### *N*-Butyl-4-(cyclooctylamino)-3-sulfamoyl-benzamide (**6e**)

The product was purified by flash chromatography on silica gel (CHCl_3_:EtOAc, 1:1). Yield: 55 mg, 42%, brownish oily residue.

^**1**^ **H NMR** (400 MHz, DMSO-*d*_6_) δ ppm: 0.91 (t, *J* = 7.3 Hz, 3H, C**H**_**3**_), 1.35 (sext, *J* = 7.3 Hz, 2H, C**H**_**2**_CH_3_), 1.44–1.73 (m, 14H, C**H** cyclooctyl and CH_2_C**H**_**2**_CH_2_), 1.81–1.86 (m, 2H, C**H** cyclooctyl), 3.25 (dt, *J* = 6.9 Hz, *J* = 5.9 Hz, 2H, C**H**_**2**_NH), 3.70–3.73 (m, 1H, C**H**NH cyclooctyl), 6.19 (d, *J* = 7.6 Hz, 1H, N**H-**cyclooctyl), 6.75 (d, *J* = 8.8 Hz, 1H, Ar**H**), 7.43 (s, 2H, SO_2_N**H**_**2**_), 7,.86 (dd, *J* = 8.8 Hz, *J* = 2.1 Hz, 1H, Ar**H**), 8.19 (d, *J* = 2.1 Hz, 1H, Ar**H**), 8.24 (t, *J* = 5.9 Hz, 1H, N**H**).

^**13**^ **C NMR** (100 MHz, DMSO-*d*_6_) δ ppm: 14.2, 20.1, 23.6, 25.5, 27.2, 31.7, 31.9, 39.2, 51.9, 111.9, 120.8, 124.6, 129.3, 132.4, 146.1, 165.5.

HRMS calcd for C_19_H_31_N_3_O_3_S [(M+H)^+^]: 382.2159, found: 382.2155.

#### General Procedure for the Syntheses of **7­(a, b, h, i, k–m), 8­(a, b, k–m)**

The mixture of appropriate methyl 4-sulfanilsubstituted-3-sulfamoyl-benzoate (**4a, b, h, i, k–m**) or appropriate *N*-butyl-4-sulfanilsubstituted-3-sulfamoyl-benzamide (**8a, b, k–m**) (0.200 mmol), acetic acid (2.0 mL) and 30% H_2_O_2_ (0.090 mL, 1.15 mmol) was stirred for 12 h. Then 1% Na_2_SO_3_(aq) (6.0 mL) was added to the mixture and the product was extracted with EtOAc (3 × 5 mL). The organic layer was washed with H_2_O, dried over anhydrous MgSO_4_, filtered, and concentrated under reduced pressure.

#### Methyl 4-(Benzenesulfinyl)-3-sulfamoyl-benzoate (**7a**)

The product was purified by flash chromatography on silica gel (CHCl_3_:EtOAc, 3:1). Yield: 56 mg, 82%, mp 107–108 °C.

^**1**^ **H NMR** (400 MHz, DMSO-*d*_6_) δ ppm: 3,91 (s, 3H, C**H**_**3**_O), 7.48–7.53 (m, 3H, Ar**H**), 7.75–7.78 (m, 2H, Ar**H**), 8.09 (s, 2H, SO_2_N**H**_**2**_), 8.32 (d, *J* = 8.2 Hz, 1H, Ar**H**), 8.37 (dd, *J* = 8.2 Hz, *J* = 1.7 Hz, 1H, Ar**H**), 8.47 (d, *J* = 1.7 Hz, 1H, Ar**H**).

^**13**^ **C NMR** (100 MHz, DMSO-*d*_6_) δ ppm: 53.3, 125.6, 126.9, 128.5, 129.9, 131.6, 132.8, 133.9, 142.3, 145.9, 149.7, 164.9.

HRMS calcd for C_14_H_13_NO_5_S_2_ [(M+H)^+^]: 340.0308, found: 340.0311.

#### Methyl 4-Cyclohexylsulfinyl-3-sulfamoyl-benzoate (**7b**)

The product was purified by flash chromatography on silica gel (CHCl_3_:EtOAc, 3:1). Yield: 52 mg, 75%, mp 188–189 °C.

^**1**^ **H NMR** (400 MHz, DMSO-*d*_6_) δ ppm: 1.14–2.07 (m, 10H, C**H**_**2**_ cyclohexyl), 2.96–3.04 (m, 1H, C**H**SO), 3.94 (s, 3H, C**H**_**3**_O), 7.97 (s, 2H, SO_2_N**H**_**2**_), 8.11 (d, *J* = 8.2 Hz, 1H, Ar**H**), 8.38 (dd, *J* = 8.2 Hz, *J* = 1.7 Hz, 1H, Ar**H**), 8.51 (d, *J* = 1.7 Hz, 1H, Ar**H**).

^**13**^ **C NMR** (100 MHz, DMSO-*d*_6_) δ ppm: 20.9, 25.1, 25.4, 26.1, 28.6, 53.3, 60.9, 127.3, 129.1, 132.5, 132.6, 142.1, 146.5, 165.1.

HRMS calcd for C_14_H_19_NO_5_S_2_ [(M+H)^+^]: 346.0777, found: 346.0783.

#### Methyl 4-Methylsulfinyl-3-sulfamoyl-benzoate (**7h**)

The product was purified by flash chromatography on silica gel (CHCl_3_:EtOAc, 5:1). Yield: 50 mg, 90%, mp 258–259 °C.

^**1**^ **H NMR** (400 MHz, DMSO-*d*_6_) δ ppm: 2.81 (s, 3H, C**H**_**3**_SO), 3.95 (s, 3H, C**H**_**3**_O), 7.97 (s, 2H, SO_2_N**H**_**2**_), 8.34 (d, *J* = 8.2 Hz, 1H, Ar**H**), 8.43 (dd, *J* = 8.2 Hz, *J* = 1.6 Hz, 1H, Ar**H**), 8.47 (d, *J* = 1.6 Hz, 1H, Ar**H**).

^**13**^ **C NMR** (100 MHz, DMSO-*d*_6_) δ ppm: 44.6, 53.3, 125.7, 128.5, 132.6, 133.8, 141.5, 151.2, 165.1.

HRMS calcd for C_9_H_11_NO_5_S_2_ [(M+H)^+^]: 278.0151, found: 278.0155.

#### Methyl 4-Propylsulfinyl-3-sulfamoyl-benzoate (**7i**)

The product was purified by flash chromatography on silica gel (CHCl_3_:EtOAc, 3:1). Yield: 40 mg, 66%, mp 144–145 °C.

^**1**^ **H NMR** (400 MHz, DMSO-*d*_6_) δ ppm: 0.99 (t, *J* = 7.4 Hz, 3H, C**H**_**3**_CH_2_), 1.59–1.62 (m, 1H, C**H**_**A**_H_B_CH_3_); 1,77–1.81 (m, 1H, CH_A_ **H**_**B**_CH_3_), 2.66–2.69 (m, 1H, C**H**_**A**_H_B_SO), 3.10–3.13 (m, 1H, CH_A_ **H**_**B**_SO), 3.93 (s, 3H, C**H**_**3**_O), 7.97 (s, 2H, SO_2_N**H**_**2**_), 8.26 (d, *J* = 8.2 Hz, 1H, Ar**H**), 8.41 (dd, *J* = 8.2 Hz, *J* = 1.7 Hz, 1H, Ar**H**), 8.48 (d, *J* = 1.7 Hz, 1H Ar**H**).

^**13**^ **C NMR** (100 MHz, DMSO-*d*_6_) δ ppm: 13.2, 16.5, 53.3, 58.9, 126.3, 128.7, 132.5, 133.4, 141.7, 149.2, 165.1.

HRMS calcd for C_11_H_15_NO_5_S_2_ [(M+H)^+^]: 306.0464, found: 306.0470.

#### Methyl 4-Isopentylsulfinyl-3-sulfamoyl-benzoate (**7k**)

The product was purified by flash chromatography on silica gel (CHCl_3_:EtOAc, 5:1). Yield: 37 mg, 55%, mp 98–99 °C.

^**1**^ **H NMR** (400 MHz, DMSO-*d*_6_) δ ppm: 0.86 (d, *J* = 6.5 Hz, 3H, C**H**_**3**_), 0.89 (d, *J* = 6.5 Hz, 3H, C**H**_**3**_), 1.40–1.45 (m, 1H, C**H**_**A**_H_B_CH), 1.63–1.68 (m, 2H, C**H**CH_3_ and CH_A_ **H**_**B**_CH), 2.66–2.69 (m, 1H, C**H**_**A**_H_B_SO), 3.15–3.19 (m, 1H, CH_A_ **H**_**B**_SO), 3.94 (s, 3H, C**H**_**3**_O), 7.97 (s, 2H, SO_2_N**H**_**2**_), 8.26 (d, *J* = 8.2 Hz, 1H, Ar**H**), 8.41 (dd, *J* = 8.2 Hz, *J* = 1.7 Hz, 1H, Ar**H**), 8.49 (d, *J* = 1.7 Hz, 1H, Ar**H**).

^**13**^ **C NMR** (100 MHz, DMSO-*d*_6_) δ ppm: 22.4, 22.8, 27.2, 31.4, 53.3, 55.1, 126.4, 128.7, 132.5, 133.4, 141.7, 149.1, 165.1.

HRMS calcd for C_13_H_19_NO_5_S_2_ [(M+H)^+^]: 334.0777, found: 334.0771.

#### Methyl 4-Cyclopentylsulfinyl-3-sulfamoyl-benzoate (**7l**)

The product was purified by flash chromatography on silica gel (CHCl_3_:EtOAc, 4:1). Yield: 51 mg, 77%, mp 109–111 °C.

^**1**^ **H NMR** (400 MHz, DMSO-*d*_6_) δ ppm: 1.08–1.18 (m, 1H, C**H** cyclopentyl), 1.43–1.53 (m, 1H, C**H** cyclopentyl), 1.54–1.66 (m, 3H, C**H** cyclopentyl), 1.80–1.94 (m, 2H, C**H** cyclopentyl), 2.00–2.11 (m, 1H, C**H** cyclopentyl), 3.53 (quint, *J* = 7.6 Hz, 1H, C**H** cyclopentyl), 3.94 (s, 3H, OC**H**_**3**_), 7.97 (s, 2H, SO_2_N**H**_**2**_), 8.16 (d, *J* = 8.4 Hz, 1H, Ar**H**), 8.36 (dd, *J* = 8.0 Hz, *J* = 1.6 Hz, 1H, Ar**H**), 8.49 (d, *J* = 1.3 Hz, 1H, Ar**H**).

^**13**^ **C NMR** (100 MHz, DMSO-*d*_6_) δ ppm: 21.3, 25.7, 26.3, 29.6, 53.3, 61.7, 126.6, 128.9, 132.4, 132.9, 141.7, 148.0, 165.1.

HRMS calcd for C_13_H_17_NO_5_S_2_ [(M+H)^+^]: 332.0621, found: 332.0615.

#### Methyl 4-(1-Naphthylsulfinyl)-3-sulfamoyl-benzoate (**7m**)

The product was purified by flash chromatography on silica gel (CHCl_3_:EtOAc, 1:1). Yield: 47 mg, 60%, mp 125–127 °C.

^**1**^ **H NMR** (400 MHz, DMSO-*d*_6_) δ ppm: 3.93 (s, 3H, C**H**_**3**_), 7.61–7.68 (m, 4H, naphthyl-**H**), 8.04–8.08 (m, 1H, naphthyl-**H**), 8.11 (s, 2H, SO_2_N**H**_**2**_), 8.14 (d, *J* = 7.6 Hz, 1H, naphthyl-**H**), 8.22 (d, *J* = 8.0 Hz, 1H, Ar**H**), 8.39 (dd, *J* = 1.6 Hz, *J* = 8.0 Hz, 1H, Ar**H**), 8.49–8.52 (m, 1H, naphthyl-**H**), 8.54 (d, 1H, *J* = 1.6 Hz, Ar**H**).

^**13**^ **C NMR** (100 MHz, DMSO-*d*_6_) δ ppm: 53.4, 123.5, 125.5, 126.3, 127.5, 128.0, 128.7, 128.8, 129.2, 129.8, 132.5, 133.2, 133.8 (2C), 141.9, 143.1, 146.7, 165.0.

HRMS calcd for C_18_H_15_NO_5_S_2_ [(M+H)^+^]: 390.0464, found: 390.0460.

#### 4-(Benzenesulfinyl)-*N*-butyl-3-sulfamoyl-benzamide (**8a**)

The product was purified by flash chromatography on silica gel (CHCl_3_:EtOAc, 1:1). Yield: 61 mg, 80%, mp 168–171 °C.

^**1**^ **H NMR** (400 MHz, DMSO-*d*_6_) δ ppm: 0.89 (t, *J* = 7.4 Hz, 3H, C**H**_**3**_),), 1.32 (sext, *J* = 7.8 Hz, 2H, NH­(CH_2_)_2_C**H**_**2**_CH_3_), 1.50 (quint, *J* = 7.2 Hz, 2H, NHCH_2_C**H**_**2**_), 3.27 (q, *J* = 7.2 Hz, 2H, NHC**H**_**2**_), 7.47–7.53 (m, 3H, Ar**H**), 7.75 (d, *J* = 6.4 Hz, 2H, Ar**H**), 7.97 (s, 2H, SO_2_N**H**_**2**_), 8.20 (s, 2H, Ar**H**), 8.35 (s, 1H, Ar**H**), 8.77 (t, *J* = 5.4 Hz, 1H, N**H**).

^**13**^ **C NMR** (100 MHz, DMSO-*d*_6_) δ ppm: 14.1, 20.1, 31.5, 39.6, 125.5, 126.4, 127.2, 129.8, 131.5, 131.7, 138.0, 142.0, 146.3, 147.3, 164.8.

HRMS calcd for C_17_H_20_N_2_O_4_S_2_ [(M+H)^+^]: 381.0937, found: 381.0933.

#### *N*-Butyl-4-cyclohexylsulfinyl-3-sulfamoyl-benzamide (**8b**)

The product was purified by flash chromatography on silica gel (CHCl_3_:EtOAc, 1:1). Yield: 63 mg, 82%, mp 195–196 °C.

^**1**^ **H NMR** (400 MHz, DMSO-*d*_6_) δ ppm: 0.93 (t, *J* = 7.3 Hz, 3H, C**H**_**3**_), 1.07–1.18 (m, 3H, C**H** cyclohexyl), 1.29–1.43 (m, 4H, C**H** cyclohexyl and C**H**_**2**_CH_3_), 1.49–1.60 (m, 4H, C**H** cyclohexyl and CH_2_ **CH**_**2**_CH_2_), 1.71–1.74 (m, 1H, C**H** cyclohexyl), 1.81–1.87 (m, 1H, C**H** cyclohexyl), 2.01–2.05 (m, 1H, C**H** cyclohexyl), 3.01 (tt, *J* = 12.3 Hz, *J* = 3.6 Hz, 1H, C**H**SO), 3.24–3.29 (m, 2H, C**H**_**2**_NH), 7.84 (s, 2H, SO_2_N**H**_**2**_), 8.02 (d, *J* = 8.2 Hz, 1H, Ar**H**), 8.24 (dd, *J* = 8.2 Hz, *J* = 1.7 Hz, 1H, Ar**H**), 8.41 (d, *J* = 1.7 Hz, 1H, Ar**H**), 8.82 (t, *J* = 5.7 Hz, 1H, N**H**).

^**13**^ **C NMR** (100 MHz, DMSO-*d*_6_) δ ppm: 14.2, 20.1, 21.0, 25.1, 25.4, 26.1, 28.5, 31.6, 39.5, 60.9, 126.6, 127.9, 130.3, 137.7, 141.7, 143.8, 164.9.

HRMS calcd for C_17_H_26_N_2_O_4_S_2_ [(M+H)^+^]: 387.1407, found: 387.1401.

#### *N*-Butyl-4-isopentylsulfinyl-3-sulfamoyl-benzamide (**8k**)

The product was purified by flash chromatography on silica gel (CHCl_3_:EtOAc, 1:1). Yield: 55 mg, 74%, mp 121–124 °C.

^**1**^ **H NMR** (400 MHz, DMSO-*d*_6_) δ ppm: 0.83 (d, *J* = 6.4 Hz, 6H, S­(CH_2_)_2_CH­(C**H**_**3**_)_2_), 0.92 (t, *J* = 7.2 Hz, 3H, NH­(CH_2_)_3_C**H**_**3**_), 1.35 (sext, *J* = 7.2 Hz, 2H, NHCH_2_CH_2_C**H**_**2**_), 1.45–1.66 (m, 5H, NHCH_2_C**H**_**2**_, SCH_2_C**H**_**2**_C**H**), 3.31 (q, *J* = 6.8 Hz, 2H, NHC **H**_ **2** _), 3.66–3.70 (m, 2H, SC**H**_**2**_), 7.33 (s, 2H, SO_2_N**H**_**2**_), 8.25 (d, *J* = 8.0 Hz, 1H, Ar**H**), 8.30 (dd, *J* = 8.0 Hz, *J* = 1.6 Hz, 1H, Ar**H**), 8.60 (d, *J* = 1.6 Hz, 1H, Ar**H**), 8.96 (t, *J* = 5.6 Hz, 1H, N**H**).

^**13**^ **C NMR** (100 MHz, DMSO-*d*_6_) δ ppm: 14.2, 20.1, 22.3, 27.0, 30.7, 31.5, 39.6, 53.4, 129.6, 131.5, 133.8, 138.1, 140.4, 143.1, 164.2.

HRMS calcd for C_16_H_26_N_2_O_4_S_2_ [(M+H)^+^]: 375.1407, found: 375.1411.

#### *N*-Butyl-4-cyclopentylsulfinyl-3-sulfamoyl-benzamide (**8l**)

The product was purified by flash chromatography on silica gel (CHCl_3_:EtOAc, 1:1). Yield: 47 mg, 63%, mp 103–107 °C.

^**1**^ **H NMR** (400 MHz, DMSO-*d*_6_) δ ppm: 0.92 (t, *J* = 7.2 Hz, 3H, C**H**_**3**_), 1.09–1.17 (m, 1H, C**H** cyclopentyl), 1.35 (sext, *J* = 7.2 Hz, 2H, NH­(CH_2_)_2_C**H**_**2**_CH_3_), 1.45–1.63 (m, 6H, NHCH_2_C**H**_**2**_, C**H** cyclopentyl), 1.82–1.94 (m, 2H, C**H** cyclopentyl), 2.01–2.10 (m, 1H, C**H** cyclopentyl), 3.26–3.33 (m, 2H, NHC**H**_**2**_), 3.52 (quint, *J* = 7.6 Hz, 1H, C**H** cyclopentyl), 7.85 (s, 2H, SO_2_N**H**_**2**_), 8.07 (d, *J* = 8.4 Hz, 1H, Ar**H**), 8.23 (dd, *J* = 8.0 Hz, *J* = 1.6 Hz, 1H, Ar**H**), 8.39 (d, *J* = 1.6 Hz, 1H, Ar**H**), 8.80 (t, *J* = 5.6 Hz, 1H, N**H**).

^**13**^ **C NMR** (100 MHz, DMSO-*d*_6_) δ ppm: 14.2, 20.1, 21.4, 25.8, 26.3, 29.6, 31.5, 39.6, 61.7, 125.9, 127.7, 130.6, 137.7, 141.3, 145.4, 164.9.

HRMS calcd for C_16_H_24_N_2_O_4_S_2_ [(M+H)^+^]: 373.1250, found: 373.1255.

#### *N*-Butyl-4-(1-naphthylsulfinyl)-3-sulfamoyl-benzamide (**8m**)

The product was purified by flash chromatography on silica gel (CHCl_3_:EtOAc, 1:1). Yield: 28 mg, 33%, mp 232–235 °C.

^**1**^ **H NMR** (400 MHz, DMSO-*d*_6_) δ ppm: 0.95 (t, *J* = 7.2 Hz, 3H, C**H**_**3**_), 1.38 (sext, *J* = 7.2 Hz, 2H, NH­(CH_2_)_2_C**H**_**2**_CH_3_), 1.56 (quint, *J* = 7.2 Hz, 2H, NHCH_2_C**H**_**2**_), 3.28–3.37 (m, 2H, NHC**H**_**2**_), 7.63–7.73 (m, 4H, naphthyl-**H**), 8.08 (s, 2H, SO_2_N**H**_**2**_), 8.11–8.13 (m, 2H, naphthyl-**H**, Ar**H**), 8.18–8.22 (m, 1H, naphthyl-**H**), 8.26 (dd, *J* = 1.6 Hz, *J* = 8.0 Hz, 1H, Ar**H**), 8.47 (d, 1H, *J* = 1.6 Hz, Ar**H**), 8.52–8.54 (m, 1H, naphthyl-**H**), 8.86 (t, *J* = 5.6 Hz, 1H, N**H**).

^**13**^ **C NMR** (100 MHz, DMSO-*d*_6_) δ ppm: 14.2, 20.1, 31.5, 39.6, 123.5, 125.3, 126.2, 127.4, 127.5, 128.0, 128.3, 129.2, 129.7, 131.5, 132.3, 133.8, 138.4, 142.0, 142.8, 144.3, 164.8.

HRMS calcd for C_21_H_22_N_2_O_4_S_2_ [(M+H)^+^]: 431.1094, found: 431.1088.

#### General Procedure for the Syntheses of **9­(a–d, h, i, l, m)** and **10­(b, k–m)**

The 30% H_2_O_2_(aq) (1.08 mmol, 0.110 mL) was added in small portions over 3 h to a solution of appropriate methyl 4-sulfanilsubstituted-3-sulfamoyl-benzoate (**4a–d, h, i, l, m**) or appropriate *N*-butyl-4-sulfanilsubstituted-3-sulfamoyl-benzamide (**10b, k–m**) (0.200 mmol) and acetic acid (2.0 mL) at 70 °C and allowed stirring for 6–8h. The solvent was removed under reduced pressure and the resultant precipitate was filtered, and washed with H_2_O.

#### Methyl 4-(Benzenesulfonyl)-3-sulfamoyl-benzoate (**9a**)

The product was purified by flash chromatography on silica gel (CHCl_3_:EtOAc, 5:1). Yield: 57 mg, 80%, mp 212–213 °C.

^**1**^ **H NMR** (400 MHz, DMSO-*d*_6_) δ ppm: 3.94 (s, 3H, C**H**_**3**_O), 7.55 (s, 2H, SO_2_N**H**_**2**_), 7.61 (t, *J* = 7.4 Hz, 2H, Ar**H**), 7.70 (t, *J* = 7.4 Hz, 1H, Ar**H**), 7.95 (d, *J* = 7.4 Hz, 2H, Ar**H**), 8.43 (dd, *J* = 8.2 Hz, *J* = 1.7 Hz, 1H, Ar**H**), 8.61 (d, *J* = 8.2 Hz, 1H, Ar**H**), 8,64 (d, *J* = 1.7 Hz, 1H, Ar**H**).

^**13**^ **C NMR** (100 MHz, DMSO-*d*_6_) δ ppm: 53.6, 128.4, 129.6, 130.7, 133.9, 134.3, 134.4, 135.1, 140.6, 141.6, 143.4, 164.5.

HRMS calcd for C_14_H_13_NO_6_S_2_ [(M+H)^+^]: 356.0257, found: 356.0266.

#### Methyl 4-Cyclohexylsulfonyl-3-sulfamoyl-benzoate (**9b**)

The product was purified by flash chromatography on silica gel (CHCl_3_:EtOAc, 5:1). Yield: 61 mg, 85%, mp 155–156 °C.

^**1**^ **H NMR** (400 MHz, DMSO-*d*_6_) δ ppm: 1.17–1.81 (m, 10H, C**H**_**2**_ cyclohexyl), 3.86–3.91 (m, 1H, C**H**SO_2_), 3.95 (s, 3H, C**H**_**3**_O), 7.42 (s, 2H, SO_2_N**H**_**2**_), 8.25 (d, *J* = 8.1 Hz, 1H, Ar**H**), 8.42 (dd, *J* = 8.1 Hz, *J* = 1.7 Hz, 1H, Ar**H**), 8.70 (d, *J* = 1.7 Hz, 1H, Ar**H**).

^**13**^ **C NMR** (100 MHz, DMSO-*d*_6_) δ ppm: 24.8, 24.9, 25.1, 53.6, 61.8, 130.9, 133.6, 134.9, 135.2, 138.6, 143.8, 164.6.

HRMS calcd for C_14_H_19_NO_6_S_2_ [(M+H)^+^]: 362.0727, found: 362.0721.

#### Methyl 4-Benzylsulfonyl-3-sulfamoyl-benzoate (**9c**)

The product was purified by flash chromatography on silica gel (CHCl_3_:EtOAc, 5:1). Yield: 66 mg, 89%, mp 189–190 °C.

^**1**^ **H NMR** (400 MHz, DMSO-*d*_6_) δ ppm: 3.94 (s, 3H, C**H**_**3**_O), 5.03 (s, 2H, C**H**_**2**_SO_2_), 7.19–7.21 (m, 2H, Ar**H**), 7.30–7.34 (m, 3H, Ar**H**), 7.53 (s, 2H, SO_2_N**H**_**2**_), 7.88 (d, *J* = 8.0 Hz, 1H, Ar**H**), 8.25 (dd, *J* = 8.0 Hz, *J* = 1.8 Hz, 1H, Ar**H**), 8.70 (d, *J* = 1.8 Hz, Ar**H**).

^**13**^ **C NMR** (100 MHz, DMSO-*d*_6_) δ ppm: 53.6, 60.9, 127.9, 129.0, 129.3, 130.6, 131.2, 133.4, 134.5, 135.1, 139.4, 143.7, 164.5.

HRMS calcd for C_15_H_15_NO_6_S_2_ [(M+H)^+^]: 370.0414, found: 370.0419.

#### Methyl 4-(2-Phenylethylsulfonyl)-3-sulfamoyl-benzoate (**9d**)

The product was purified by flash chromatography on silica gel (CHCl_3_:EtOAc, 5:1). Yield: 64 mg, 83%, mp 186–187 °C.

^**1**^ **H NMR** (400 MHz, DMSO-*d*_6_) δ ppm: 2.98 (t, *J* = 8.0 Hz, 2H, C**H**_**2**_Ph), 3.94 (s, 3H, C**H**_**3**_O), 4.02 (t, *J* = 8.0 Hz, 2H,C**H**_**2**_SO_2_), 7.18–7.27 (m, 5H, Ar**H**), 7.44 (s, 2H, SO_2_N**H**_**2**_), 8.30 (d, *J* = 8.0 Hz, 1H, Ar**H**), 8.40 (d, *J* = 8.0 Hz, 1H, Ar**H**), 8.67 (s, 1H, Ar**H**).

^**13**^ **C NMR** (100 MHz, DMSO-*d*_6_) δ ppm: 28.1, 53.6, 55.9, 127.1, 128.9, 129.0, 130.7, 133.9, 134.3, 135.1, 137.9, 140.0, 143.5, 164.5.

HRMS calcd for C_16_H_17_NO_6_S_2_ [(M+H)^+^]: 384.0570, found: 384.0578.

#### Methyl 4-Methylsulfonyl-3-sulfamoyl-benzoate (**9h**)

The product was purified by flash chromatography on silica gel (CHCl_3_:EtOAc, 5:1). Yield: 55 mg, 95%, mp 187–188 °C.

^**1**^ **H NMR** (400 MHz, DMSO-*d*_6_) δ ppm: 3.51 (s, 3H, C**H**_**3**_SO_2_), 3.96 (s, 3H, C**H**_**3**_O), 7.42 (s, 2H, SO_2_N**H**_**2**_), 8.36 (d, *J* = 8.2 Hz, 1H, Ar**H**), 8.45 (dd, *J* = 8.2 Hz, *J* = 1.6 Hz, 1H, Ar**H**), 8.67 (d, *J* = 1.6 Hz, 1H, Ar**H**).

^**13**^ **C NMR** (100 MHz, DMSO-*d*_6_) δ ppm: 44.1, 53.6, 130.5, 133.4, 134.1, 135.1, 141.6, 143.2, 164.6.

HRMS calcd for C_9_H_11_NO_6_S_2_ [(M+H)^+^]: 294.0101, found: 294.0106.

#### Methyl 4-Propylsulfonyl-3-sulfamoyl-benzoate (**9i**)

The product was purified by flash chromatography on silica gel (CHCl_3_:EtOAc, 5:1). Yield: 57 mg, 88%, mp 169–170 °C.

^**1**^ **H NMR** (400 MHz, DMSO-*d*_6_) δ ppm: 0.94 (t, *J* = 7.4 Hz, 3H, C**H**_**3**_CH_2_), 1.63–1.66 (m, 2H, C**H**_**2**_CH_3_), 3.66 (t, *J* = 7.7 Hz, 2H, C**H**_**2**_SO_2_), 3.96 (s, 3H, C**H**_**3**_O), 7.43 (s, 2H, SO_2_N**H**_**2**_), 8.33 (d, *J* = 8.1 Hz, 1H, Ar**H**), 8.44 (dd, *J* = 8.1 Hz, *J* = 1.6 Hz, 1H, Ar**H**), 8.69 (d, *J* = 1.6 Hz, 1H, Ar**H**).

^**13**^ **C NMR** (100 MHz, DMSO-*d*_6_) δ ppm: 13.1, 16.2, 53.6, 56.7, 130.7, 133.9, 134.2, 135.2, 140.2, 143.5, 164.6.

HRMS calcd for C_11_H_15_NO_6_S_2_ [(M+H)^+^]: 322.0414, found: 322.0422.

#### Methyl 4-Cyclopentylsulfonyl-3-sulfamoyl-benzoate (**9l**)

The product was purified by flash chromatography on silica gel (CHCl_3_:EtOAc, 5:1). Yield: 63 mg, 90%, mp 199–200 °C.

^**1**^ **H NMR** (400 MHz, DMSO-*d*_6_) δ ppm: 1.55–1.65 (m, 2H, C**H** cyclopentyl), 1.68–1.76 (m, 2H, C**H** cyclopentyl), 1.77–1.86 (m, 2H, C**H** cyclopentyl), 1.88–1.96 (m, 2H, C**H** cyclopentyl), 3.96 (s, 3H, OC**H**_**3**_), 4.44 (quint, *J* = 7.2 Hz, 1H, C**H** cyclopentyl), 7.41 (s, 2H, SO_2_N**H**_**2**_), 8.34 (d, *J* = 8.0 Hz, 1H, Ar**H**), 8.42 (dd, *J* = 8.0 Hz, *J* = 1.6 Hz, 1H, Ar**H**), 8.70 (d, *J* = 1.6 Hz, 1H, Ar**H**).

^**13**^ **C NMR** (100 MHz, DMSO-*d*_6_) δ ppm: 26.0, 27.0, 53.6, 62.7, 130.9, 133.8, 134.6, 135.1, 139.7, 143.6, 164.6.

HRMS calcd for C_13_H_17_NO_6_S_2_ [(M+H)^+^]: 348.0570, found: 348.0560.

#### Methyl 4-(1-Naphthylsulfonyl)-3-sulfamoyl-benzoate (**9m**)

The product was purified by flash chromatography on silica gel (CHCl_3_:EtOAc, 8:1). Yield: 63 mg, 78%, mp 223–224 °C.

^**1**^ **H NMR** (400 MHz, DMSO-*d*_6_) δ ppm: 3.92 (s, 3H, OC**H**_**3**_), 7.63–7.67 (m, 4H, naphthyl**H** and SO_2_N**H**_**2**_), 7.74 (t, *J* = 8.0 Hz, 1H, Ar**H**), 8.12–8.14 (m, 1H, naphthyl**H**), 8.25 (d, *J* = 8.0 Hz, 1H, Ar**H**), 8.31–8.37 (m, 4H, naphthyl**H**), 8.70 (s, 1H, Ar**H**).

^**13**^ **C NMR** (100 MHz, DMSO-*d*_6_) δ ppm: 53.6, 123.9, 125.0, 127.5, 127.8, 129.1, 129.9, 130.8, 131.0, 133.0, 133.8, 134.2, 134.9, 135.2, 135.9, 141.8, 143.5, 164.5.

HRMS calcd for C_18_H_15_NO_6_S_2_ [(M+H)^+^]: 406.0414, found: 406.0423.

#### *N*-Butyl-4-cyclohexylsulfonyl-3-sulfamoyl-benzamide (**10b**)

The product was purified by flash chromatography on silica gel (CHCl_3_:EtOAc, 3:1). Yield: 68 mg, 84%, mp 155–156 °C.

^**1**^ **H NMR** (400 MHz, DMSO-*d*_6_) δ ppm: 0.93 (t, *J* = 7.3 Hz, 3H, C**H**_**3**_), 1.17–1.81 (m, 14H, C**H**_**2**_C**H**_**2**_CH_3_ and C**H** cyclohexyl), 3.30–3.32 (m, 2H, C**H**_**2**_NH), 3.90–3.93 (m, 1H, C**H**SO_2_), 7.33 (s, 2H, SO_2_N**H**_**2**_), 8.19 (d, *J* = 8.1 Hz, 1H, Ar**H**), 8.29 (d, *J* = 8.1 Hz, 1H, Ar**H**), 8.61 (s, 1H, Ar**H**), 8.96 (t, *J* = 5.3 Hz, 1H, N**H**).

^**13**^ **C NMR** (100 MHz, DMSO-*d*_6_) δ ppm: 14.1, 20.1, 24.9, 24.9, 25.1, 31.5, 39.6, 61.8, 129.8, 131.2, 134.4, 136.5, 140.4, 143.4, 164.2.

HRMS calcd for C_17_H_26_N_2_O_5_S_2_ [(M+H)^+^]: 403.1356, found: 403.1351.

#### *N*-Butyl-4-isopentylsulfonyl-3-sulfamoyl-benzamide (**10k**)

The product was purified by flash chromatography on silica gel (CHCl_3_:EtOAc, 1:1). Yield: 53 mg, 68%, mp 160–162 °C.

^**1**^ **H NMR** (400 MHz, DMSO-*d*_6_) δ ppm: 0.83 (d, *J* = 6.4 Hz, 6H, S­(CH_2_)_2_CH­(C**H**_**3**_)_**2**_), 0.92 (t, *J* = 7.6 Hz, 3H, NH­(CH_2_)_3_C**H**_**3**_), 1.35 (sext, *J* = 7.2 Hz, 2H, NHCH_2_CH_2_C**H**_**2**_), 1.46–1.66 (m, 5H, NHCH_2_C**H**_**2**_, SCH_2_C**H**_**2**_C**H**), 3.31 (q, *J* = 6.8 Hz, 2H, NHC**H**_**2**_), 3.66–3.70 (m, 2H, SC**H**_**2**_), 7.33 (s, 2H, SO_2_N**H**_**2**_), 8.25 (d, *J* = 8.0 Hz, 1H, Ar**H**), 8.30 (dd, *J* = 8.0 Hz, *J* = 1.6 Hz, 1H, Ar**H**), 8.60 (s, 1H, Ar**H**), 8.97 (t, *J* = 5.2 Hz, 1H, N**H**).

^**13**^ **C NMR** (100 MHz, DMSO-*d*_6_) δ ppm: 14.2, 20.1, 22.3, 27.0, 30.7, 31.5, 39.7, 53.4, 129.6, 131.5, 133.8, 138.1, 140.4, 143.1, 164.2.

HRMS calcd for C_16_H_26_N_2_O_5_S_2_ [(M+H)^+^]: 391.1356, found: 391.1359.

#### *N*-Butyl-4-cyclopentylsulfonyl-3-sulfamoyl-benzamide (**10l**)

The product was purified by flash chromatography on silica gel (CHCl_3_:EtOAc, 1:1). Yield: 57 mg, 73%, mp 143–145 °C.

^**1**^ **H NMR** (400 MHz, DMSO-*d*_6_) δ ppm: 0.92 (t, *J* = 7.6 Hz, 3H, CH_2_C**H**_**3**_), 1.35 (sext, *J* = 7.6 Hz, 2H, CH_2_C**H**_**2**_CH_3_), 1.54 (quint, *J* = 7.2 Hz, 2H, NHCH_2_C**H**_**2**_), 1.57–1.65 (m, 2H, C**H** cyclopentyl), 1.68–1.85 (m, 4H, C**H** cyclopentyl), 1.88–1.96 (m, 2H, C**H** cyclopentyl), 3.31 (q, *J* = 6.8 Hz, 2H, NHC**H**_**2**_), 4.44 (quint, *J* = 7.2 Hz, 1H, SO_2_C**H** cyclopentyl), 7.32 (br. s, 2H, SO_2_N**H**_**2**_), 8.26–8.31 (m, 2H, Ar**H**), 8.62 (s, 1H, Ar**H**), 8.96 (t, *J* = 5.6 Hz, 1H, N**H**).

^**13**^ **C NMR** (100 MHz, DMSO-*d*_6_) δ ppm: 14.2, 20.1, 26.0, 27.0, 31.5, 39.6, 62.6, 129.7, 131.4, 134.1, 137.7, 140.3, 143.2, 164.2.

HRMS calcd for C_16_H_24_N_2_O_5_S_2_ [(M+H)^+^]: 389.1199, found: 389.1205.

#### *N*-Butyl-4-(1-naphthylsulfonyl)-3-sulfamoyl-benzamide (**10m**)

The product was purified by flash chromatography on silica gel (CHCl_3_:EtOAc, 1:1). Yield: 22 mg, 24%, mp 266–268 °C.

^**1**^ **H NMR** (400 MHz, DMSO-*d*_6_) δ ppm: 0.89 (t, *J* = 7.6 Hz, 3H, CH_2_C**H**_**3**_), 1.32 (sext, *J* = 7.2 Hz, 2H, CH_2_C**H**_**2**_CH_3_), 1.51 (quint, *J* = 7.2 Hz, 2H, NHCH_2_C**H**_**2**_), 3.29 (q, *J* = 6.4 Hz, 2H, NHC**H**_**2**_), 7.52 (s, 2H, SO_2_N**H**_**2**_), 7.62–7.68 (m, 2H, naphthyl**H**), 7.74 (t, *J* = 8.0 Hz, 1H, naphthyl**H**), 8.13–8.15 (m, 1H, naphthyl**H**), 8.19–8.23 (m, 2H, Ar**H**), 8.30 (d, *J* = 8.4 Hz, 1H, naphthyl**H**), 8.34–8.39 (m, 2H, naphthyl**H**), 8.60 (d, *J* = 1.6 Hz, 1H, Ar**H**), 8.92 (t, *J* = 5.2 Hz, 1H, N**H**).

^**13**^ **C NMR** (100 MHz, DMSO-*d*_6_) δ ppm: 14.1, 20.1, 31.4, 39.6, 124.0, 125.0, 127.5, 127.8, 129.0, 129.8, 129.9, 130.7, 131.4, 132.7, 134.2, 135.5, 135.8, 139.8, 140.2, 143.1, 164.3.

HRMS calcd for C_21_H_22_N_2_O_5_S_2_ [(M+H)^+^]: 447.1043, found: 447.1048.

### Protein Preparation

Recombinant human carbonic anhydrases (CAI, CAII, CAIII, CAIV, CAVA, CAVB, CAVI, CAVII, CAIX, CAXII, CAXIII, CAXIV) were expressed and chromatographically purified according to previously published protocols^47^ and were used for FTSA experiments. Proteins (CAII and CAXIII) used for crystallization were additionally purified by affinity chromatography and concentrated. CAIX used for crystallization were expressed and purified according to this protocol.^48^ Production of recombinant CAII and the extracellular part of CAIX comprising PG and CA domains (residues 38–391) used in SFA experiments is described in ref 49.

### Determination of Observed Binding Parameters

#### Fluorescent Thermal Shift Assay (FTSA)

Dissociation constants, *K*_d,obs_, (listed in Table 1) of the compounds binding to CAs were determined by the fluorescent thermal shift assay using a QIAGEN’s real-time PCR cycler the “Rotor-Gene Q” and Rotor-Gene Style 4-strip tubes from STARLAB. Ligands were dissolved in DMSO stock solutions to concentrations of 10 mM or 20 mM and used for the serial dilution of the dilution factor 2 in DMSO. These samples were diluted with buffer solution and mixed with a prepared protein solution, consisting of protein stock, buffer solution, and solvatochromic dye 8-anilino-1-naphthalenesulfonate (ANS). All final samples typically contained up to 10 μM CA, compound solutions of serial dilution from 0 μM to 200 μM at 8 different concentrations differing by 2 times, 50 μM ANS, 50 mM sodium phosphate buffer at pH 7.0, 100 mM sodium chloride, and 2.0% (v/v) DMSO. Samples preparation is explained in detail in.^50^ The excitation and emission wavelengths of ANS were 365 ± 20 and 460 ± 15 nm. Samples were heated from 25 to 99 °C at the rate of 1 °C/min. The curve-fitting procedure was performed by Thermott^51^ at 37 °C. Data are deposited in the public database: Protein–Ligand Binding Database^52^ (Database URL: https://plbd.org/).

#### Stopped-Flow Carbon Dioxide Hydration Assay

Recombinant CAII and the extracellular part of CAIX comprising PG and CA domains (residues 38–391) were used in inhibition assays. A stopped-flow instrument (BioLogic) was used for measuring the CA-catalyzed CO_2_ hydration activity in the presence of inhibitors.^53^ The assay buffer consisted of 0.2 mM Phenol Red (pH indicator used in absorbance maximum of 557 nm), 20 mM HEPES Na (pH 7.5), and 20 mM Na_2_SO_4_. The concentration of CAII and CAIX in the enzyme assay was 4 nM and 1 nM, respectively. To stabilize CAIX during the measurements, 0.0025% dodecyl-β-D-maltopyranoside (DDM, Anatrace, and Anagrade purity) was included in the reaction mixture.

The substrate (CO_2_) concentration in the reaction was 8.5 mM. Rates of the CA-catalyzed CO_2_ hydration reaction were followed for 30 s at 25 °C. Four traces of substrate conversion in the reaction were fitted by the exponential function to determine the rate for each inhibitor concentration. The uncatalyzed rates were determined in the same manner and subtracted from the total observed rates. Stock solutions of inhibitors (100 mM) were prepared in dimethyl sulfoxide, and dilutions of up to 100 nM were made thereafter in DMSO. Apparent *K*_i_’ values were obtained from dose–response curves recorded for at least six different concentrations of the test compound by the nonlinear least-squares method using an Excel spreadsheet fitting the Williams-Morrison equation.^54^ *K*_i_ values were then derived using the Cheng-Prusoff equation.^55^ The *K*_m_ values used in the Cheng–Prusoff equation were 9.3 mM for CAII and 7.5 mM for CAIX. Inhibition data are provided in Figure S2.

### Calculation of the Intrinsic Binding Parameters

The detailed description of the importance and calculation of the intrinsic values have been previously described.^27^ For the calculation of the *intrinsic* dissociation constants, the experimentally measured *observed* dissociation constants determined by the FTSA, the p*K*_a_ of the sulfonamide group of the compound, and the p*K*_a_ of the water molecule bound to the zinc cation by the CA were used.

The intrinsic dissociation constant, *K*_d,int_, is equal to

$$K_{d,\mathrm{int}}=K_{d,\mathrm{obs}}\times f_{{\mathrm{RSO}}_{2}{\mathrm{NH}}^{-}}\times f_{\mathrm{CAZn}H_{2}O}$$ 1

The fraction of deprotonated sulfonamide:

$$f_{{\mathrm{RSO}}_{2}{\mathrm{NH}}^{-}}=\frac{10^{\mathrm{pH}-pK_{a,\mathrm{SA}}}}{1+10^{\mathrm{pH}-pK_{a,\mathrm{SA}}}}$$ 2

The fraction of Zn­(II)-bound water form of CA:

$$f_{\mathrm{CAZn}H_{2}O}=\frac{10^{pK_{a,\mathrm{CA}}-\mathrm{pH}}}{1+10^{pK_{a,\mathrm{CA}}-\mathrm{pH}}}$$ 3

- *K*_d,obs_ – observed dissociation constant;
-
$f_{{\mathrm{RSO}}_{2}{\mathrm{NH}}^{-}}$ and $f_{\mathrm{CAZn}H_{2}O}$ – fractions of deprotonated sulfonamide and Zn­(II)-bound water molecule;
-
$pK_{a,\mathrm{SA}}$ – p*K*_a_ of the sulfonamide group;
- p*K*_a,CA_ – p*K*_a_ value of water molecule bound to Zn­(II) in the active site of CA.

In this study, the pH value was equal to 7.0.

#### Determination of p*K*_a_ Values of the Compound Sulfonamide Group

The p*K*_a_ values of the water molecule bound to Zn^2+^ in the active site of CAs, p*K*_a,CA_, were taken from ref 42 and of compounds, p*K*_a,SA_, (Figure S3) were experimentally determined as described in ref 56.

We used a constant concentration of sulfonamide (25–400 μM) and 2.0% (v/v) or 20% (v/v; but only for very poorly soluble ones) of DMSO in universal buffer (50 mM sodium acetate, 25 mM sodium borate, and 50 mM sodium phosphate) at different pH values (in the range from pH 6.0 to 12.0 with 0.5 pH increment). UV/vis spectra of the compound solution were recorded at 37 °C using BMG Labtech CLARIOstarPlus plate reading spectrophotometer. The p*K*_a_ values were calculated by normalizing the absorbance and plotting it as a function of pH, then fitting it to the Henderson–Hasselbalch equation using the least-square method. The midpoint of this fitted curve is equal to the sulfonamide group p*K*_a,SA_.

### X-ray Crystallography

#### Crystallization

Crystals of CAII, CAIX, and CAXIII were obtained using the sitting-drop vapor diffusion method at room temperature. Table 4 lists the concentrations of proteins and buffers used for crystallization.

**Table 4:** Crystallization Conditions Used to Grow Protein Crystals in This Study

**PDB ID**	**isozyme—compound**	**cystallization buffer**	**sitting drop**
9FPT	CAII—**2**	0.1 M sodium bicine (pH 9.0) and 2 M sodium malonate (pH 7.0)	2 μL of 41 mg/mL CAII solution and 2 μL of crystallization buffer
9FPU	CAII—**3**	0.1 M sodium bicine (pH 9.0) and 2 M sodium malonate (pH 7.0)	2 μL of 41 mg/mL CAII solution and 2 μL of crystallization buffer
9FPQ	CAII—**4c**	0.1 M sodium bicine (pH 9.0), 0.2 M ammonium sulfate, and 2 M sodium malonate (pH 7.0)	2 μL of 41 mg/mL CAII solution and 2.5 μL of crystallization buffer
9FPR	CAII—**4d**	0.1 M sodium bicine (pH 9.0), 0.2 M ammonium sulfate, and 2 M sodium malonate (pH 7.0)	3 μL of 12 mg/mL CAII solution and 3 μL of crystallization buffer
9FPS	CAII—**4h**	0.1 M sodium bicine (pH 9.0) and 2 M sodium malonate (pH 7.0)	3 μL of 12 mg/mL CAII solution and 3 μL of crystallization buffer
9R8X	CAIX—**4d**	1.0 M diammonium hydrogen phosphate and 0.1 M sodium acetate (pH 4.5)	10 mg/mL CAIX solution
9R8Y	CAIX—**5b**	1.0 M diammonium hydrogen phosphate and 0.1 M sodium acetate (pH 4.5)	10 mg/mL CAIX solution
9FPV	CAXIII—**4c**	0.1 M sodium citrate (pH 5.5), 0.1 M sodium acetate (pH 4.5), and 26% of PEG4000	1 μL of 23 mg/mL CAXIII solution and 0.4 μL of crystallization buffer
9FPW	CAXIII—**4d**	0.1 M sodium citrate (pH 5.5), 0.1 M sodium acetate (pH 4.5), and 26% of PEG4000	1 μL of 23 mg/mL CAXIII solution and 0.4 μL of crystallization buffer

#### Ligand Soaking

The crystal structures of CAII and CAXIII with ligands were obtained by soaking. A 50 mM solution of each ligand was prepared in dimethyl sulfoxide. One μL of this solution was then diluted using 50 μL of matching reservoir solution corresponding to the conditions under which the crystal was formed. Crystals were incubated for up to 1 week in the soaking solution.

#### Cocrystallization

The crystal structures of CAIX with ligands were obtained by cocrystallization. Table 4 lists crystallization conditions. The ligand used for cocrystallization was in 5–10 mM concentration, while the stock solution contained 100 mM ligand dissolved in dimethyl sulfoxide.

#### Data Collection and Structure Determination

Data for CAII and CAXII were collected at PETRA III BEAMLINE P13 (MX1) and for CAIX at BESSY II beamline 14.1.

The data were processed and scaled using XDS,^58^ MOSFLM,^59,60^ and SCALA.^61^ The crystal structures were solved by molecular replacement using MOLREP.^62^ The initial model for molecular replacement–CAII: 3HLJ; CAIX: 8Q18,^63^ CAXIII: 2NNO. The structure was refined by REFMAC^64^ and remodeled using COOT.^57^ The 3D models of compounds were constructed by the AVOGADRO^65^ program and ligand parameter files were created using LIBCHECK.^66,67^

The data diffraction and final model refinement statistics and PDB IDs are summarized in Table 3. All graphics were created using PyMOL Molecular Graphics System. Authors will release the atomic coordinates upon article publication.

### Molecular Docking

The following receptors PDB IDs were chosen for docking: CAII: 3HS4; CAIX: 6G9U, chain A; CAXIII: 4KNN, chain A. The receptors selected from the Protein Data Bank differed from the new X-ray structures presented in this Paper to decrease bias. The proteins were prepared for docking using ChimeraX (version 1.9).^68–70^ The ligands were optimized using the MMFF94s force field^71–76^ within Avogadro molecular viewer (version 1.2.0).^65^ For series **7** ligands, two enantiomers of the chiral sulfur were created and docked separately. The format conversions were performed using OpenBabel (The Open Babel Package, version 3.1.1, http://openbabel.org).^77^ The docking was performed using the Smina program (version master:dc3dfab+).^34^ Smina is based on Autodock Vina.^36^ The constrained optimization was done using Smina, using a custom scoring function with a quadratic bias function with weight *w*=–10 added to the default Vina scoring function.^36^ The quadratic constraint forced sulfonamide nitrogen to adhere to its position in the X-ray structure. A cubic docking box of size (24 Å),^3^ exhaustiveness 100, and energy range 10 kcal/mol was set as docking parameters. The resulting poses were rescored with Smina using the Vinardo scoring function^35^ without the constraint. Only one of the symmetry equivalent poses (e.g., phenyl ring flip) was included when evaluating pose ranking after docking. Heavy atom Root Mean Square Deviations (RMSD) were calculated using DockRMSD software (version 1.1).^78^

The quantum Density Functional Theory (DFT) optimizations were performed using GAMESS-US (version 2019.R2)^40^ using DFT functional ωB97X-D^79^ with the cc-pVDZ basis set^80,81^ and the C-PCM implicit solvation model for water.^82^ The conformational search was performed using CREST software (version 3.0.2)^38^ using xTB (version 6.7.1)^83^ computational engine, employing the GFN2-xTB semiempirical tight binding method^39^ and the ALPB implicit solvation model for water.^84^

## Supplementary Material





## Notes

FTSA data of compound binding to CA isozymes; SFA data of CA isozyme inhibition by compounds; determination of p*K*_a_ values of compound – RSO_2_NH_2_; NMR spectra of compounds; HPLC chromatograms of representative compounds; ESI-MS spectra of representative compounds; and comparison of docking and crystallographic binding poses (PDF)
SMILE strings of the synthesized compound and corresponding biological activity: *observed* and *intrinsic* (*K*_d,obs_ and *K*_d,int_) dissociation constants (in nM units) of investigated compounds to all catalytically active human CAs at 37 °C obtained by FTSA (CSV)

## Abbreviations

CA: human carbonic anhydrase
FTSA: fluorescent thermal shift assay (or differential scanning fluorimetry DSF)
int: intrinsic
*K*_d,int_: intrinsic equilibrium dissociation constant
*K*_d,obs_: observed equilibrium dissociation constant
obs: observed
p*K*_a,CA_: p*K*_a_ value of water molecule bound to Zn­(II) in the active site of CA
p*K*_a,SA_: p*K*_a_ of the sulfonamide group
SFA: stopped-flow carbon dioxide hydration assay
