# Supplementary material for: Affinity and Selectivity of Protein–Ligand Recognition: A Minor Chemical Modification Changes Carbonic Anhydrase Binding Profile
Source: J Med Chem. 2025 Aug 13;68(16):17752–73. doi: 10.1021/acs.jmedchem.5c01421 (PMC12406193; doi:10.1021/acs.jmedchem.5c01421)
Supplement: Supplementary file 1 [file jm5c01421_si_001.pdf]

Supplementary information for the manuscript:

Affinity and Selectivity of Protein–Ligand Recognition: A Minor Chemical Modification Changes Carbonic Anhydrase Binding Profile

Audrius Zakšauskas[a], Vaida Paketurytė-Latvė[a], Alberta Jankūnaitė[a], Edita Čapkauskaitė[a], Yann Becart[a], Alexey Smirnov[a], Klára Pospíšilová[b], Janis Leitans[c], Jiří Brynda[b], Andris Kazaks[c], Lina Baranauskienė[a], Elena Manakova[d], Saulius Gražulis[d], Visvaldas Kairys[e], Kaspars Tars[c], Pavlína Řezáčová[b], and Daumantas Matulis[a,*]

[a] Department of Biothermodynamics and Drug Design, Institute of Biotechnology, Life Sciences Center, Vilnius University, Saulėtekio al. 7, Vilnius LT-10257, Lithuania.

[b] Institute of Organic Chemistry and Biochemistry of the Czech Academy of Sciences, Flemingovo n. 2, Prague 6 16610, Czech Republic.

[c] Latvian Biomedical Research and Study Centre, Ratsupites 1 k-1, Riga LV-1067, Latvia

[d] Department of Protein–DNA Interactions, Institute of Biotechnology, Life Sciences Center, Vilnius University, Saulėtekio al. 7, Vilnius LT-10257, Lithuania.

[e] Department of Bioinformatics, Institute of Biotechnology, Life Sciences Center, Vilnius University, Saulėtekio 7, Vilnius, LT-10257, Lithuania

* Corresponding author: Daumantas Matulis
Address:
Saulėtekio al. 7, Vilnius LT-10257, Lithuania.
Email: daumantas.matulis@bti.vu.lt, matulis@ibt.lt

Contents

FTSA data of compound binding to CA isozymes S3

SFA data of CA isozyme inhibition by compounds S7

Determination of pK_a values of compound $–RSO_2NH_2$ S10

Comparison of docking and crystallographic binding poses S27

NMR spectra of synthesized compounds S29

HPLC chromatograms of representative compounds S76

ESI-MS spectra of representative compounds S79

FTSA data of compound binding to CA isozymes

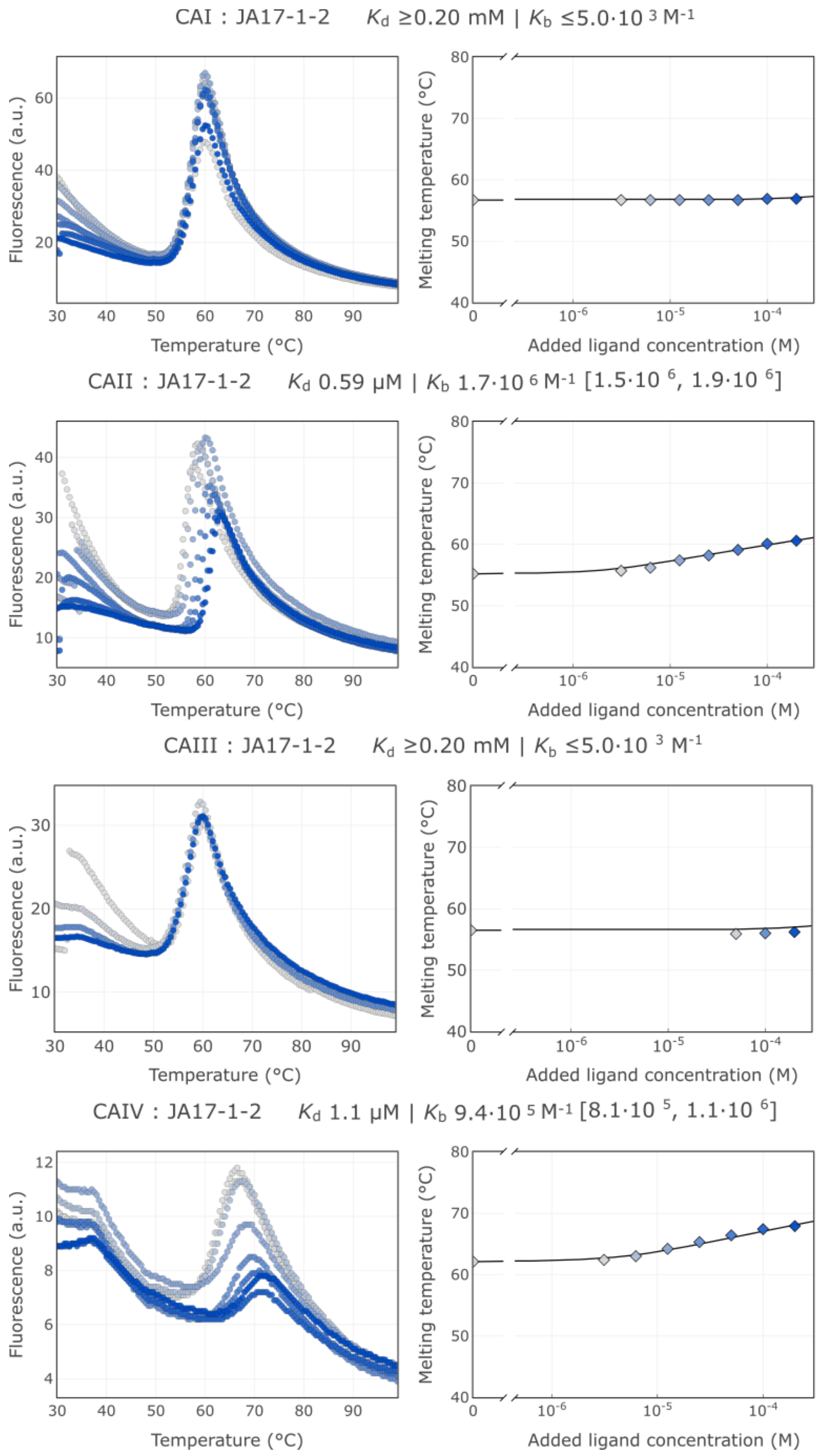

CAVA : JA17-1-2 K_d ≥0.20 mM | K_b ≤$5.0 \cdot 10^{3}$ M^{-1}

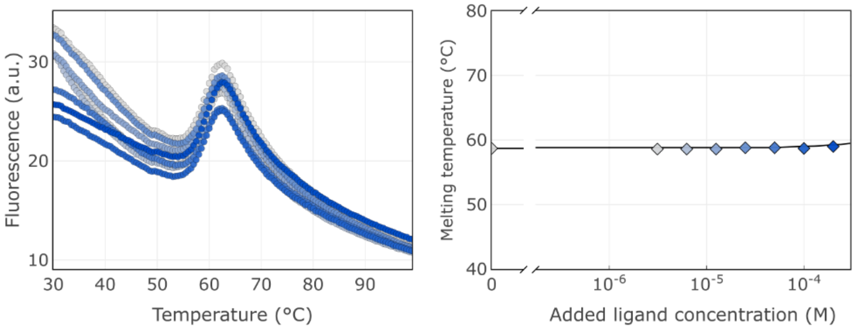

CAVB : JA17-1-2 K_d 1.2 μM | K_b $8.7 \cdot 10^{5}$ M^{-1} [$7.7 \cdot 10^{5}$, $1.0 \cdot 10^{6}$]

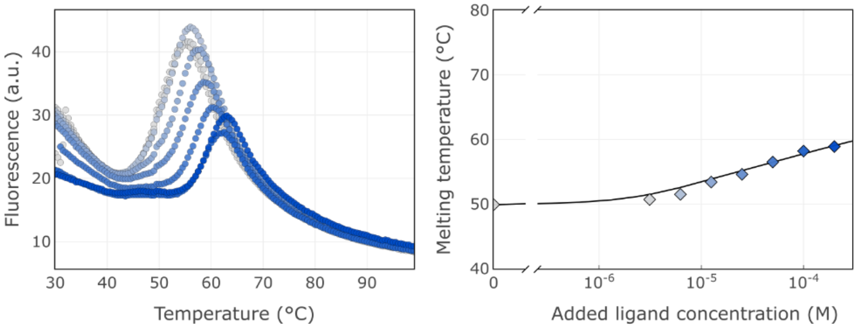

CAVII : JA17-1-2 K_d 0.73 μM | K_b $1.4 \cdot 10^{6}$ M^{-1} [$1.2 \cdot 10^{6}$, $1.6 \cdot 10^{6}$]

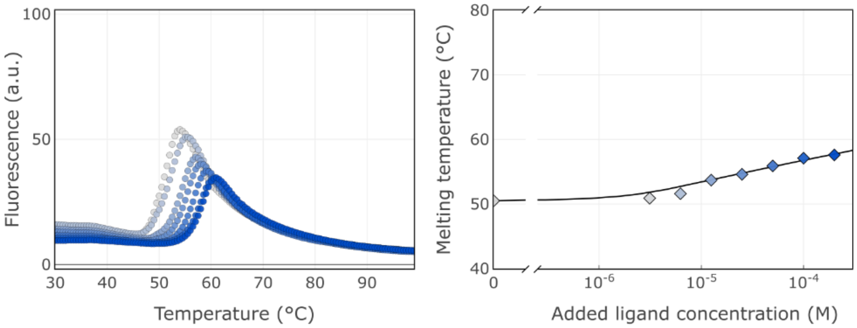

CAVI : JA17-1-2 K_d 1.1 μM | K_b $9.4 \cdot 10^{5}$ M^{-1} [$8.7 \cdot 10^{5}$, $1.0 \cdot 10^{6}$]

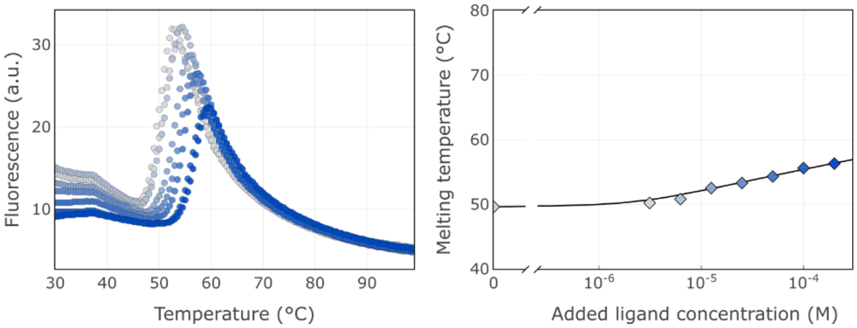

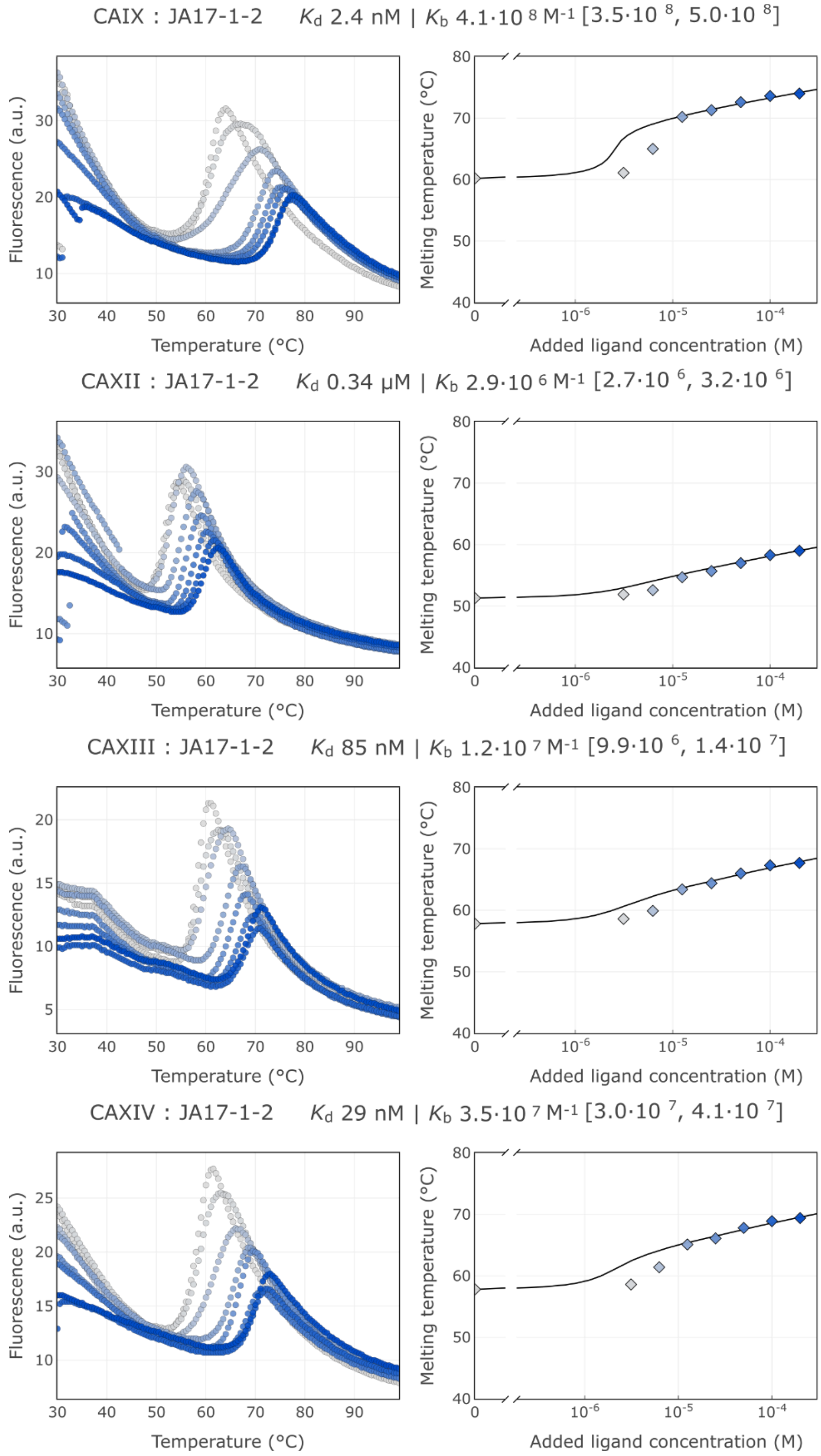

Figure S1. Fluorescent thermal shift assay data of compound **4b** binding to CAI, II, III, IV, VA, VB, VI, VII, IX, XII, XIII, and XIV in 50 mM sodium phosphate buffer at pH 7.0 containing 100 mM sodium chloride, 50 µM ANS dye and 2 % (v/v) DMSO, fitted at 37 °C. The figures on the left represent the thermal denaturation fluorescence curves of the protein at different compound concentrations. The

fluorescence curves are fit to determine the melting temperature at each condition. The plots on the right show the dependence of the melting temperature on the added ligand concentration. The experimental data points were fitted by Thermott, yielding the dosing curve line and the dissociation constants for the interaction of all compounds with 12 CA isozymes, listed in **Table 1.** The confidence interval for each experiment is shown next to the binding constant. The dissociation constants K_ds correspond to the constants denoted $K_{d,obs}$ in the table. **Table 1** presents geometric mean values from multiple replicates, while the figures show values from a single experiment, so they may not match.

SFA data of CA isozyme inhibition by compounds

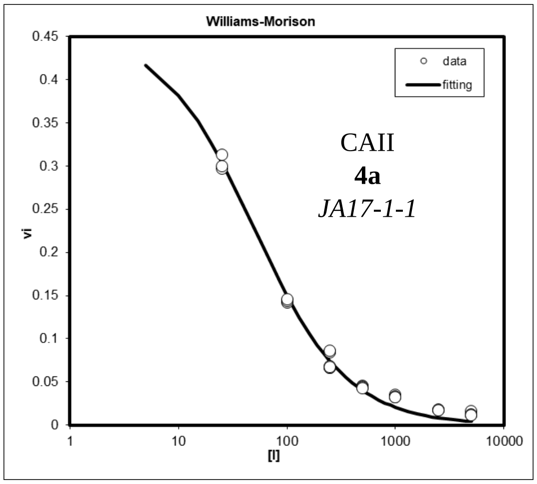

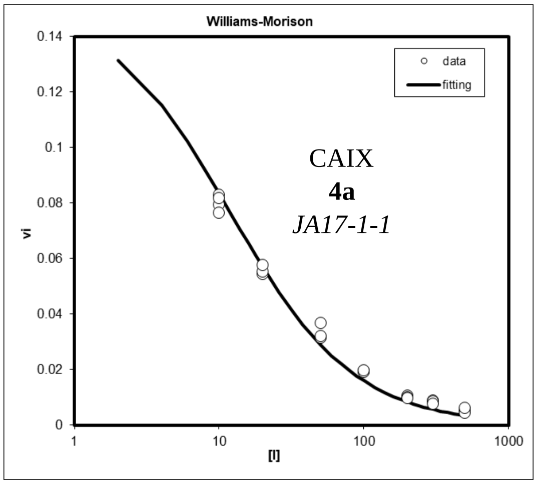

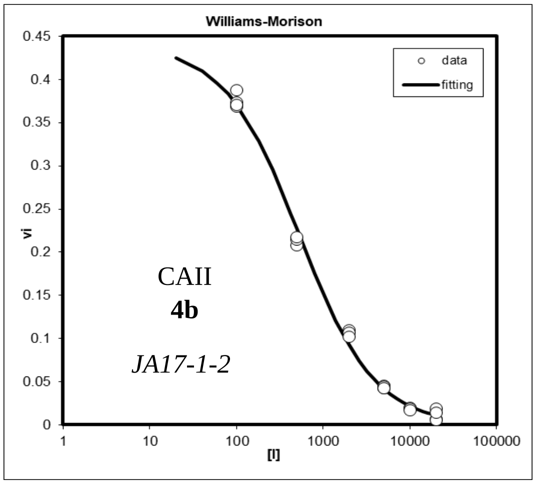

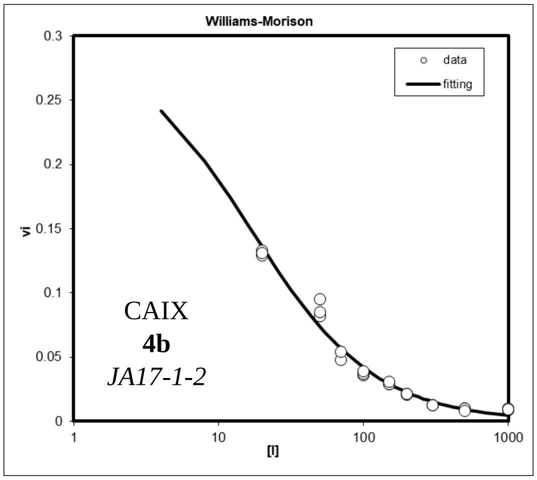

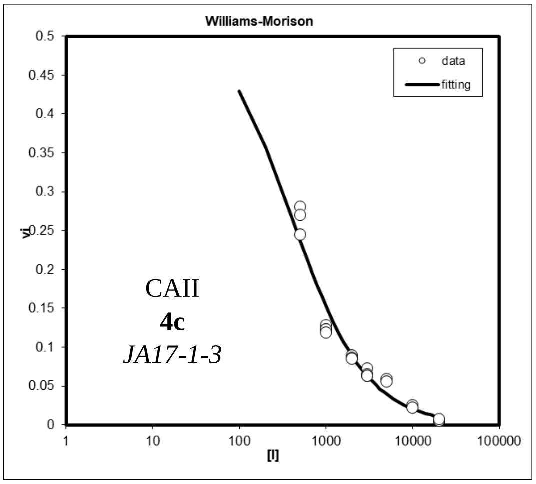

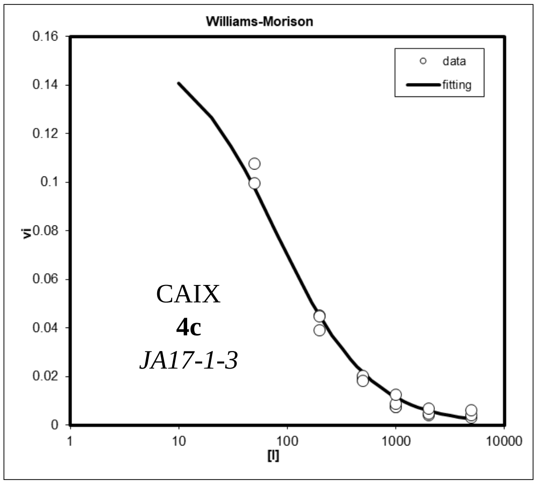

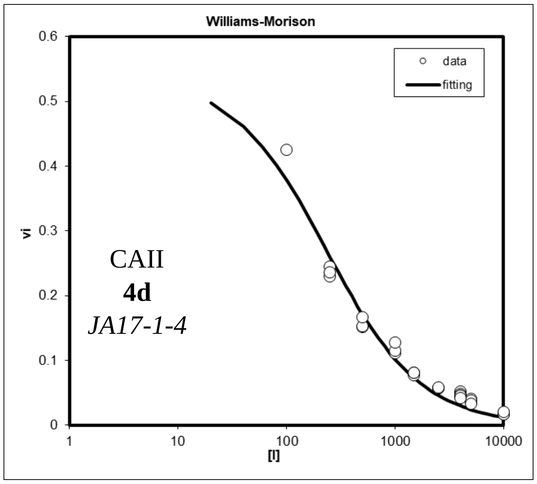
Williams-Morison
data
fitting
vi
[I]
CAII
4d
JA17-1-4

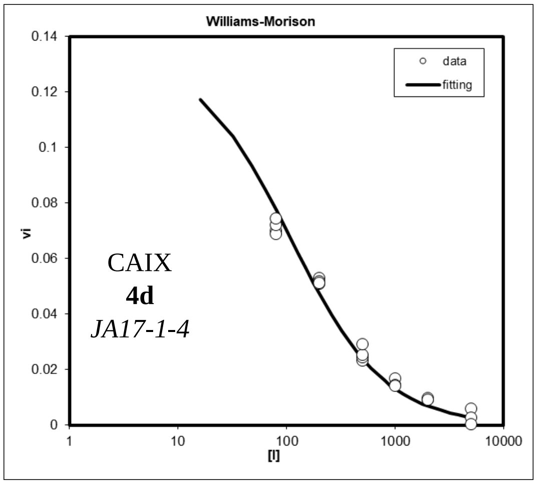
Williams-Morison
data
fitting
vi
[I]
CAIX
4d
JA17-1-4

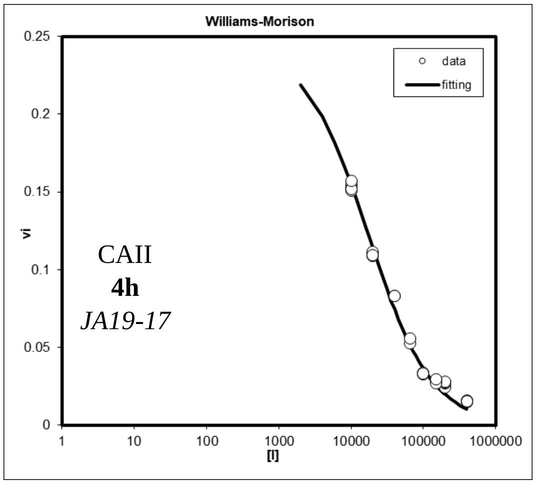
Williams-Morison
data
fitting
vi
[I]
CAII
4h
JA19-17

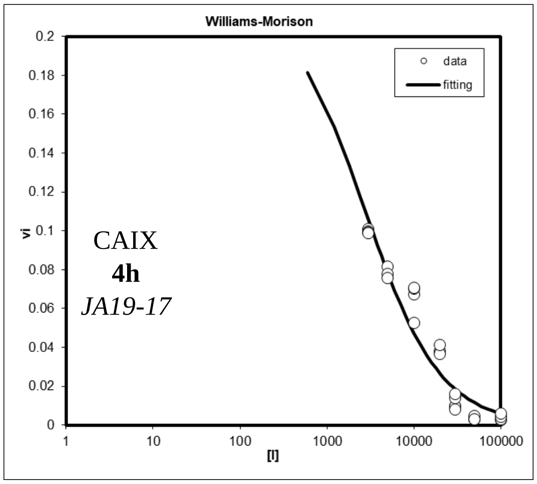
Williams-Morison
data
fitting
vi
[I]
CAIX
4h
JA19-17

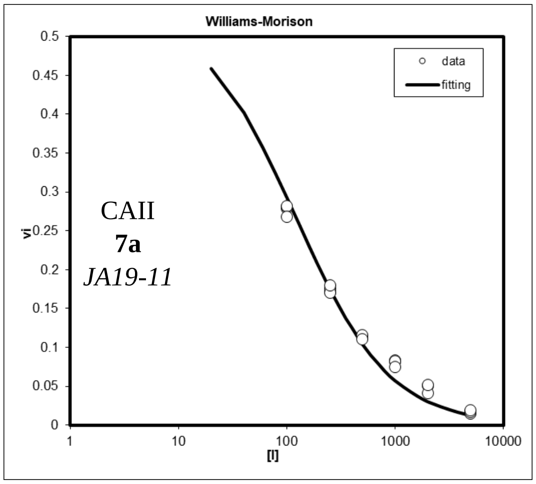
Williams-Morison
data
fitting
vi
[I]
CAII
7a
JA19-11

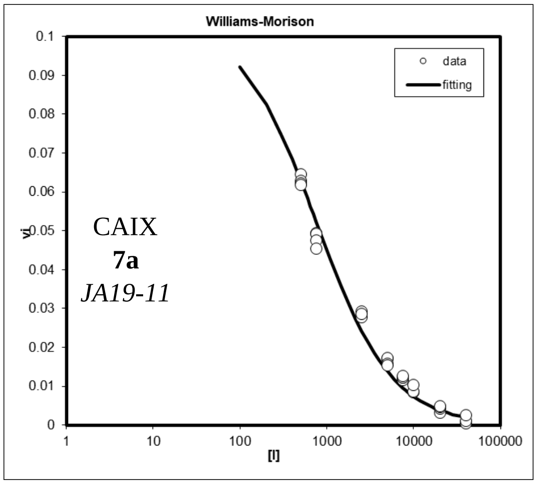
Williams-Morison
data
fitting
vi
[I]
CAIX
7a
JA19-11

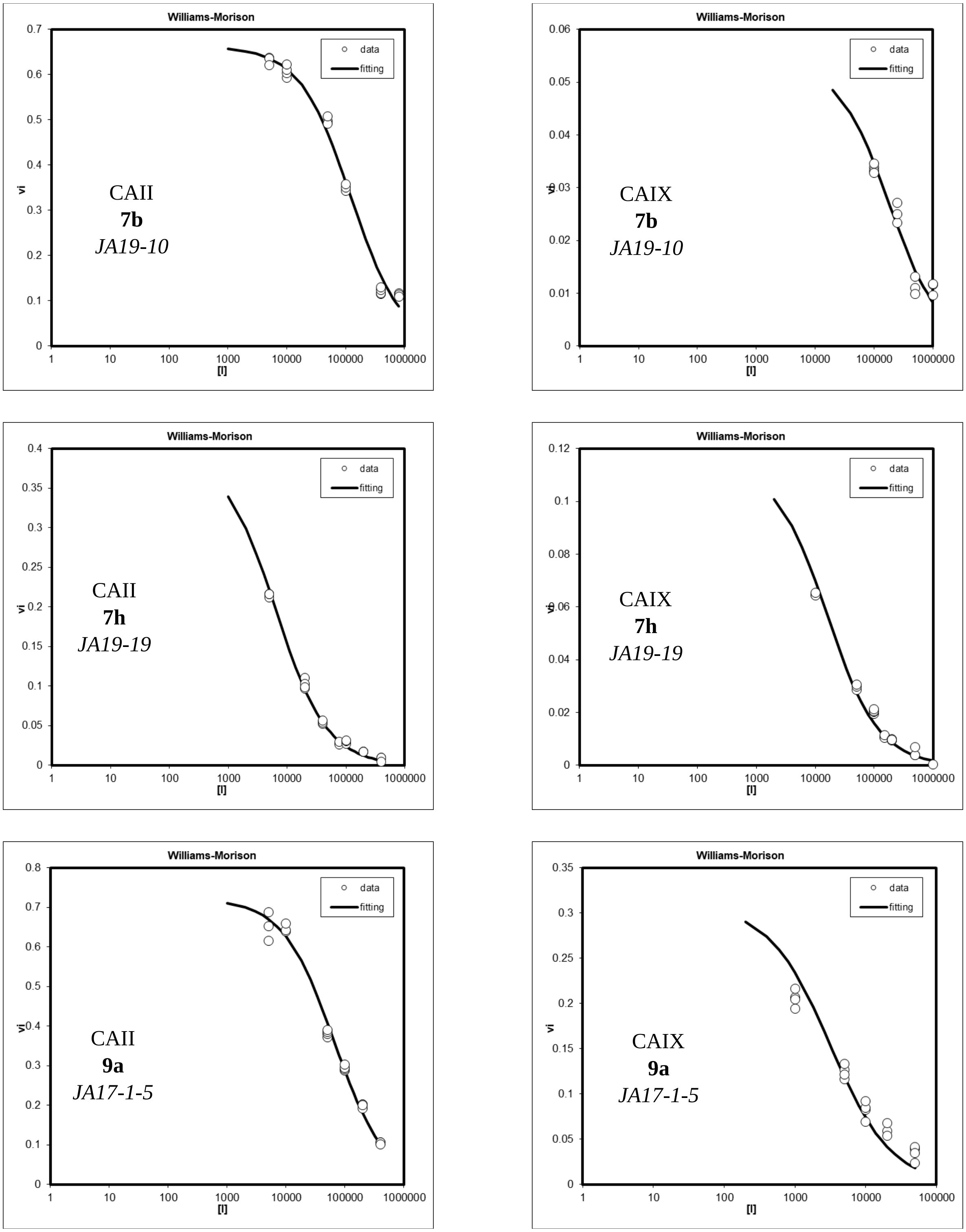

Figure S2. Stopped-flow carbon dioxide hydration assay (SFA) data of compound **4a**, **4b**, **4c**, **4d**, **4h**, **7a**, **7b**, **7h**, and **9a** inhibition of CAII (left column) and CAIX (right column) in 20 mM HEPES at pH 7.5 containing 0.2 mM phenol red and 20 mM Na_2SO_4, at 25 °C. Inhibition constants and IC_{50} are listed in **Table 2**.

Determination of pK_a values of compound –RSO_2NH_2

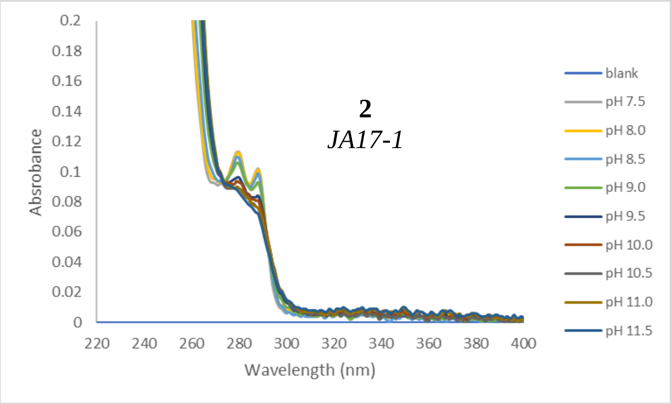

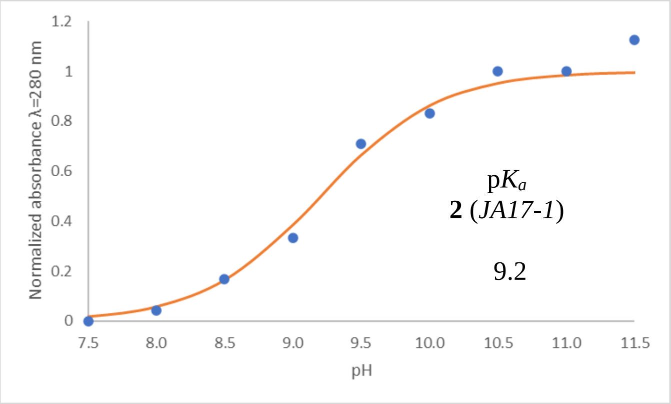

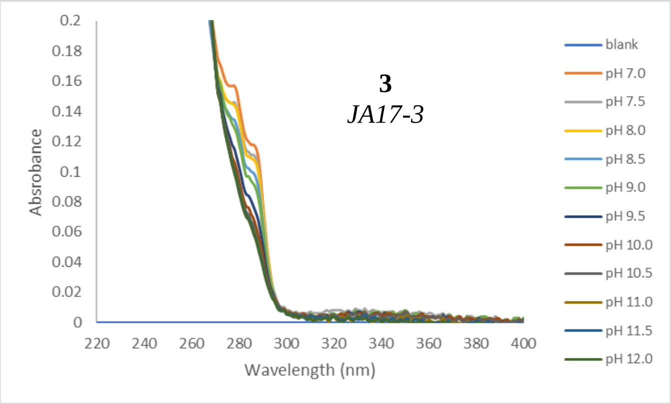

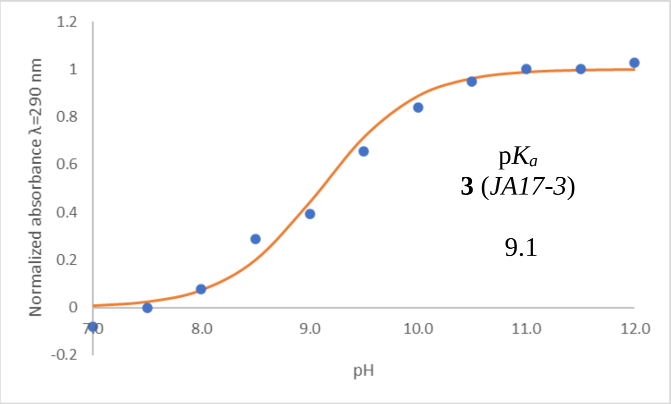

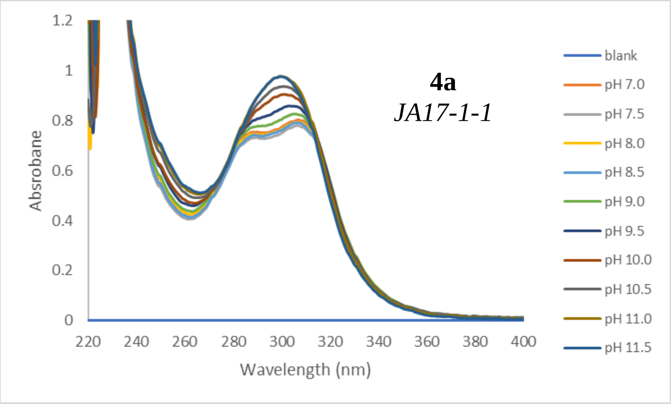

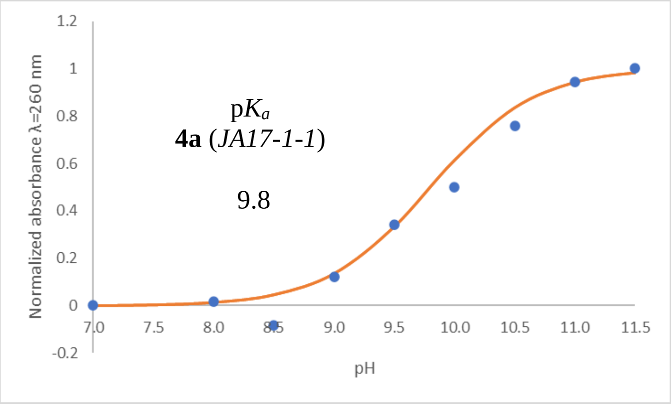

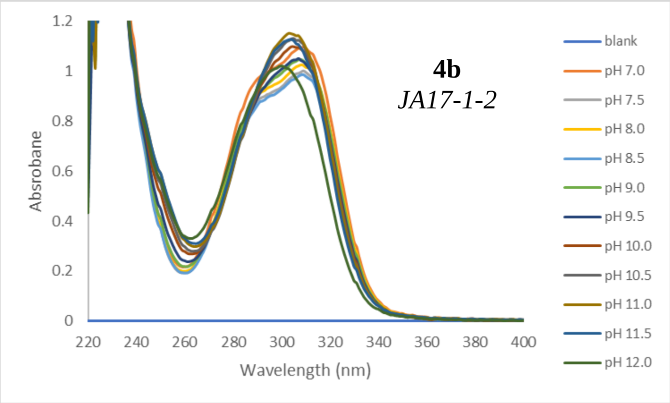
4b
JA17-1-2
Absrobane
Wavelength (nm)
blank
pH 7.0
pH 7.5
pH 8.0
pH 8.5
pH 9.0
pH 9.5
pH 10.0
pH 10.5
pH 11.0
pH 11.5
pH 12.0

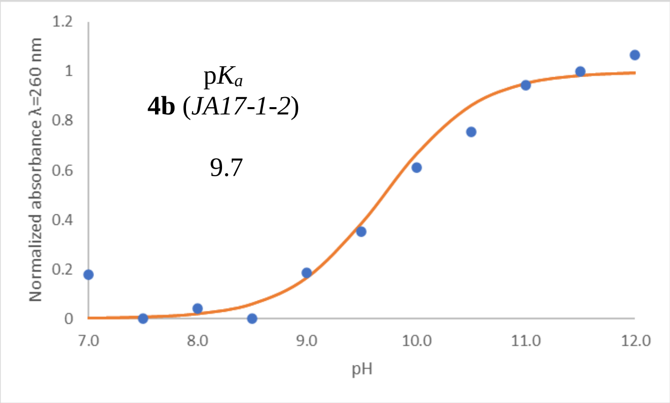
pK_a
4b (JA17-1-2)
9.7
Normalized absorbance λ=260 nm
pH

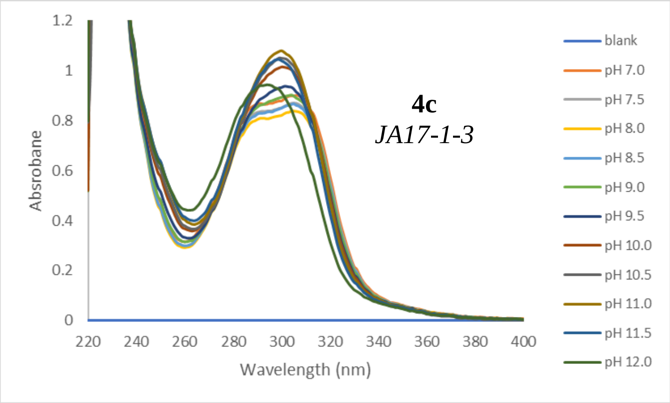
4c
JA17-1-3
Absrobane
Wavelength (nm)
blank
pH 7.0
pH 7.5
pH 8.0
pH 8.5
pH 9.0
pH 9.5
pH 10.0
pH 10.5
pH 11.0
pH 11.5
pH 12.0

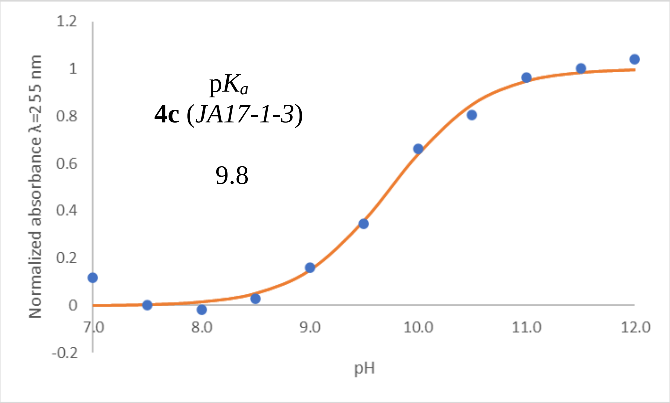
pK_a
4c (JA17-1-3)
9.8
Normalized absorbance λ=255 nm
pH

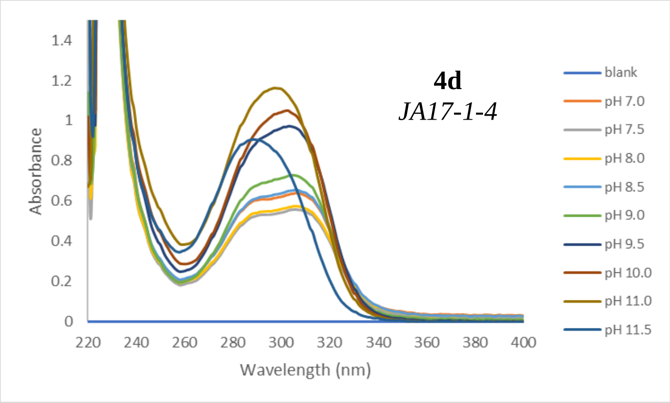
4d
JA17-1-4
Absorbance
Wavelength (nm)
blank
pH 7.0
pH 7.5
pH 8.0
pH 8.5
pH 9.0
pH 9.5
pH 10.0
pH 11.0
pH 11.5

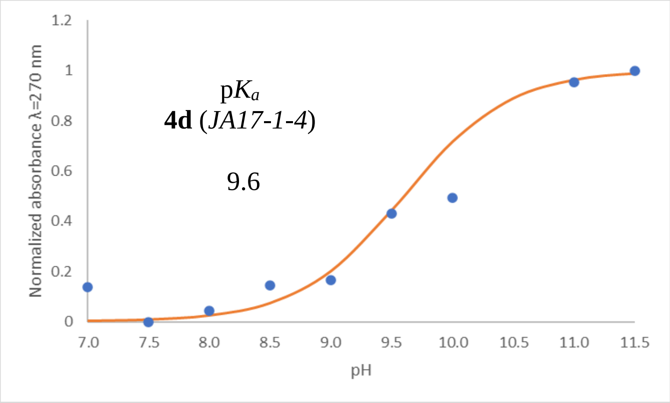
pK_a
4d (JA17-1-4)
9.6
Normalized absorbance λ=270 nm
pH

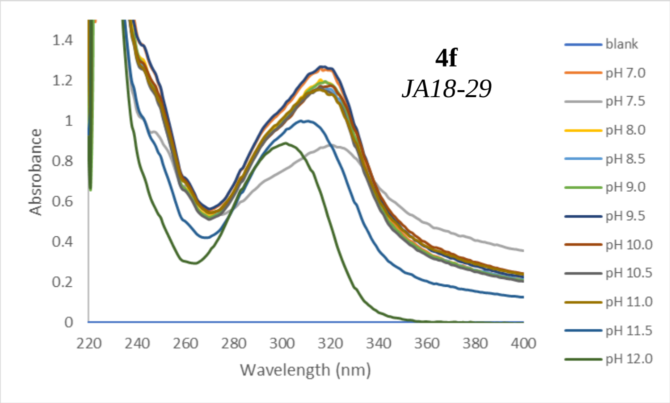
4f
JA18-29
Absrobance
Wavelength (nm)
blank
pH 7.0
pH 7.5
pH 8.0
pH 8.5
pH 9.0
pH 9.5
pH 10.0
pH 10.5
pH 11.0
pH 11.5
pH 12.0

NA

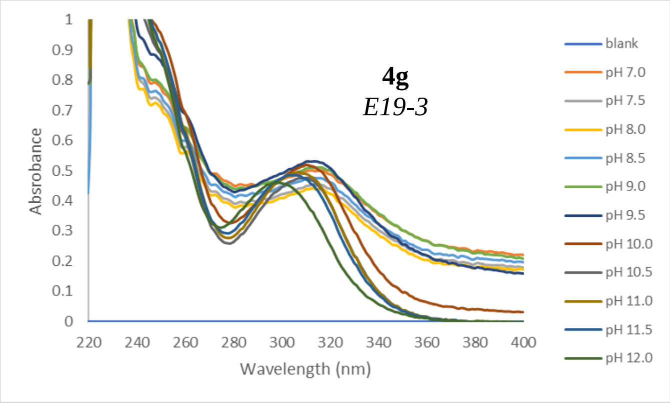
4g
E19-3
Absrobance
Wavelength (nm)
blank
pH 7.0
pH 7.5
pH 8.0
pH 8.5
pH 9.0
pH 9.5
pH 10.0
pH 10.5
pH 11.0
pH 11.5
pH 12.0

NA

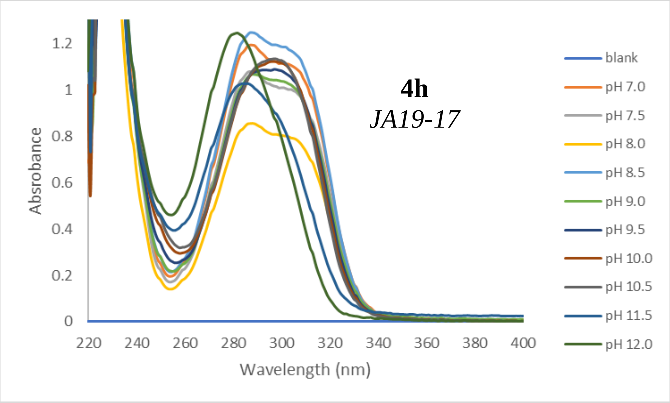
4h
JA19-17
Absrobance
Wavelength (nm)
blank
pH 7.0
pH 7.5
pH 8.0
pH 8.5
pH 9.0
pH 9.5
pH 10.0
pH 10.5
pH 11.0
pH 11.5
pH 12.0

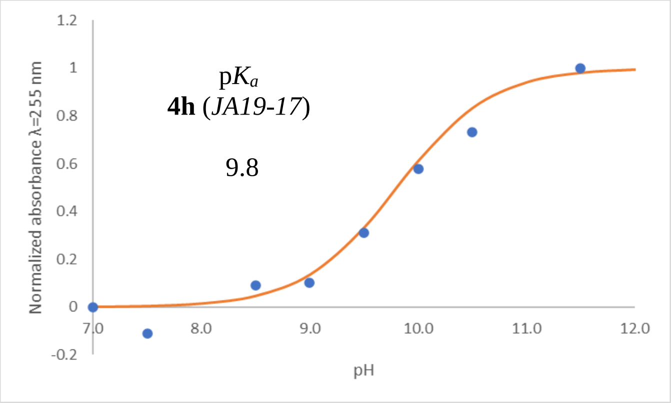
pKa
4h (JA19-17)
9.8
Normalized absorbance λ=255 nm
pH

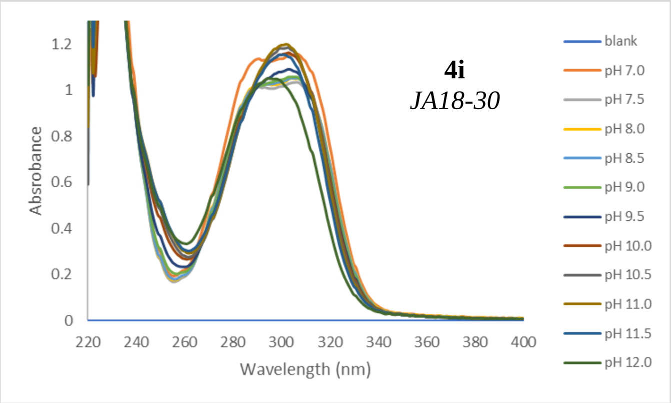
4i
JA18-30
Absorbance
Wavelength (nm)
blank
pH 7.0
pH 7.5
pH 8.0
pH 8.5
pH 9.0
pH 9.5
pH 10.0
pH 10.5
pH 11.0
pH 11.5
pH 12.0

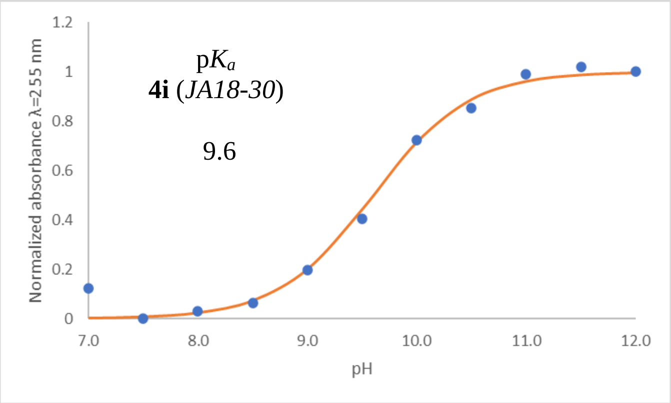
pK_a
4i (JA18-30)
9.6
Normalized absorbance λ=255 nm
pH

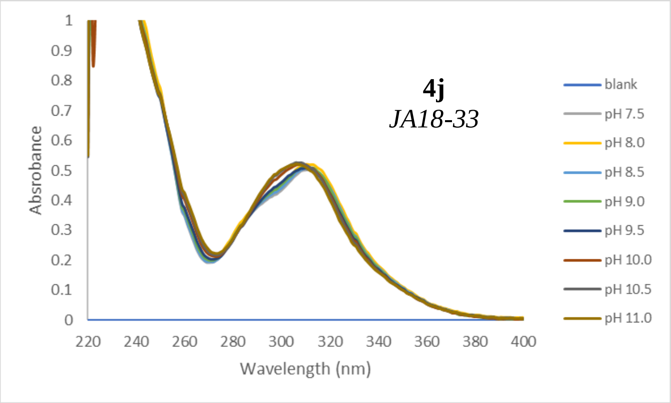
4j
JA18-33
Absrobance
Wavelength (nm)
blank
pH 7.5
pH 8.0
pH 8.5
pH 9.0
pH 9.5
pH 10.0
pH 10.5
pH 11.0

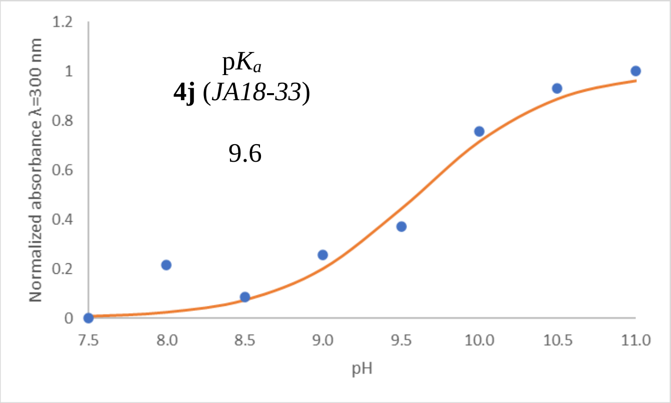
pK_a
4j (JA18-33)
9.6
Normalized absorbance λ=300 nm
pH

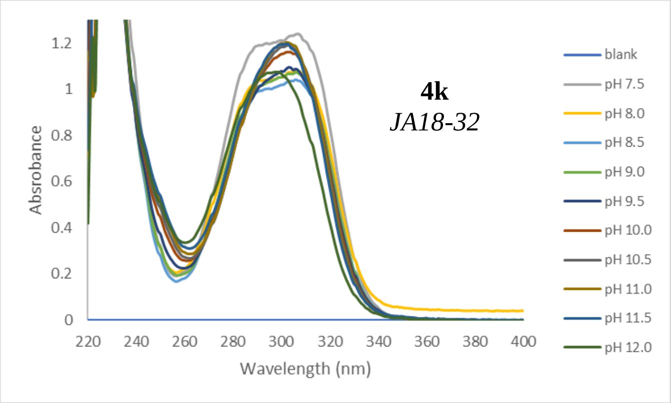
4k
JA18-32
Absrobance
Wavelength (nm)
blank
pH 7.5
pH 8.0
pH 8.5
pH 9.0
pH 9.5
pH 10.0
pH 10.5
pH 11.0
pH 11.5
pH 12.0

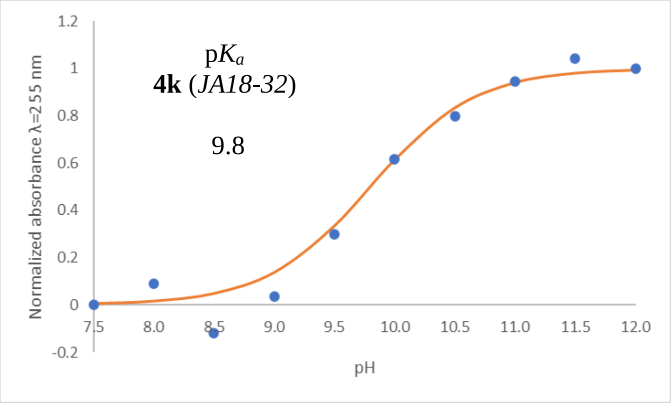
pK_a
4k (JA18-32)
9.8
Normalized absorbance λ=255 nm
pH

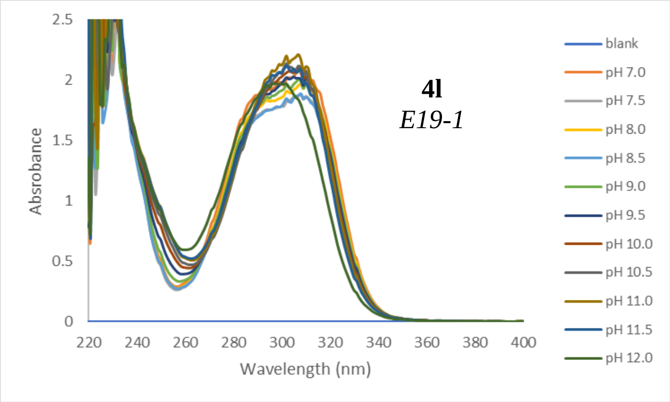
4l
E19-1
Absrobance
Wavelength (nm)
2.5
2
1.5
1
0.5
0
220
240
260
280
300
320
340
360
380
400
blank
pH 7.0
pH 7.5
pH 8.0
pH 8.5
pH 9.0
pH 9.5
pH 10.0
pH 10.5
pH 11.0
pH 11.5
pH 12.0

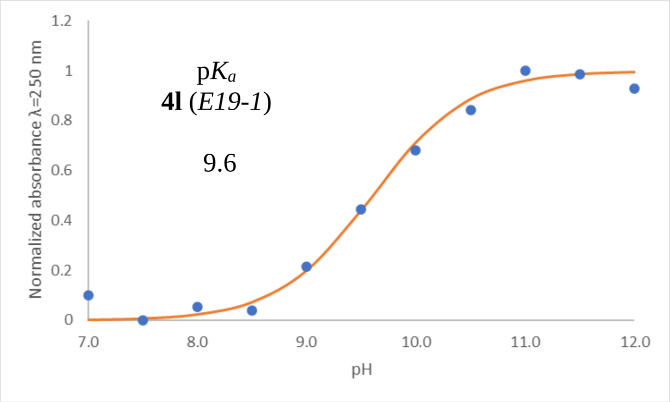
pK_a
4l (E19-1)
9.6
Normalized absorbance λ=250 nm
pH
1.2
1
0.8
0.6
0.4
0.2
0
7.0
8.0
9.0
10.0
11.0
12.0

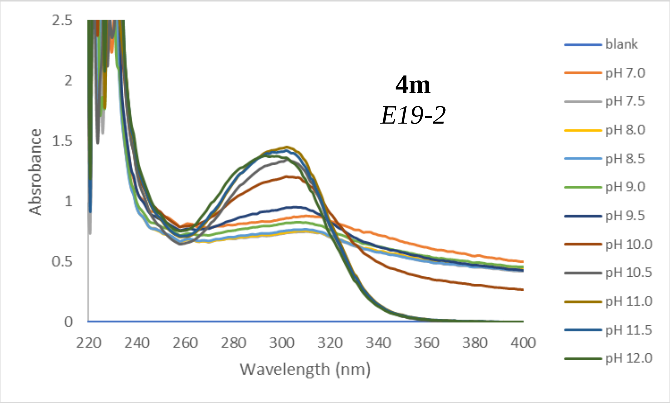
4m
E19-2
Absrobance
Wavelength (nm)
2.5
2
1.5
1
0.5
0
220
240
260
280
300
320
340
360
380
400
blank
pH 7.0
pH 7.5
pH 8.0
pH 8.5
pH 9.0
pH 9.5
pH 10.0
pH 10.5
pH 11.0
pH 11.5
pH 12.0

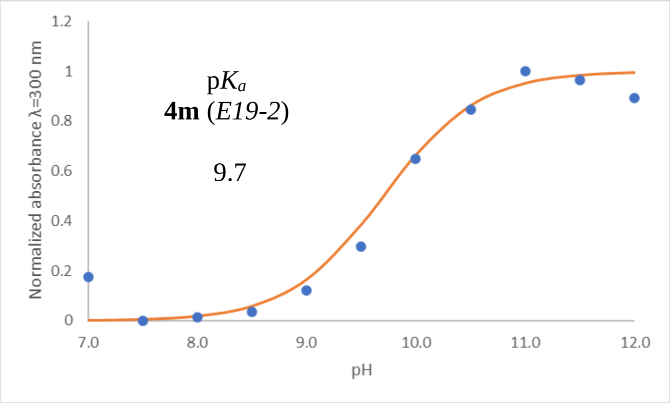
pK_a
4m (E19-2)
9.7
Normalized absorbance λ=300 nm
pH
1.2
1
0.8
0.6
0.4
0.2
0
7.0
8.0
9.0
10.0
11.0
12.0

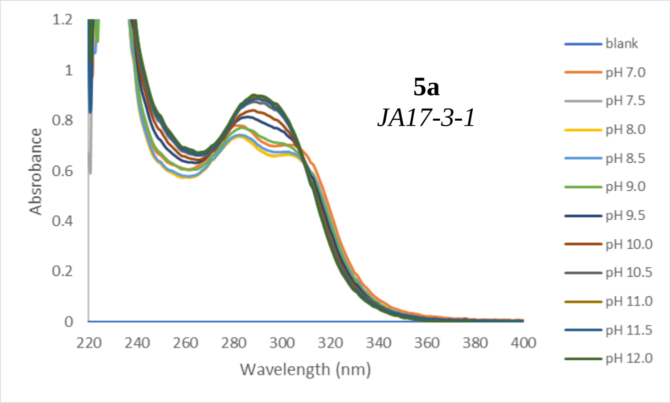
5a
JA17-3-1
Absrobance
Wavelength (nm)
1.2
1
0.8
0.6
0.4
0.2
0
220
240
260
280
300
320
340
360
380
400
blank
pH 7.0
pH 7.5
pH 8.0
pH 8.5
pH 9.0
pH 9.5
pH 10.0
pH 10.5
pH 11.0
pH 11.5
pH 12.0

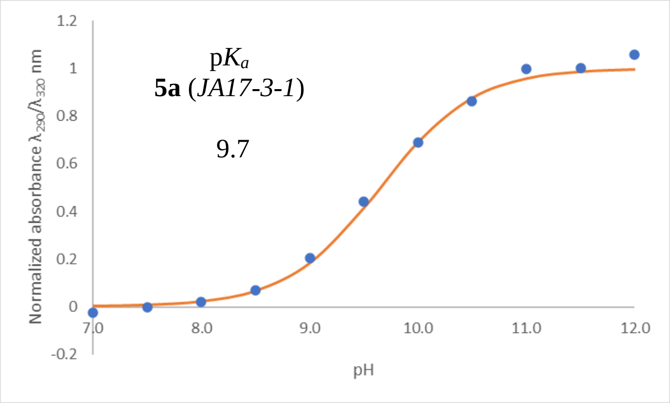
pK_a
5a (JA17-3-1)
9.7
Normalized absorbance $\lambda_{290}/\lambda_{320}$ nm
pH
1.2
1
0.8
0.6
0.4
0.2
0
-0.2
7.0
8.0
9.0
10.0
11.0
12.0

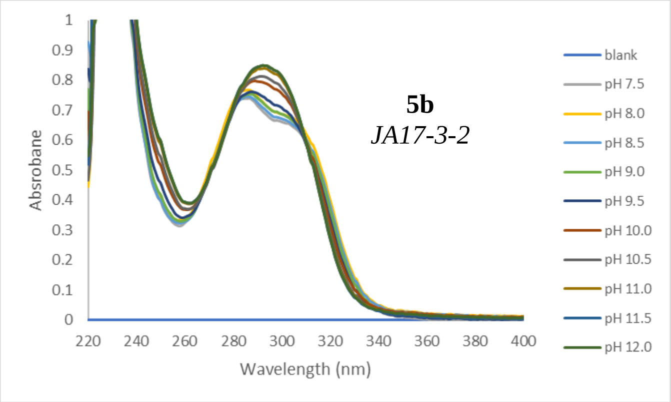
5b
JA17-3-2
Absrobane
Wavelength (nm)
blank
pH 7.5
pH 8.0
pH 8.5
pH 9.0
pH 9.5
pH 10.0
pH 10.5
pH 11.0
pH 11.5
pH 12.0

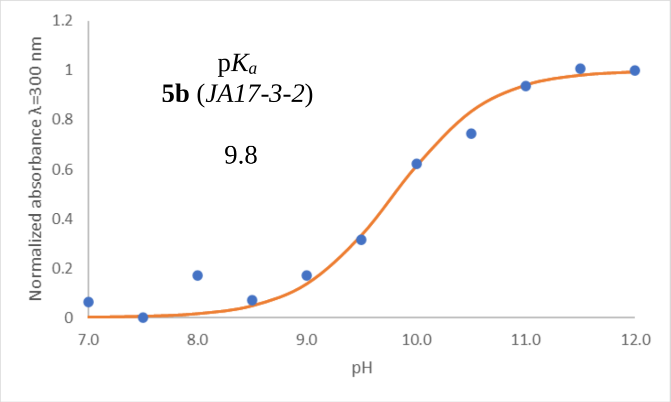
pK_a
5b (*JA17-3-2*)
9.8
Normalized absorbance λ=300 nm
pH

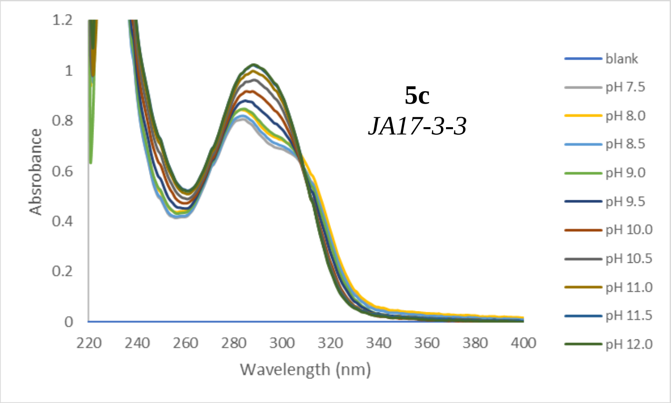
5c
JA17-3-3
Absorbance
Wavelength (nm)
blank
pH 7.5
pH 8.0
pH 8.5
pH 9.0
pH 9.5
pH 10.0
pH 10.5
pH 11.0
pH 11.5
pH 12.0

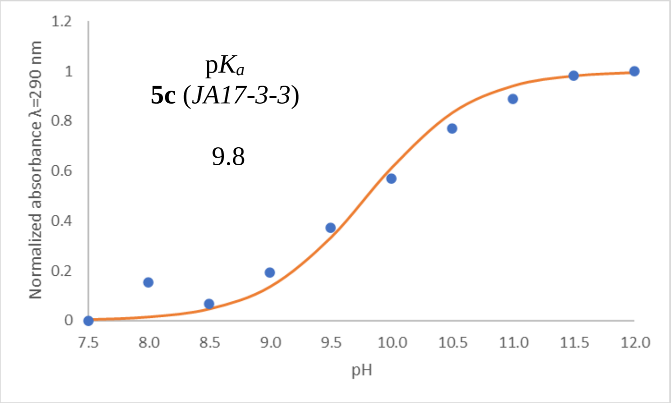
pK_a
5c (*JA17-3-3*)
9.8
Normalized absorbance λ=290 nm
pH

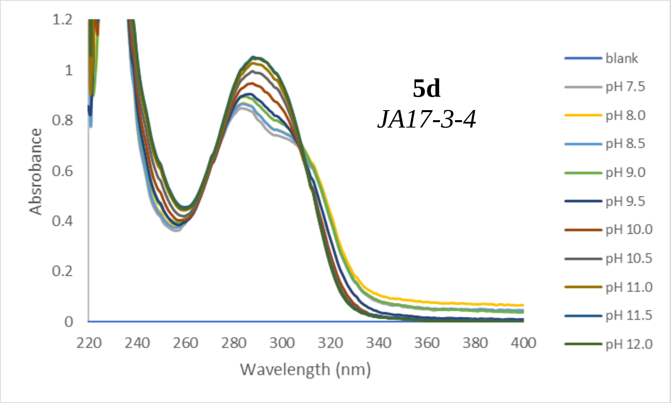
5d
JA17-3-4
Absorbance
Wavelength (nm)
blank
pH 7.5
pH 8.0
pH 8.5
pH 9.0
pH 9.5
pH 10.0
pH 10.5
pH 11.0
pH 11.5
pH 12.0

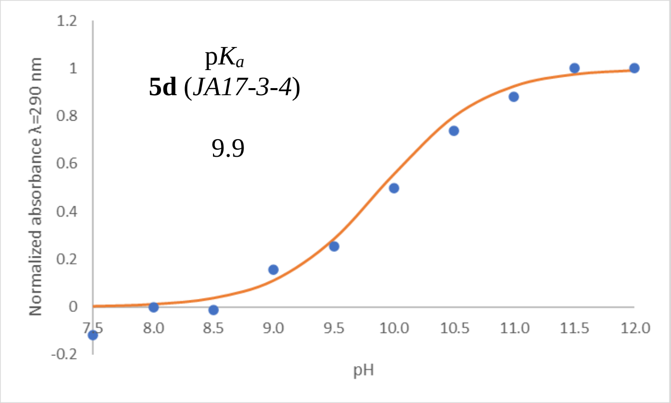
pK_a
5d (*JA17-3-4*)
9.9
Normalized absorbance λ=290 nm
pH

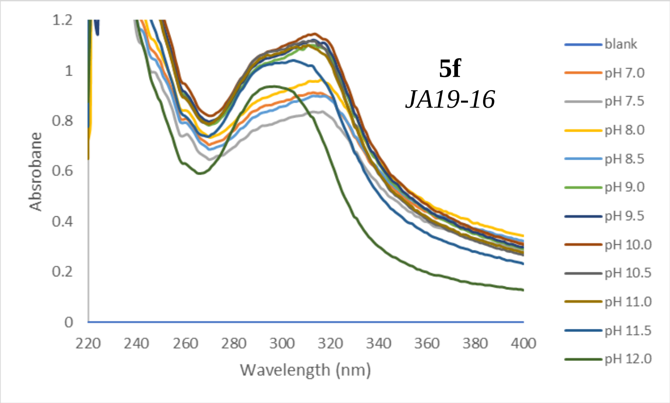
5f
JA19-16
Absrobane
Wavelength (nm)
blank
pH 7.0
pH 7.5
pH 8.0
pH 8.5
pH 9.0
pH 9.5
pH 10.0
pH 10.5
pH 11.0
pH 11.5
pH 12.0

NA

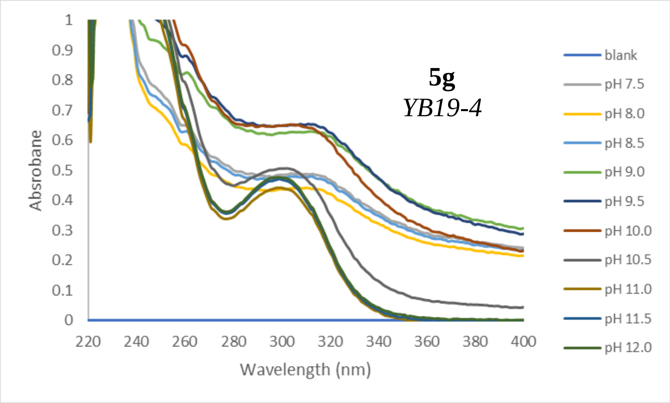
5g
YB19-4
Absrobane
Wavelength (nm)
blank
pH 7.5
pH 8.0
pH 8.5
pH 9.0
pH 9.5
pH 10.0
pH 10.5
pH 11.0
pH 11.5
pH 12.0

NA

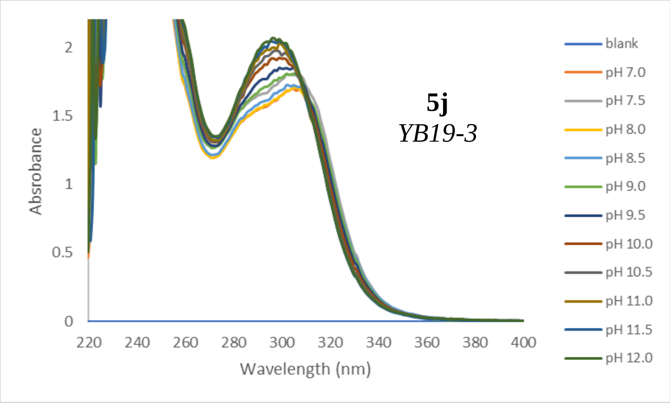
5j
YB19-3
Absrobance
Wavelength (nm)
blank
pH 7.0
pH 7.5
pH 8.0
pH 8.5
pH 9.0
pH 9.5
pH 10.0
pH 10.5
pH 11.0
pH 11.5
pH 12.0

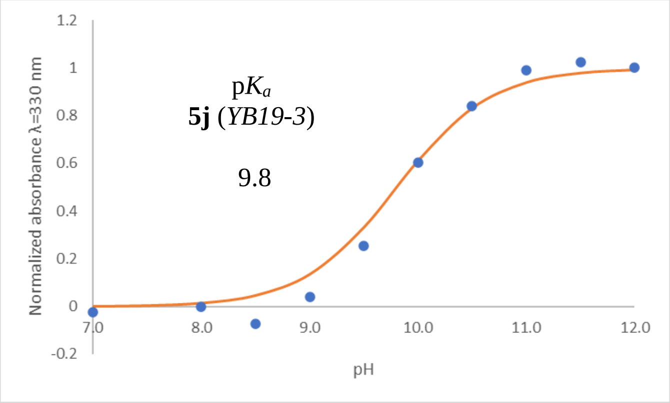
pKa
5j (YB19-3)
9.8
Normalized absorbance λ=330 nm
pH

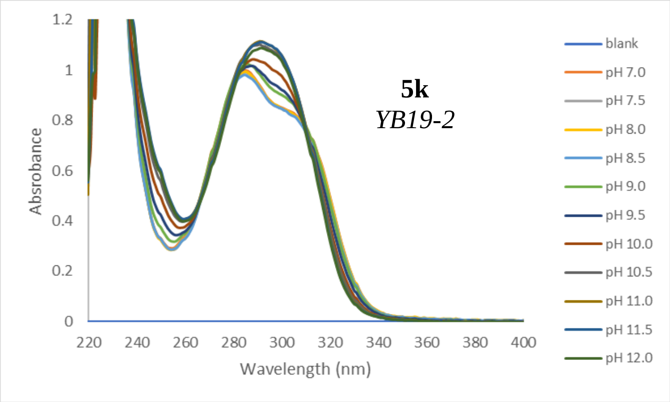
5k
YB19-2
Absrobance
Wavelength (nm)
blank
pH 7.0
pH 7.5
pH 8.0
pH 8.5
pH 9.0
pH 9.5
pH 10.0
pH 10.5
pH 11.0
pH 11.5
pH 12.0

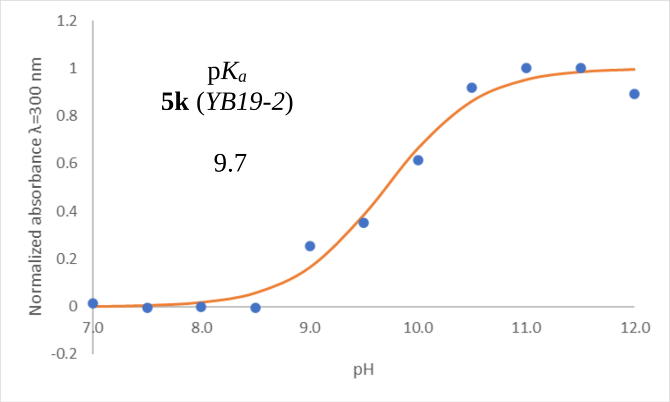
pK_a
5k (YB19-2)
9.7
Normalized absorbance λ=300 nm
pH

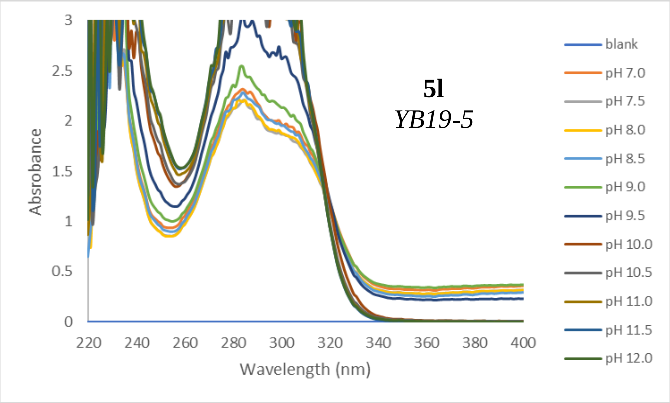
5l
YB19-5
Absrobane
Wavelength (nm)
blank
pH 7.0
pH 7.5
pH 8.0
pH 8.5
pH 9.0
pH 9.5
pH 10.0
pH 10.5
pH 11.0
pH 11.5
pH 12.0

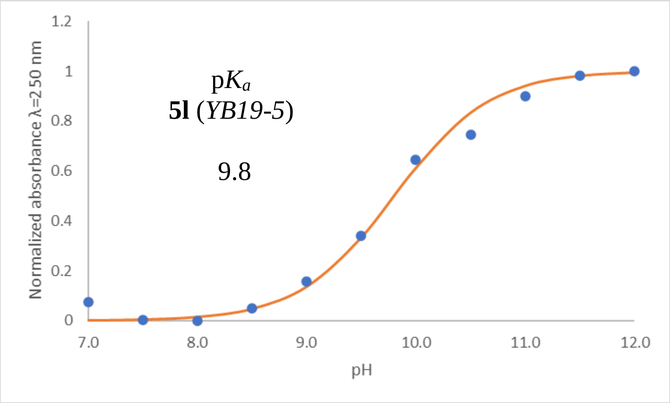
pK_a
5l (YB19-5)
9.8
Normalized absorbance λ=250 nm
pH

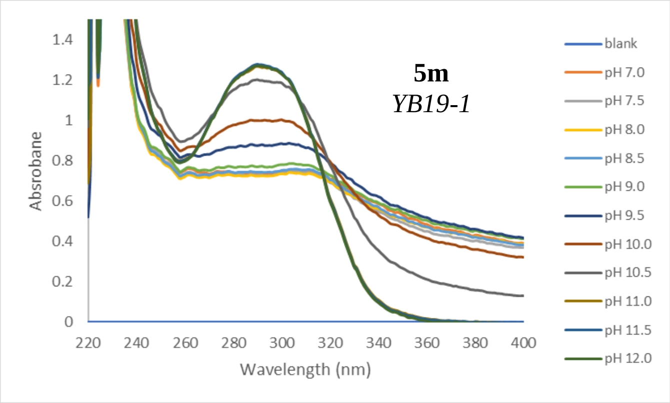
5m
YB19-1
Absrobane
Wavelength (nm)
blank
pH 7.0
pH 7.5
pH 8.0
pH 8.5
pH 9.0
pH 9.5
pH 10.0
pH 10.5
pH 11.0
pH 11.5
pH 12.0

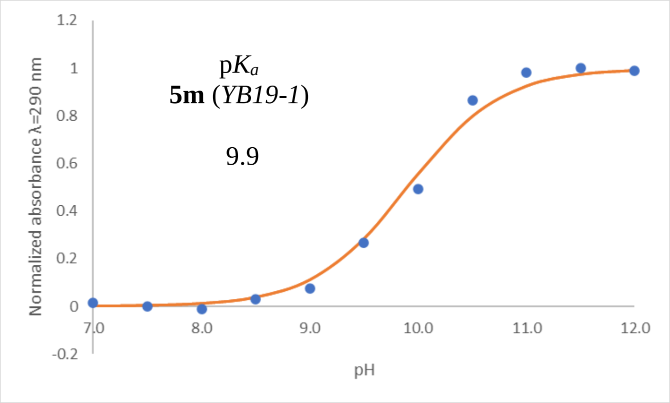
pK_a
5m (YB19-1)
9.9
Normalized absorbance λ=290 nm
pH

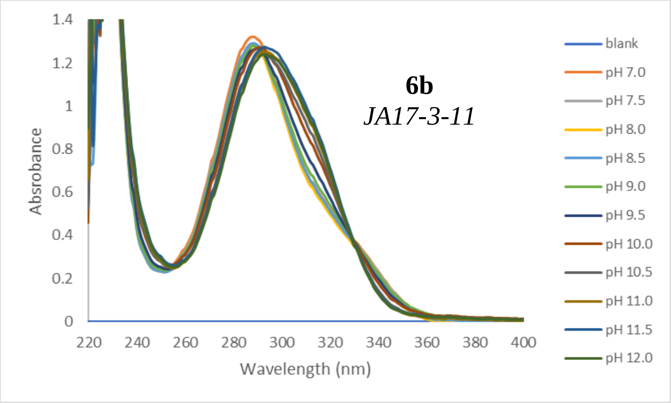
6b
JA17-3-11
Absrobane
Wavelength (nm)
blank
pH 7.0
pH 7.5
pH 8.0
pH 8.5
pH 9.0
pH 9.5
pH 10.0
pH 10.5
pH 11.0
pH 11.5
pH 12.0

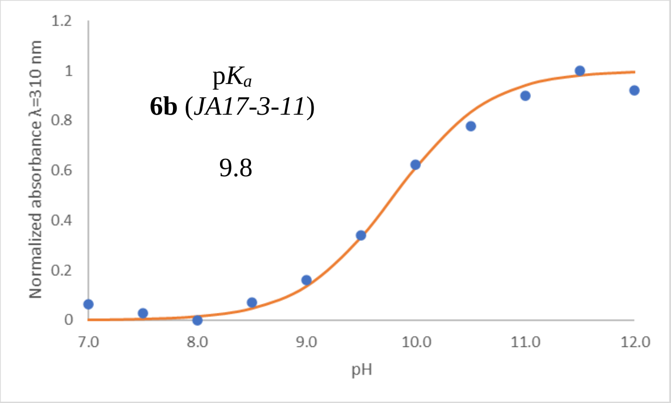
pK_a
6b (JA17-3-11)
9.8
Normalized absorbance λ=310 nm
pH

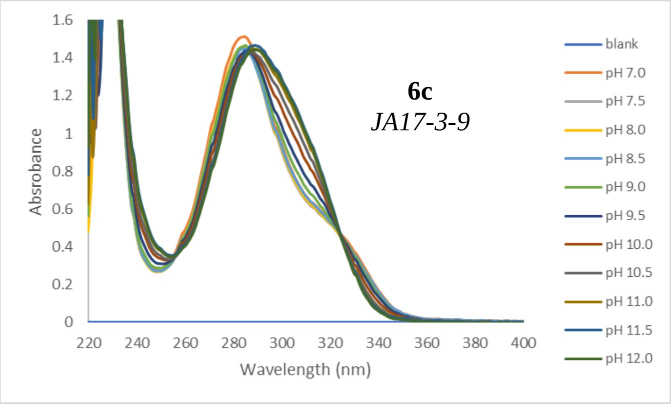
6c
JA17-3-9
Absrobance
Wavelength (nm)
blank
pH 7.0
pH 7.5
pH 8.0
pH 8.5
pH 9.0
pH 9.5
pH 10.0
pH 10.5
pH 11.0
pH 11.5
pH 12.0

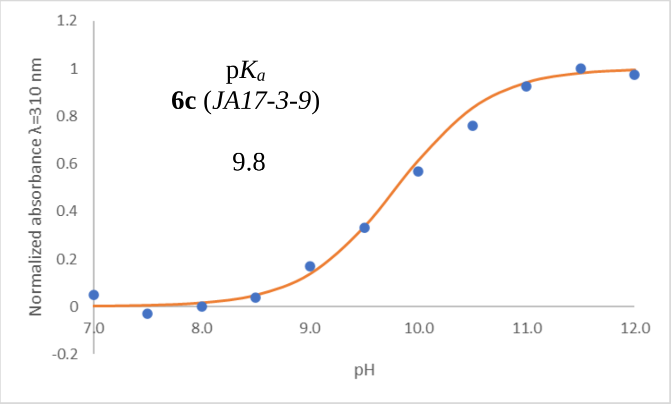
pK_a
6c (JA17-3-9)
9.8
Normalized absorbance λ=310 nm
pH

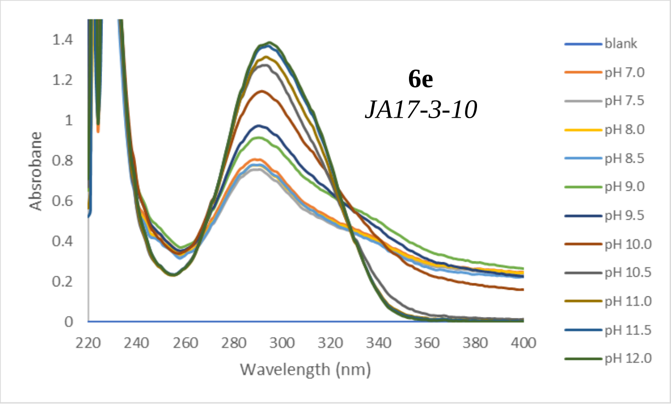
6e
JA17-3-10
Absrobane
Wavelength (nm)
blank
pH 7.0
pH 7.5
pH 8.0
pH 8.5
pH 9.0
pH 9.5
pH 10.0
pH 10.5
pH 11.0
pH 11.5
pH 12.0

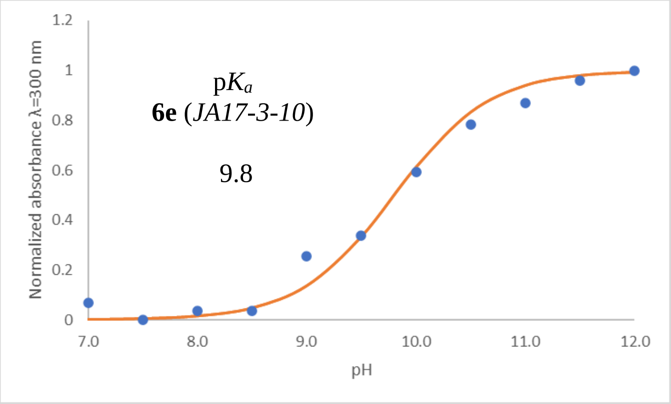
pK_a
6e (JA17-3-10)
9.8
Normalized absorbance λ=300 nm
pH

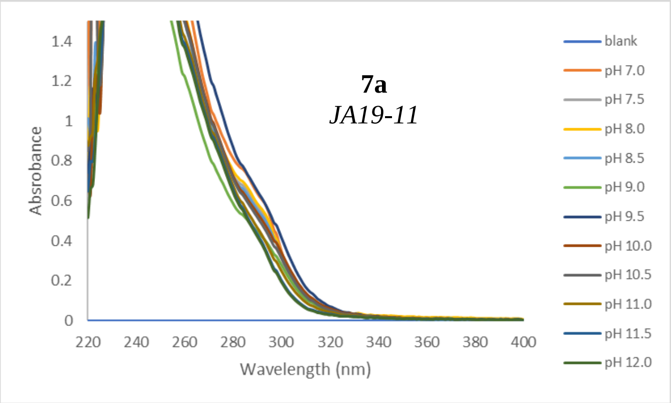

1.4
1.2
1
0.8
0.6
0.4
0.2
0
Absrobance
220 240 260 280 300 320 340 360 380 400
Wavelength (nm)
7a
JA19-11
blank
pH 7.0
pH 7.5
pH 8.0
pH 8.5
pH 9.0
pH 9.5
pH 10.0
pH 10.5
pH 11.0
pH 11.5
pH 12.0

NA

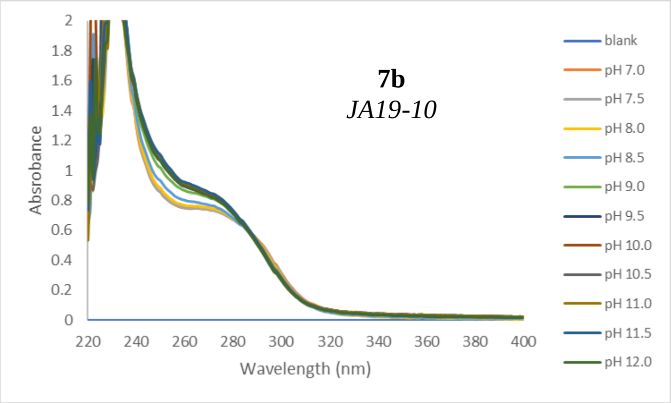

2
1.8
1.6
1.4
1.2
1
0.8
0.6
0.4
0.2
0
Absrobance
220 240 260 280 300 320 340 360 380 400
Wavelength (nm)
7b
JA19-10
blank
pH 7.0
pH 7.5
pH 8.0
pH 8.5
pH 9.0
pH 9.5
pH 10.0
pH 10.5
pH 11.0
pH 11.5
pH 12.0

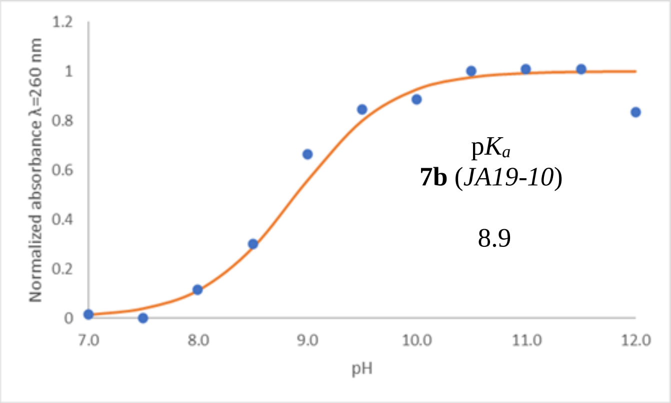

1.2
1
0.8
0.6
0.4
0.2
0
Normalized absorbance λ=260 nm
7.0 8.0 9.0 10.0 11.0 12.0
pH
p*K*a
7b (*JA19-10*)
8.9

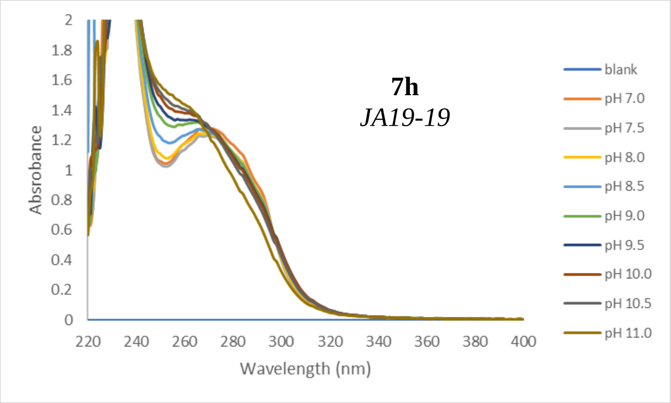

2
1.8
1.6
1.4
1.2
1
0.8
0.6
0.4
0.2
0
Absrobance
220 240 260 280 300 320 340 360 380 400
Wavelength (nm)
7h
JA19-19
blank
pH 7.0
pH 7.5
pH 8.0
pH 8.5
pH 9.0
pH 9.5
pH 10.0
pH 10.5
pH 11.0

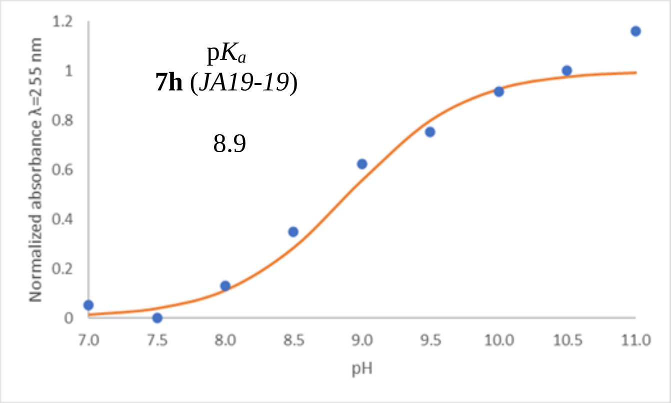

1.2
1
0.8
0.6
0.4
0.2
0
Normalized absorbance λ=255 nm
7.0 7.5 8.0 8.5 9.0 9.5 10.0 10.5 11.0
pH
p*K*a
7h (*JA19-19*)
8.9

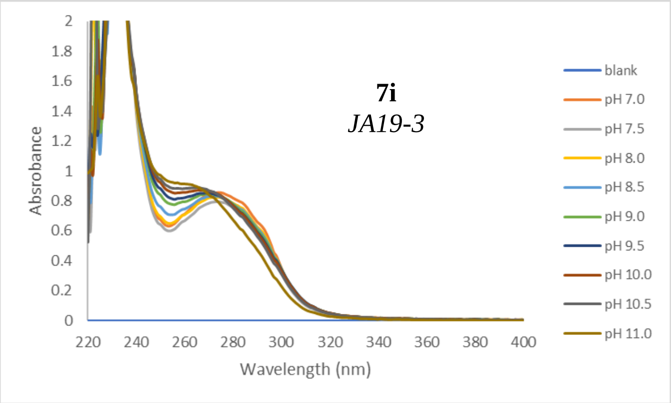
7i
JA19-3
Absrobance
Wavelength (nm)
blank
pH 7.0
pH 7.5
pH 8.0
pH 8.5
pH 9.0
pH 9.5
pH 10.0
pH 10.5
pH 11.0

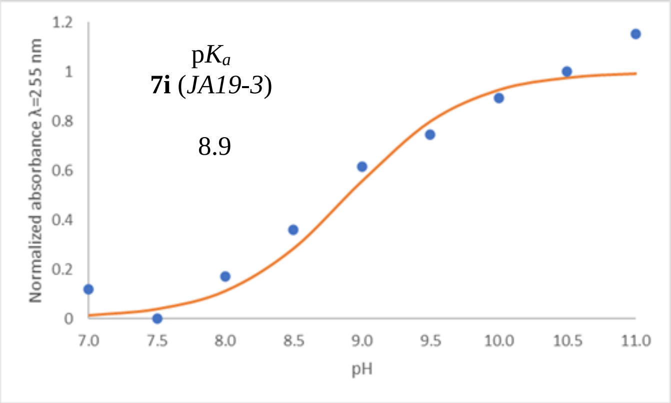
pK_a
7i (JA19-3)
8.9
Normalized absorbance λ=255 nm
pH

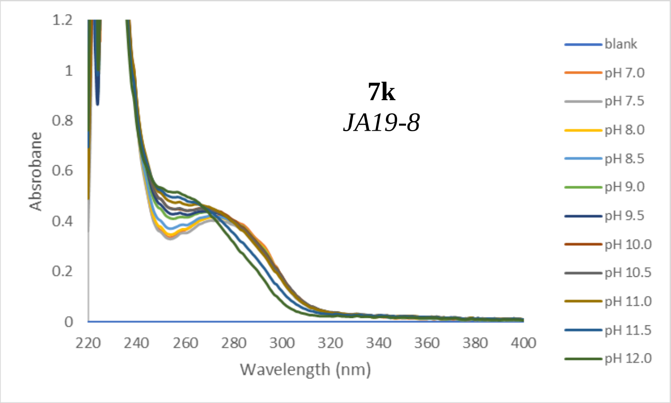
7k
JA19-8
Absrobance
Wavelength (nm)
blank
pH 7.0
pH 7.5
pH 8.0
pH 8.5
pH 9.0
pH 9.5
pH 10.0
pH 10.5
pH 11.0
pH 11.5
pH 12.0

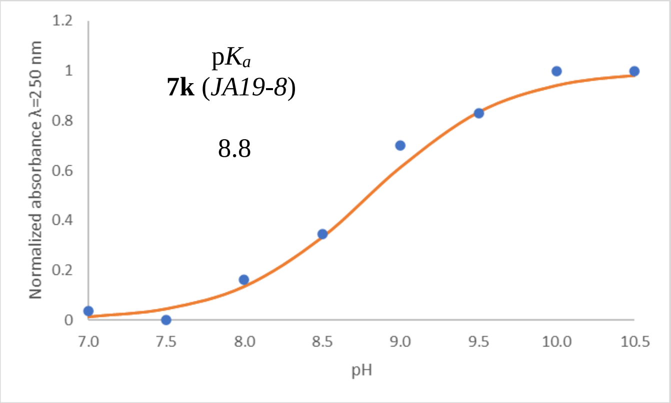
pK_a
7k (JA19-8)
8.8
Normalized absorbance λ=250 nm
pH

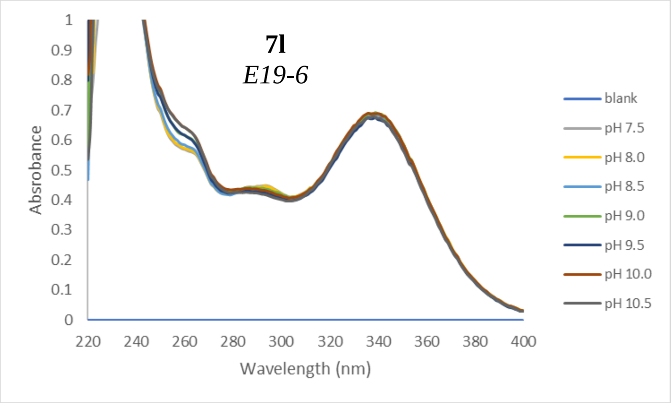
7l
E19-6
Absrobance
Wavelength (nm)
blank
pH 7.5
pH 8.0
pH 8.5
pH 9.0
pH 9.5
pH 10.0
pH 10.5

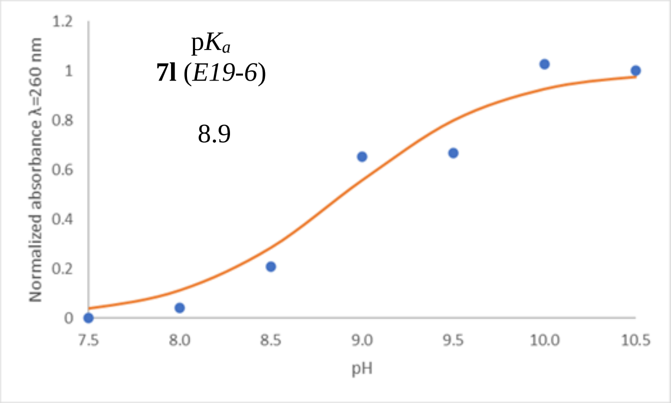
pK_a
7l (E19-6)
8.9
Normalized absorbance λ=260 nm
pH

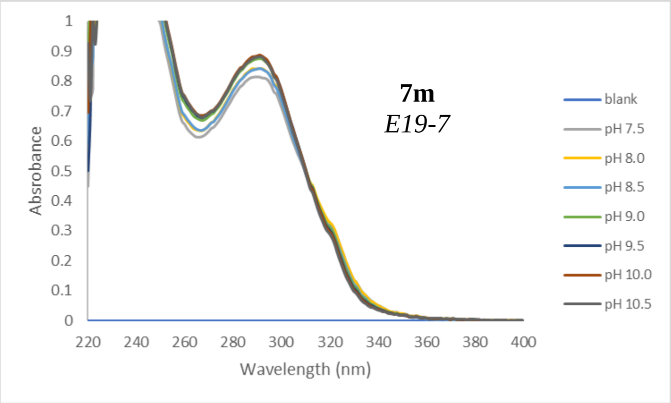
7m
E19-7
Absrobance
Wavelength (nm)
blank
pH 7.5
pH 8.0
pH 8.5
pH 9.0
pH 9.5
pH 10.0
pH 10.5

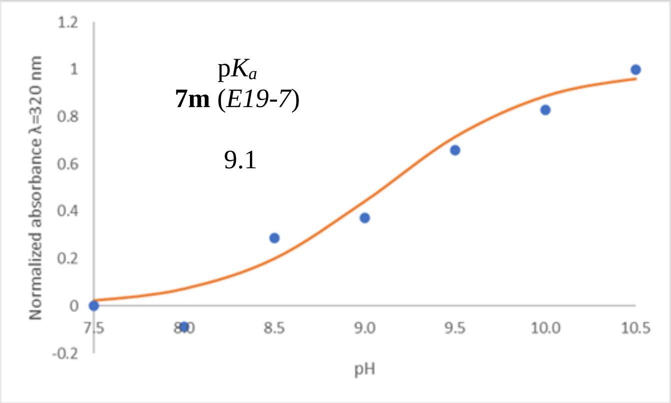
pK_a
7m (E19-7)
9.1
Normalized absorbance λ=320 nm
pH

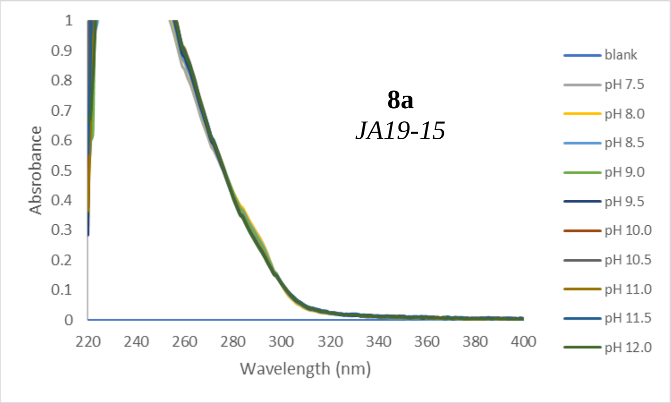
8a
JA19-15
Absrobance
Wavelength (nm)
blank
pH 7.5
pH 8.0
pH 8.5
pH 9.0
pH 9.5
pH 10.0
pH 10.5
pH 11.0
pH 11.5
pH 12.0

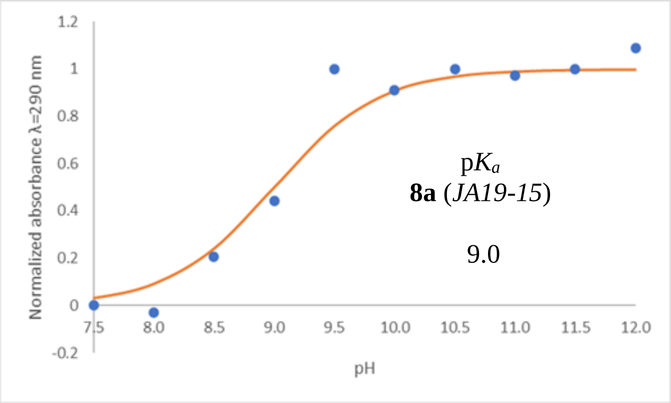
pK_a
8a (JA19-15)
9.0
Normalized absorbance λ=290 nm
pH

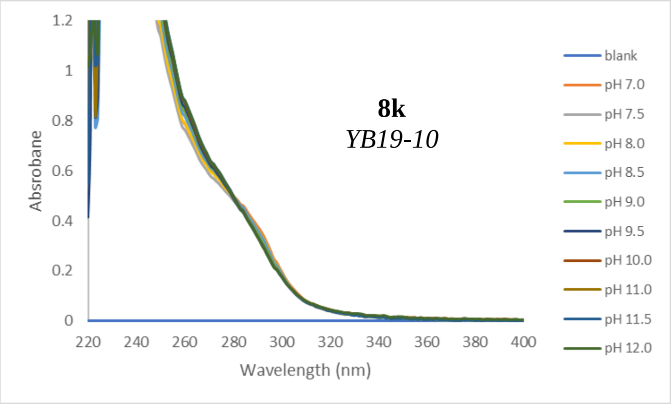
8k
YB19-10
Absrobane
Wavelength (nm)
blank
pH 7.0
pH 7.5
pH 8.0
pH 8.5
pH 9.0
pH 9.5
pH 10.0
pH 11.0
pH 11.5
pH 12.0

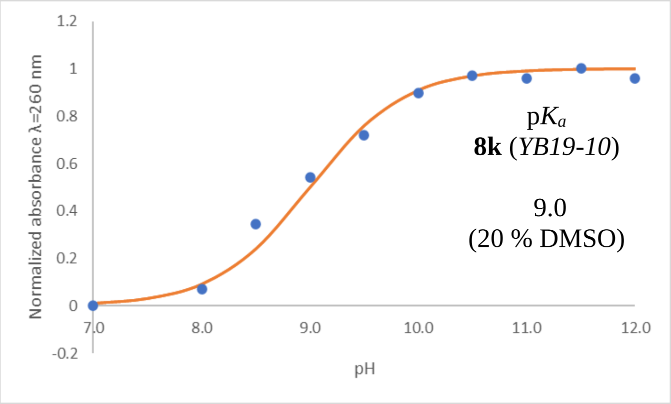
pK_a
8k (YB19-10)
9.0
(20 % DMSO)
Normalized absorbance λ=260 nm
pH

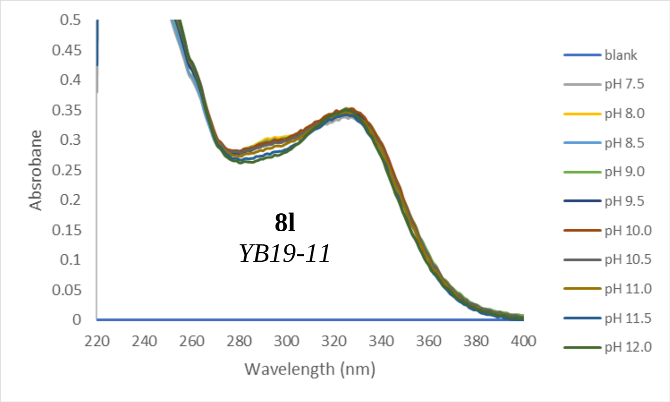

Absrobane
Wavelength (nm)
8l
YB19-11
blank
pH 7.5
pH 8.0
pH 8.5
pH 9.0
pH 9.5
pH 10.0
pH 10.5
pH 11.0
pH 11.5
pH 12.0

NA

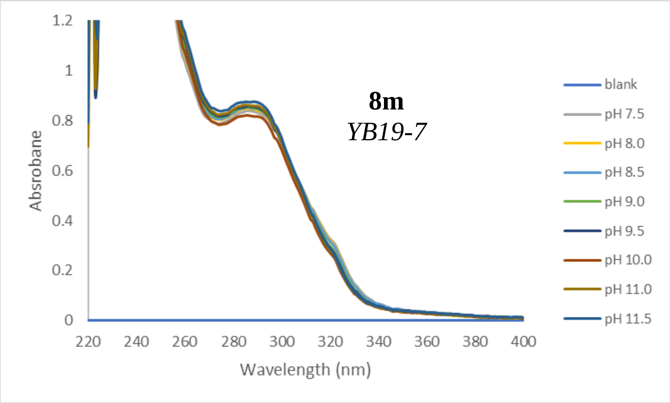

Absrobane
Wavelength (nm)
8m
YB19-7
blank
pH 7.5
pH 8.0
pH 8.5
pH 9.0
pH 9.5
pH 10.0
pH 11.0
pH 11.5

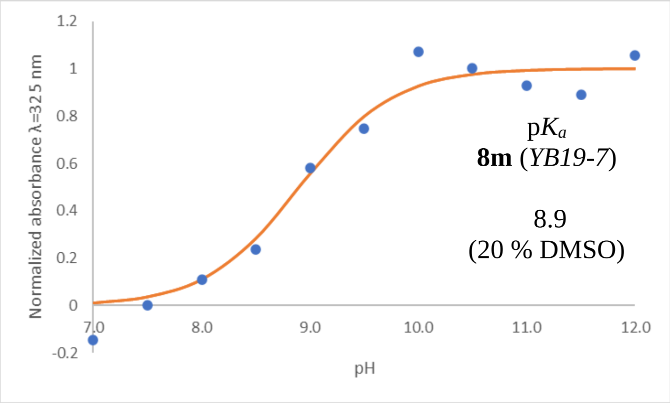

Normalized absorbance λ=325 nm
pH
p*K*a
8m (*YB19-7*)
8.9
(20 % DMSO)

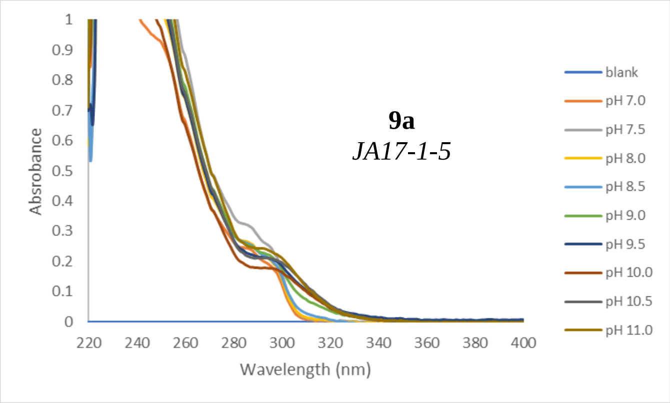

Absrobance
Wavelength (nm)
9a
JA17-1-5
blank
pH 7.0
pH 7.5
pH 8.0
pH 8.5
pH 9.0
pH 9.5
pH 10.0
pH 10.5
pH 11.0

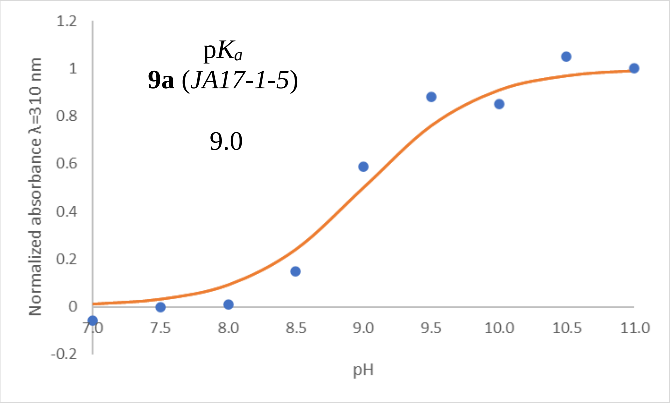

Normalized absorbance λ=310 nm
pH
p*K*a
9a (*JA17-1-5*)
9.0

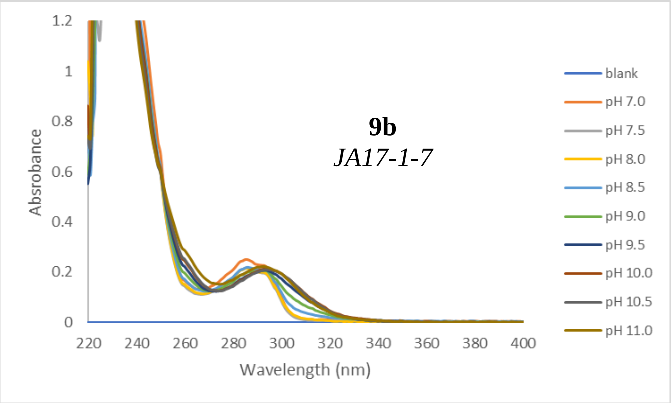
9b
JA17-1-7
Absrobance
Wavelength (nm)
blank
pH 7.0
pH 7.5
pH 8.0
pH 8.5
pH 9.0
pH 9.5
pH 10.0
pH 10.5
pH 11.0

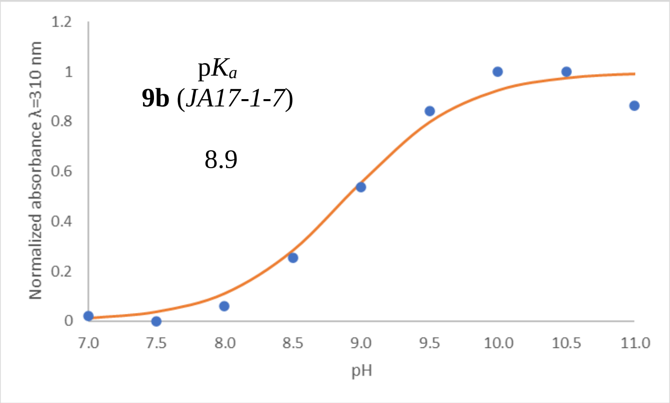
pKa
9b (JA17-1-7)
8.9
Normalized absorbance λ=310 nm
pH

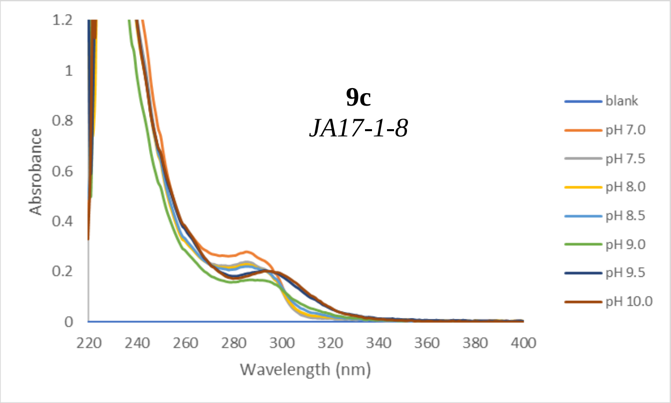
9c
JA17-1-8
Absrobance
Wavelength (nm)
blank
pH 7.0
pH 7.5
pH 8.0
pH 8.5
pH 9.0
pH 9.5
pH 10.0

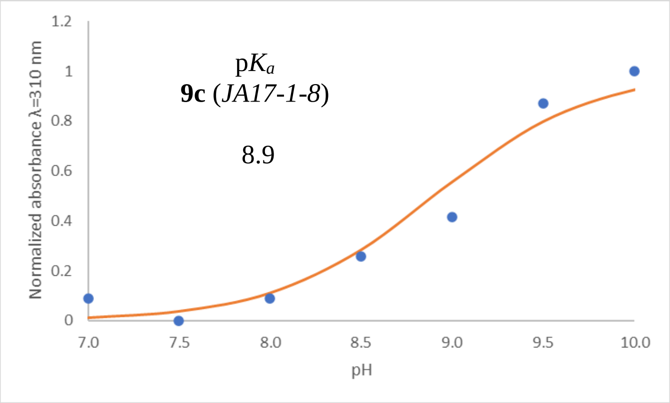
pKa
9c (JA17-1-8)
8.9
Normalized absorbance λ=310 nm
pH

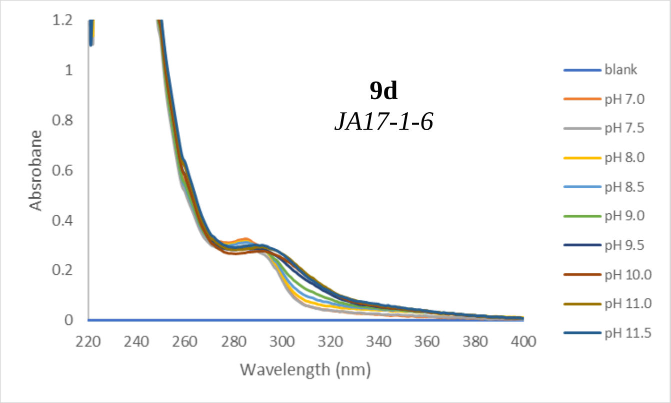
9d
JA17-1-6
Absrobance
Wavelength (nm)
blank
pH 7.0
pH 7.5
pH 8.0
pH 8.5
pH 9.0
pH 9.5
pH 10.0
pH 11.0
pH 11.5

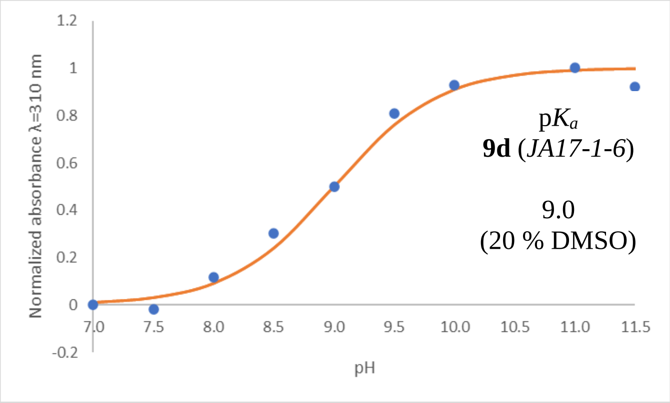
pKa
9d (JA17-1-6)
9.0
(20 % DMSO)
Normalized absorbance λ=310 nm
pH

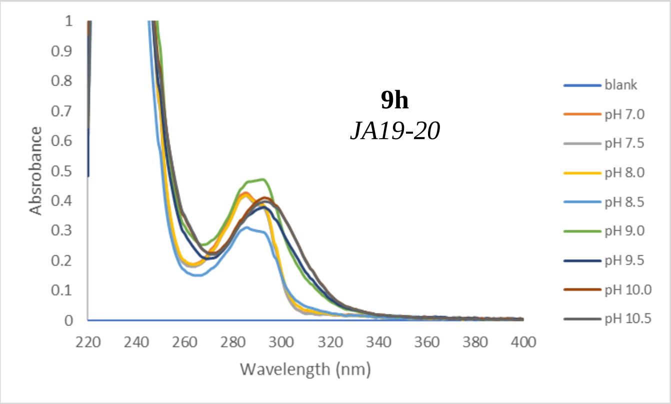
9h
JA19-20
Absrobance
Wavelength (nm)
0
0.1
0.2
0.3
0.4
0.5
0.6
0.7
0.8
0.9
1
220 240 260 280 300 320 340 360 380 400
blank
pH 7.0
pH 7.5
pH 8.0
pH 8.5
pH 9.0
pH 9.5
pH 10.0
pH 10.5

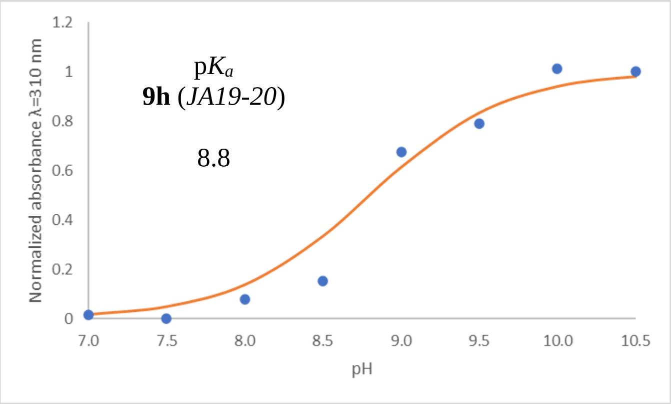
pK_a
9h (*JA19-20*)
8.8
Normalized absorbance λ=310 nm
pH
0
0.2
0.4
0.6
0.8
1
1.2
7.0 7.5 8.0 8.5 9.0 9.5 10.0 10.5

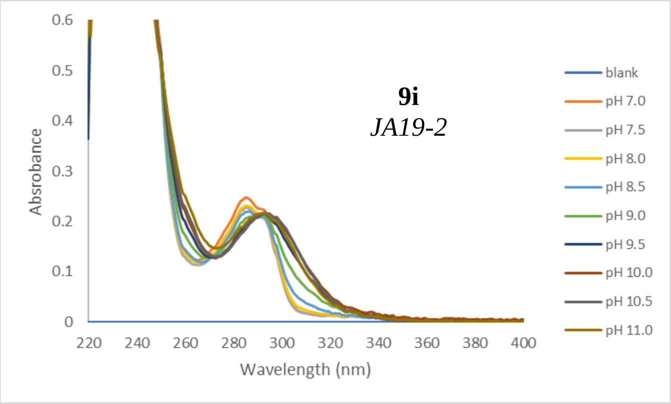
9i
JA19-2
Absrobance
Wavelength (nm)
0
0.1
0.2
0.3
0.4
0.5
0.6
220 240 260 280 300 320 340 360 380 400
blank
pH 7.0
pH 7.5
pH 8.0
pH 8.5
pH 9.0
pH 9.5
pH 10.0
pH 10.5
pH 11.0

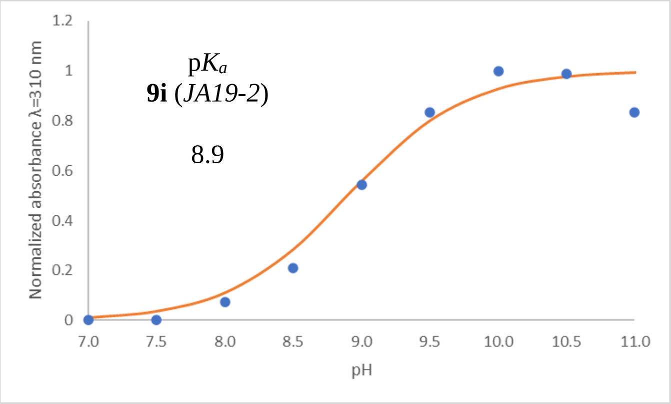
pK_a
9i (*JA19-2*)
8.9
Normalized absorbance λ=310 nm
pH
0
0.2
0.4
0.6
0.8
1
1.2
7.0 7.5 8.0 8.5 9.0 9.5 10.0 10.5 11.0

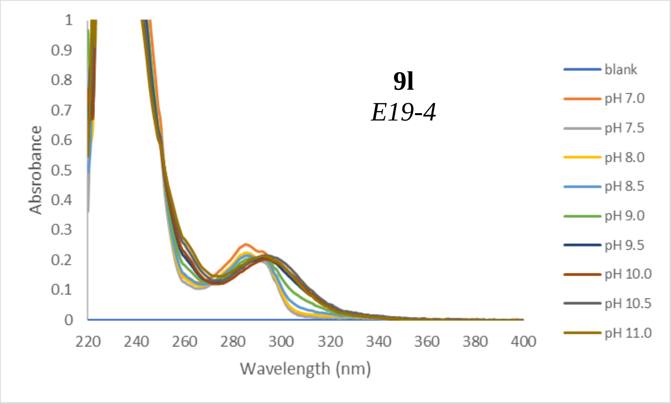
9l
E19-4
Absrobance
Wavelength (nm)
0
0.1
0.2
0.3
0.4
0.5
0.6
0.7
0.8
0.9
1
220 240 260 280 300 320 340 360 380 400
blank
pH 7.0
pH 7.5
pH 8.0
pH 8.5
pH 9.0
pH 9.5
pH 10.0
pH 10.5
pH 11.0

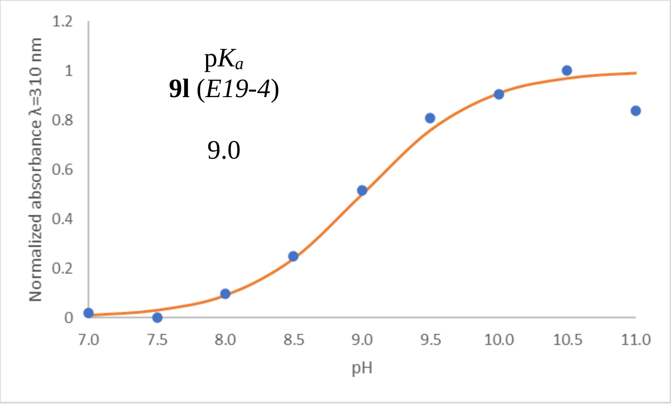
pK_a
9l (*E19-4*)
9.0
Normalized absorbance λ=310 nm
pH
0
0.2
0.4
0.6
0.8
1
1.2
7.0 7.5 8.0 8.5 9.0 9.5 10.0 10.5 11.0

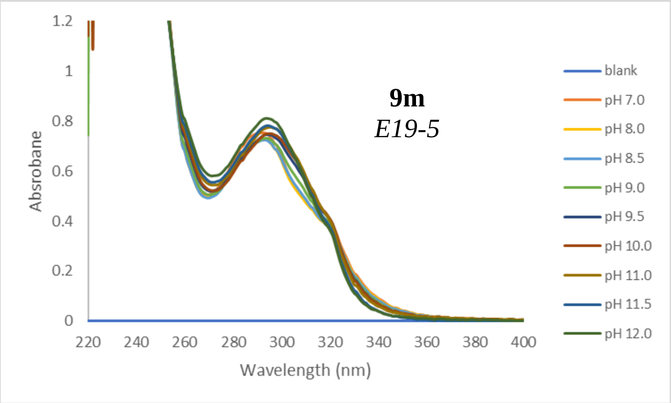
9m
E19-5
Absrobane
Wavelength (nm)
1.2
1
0.8
0.6
0.4
0.2
0
220
240
260
280
300
320
340
360
380
400
blank
pH 7.0
pH 8.0
pH 8.5
pH 9.0
pH 9.5
pH 10.0
pH 11.0
pH 11.5
pH 12.0

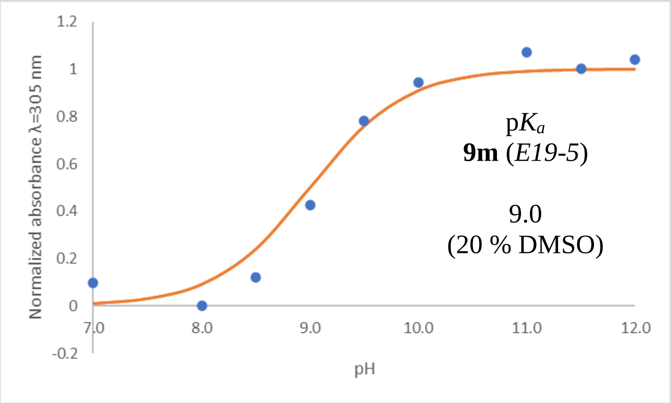
Normalized absorbance λ=305 nm
pH
1.2
1
0.8
0.6
0.4
0.2
0
-0.2
7.0
8.0
9.0
10.0
11.0
12.0
pK_a
9m (*E19-5*)
9.0
(20 % DMSO)

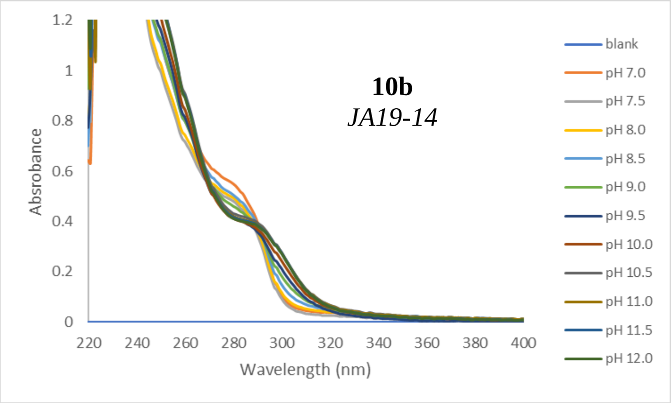
10b
JA19-14
Absrobane
Wavelength (nm)
1.2
1
0.8
0.6
0.4
0.2
0
220
240
260
280
300
320
340
360
380
400
blank
pH 7.0
pH 7.5
pH 8.0
pH 8.5
pH 9.0
pH 9.5
pH 10.0
pH 10.5
pH 11.0
pH 11.5
pH 12.0

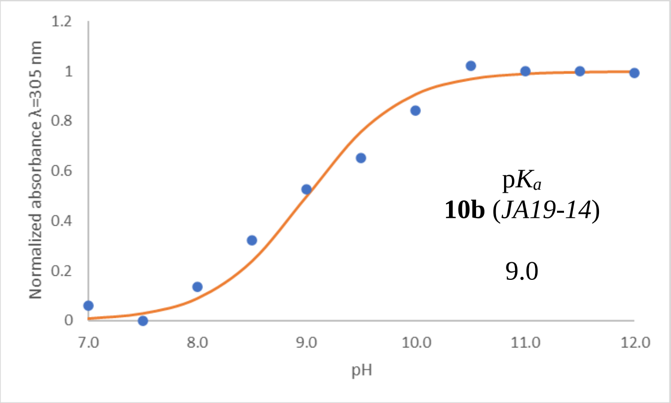
Normalized absorbance λ=305 nm
pH
1.2
1
0.8
0.6
0.4
0.2
0
7.0
8.0
9.0
10.0
11.0
12.0
pK_a
10b (*JA19-14*)
9.0

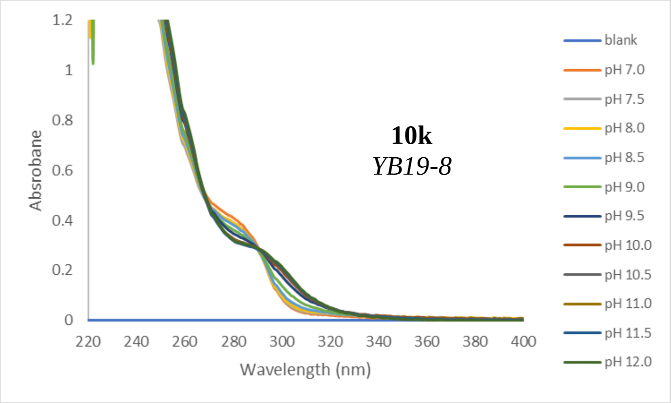
10k
YB19-8
Absrobane
Wavelength (nm)
1.2
1
0.8
0.6
0.4
0.2
0
220
240
260
280
300
320
340
360
380
400
blank
pH 7.0
pH 7.5
pH 8.0
pH 8.5
pH 9.0
pH 9.5
pH 10.0
pH 10.5
pH 11.0
pH 11.5
pH 12.0

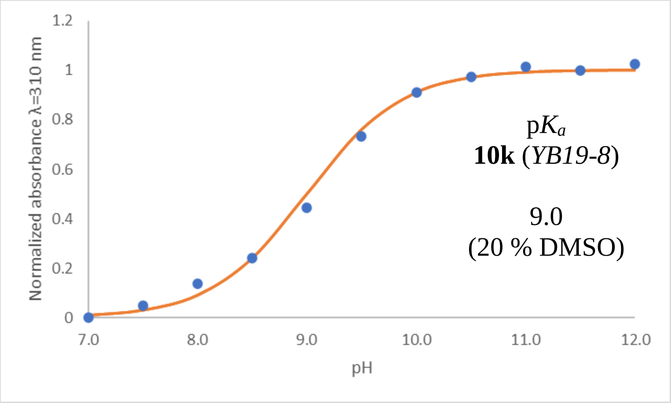
Normalized absorbance λ=310 nm
pH
1.2
1
0.8
0.6
0.4
0.2
0
7.0
8.0
9.0
10.0
11.0
12.0
pK_a
10k (*YB19-8*)
9.0
(20 % DMSO)

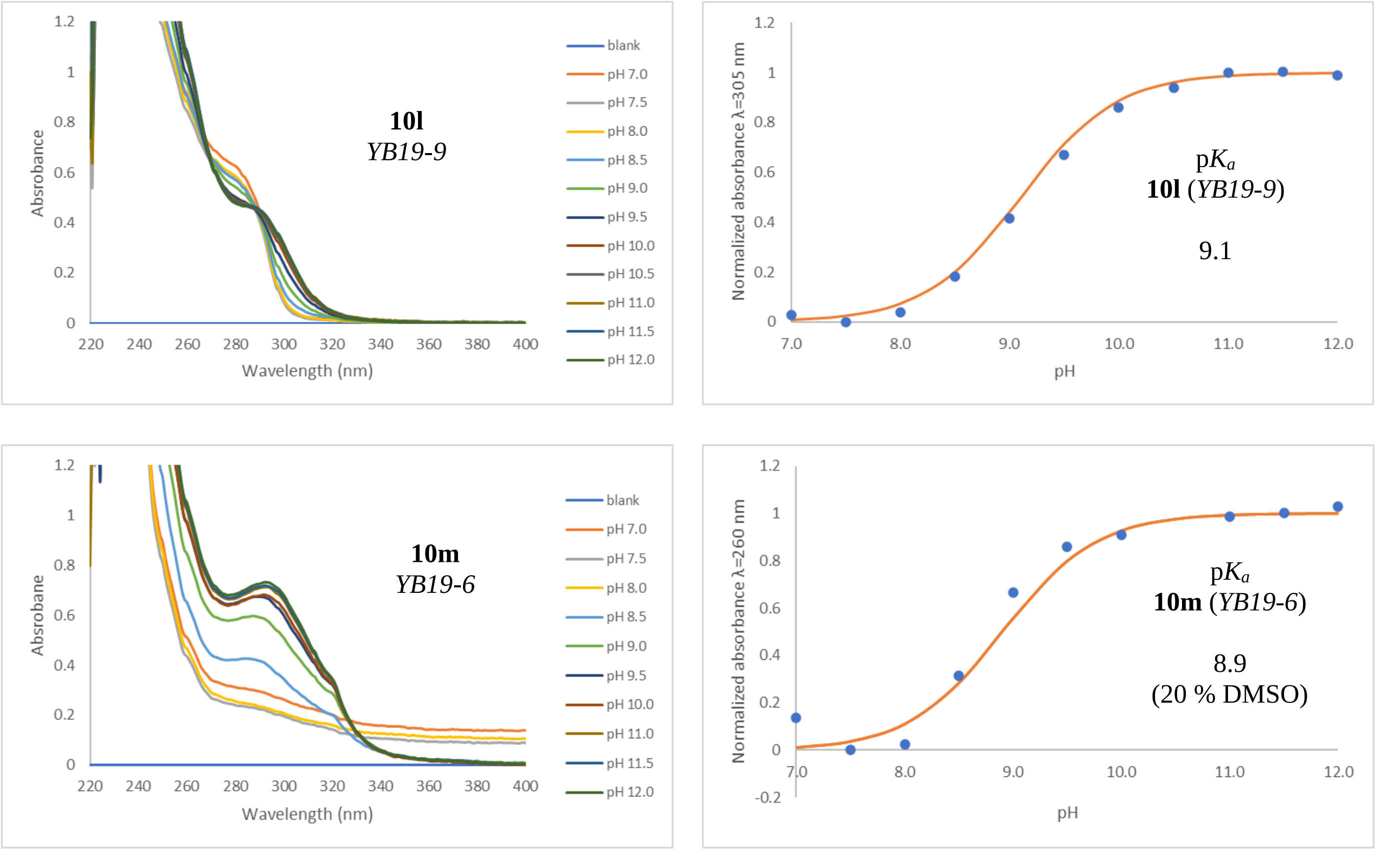

Figure S3. Determination of p$K_{a,\mathrm{SA}}$ values of sulfonamide amino group by UV-VIS spectrophotometry. UV absorbance spectra of the compound solution in buffers of various pH values (left column) and dependence of normalized absorbance on pH (right column) are used to calculate the pK_a according to the Henderson–Hasselbach equation. The buffer was prepared by mixing various ratios of 50 mM phosphate, 50 mM acetate, and 25 mM borate, containing 50 mM NaCl. All samples contained 2 % (v/v) DMSO or 20 % (v/v) DMSO for less soluble compounds. NA – not available (inconclusive) due to solubility issues or low intensity of the absorbance curves. The spectra were determined at 37 °C.

Comparison of docking and crystallographic binding poses

Table S1. The binding affinities of the series **4** compounds docked into CAII and CAIX were calculated using the Vinardo scoring function, and the corresponding intrinsic Gibbs energy changes of binding (obtained experimentally by FTSA and recalculated using the corresponding ligand and protein p*K*a values).

Compound	CAII binding affinity, kJ/mol		CAIX binding affinity, kJ/mol	
	Calculated[a]	Experimental	Calculated[a]	Experimental
**4a**	-32.7	-58.3	-26.5	-66.7
**4b**	-29.9	-53.1	-26.5	-68.4
**4c**	-32.8	-53.5	-27.3	-64.5
**4d**	-33.5	-52.5	-29.1	-62.7
**4g**	-23.3	-48.7	-28.3	-65.7
**4h**	-25.3	-50.2	-22.5	-54.4
**4i**	-27.2	-51.1	-23.6	-61.0
**4j**	-23.5	-48.9	-23.2	-55.2
**4k**	-29.2	-54.7	-25.4	-68.4
**4l**	-30.0	-52.1	-25.3	-66.7
**4m**	-35.4	-59.4	-31.2	-68.4

[a] The significant difference between the computed and experimental binding affinities is due to the scoring function not considering interaction with zinc, which is likely to be nearly constant.

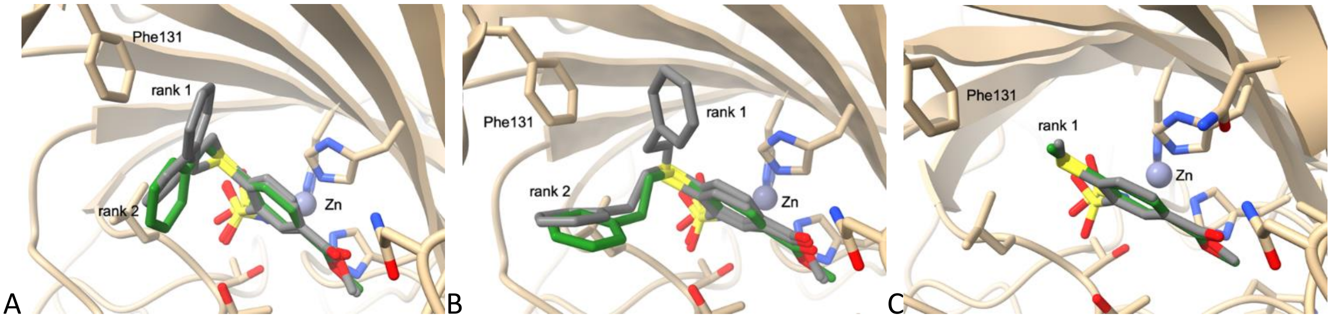

Figure S4. X-ray structures (green) of (A) **4c** (PDB ID: 9FPQ), (B) **4d** (PDB ID: 9FPR), and (C) **4h** (PDB ID: 9FPS) bound to CAII superposed with the docked ligands (gray). The docking reproduces the scaffold quite well in (C), while (A) and (B) show difficulty placing flexible hydrophobic substituents near Phe131. However, rank 2 poses in (A) and (B) correspond much better to the experimental conformations than rank 1, apparently due to the difficulty of the force field in capturing subtleties of the π-π interaction (parallel or T-shaped) interactions with Phe131.

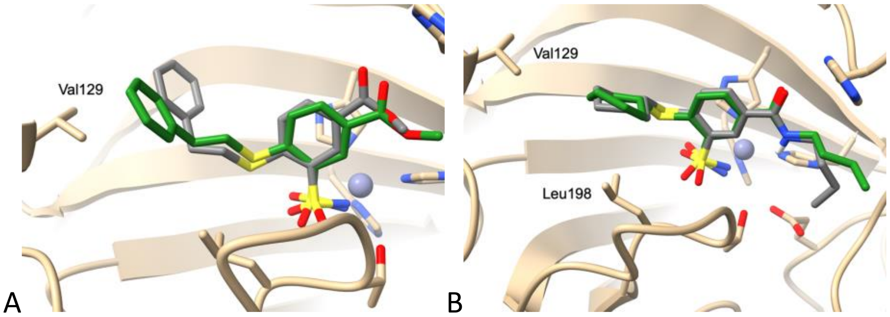

Figure S5. X-ray structures (green) and best-ranked docked (gray) conformations of (A) **4d** (PDB ID: 9R8X) and (B) **5b** (PDB ID: 9R8Y) bound to CAIX.

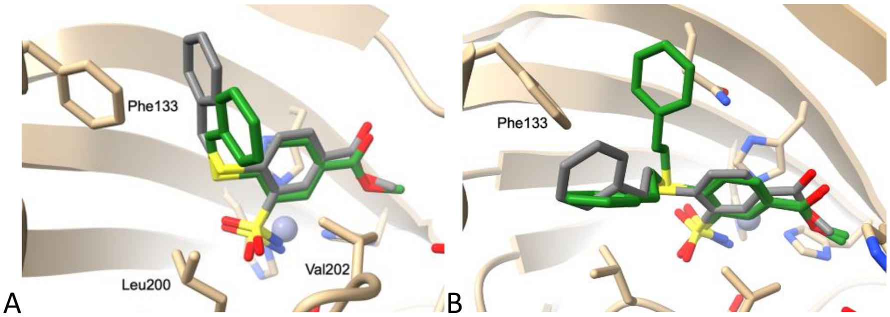

Figure S6. X-ray structures (green) and best-ranked docked (gray) conformations of (A) **4c** (PDB ID: 9FPV) and (B) **4d** (PDB ID: 9FPW) bound to CAXIII. In the X-ray structure in (B), two alternative ligand conformations exist in two chains; docking reproduces one quite well (RMSD=0.95 Å).

NMR spectra of synthesized compounds

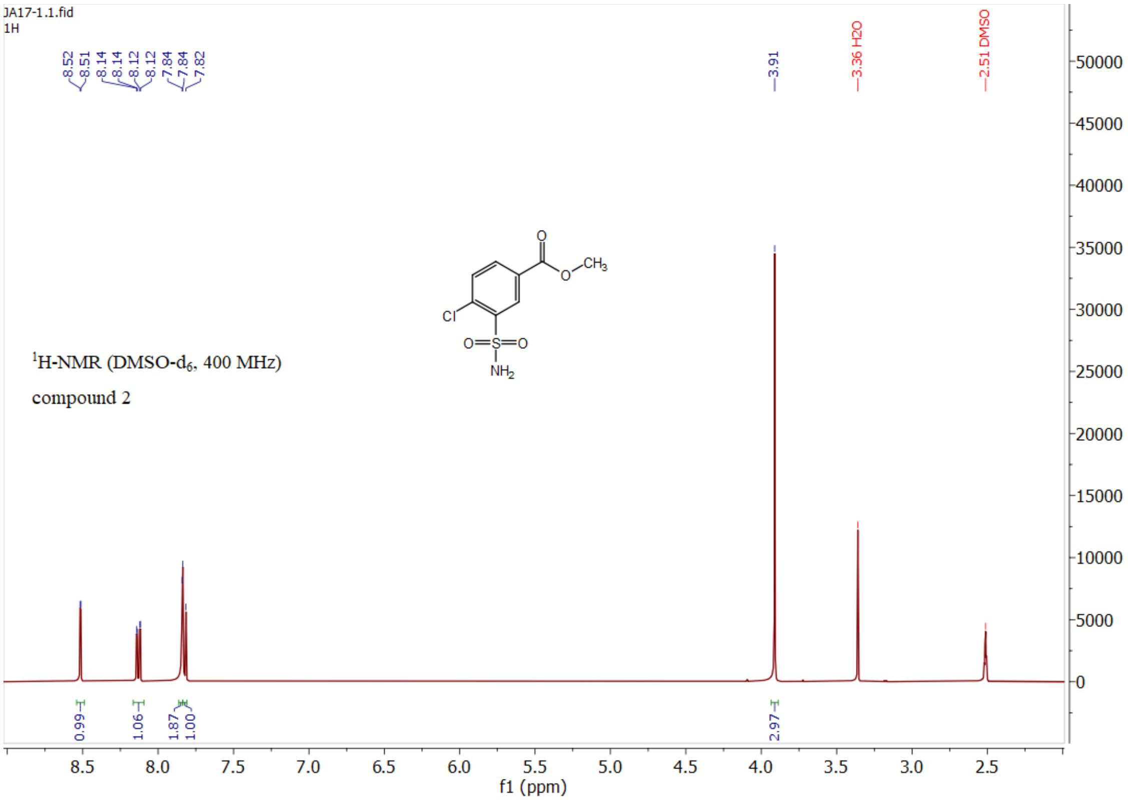

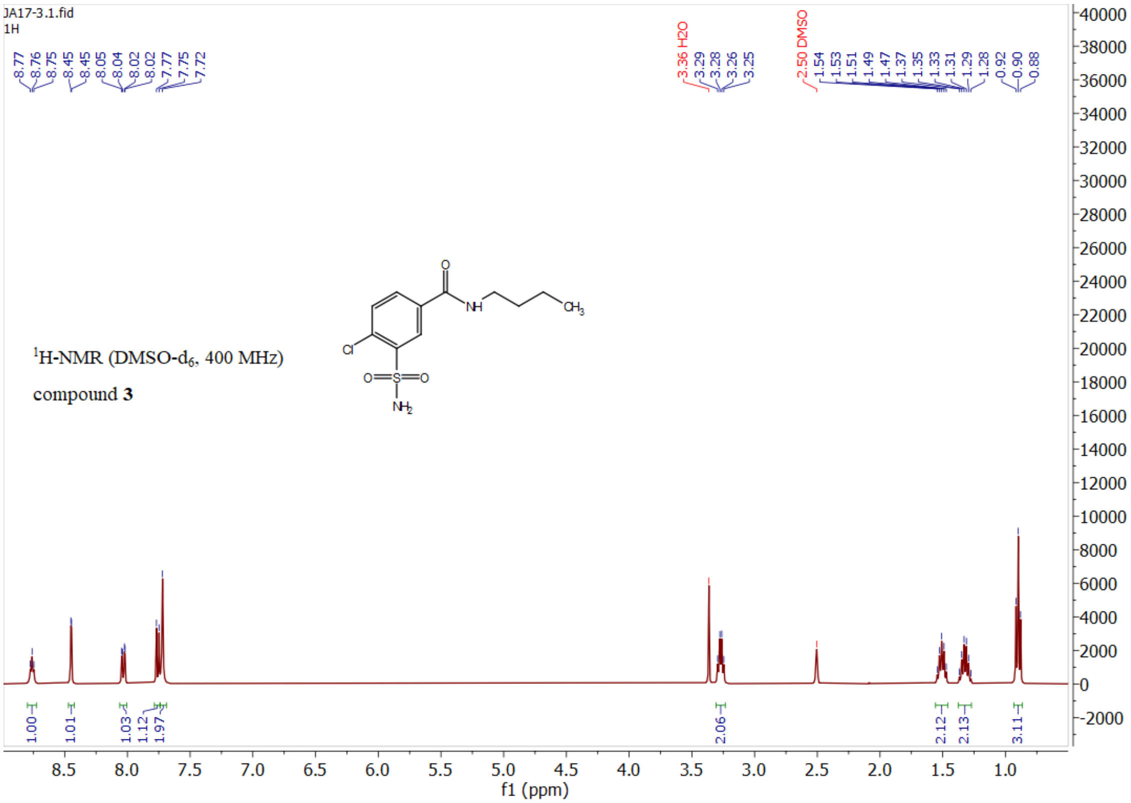

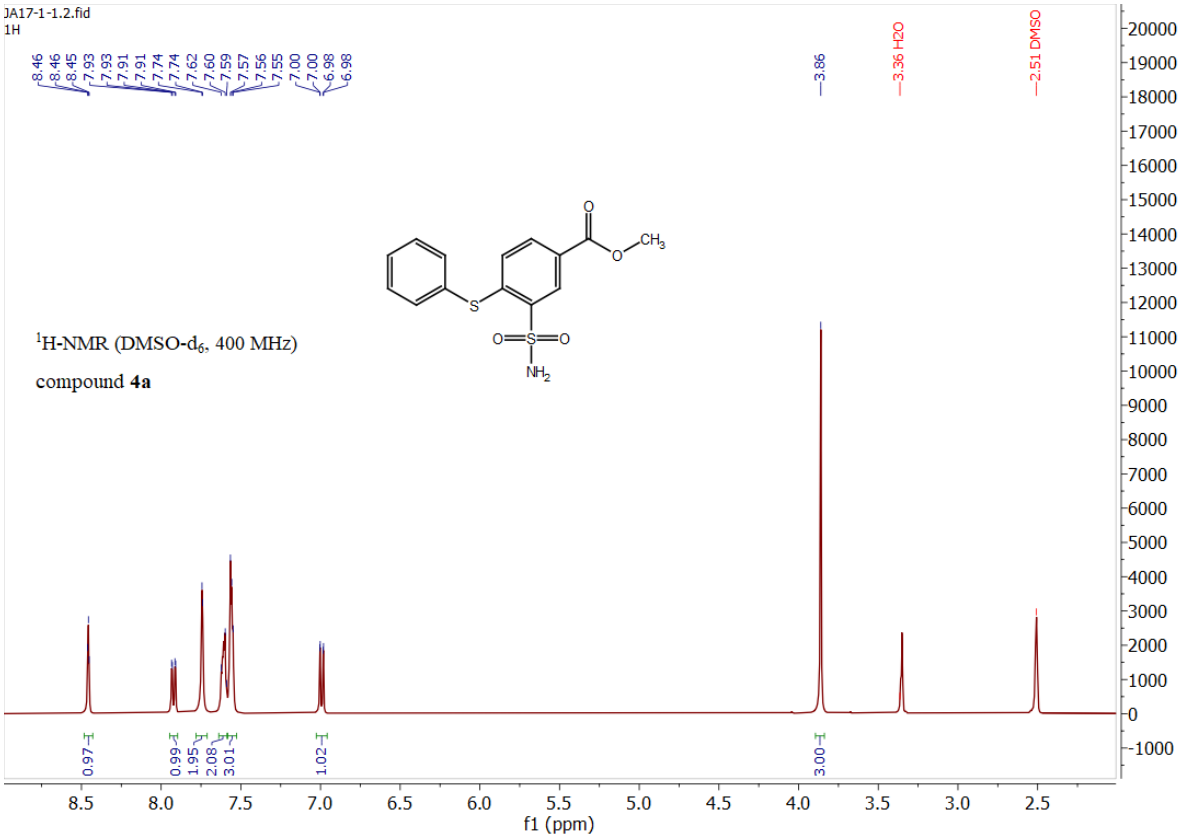

JA17-1-1.2.fid
1H
8.46
8.46
8.45
7.93
7.93
7.91
7.91
7.74
7.74
7.62
7.60
7.59
7.57
7.56
7.55
7.00
7.00
6.98
6.98
3.86
3.36 H2O
2.51 DMSO
^{1}H-NMR (DMSO-d$_6$, 400 MHz)
compound **4a**
0.97
0.99
1.95
2.08
3.01
1.02
3.00
f1 (ppm)

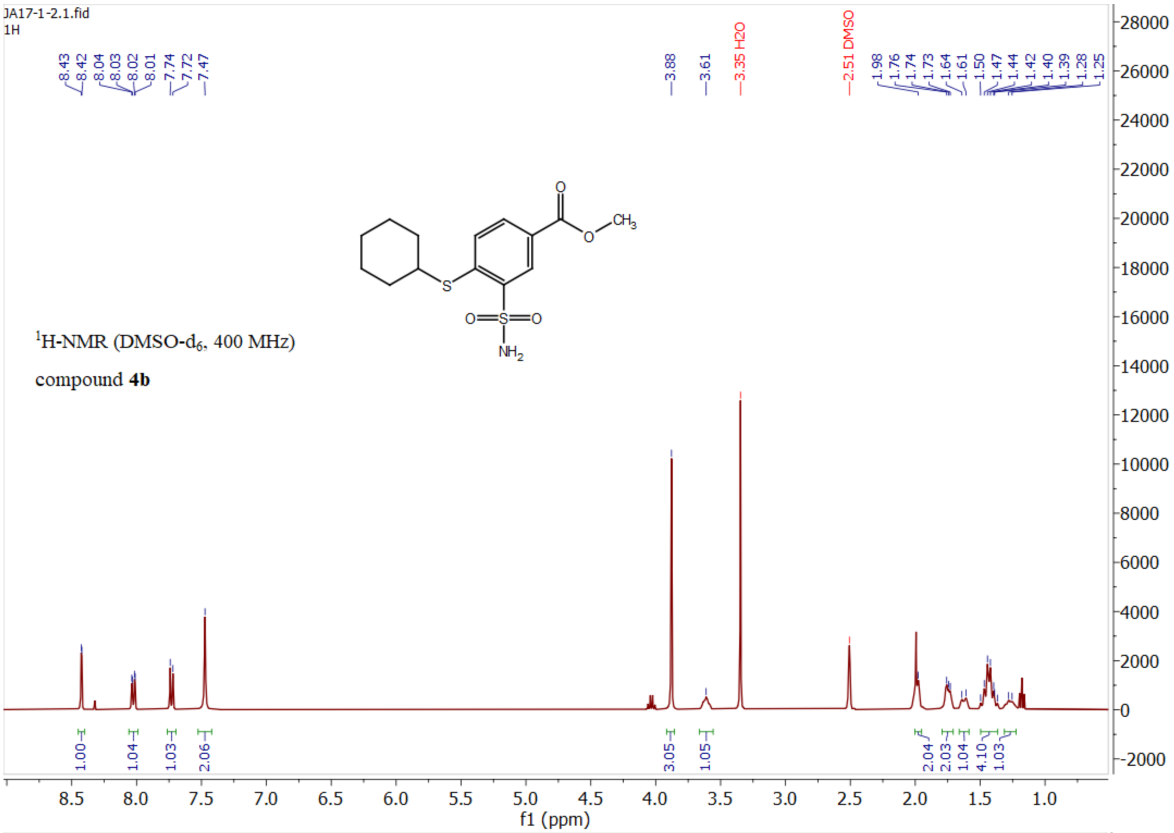

^{1}H-NMR (DMSO-d$_6$, 400 MHz)

compound **4b**

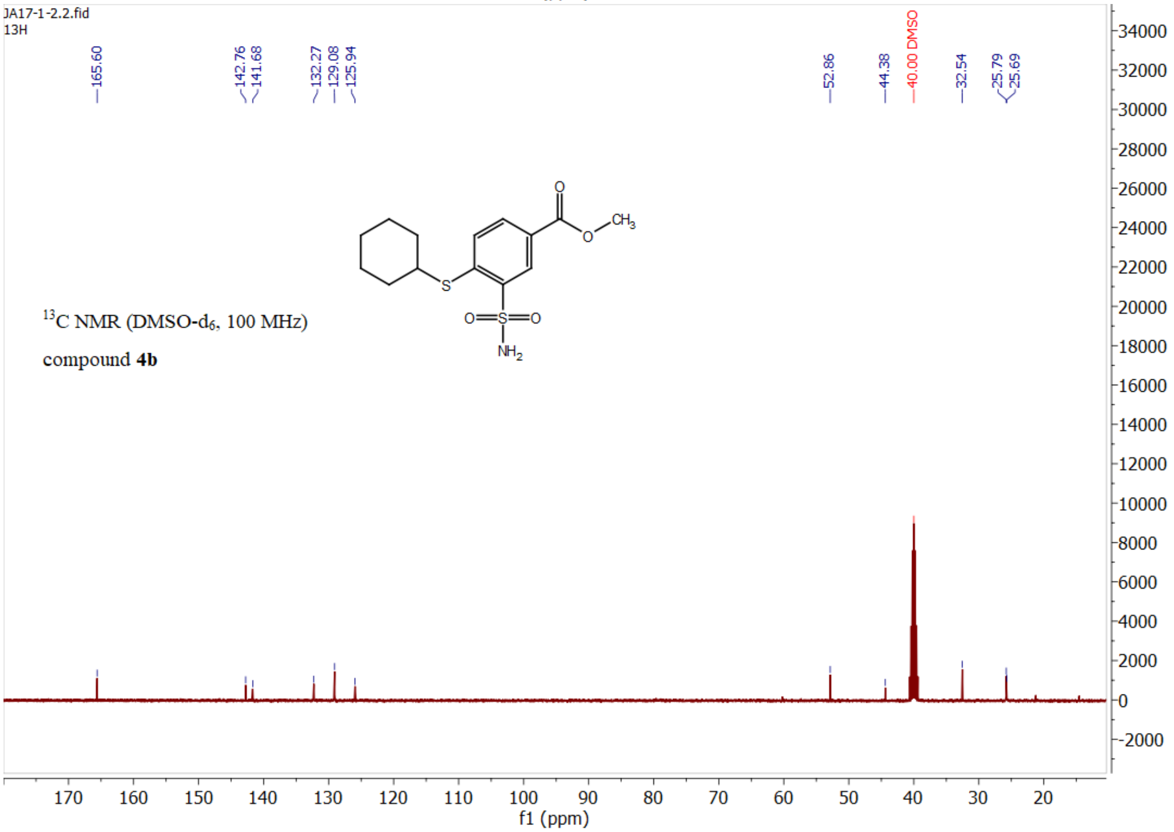

^{13}C NMR (DMSO-d$_6$, 100 MHz)

compound **4b**

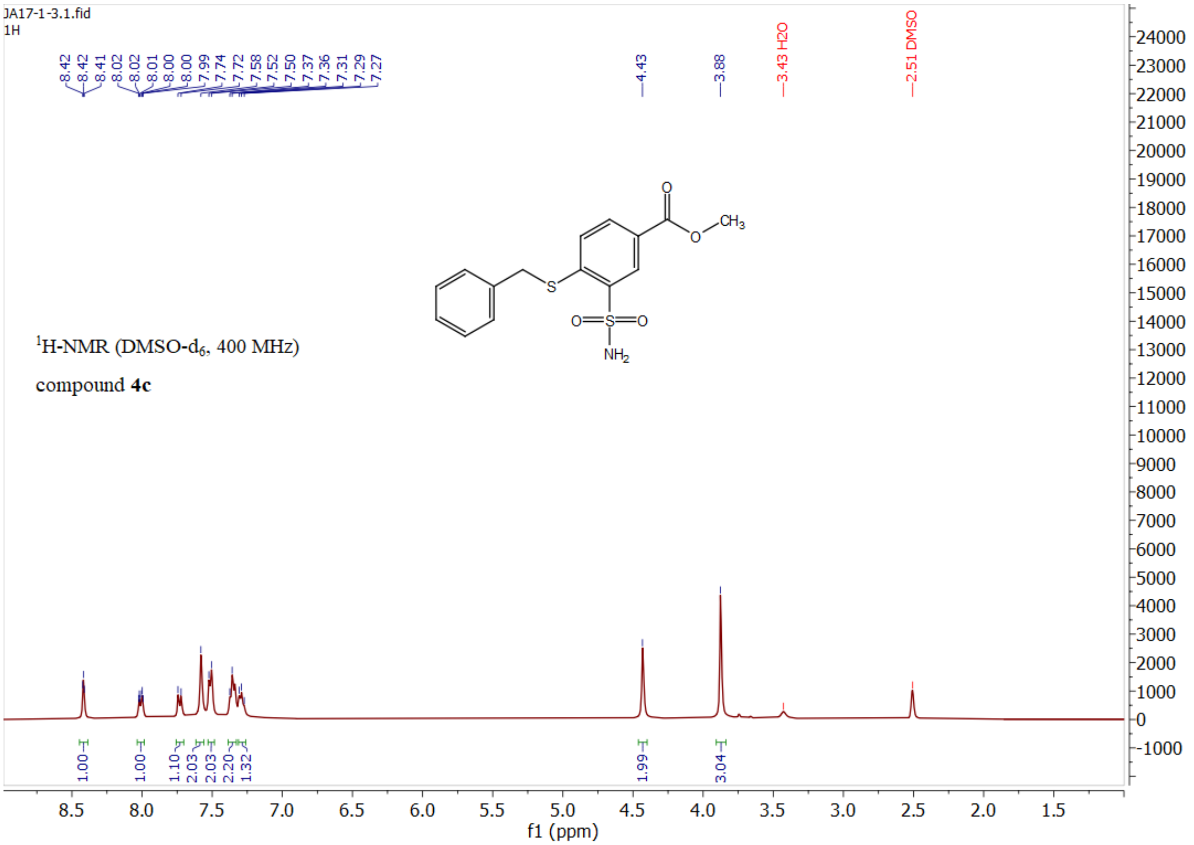
JA17-1-3.1.fid
1H
8.42 8.42 8.41 8.02 8.02 8.01 8.00 8.00 7.99 7.74 7.72 7.58 7.52 7.50 7.37 7.36 7.31 7.29 7.27
4.43
3.88
3.43 H2O
2.51 DMSO
^{1}H-NMR (DMSO-d$_6$, 400 MHz)
compound **4c**
1.00 1.00 1.10 2.03 2.03 2.20 1.32 1.99 3.04
f1 (ppm)

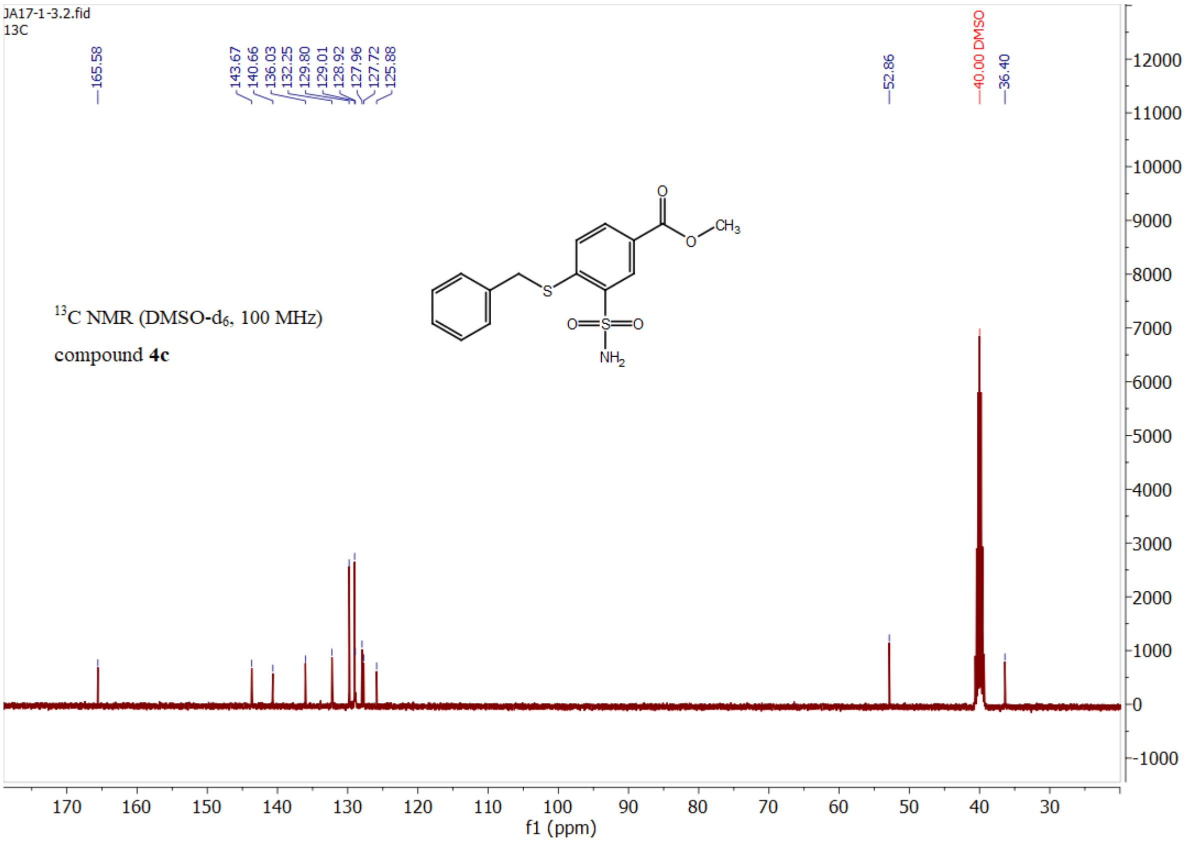
JA17-1-3.2.fid
13C
165.58
143.67 140.66 136.03 132.25 129.80 129.01 128.92 127.96 127.72 125.88
52.86
40.00 DMSO
36.40
^{13}C NMR (DMSO-d$_6$, 100 MHz)
compound **4c**
f1 (ppm)

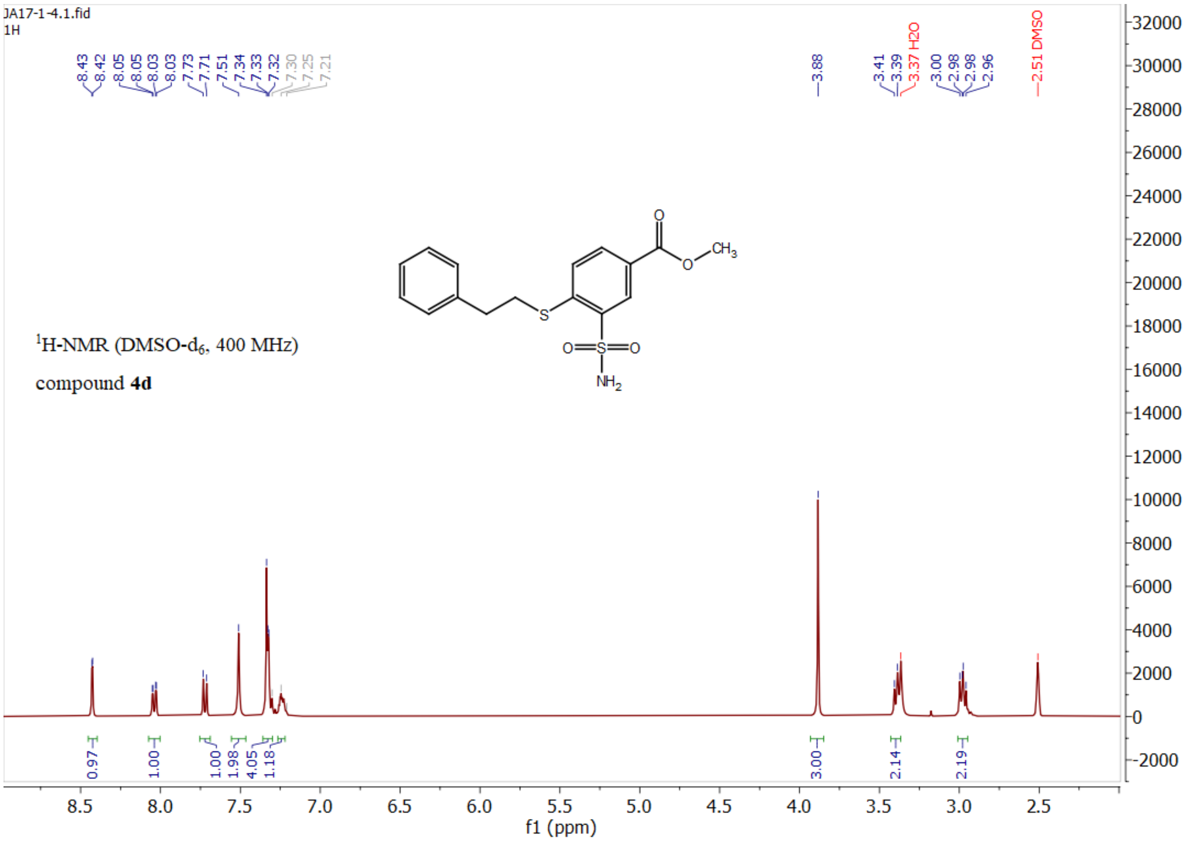
JA17-1-4.1.fid
1H
^{1}H-NMR (DMSO-d6, 400 MHz)
compound 4d
f1 (ppm)
3.37 H2O
2.51 DMSO

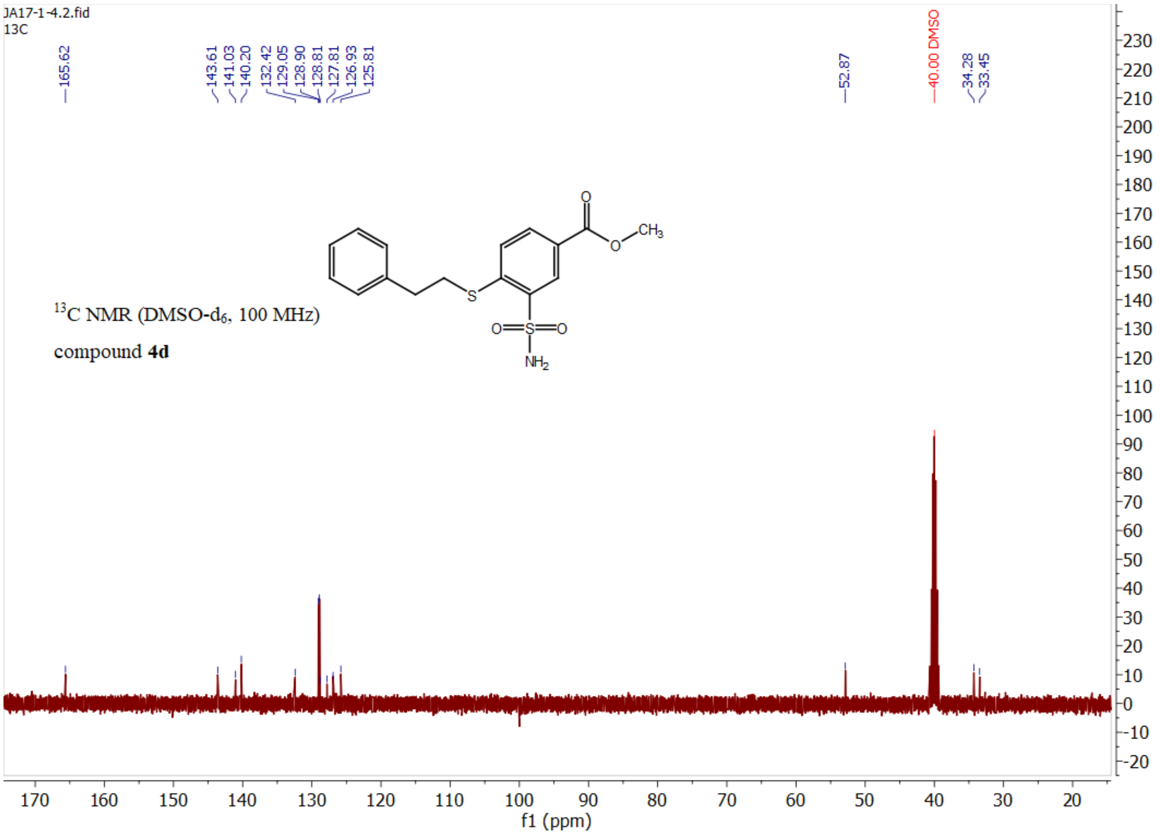
JA17-1-4.2.fid
13C
^{13}C NMR (DMSO-d6, 100 MHz)
compound 4d
f1 (ppm)
40.00 DMSO

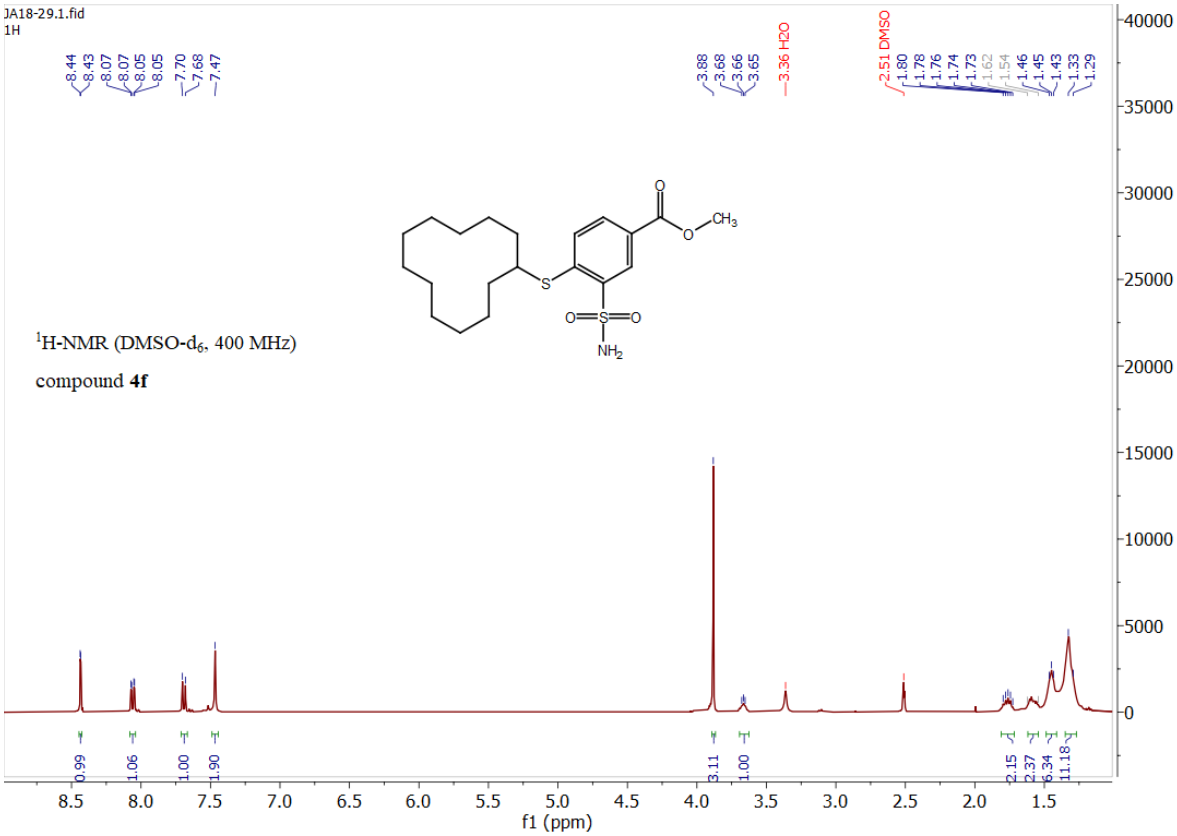
JA18-29.1.fid
1H
8.44
8.43
8.07
8.07
8.05
8.05
7.70
7.68
7.47
3.88
3.68
3.66
3.65
3.36 H2O
2.51 DMSO
1.80
1.78
1.76
1.74
1.73
1.62
1.54
1.46
1.45
1.43
1.33
1.29
O
CH3
O
S
O=S=O
NH2
1H-NMR (DMSO-d6, 400 MHz)
compound 4f
0.99
1.06
1.00
1.90
3.11
1.00
2.15
2.37
6.34
11.18
f1 (ppm)

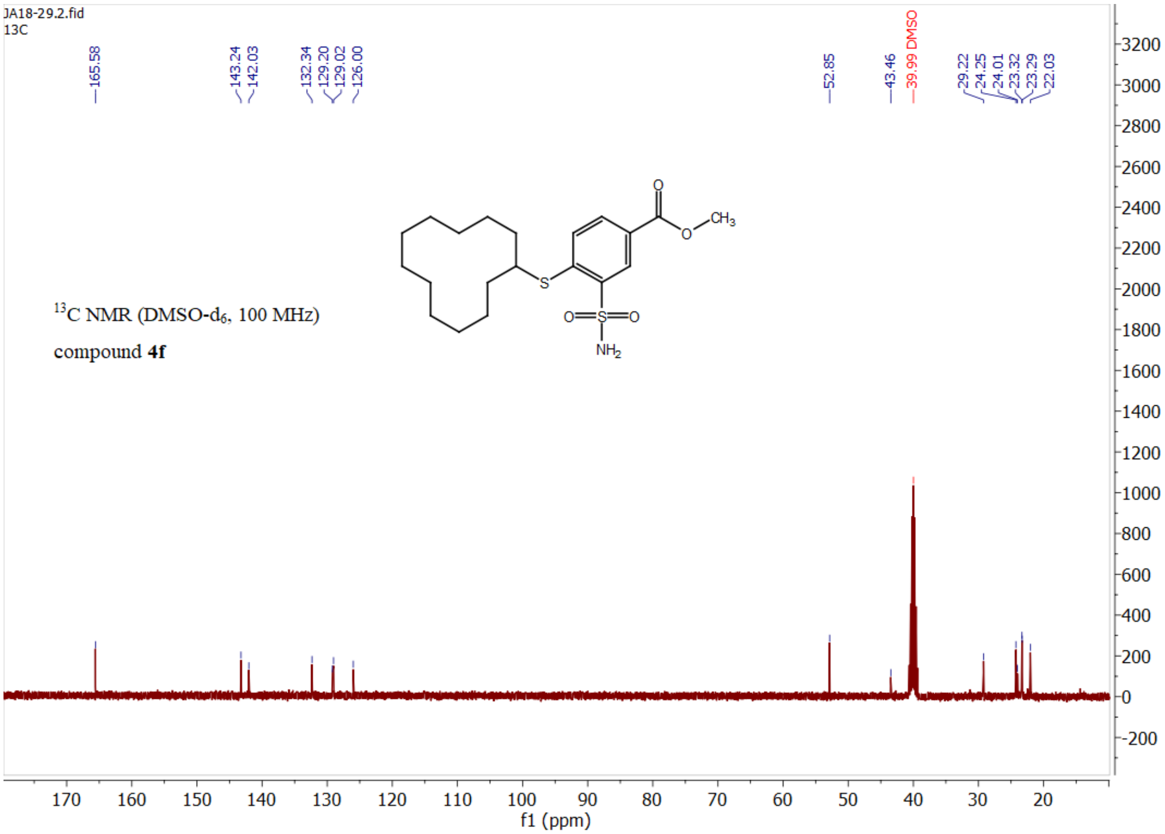
JA18-29.2.fid
13C
165.58
143.24
142.03
132.34
129.20
129.02
126.00
52.85
43.46
39.99 DMSO
29.22
24.25
24.01
23.32
23.29
22.03
O
CH3
O
S
O=S=O
NH2
13C NMR (DMSO-d6, 100 MHz)
compound 4f
f1 (ppm)

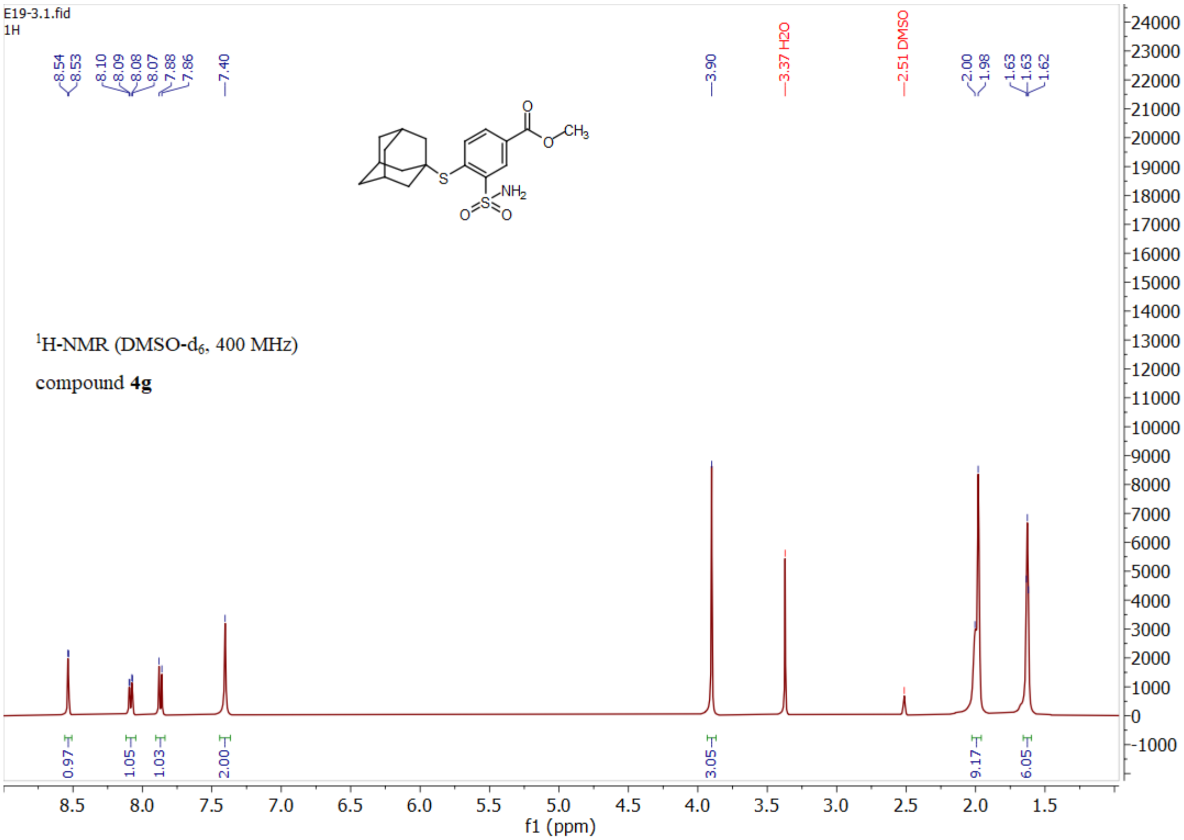
E19-3.1.fid
1H
8.54
8.53
8.10
8.09
8.08
8.07
7.88
7.86
7.40
3.90
3.37 H2O
2.51 DMSO
2.00
1.98
1.63
1.63
1.62
CH3
O
S
NH2
^{1}H-NMR (DMSO-d6, 400 MHz)
compound 4g
0.97
1.05
1.03
2.00
3.05
9.17
6.05
f1 (ppm)

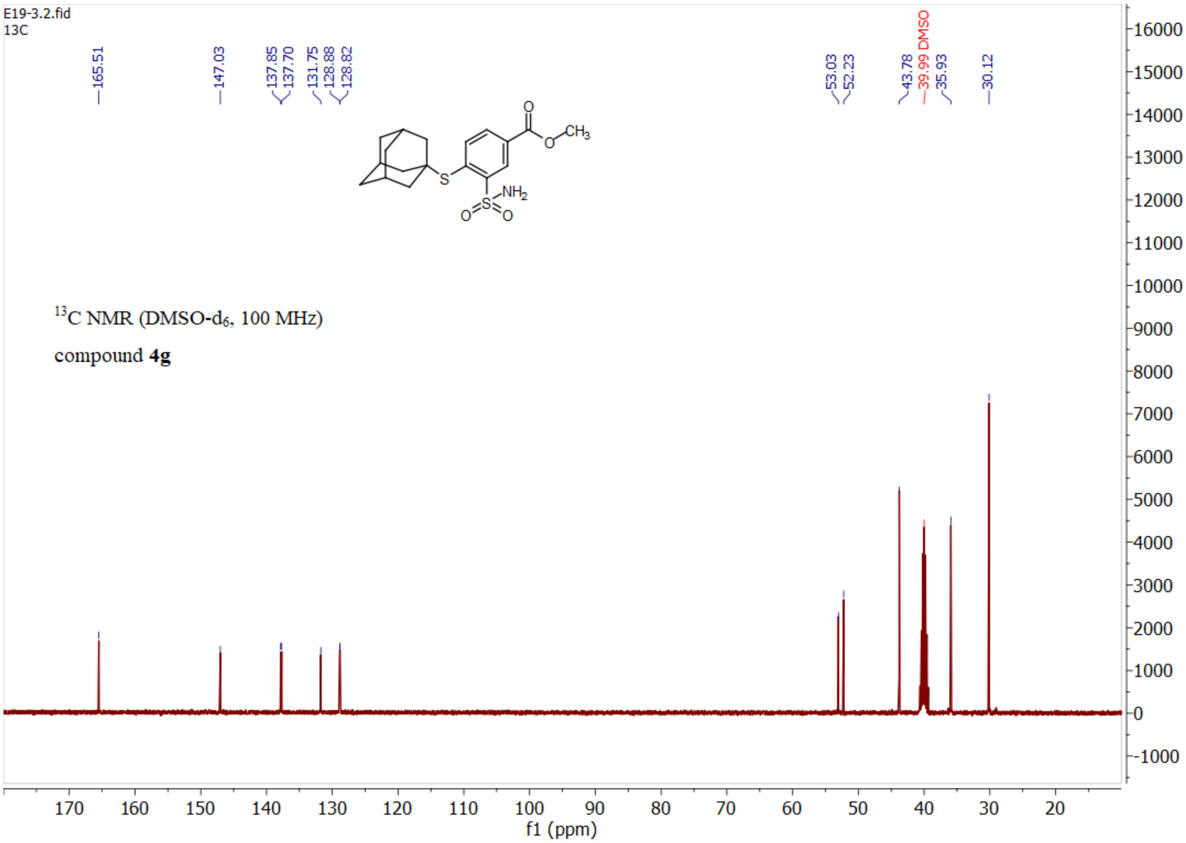
E19-3.2.fid
13C
165.51
147.03
137.85
137.70
131.75
128.88
128.82
53.03
52.23
43.78
39.99 DMSO
35.93
30.12
CH3
O
S
NH2
^{13}C NMR (DMSO-d6, 100 MHz)
compound 4g
f1 (ppm)

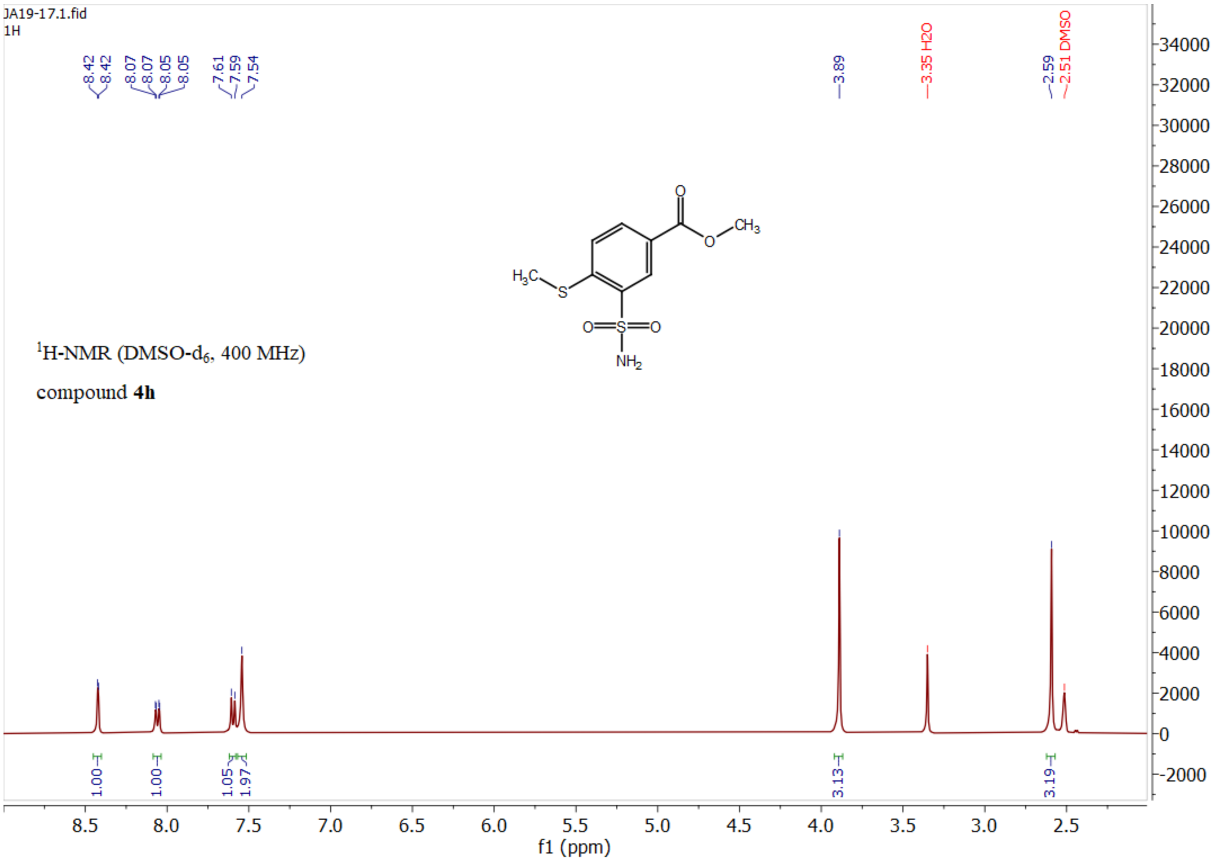
JA19-17.1.fid
1H
8.42
8.42
8.07
8.07
8.05
8.05
7.61
7.59
7.54
3.89
3.35 H2O
2.59
2.51 DMSO
¹H-NMR (DMSO-d6, 400 MHz)
compound 4h
1.00
1.00
1.05
1.97
3.13
3.19
f1 (ppm)

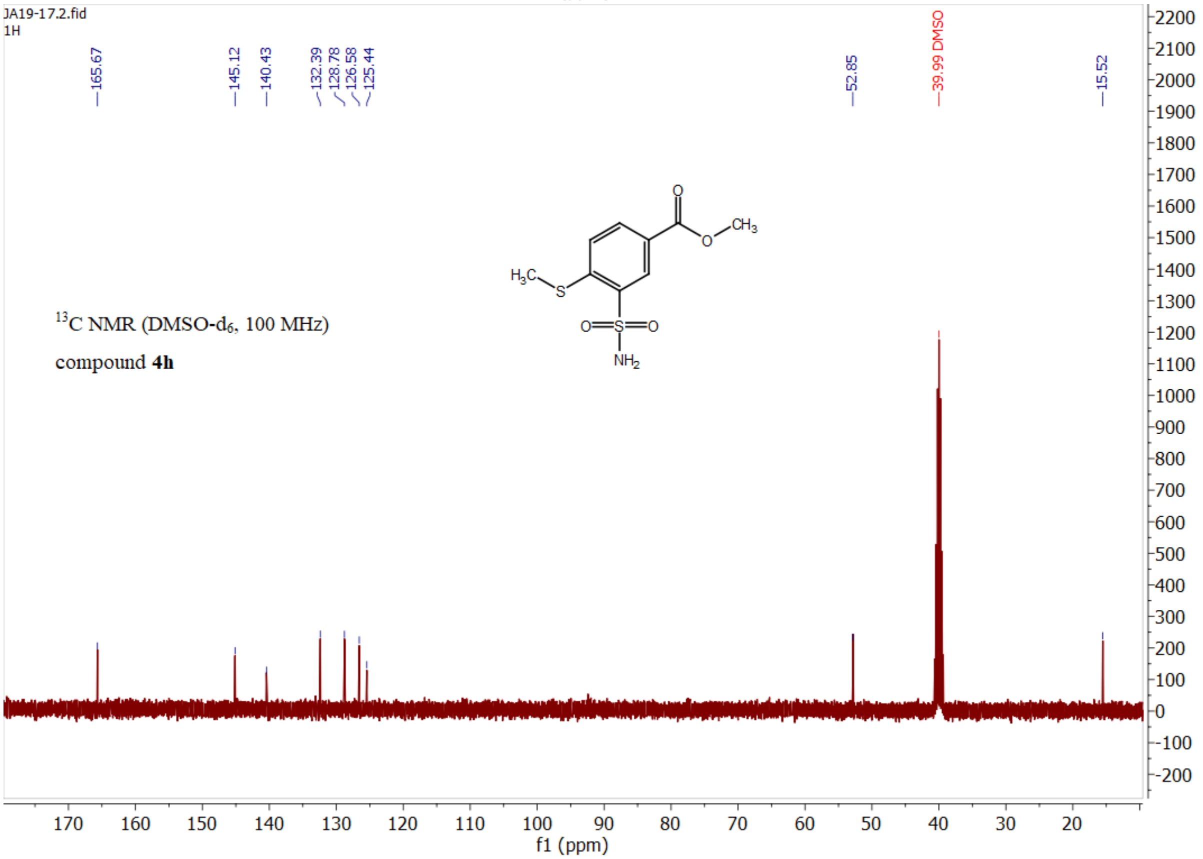
JA19-17.2.fid
1H
165.67
145.12
140.43
132.39
128.78
126.58
125.44
52.85
39.99 DMSO
15.52
¹³C NMR (DMSO-d6, 100 MHz)
compound 4h
f1 (ppm)

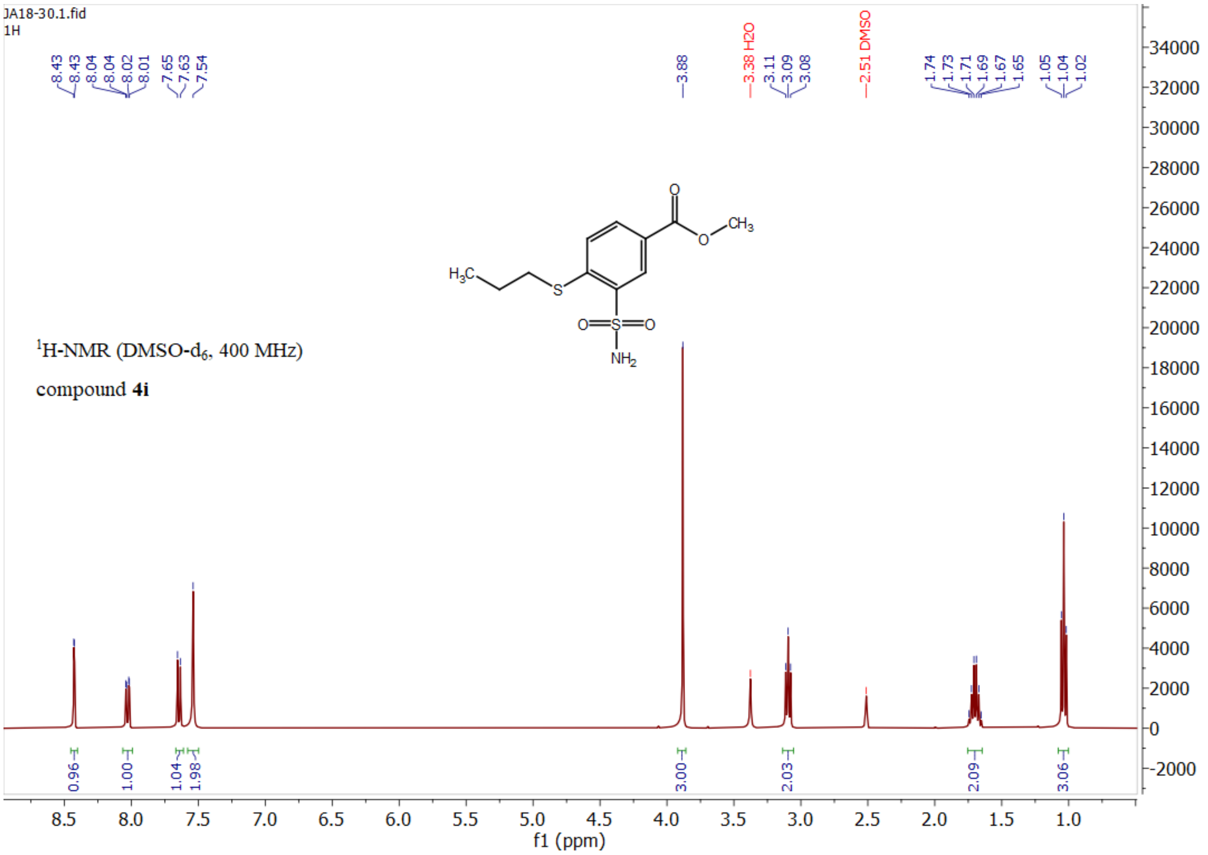
JA18-30.1.fid
1H
8.43
8.43
8.04
8.04
8.02
8.01
7.65
7.63
7.54
3.88
3.38 H2O
3.11
3.09
3.08
2.51 DMSO
1.74
1.73
1.71
1.69
1.67
1.65
1.05
1.04
1.02
^{1}H-NMR (DMSO-d$_6$, 400 MHz)
compound **4i**
0.96
1.00
1.04
1.98
3.00
2.03
2.09
3.06
f1 (ppm)

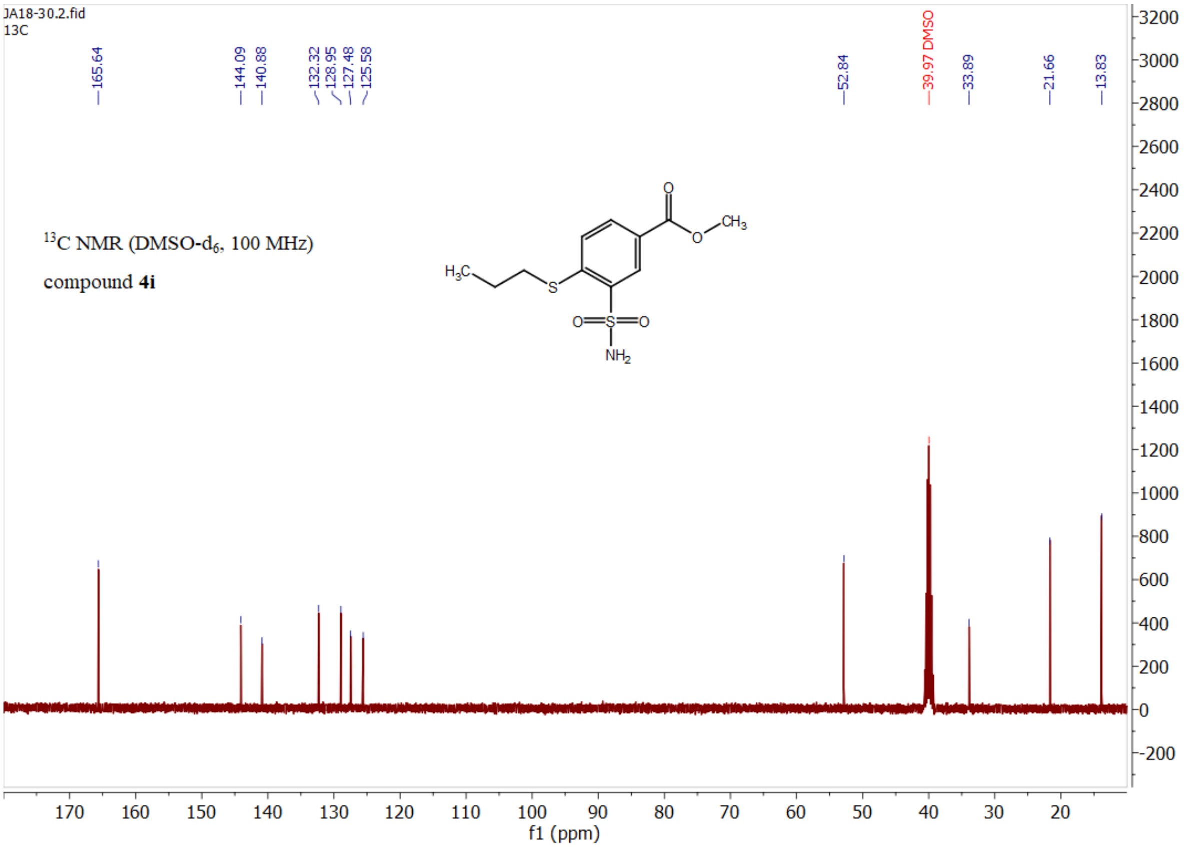
JA18-30.2.fid
13C
165.64
144.09
140.88
132.32
128.95
127.48
125.58
52.84
39.97 DMSO
33.89
21.66
13.83
^{13}C NMR (DMSO-d$_6$, 100 MHz)
compound **4i**
f1 (ppm)

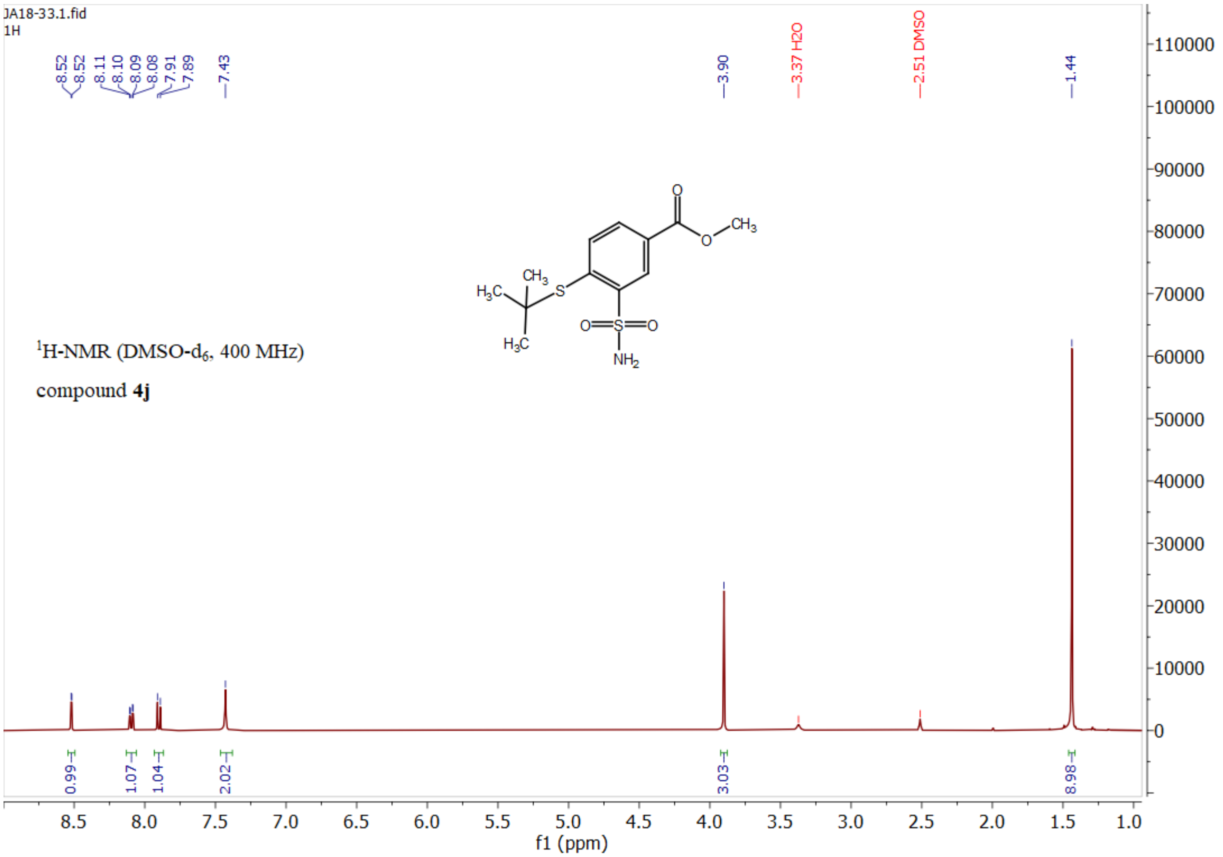
JA18-33.1.fid
1H
8.52
8.52
8.11
8.10
8.09
8.08
7.91
7.89
7.43
3.90
3.37 H2O
2.51 DMSO
1.44
^{1}H-NMR (DMSO-d$_6$, 400 MHz)
compound **4j**
0.99
1.07
1.04
2.02
3.03
8.98
f1 (ppm)

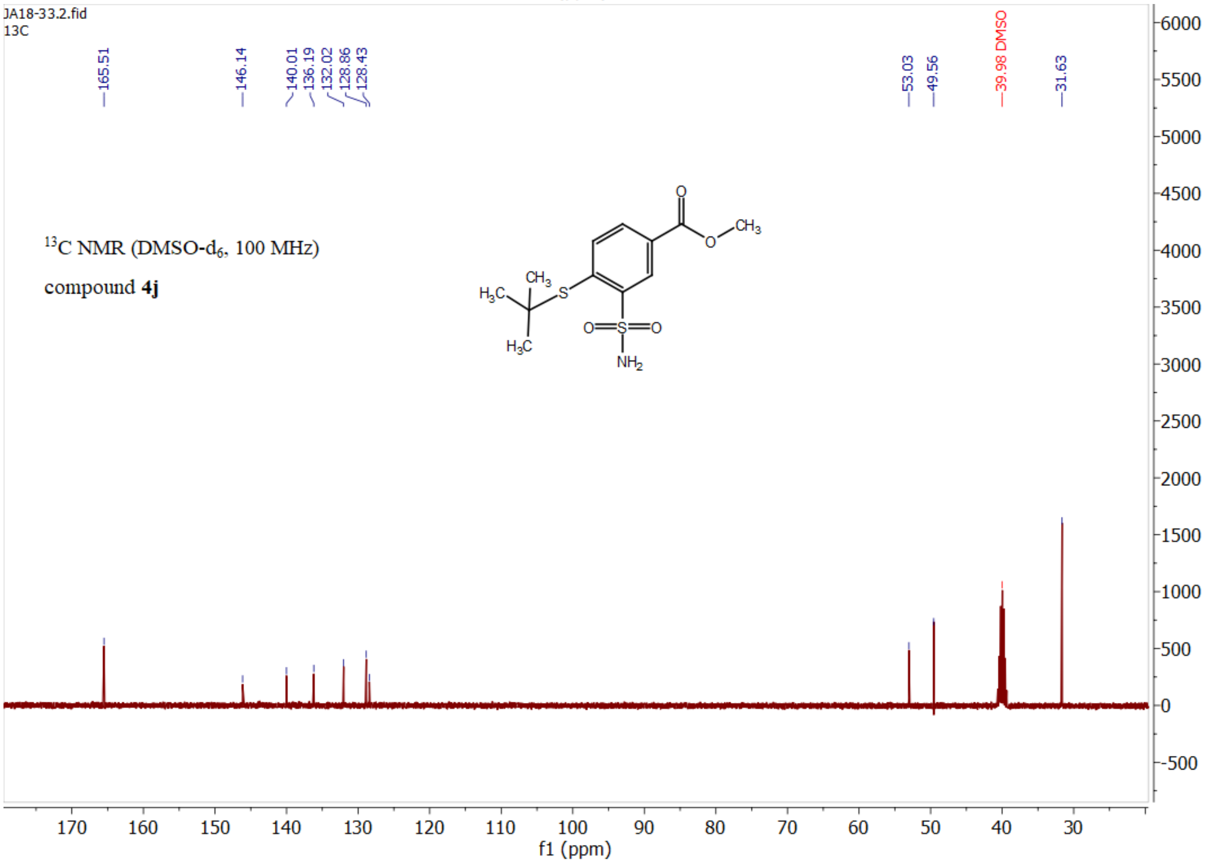
JA18-33.2.fid
13C
165.51
146.14
140.01
136.19
132.02
128.86
128.43
53.03
49.56
39.98 DMSO
31.63
^{13}C NMR (DMSO-d$_6$, 100 MHz)
compound **4j**
f1 (ppm)

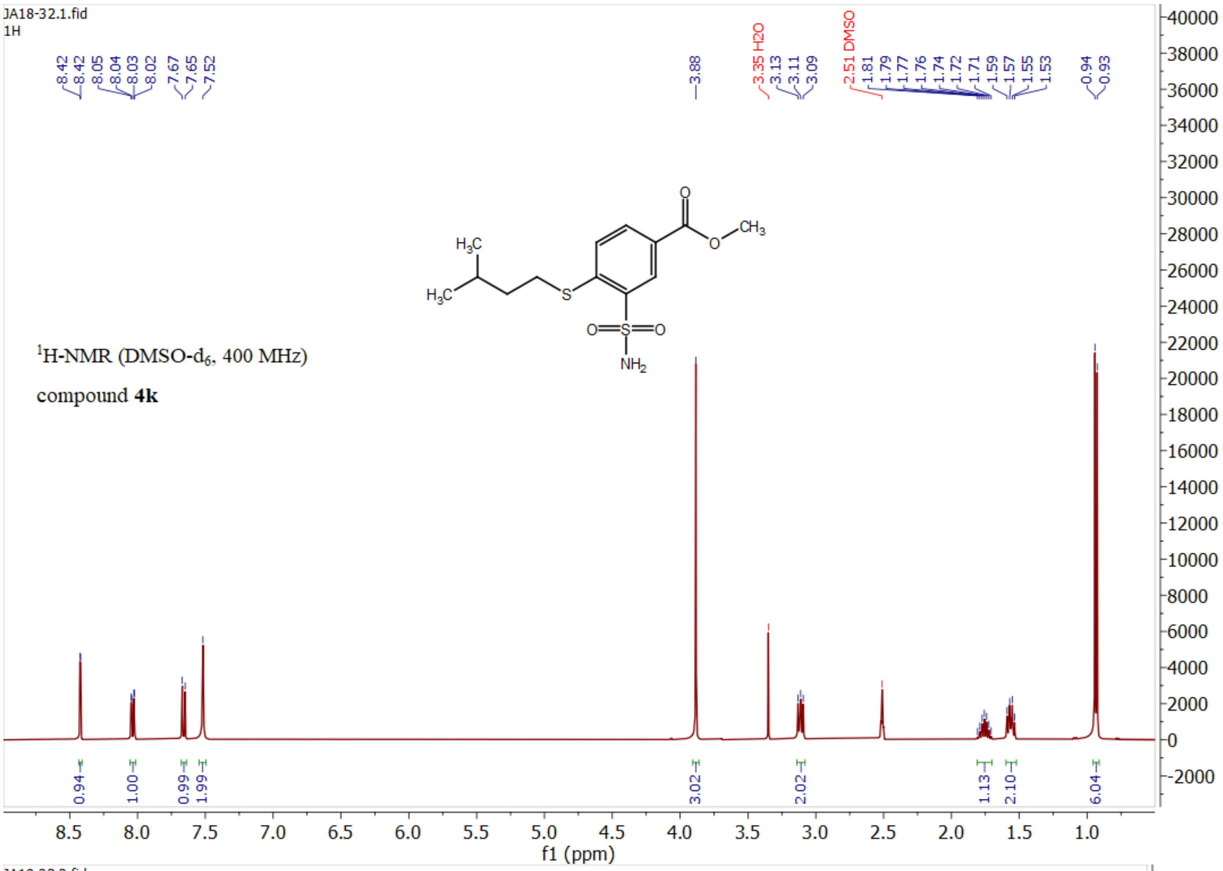
JA18-32.1.fid
1H
8.42
8.42
8.05
8.04
8.03
8.02
7.67
7.65
7.52
3.88
3.35 H2O
3.13
3.11
3.09
2.51 DMSO
1.81
1.79
1.77
1.76
1.74
1.72
1.71
1.59
1.57
1.55
1.53
0.94
0.93
^{1}H-NMR (DMSO-d6, 400 MHz)
compound 4k
0.94
1.00
0.99
1.99
3.02
2.02
1.13
2.10
6.04
f1 (ppm)

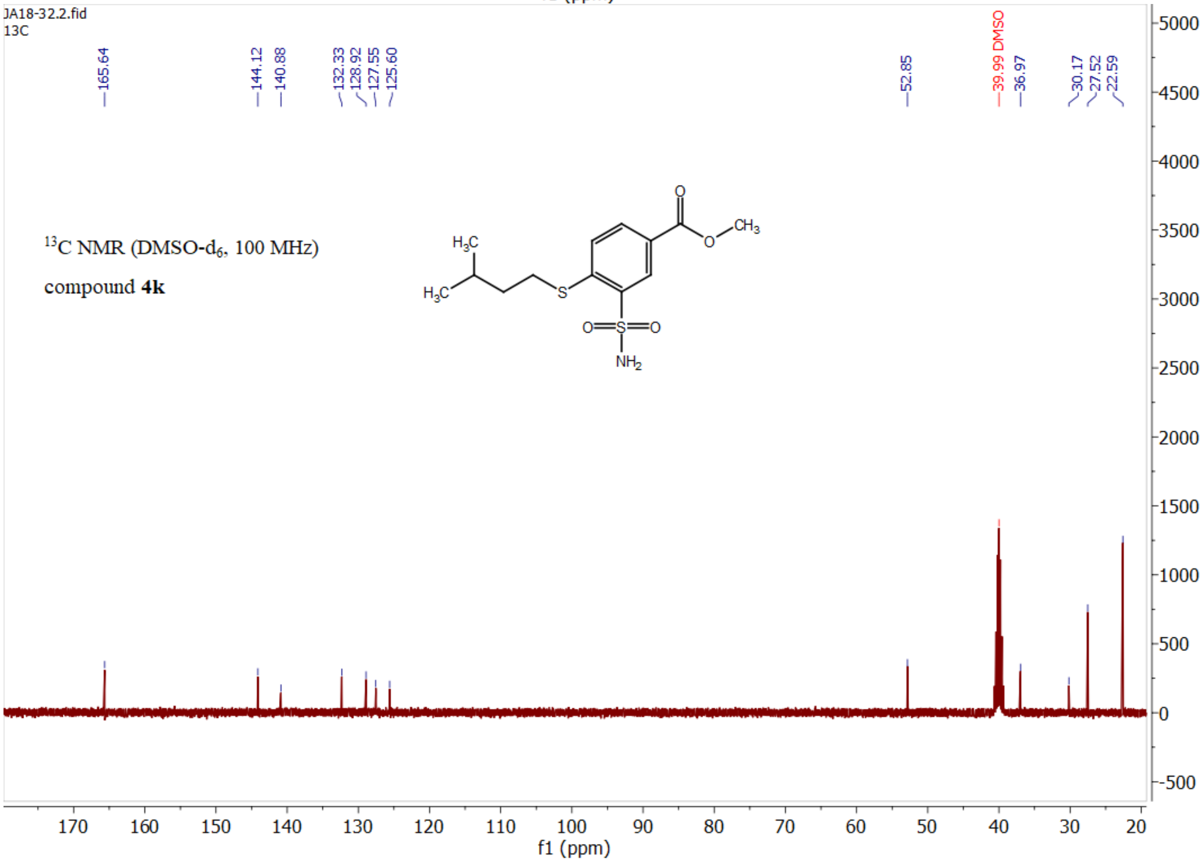
JA18-32.2.fid
13C
165.64
144.12
140.88
132.33
128.92
127.55
125.60
52.85
39.99 DMSO
36.97
30.17
27.52
22.59
^{13}C NMR (DMSO-d6, 100 MHz)
compound 4k
f1 (ppm)

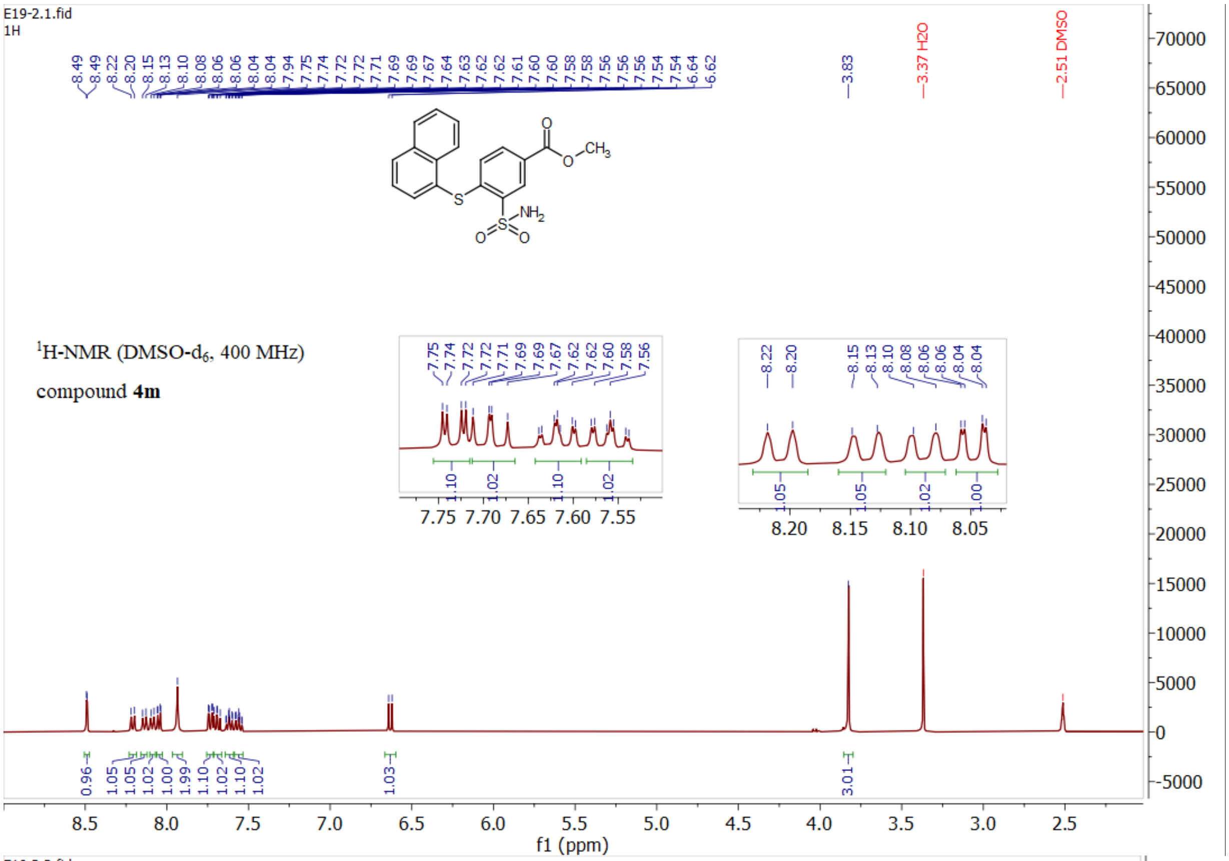
E19-2.1.fid
1H
3.37 H2O
2.51 DMSO
1H-NMR (DMSO-d6, 400 MHz)
compound 4m
f1 (ppm)

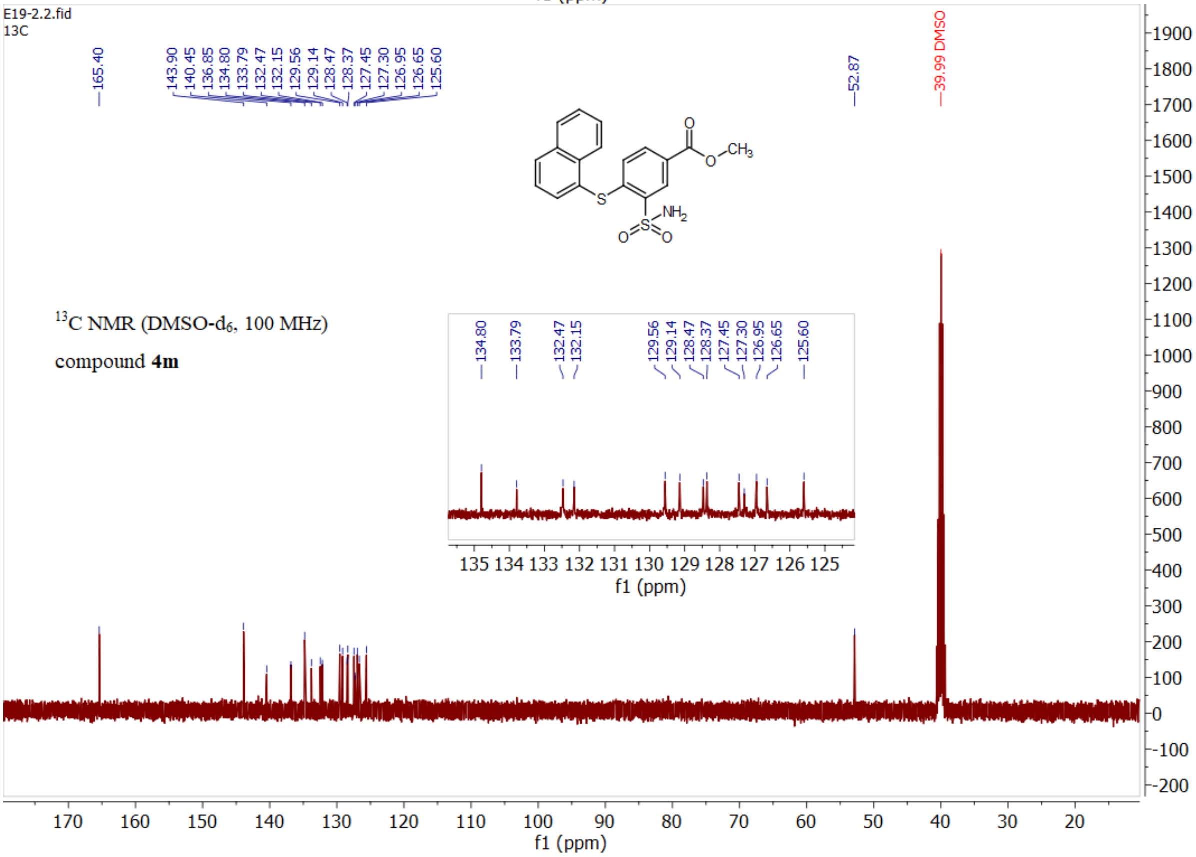
E19-2.2.fid
13C
39.99 DMSO
13C NMR (DMSO-d6, 100 MHz)
compound 4m
f1 (ppm)

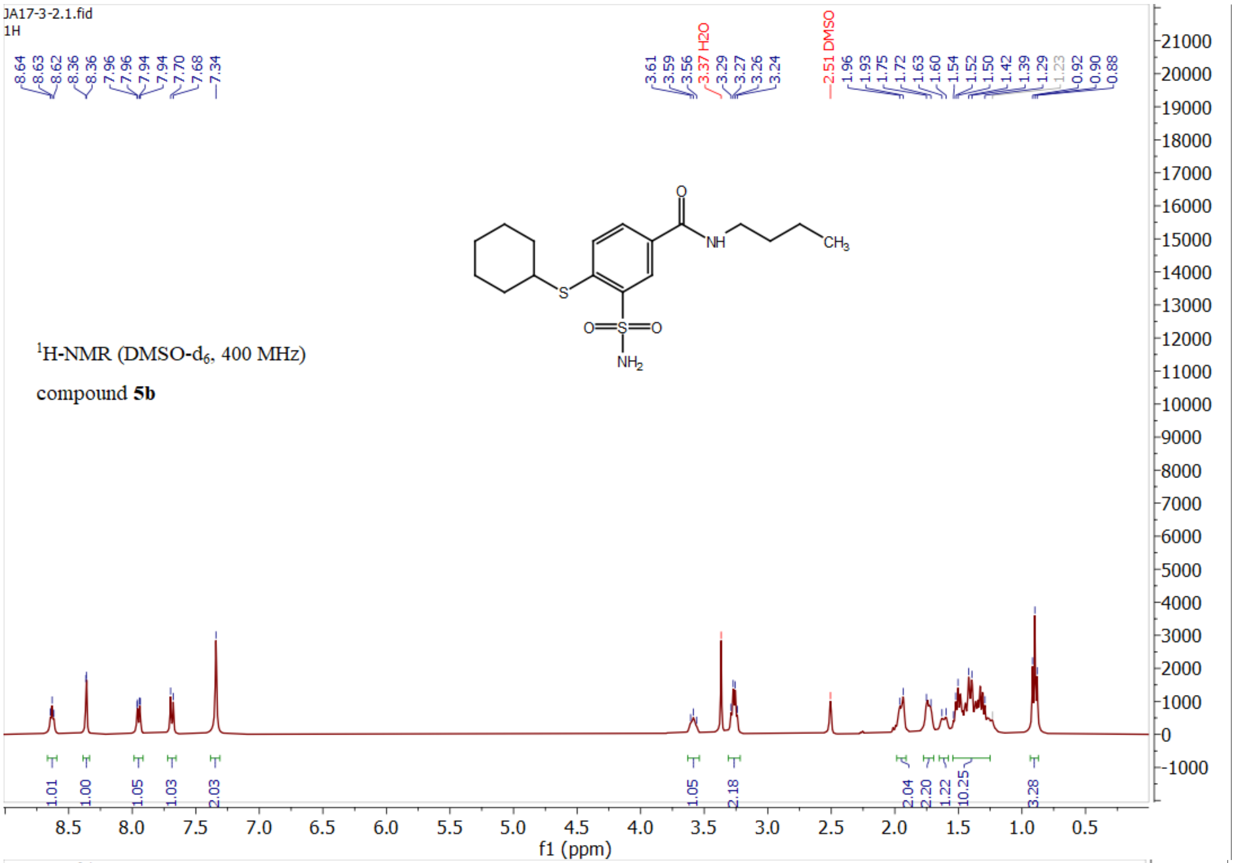
JA17-3-2.1.fid
1H
8.64
8.63
8.62
8.36
8.36
7.96
7.96
7.94
7.94
7.70
7.68
7.34
3.61
3.59
3.56
3.37 H2O
3.29
3.27
3.26
3.24
2.51 DMSO
1.96
1.93
1.75
1.72
1.63
1.60
1.54
1.52
1.50
1.42
1.39
1.29
1.23
0.92
0.90
0.88
O
NH
CH3
S
O=S=O
NH2
1H-NMR (DMSO-d6, 400 MHz)
compound 5b
1.01
1.00
1.05
1.03
2.03
1.05
2.18
2.04
2.20
1.22
10.25
3.28
f1 (ppm)
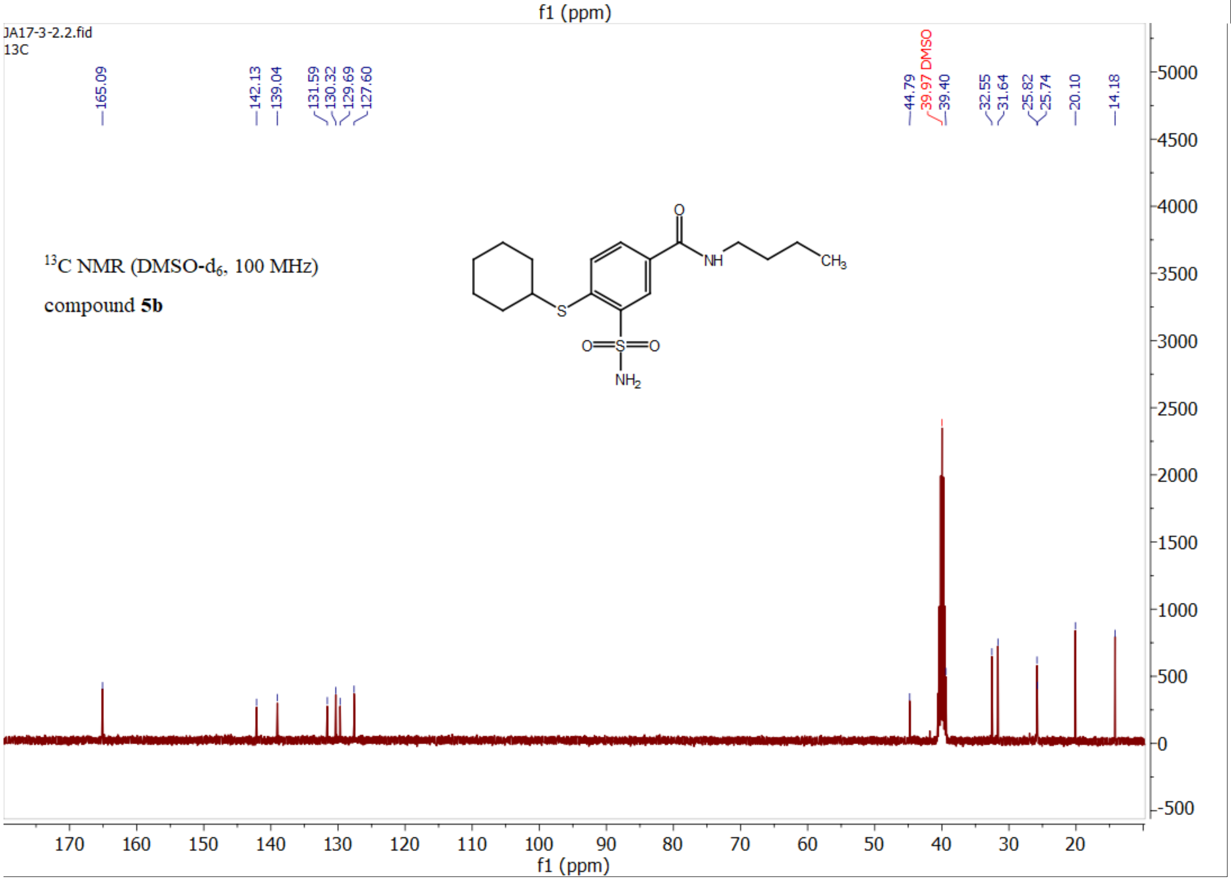
JA17-3-2.2.fid
13C
165.09
142.13
139.04
131.59
130.32
129.69
127.60
44.79
39.97 DMSO
39.40
32.55
31.64
25.82
25.74
20.10
14.18
13C NMR (DMSO-d6, 100 MHz)
compound 5b
O
NH
CH3
S
O=S=O
NH2
f1 (ppm)

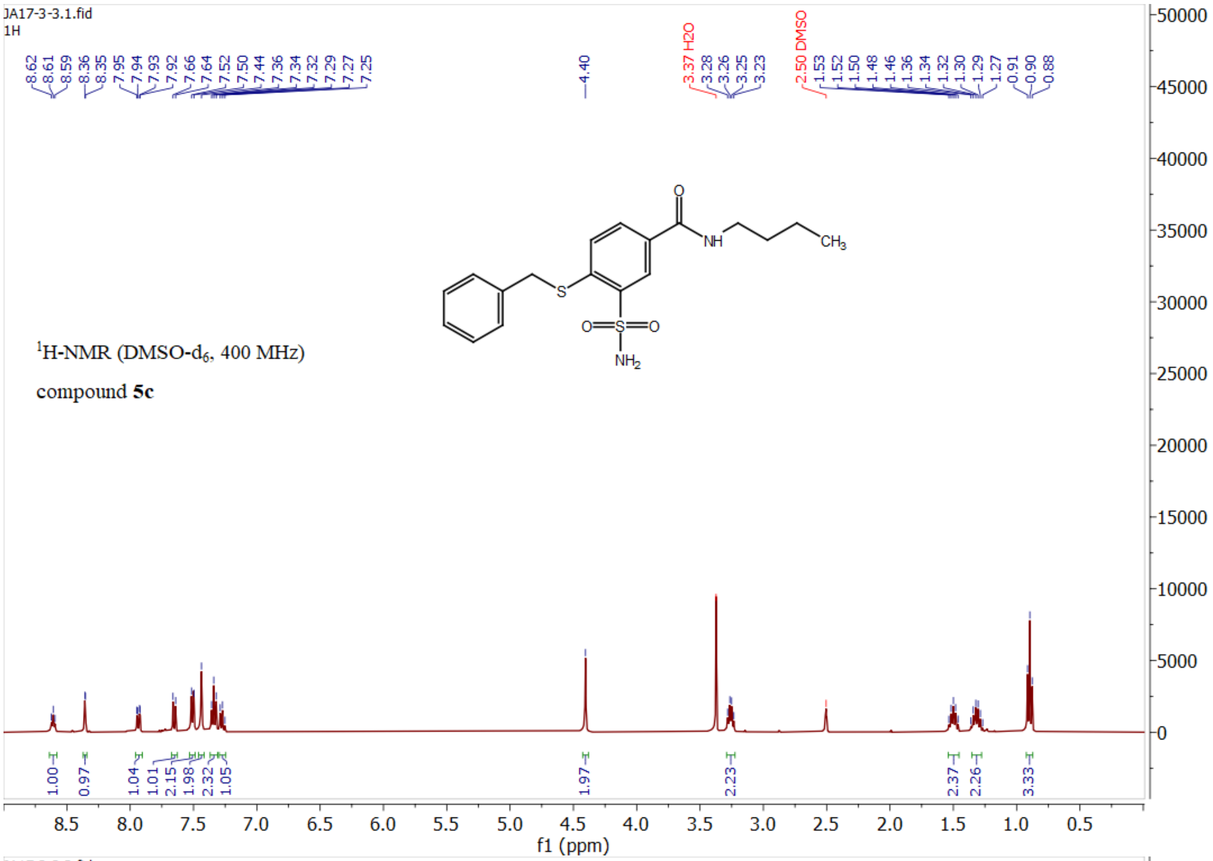
JA17-3-3.1.fid
1H
^{1}H-NMR (DMSO-d$_6$, 400 MHz)
compound **5c**
NH
CH_3
S
O=S=O
NH_2
f1 (ppm)

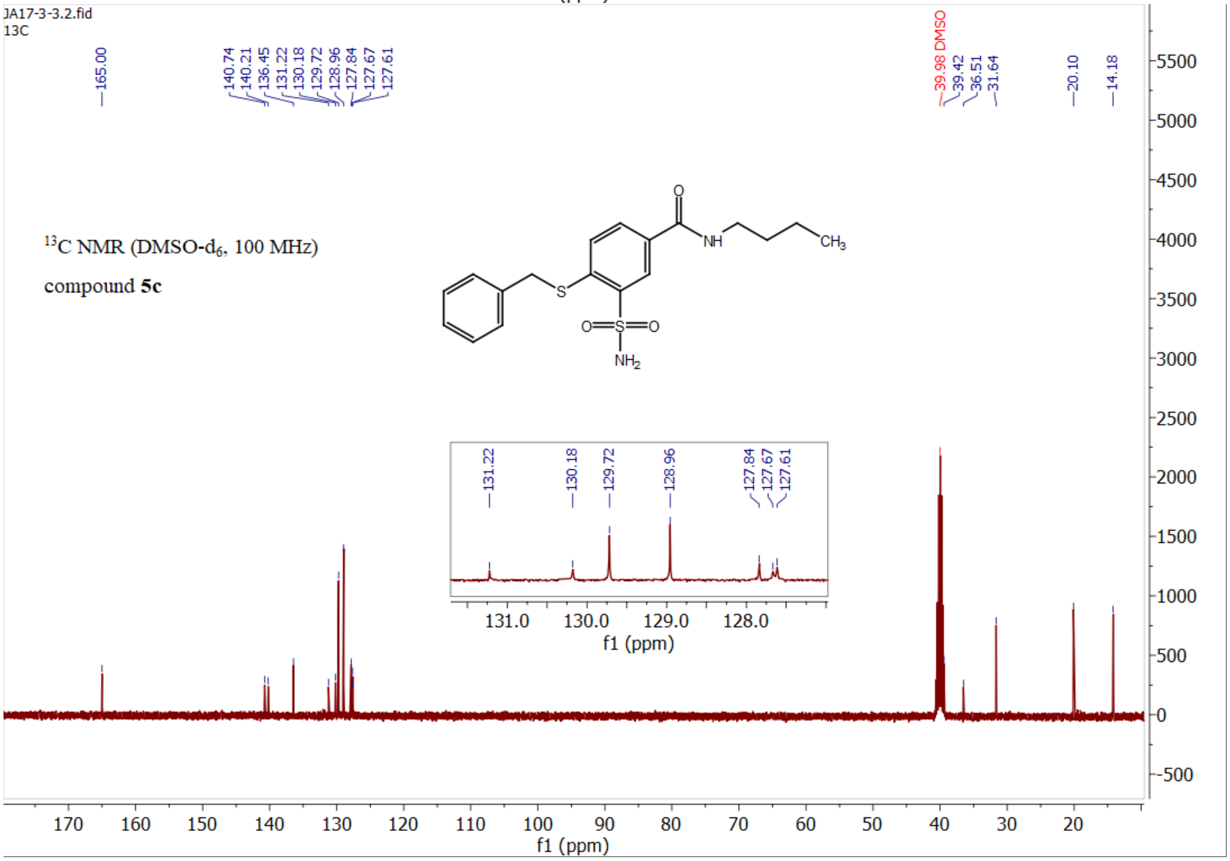
JA17-3-3.2.fid
13C
^{13}C NMR (DMSO-d$_6$, 100 MHz)
compound **5c**
NH
CH_3
S
O=S=O
NH_2
f1 (ppm)

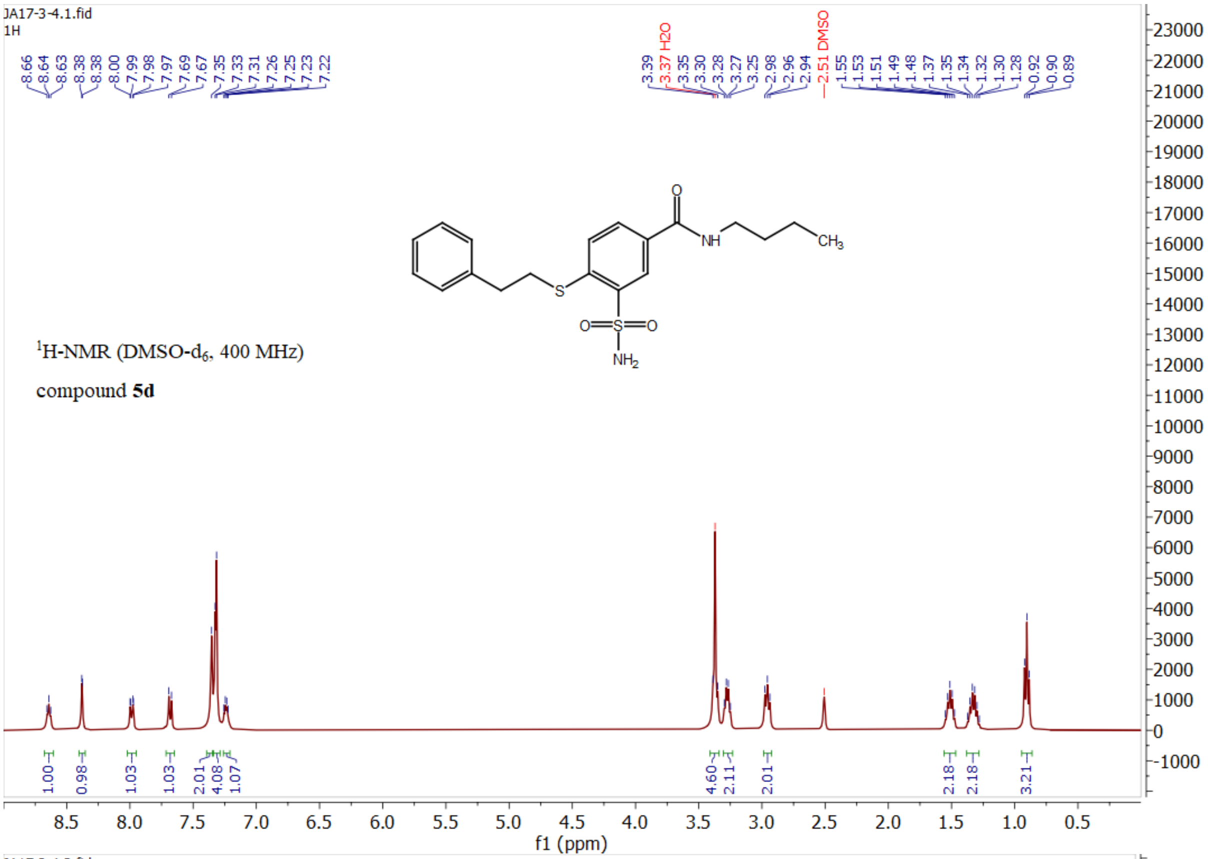
JA17-3-4.1.fid
1H
8.66 8.64 8.63 8.38 8.38 8.00 7.99 7.98 7.97 7.69 7.67 7.35 7.33 7.31 7.26 7.25 7.23 7.22
3.39 3.37 H2O 3.35 3.30 3.28 3.27 3.25 2.98 2.96 2.94 2.51 DMSO 1.55 1.53 1.51 1.49 1.48 1.37 1.35 1.34 1.32 1.30 1.28 0.92 0.90 0.89
¹H-NMR (DMSO-d6, 400 MHz)
compound 5d
1.00 0.98 1.03 1.03 2.01 4.08 1.07 4.60 2.11 2.01 2.18 2.18 3.21
f1 (ppm)

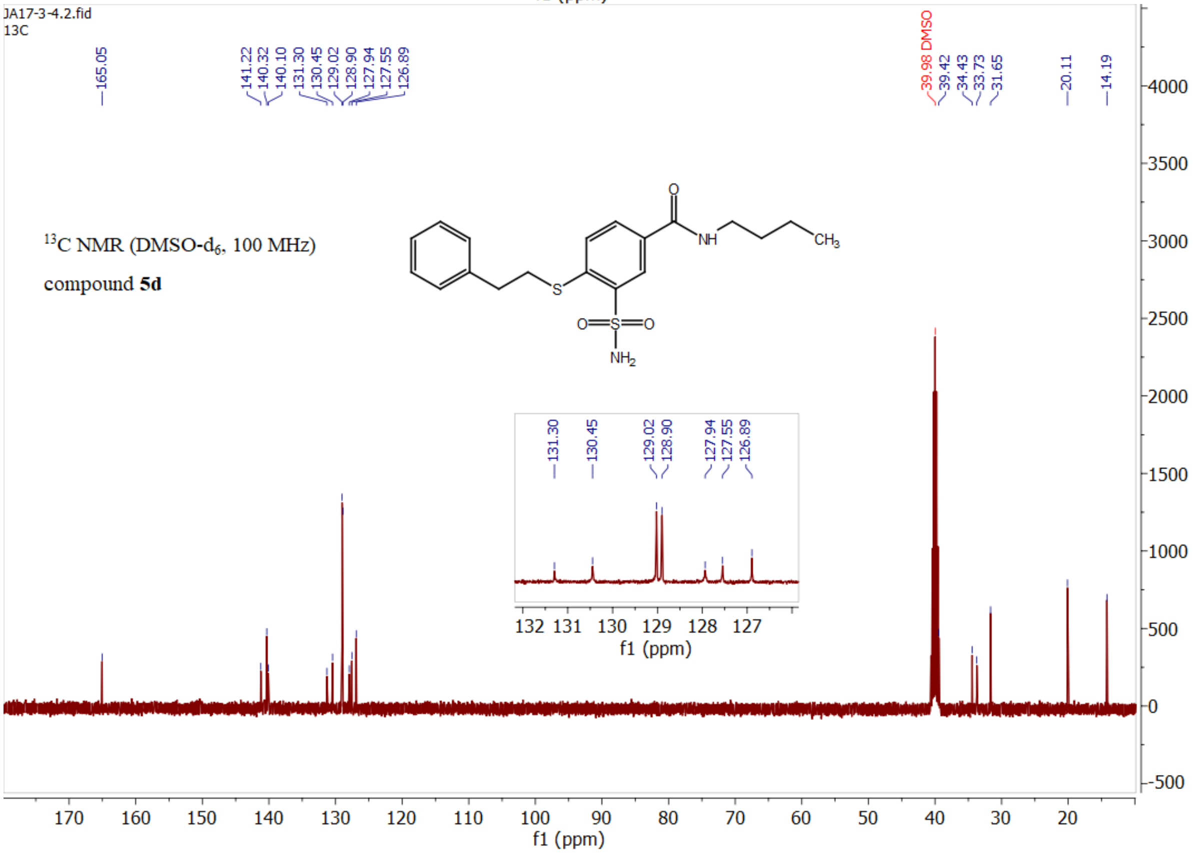
JA17-3-4.2.fid
13C
165.05
141.22 140.32 140.10 131.30 130.45 129.02 128.90 127.94 127.55 126.89
39.98 DMSO 39.42 34.43 33.73 31.65 20.11 14.19
¹³C NMR (DMSO-d6, 100 MHz)
compound 5d
f1 (ppm)

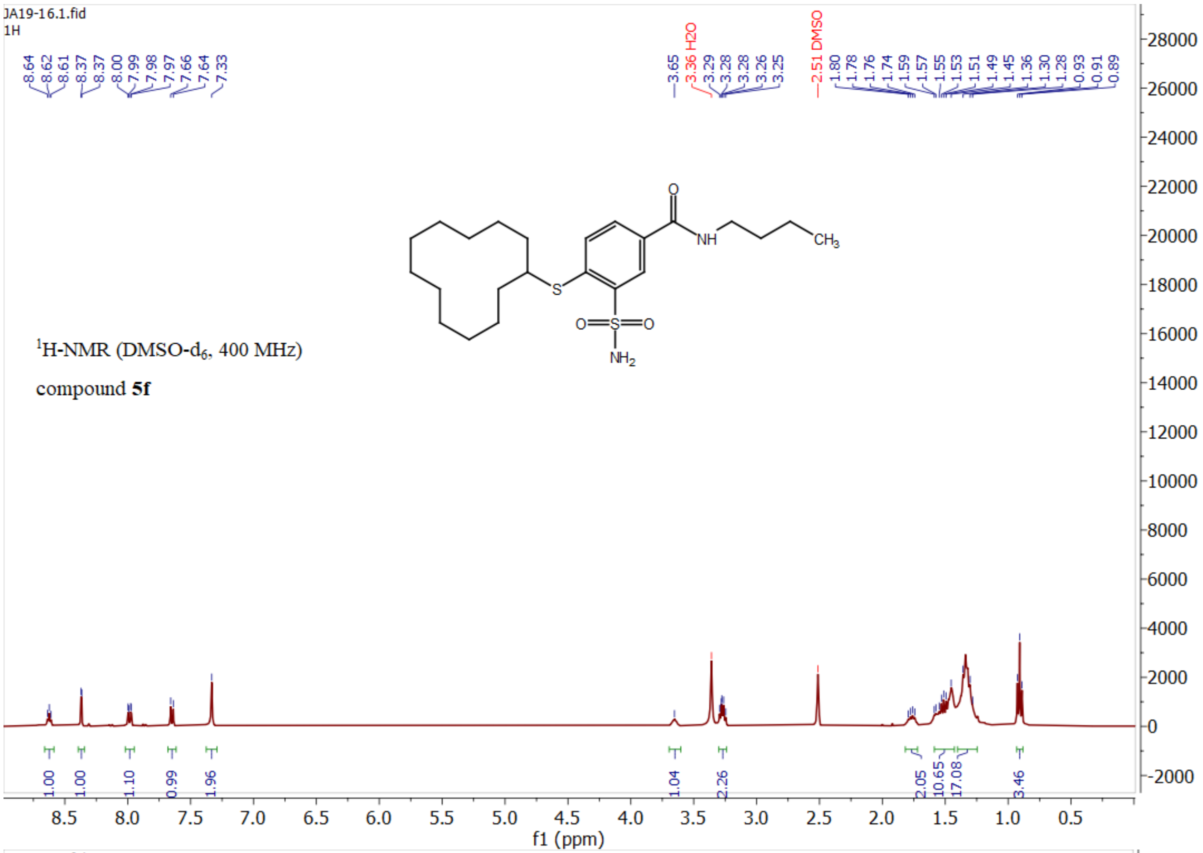
JA19-16.1.fid
1H
8.64 8.62 8.61 8.37 8.37 8.00 7.99 7.98 7.97 7.66 7.64 7.33
3.65 3.36 H2O 3.29 3.28 3.28 3.26 3.25
2.51 DMSO 1.80 1.78 1.76 1.74 1.59 1.57 1.55 1.53 1.51 1.49 1.45 1.36 1.30 1.28 0.93 0.91 0.89
^{1}H-NMR (DMSO-d$_6$, 400 MHz)
compound **5f**
1.00 1.00 1.10 0.99 1.96 1.04 2.26 2.05 10.65 17.08 3.46
f1 (ppm)

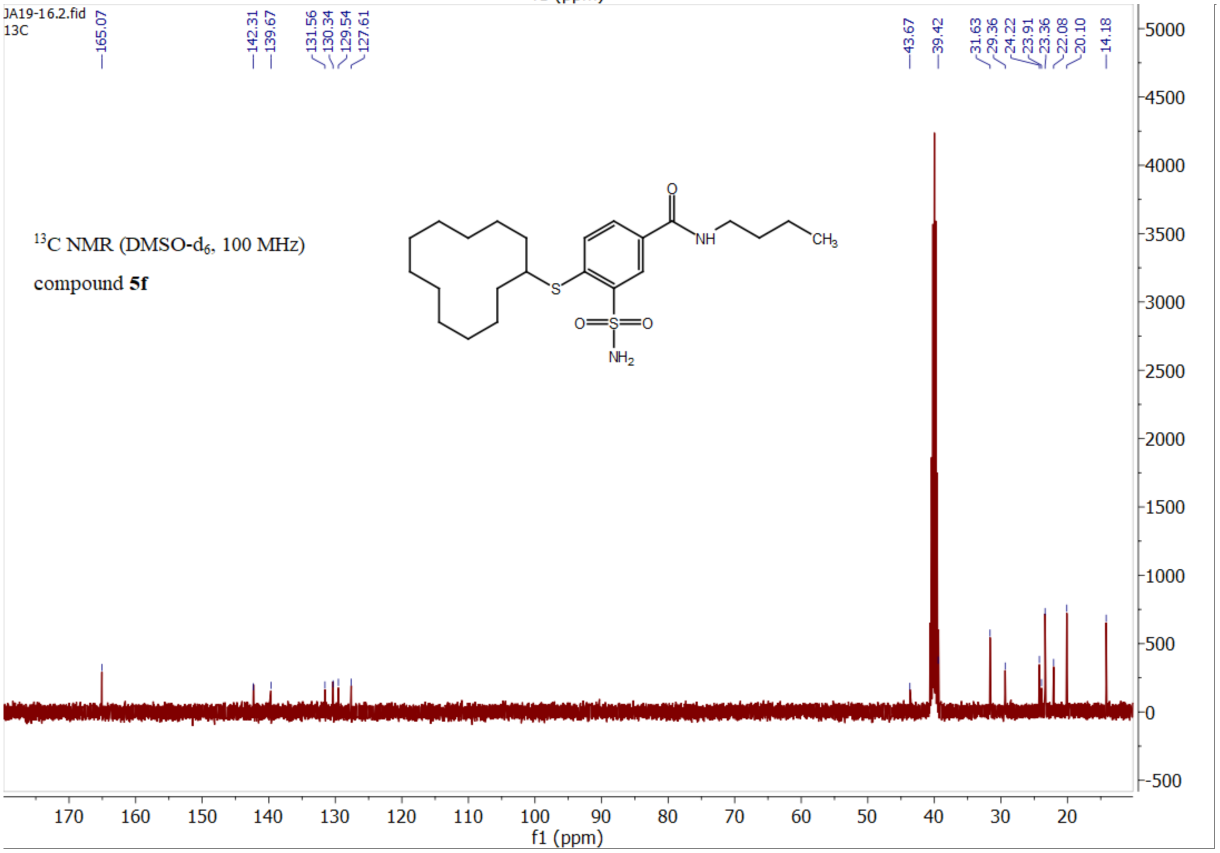
JA19-16.2.fid
13C
165.07
142.31 139.67
131.56 130.34 129.54 127.61
43.67 39.42 31.63 29.36 24.22 23.91 23.36 22.08 20.10 14.18
^{13}C NMR (DMSO-d$_6$, 100 MHz)
compound **5f**
f1 (ppm)

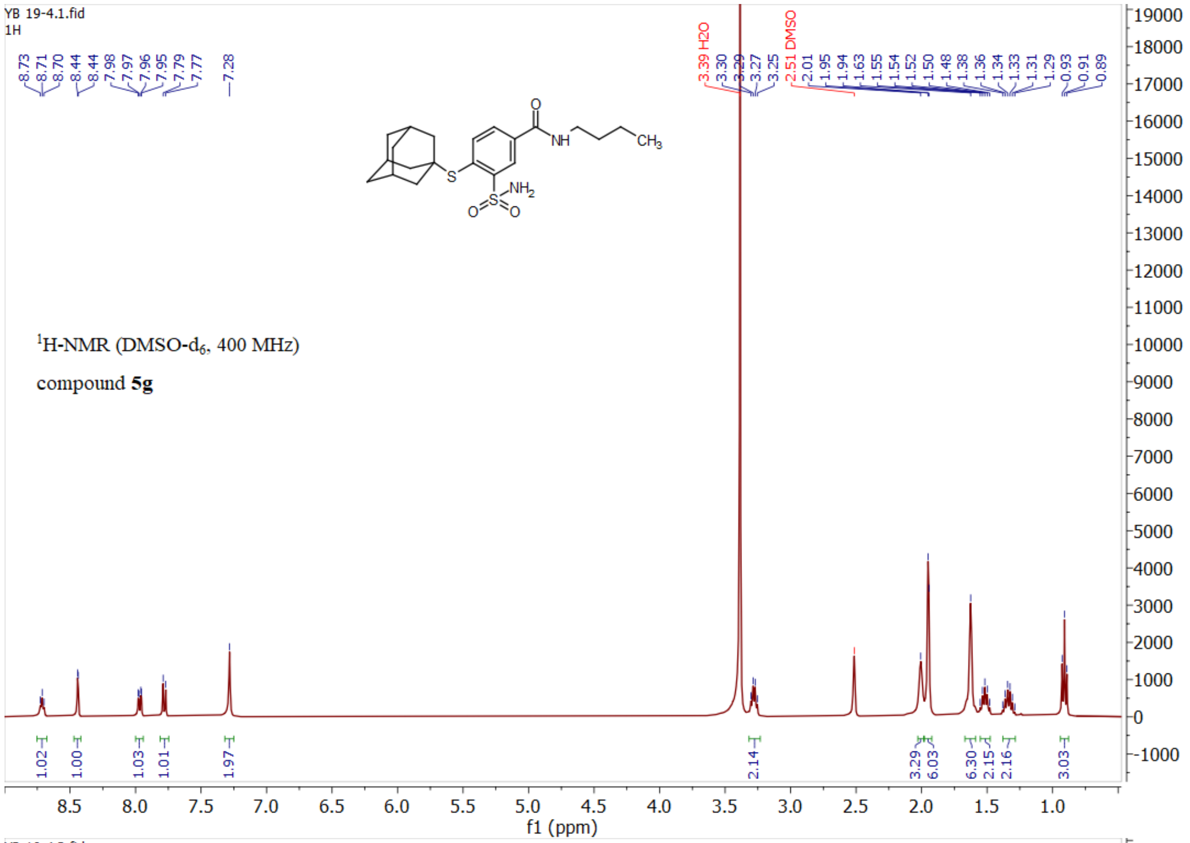
YB 19-4.1.fid
1H
^{1}H-NMR (DMSO-d$_6$, 400 MHz)
compound **5g**
f1 (ppm)

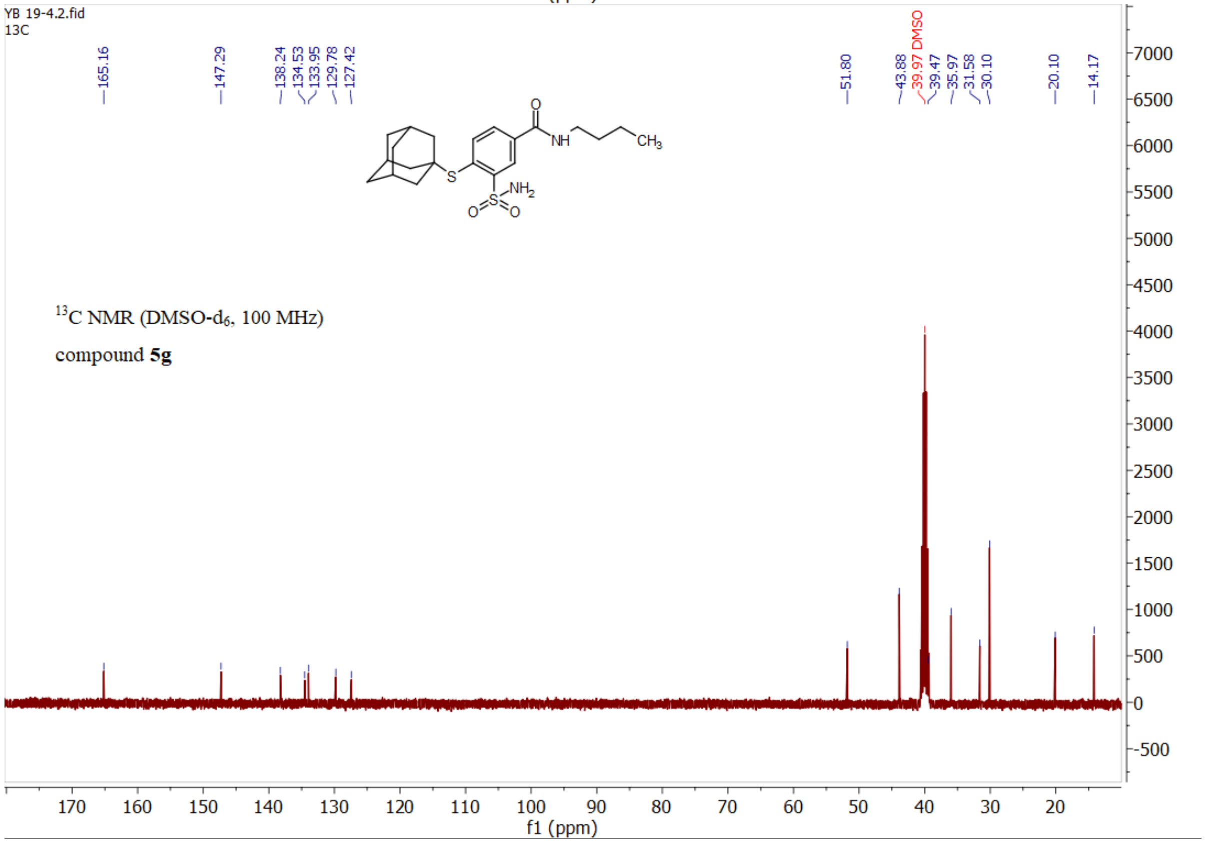
YB 19-4.2.fid
13C
^{13}C NMR (DMSO-d$_6$, 100 MHz)
compound **5g**
f1 (ppm)

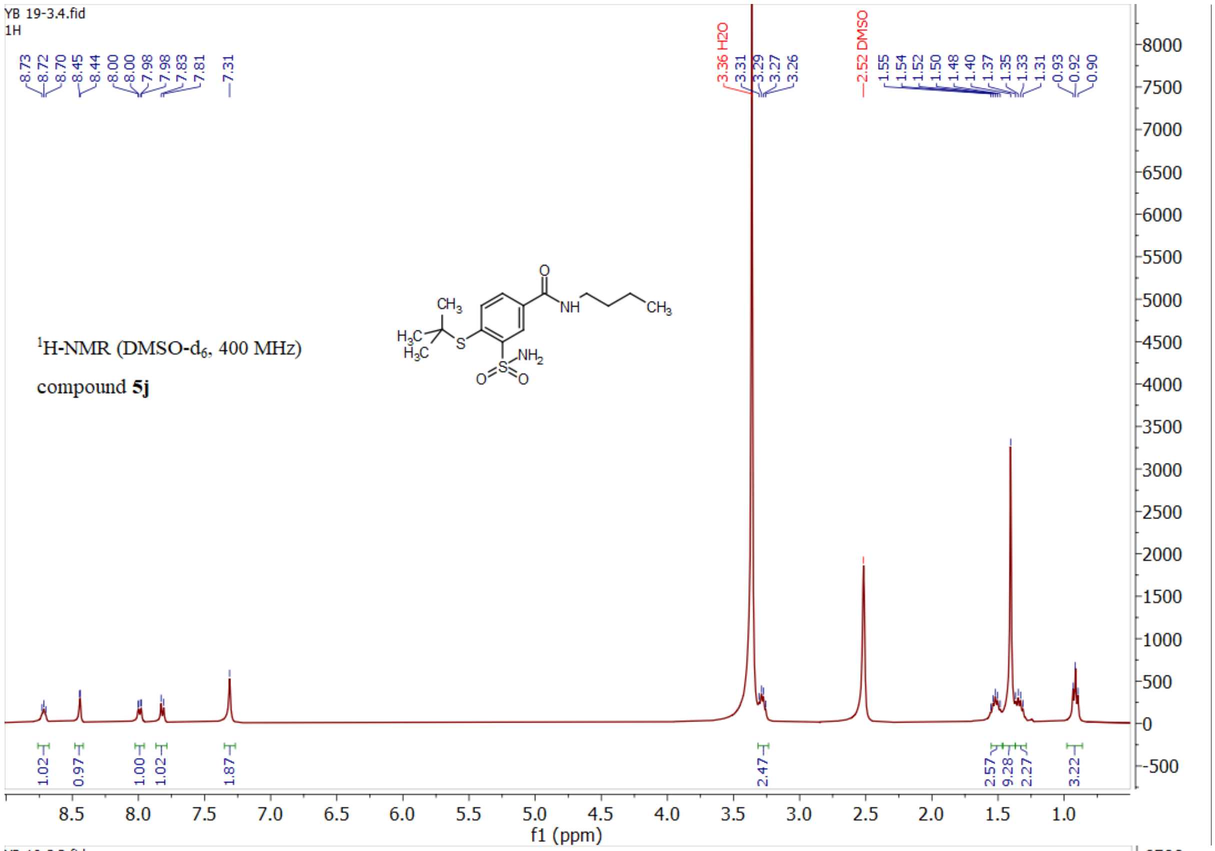

YB 19-3.4.fid
1H
^{1}H-NMR (DMSO-d$_6$, 400 MHz)
compound **5j**
f1 (ppm)

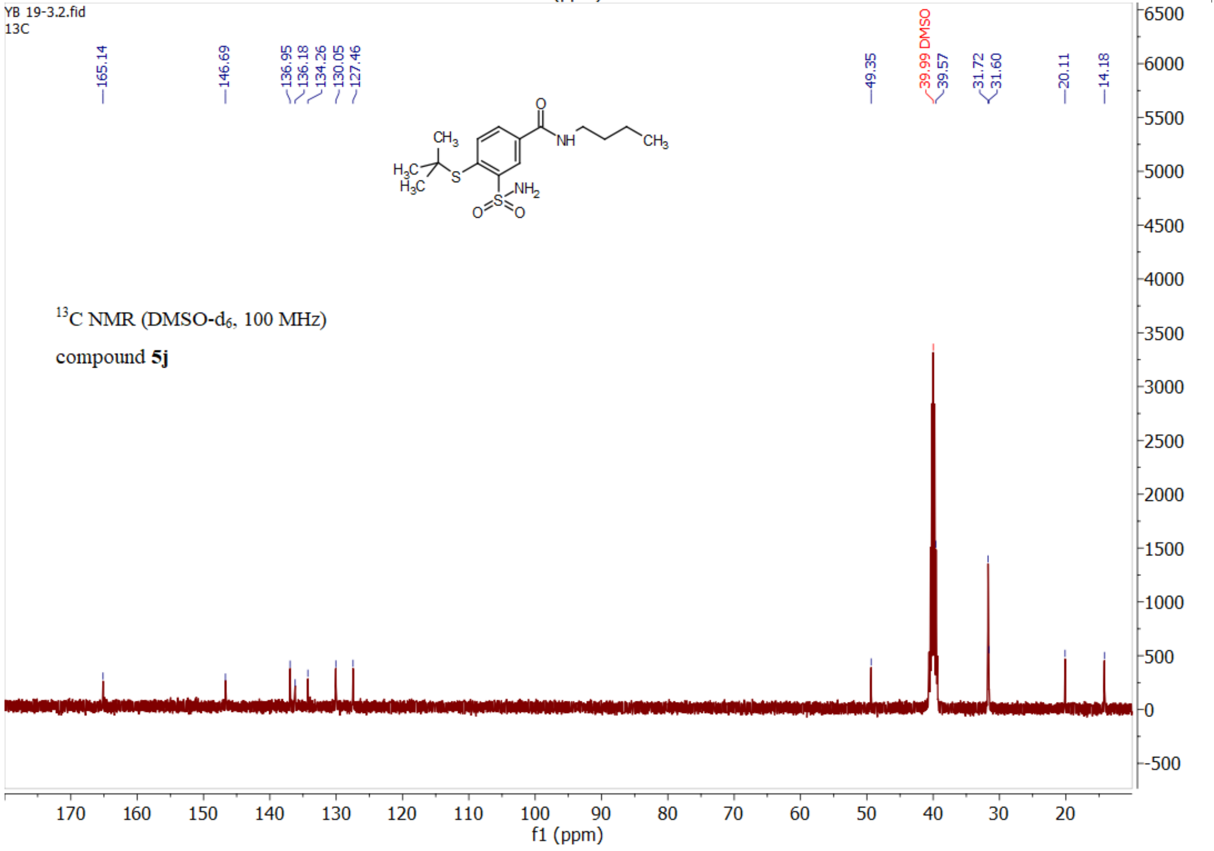

YB 19-3.2.fid
13C
^{13}C NMR (DMSO-d$_6$, 100 MHz)
compound **5j**
f1 (ppm)

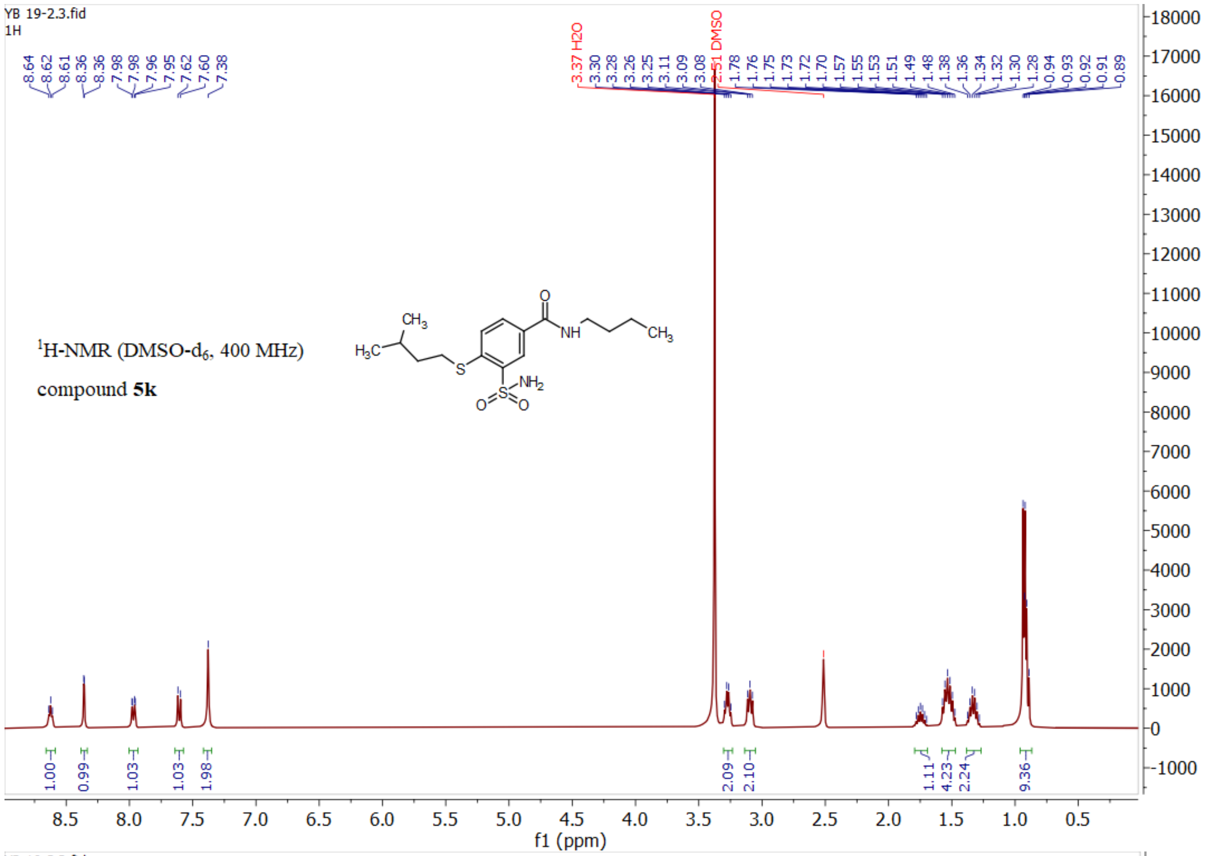

YB 19-2.3.fid
1H
^{1}H-NMR (DMSO-d$_6$, 400 MHz)
compound **5k**
f1 (ppm)

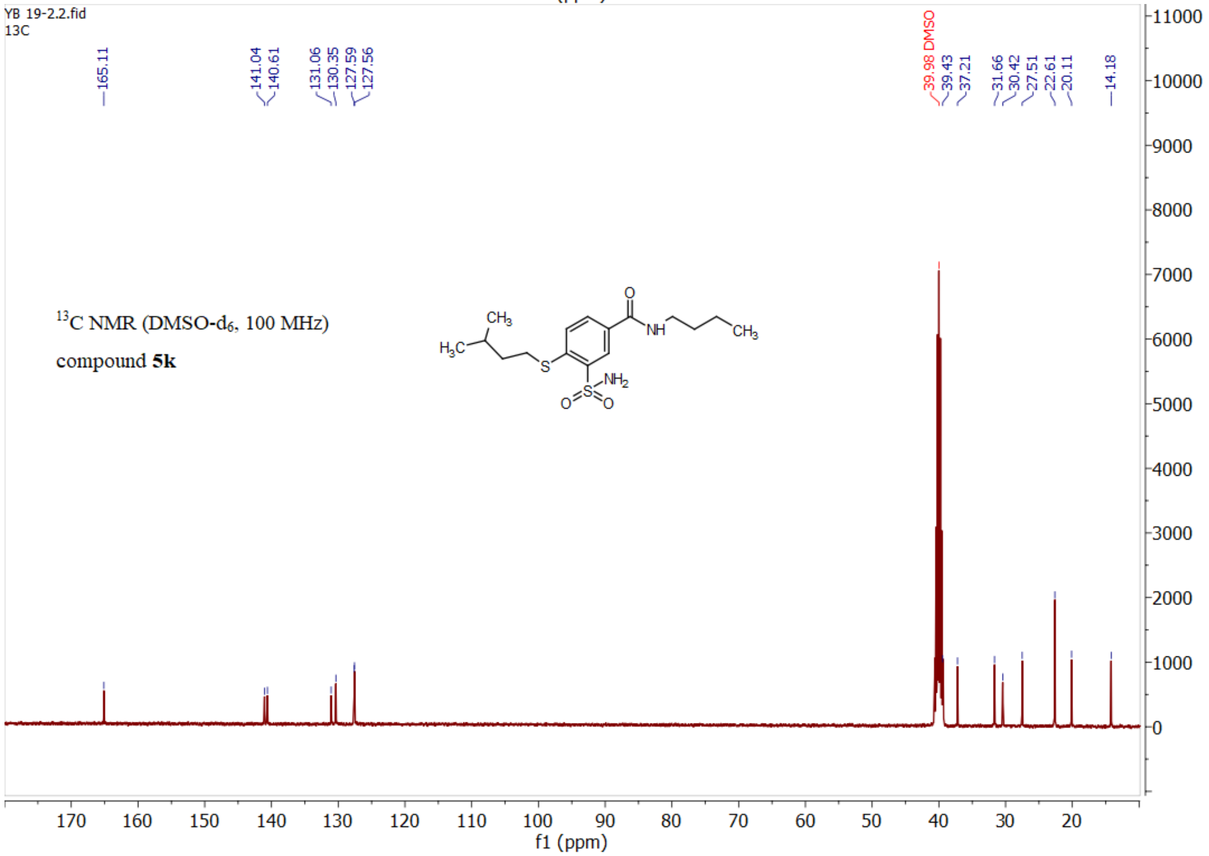

YB 19-2.2.fid
13C
^{13}C NMR (DMSO-d$_6$, 100 MHz)
compound **5k**
f1 (ppm)

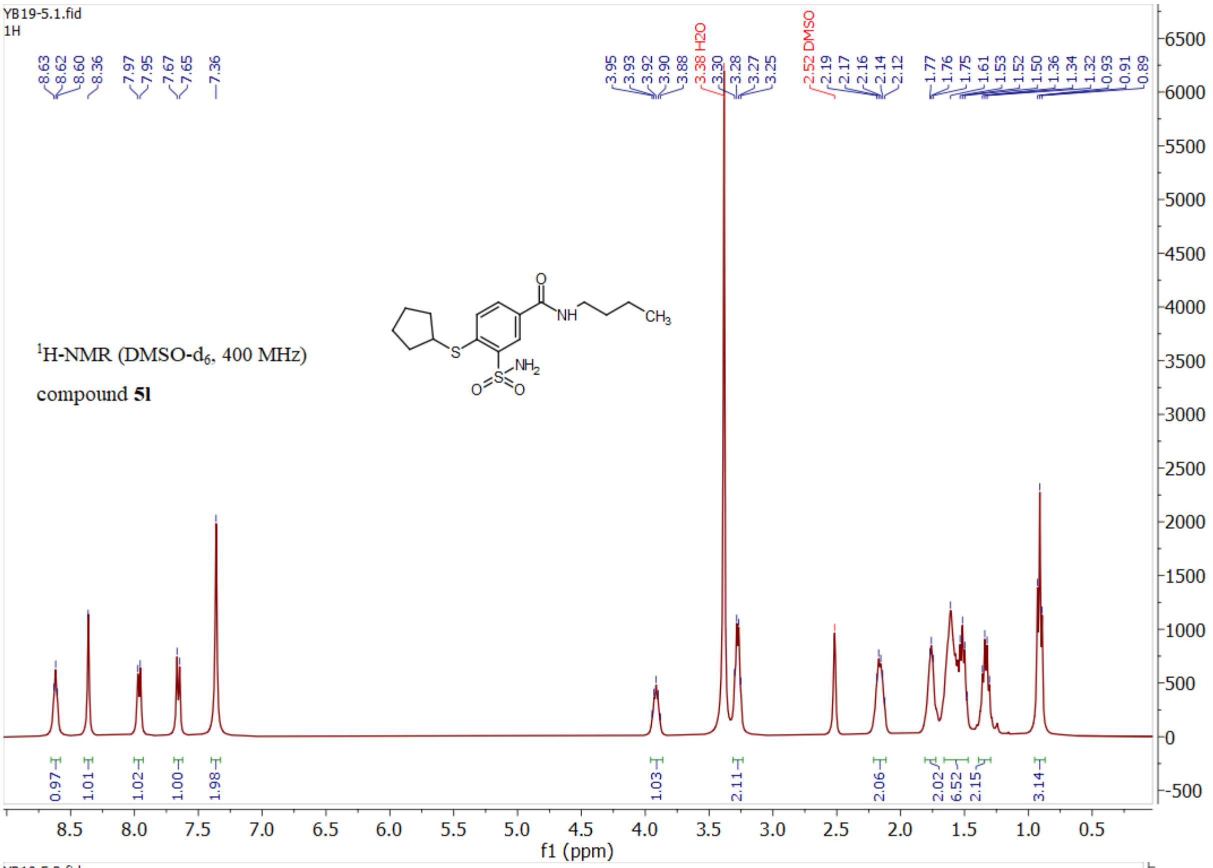
YB19-5.1.fid
1H
^{1}H-NMR (DMSO-d$_6$, 400 MHz)
compound **5l**
3.38 H2O
2.52 DMSO
f1 (ppm)

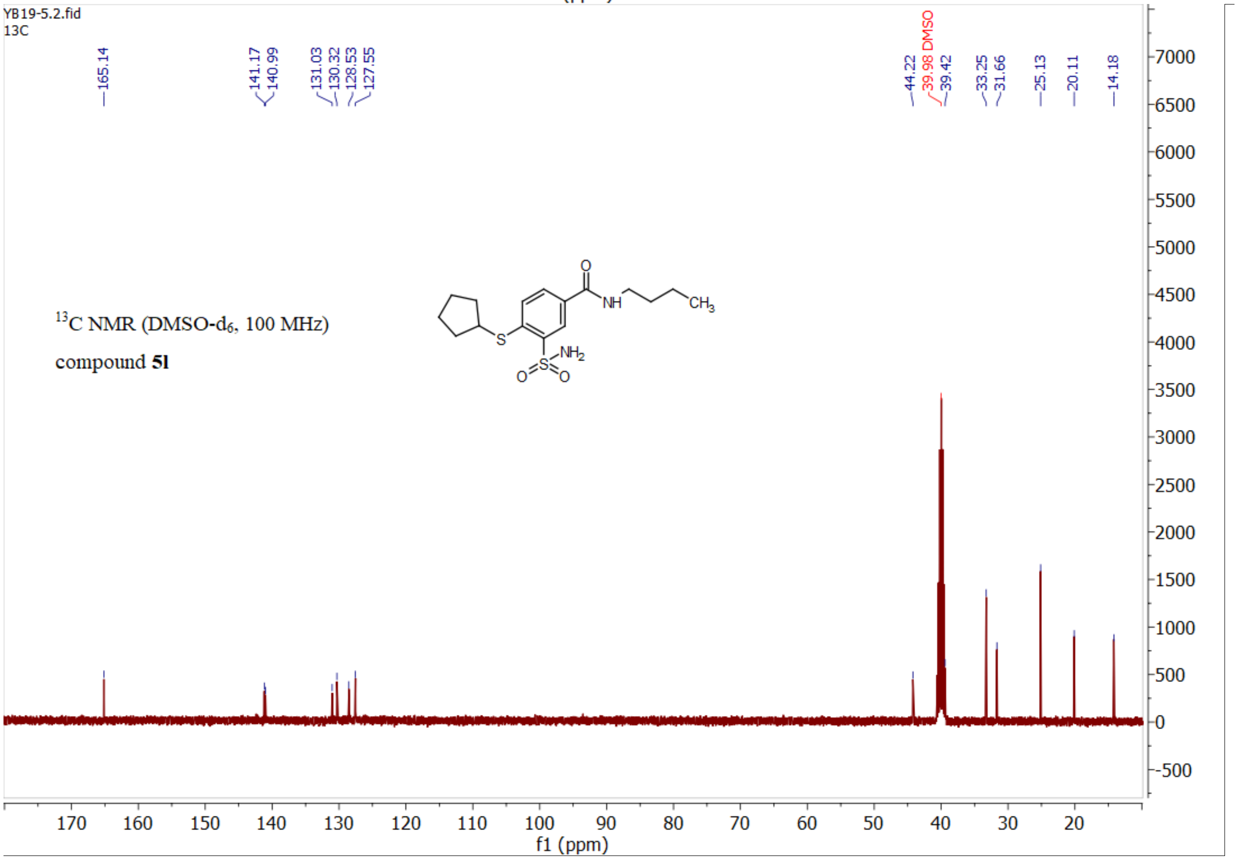
YB19-5.2.fid
13C
^{13}C NMR (DMSO-d$_6$, 100 MHz)
compound **5l**
39.98 DMSO
f1 (ppm)

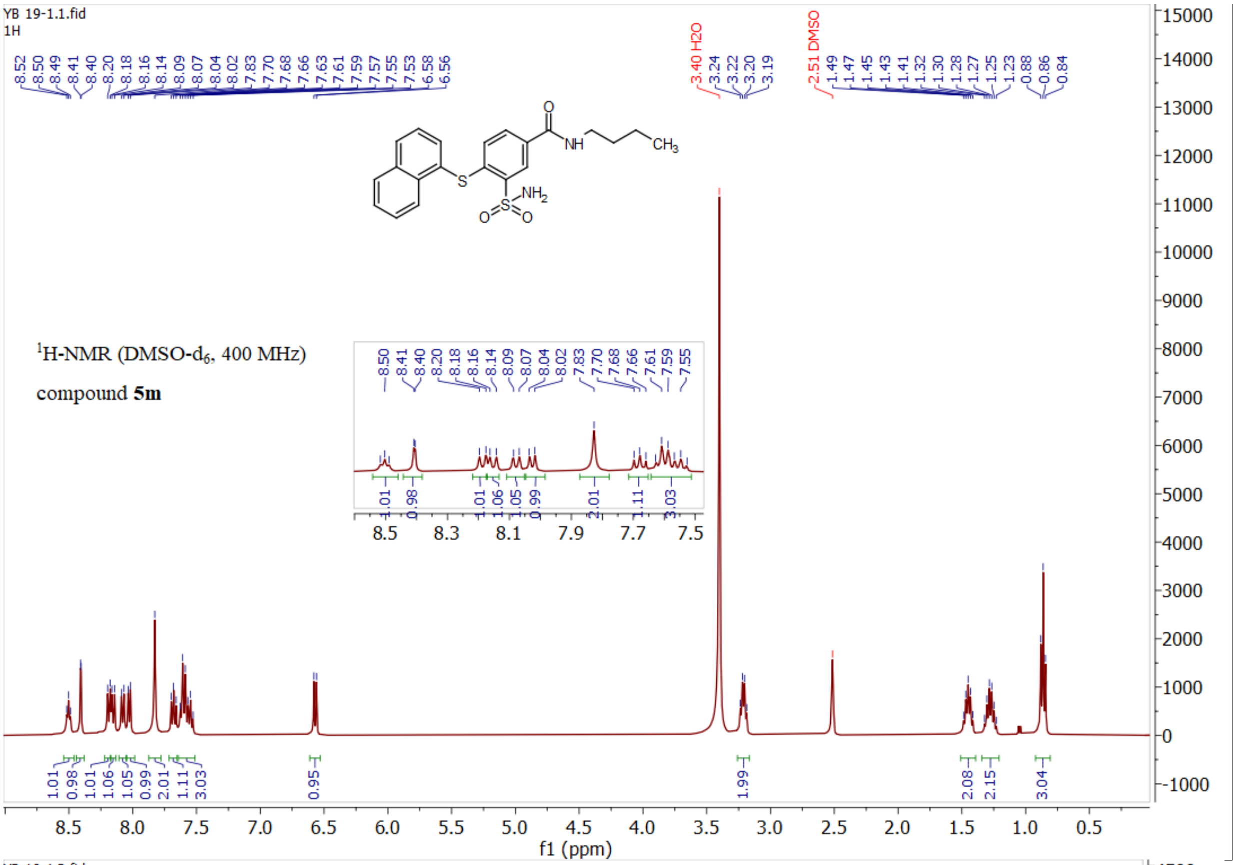
YB 19-1.1.fid
1H
^{1}H-NMR (DMSO-d6, 400 MHz)
compound 5m
3.40 H2O
2.51 DMSO
f1 (ppm)

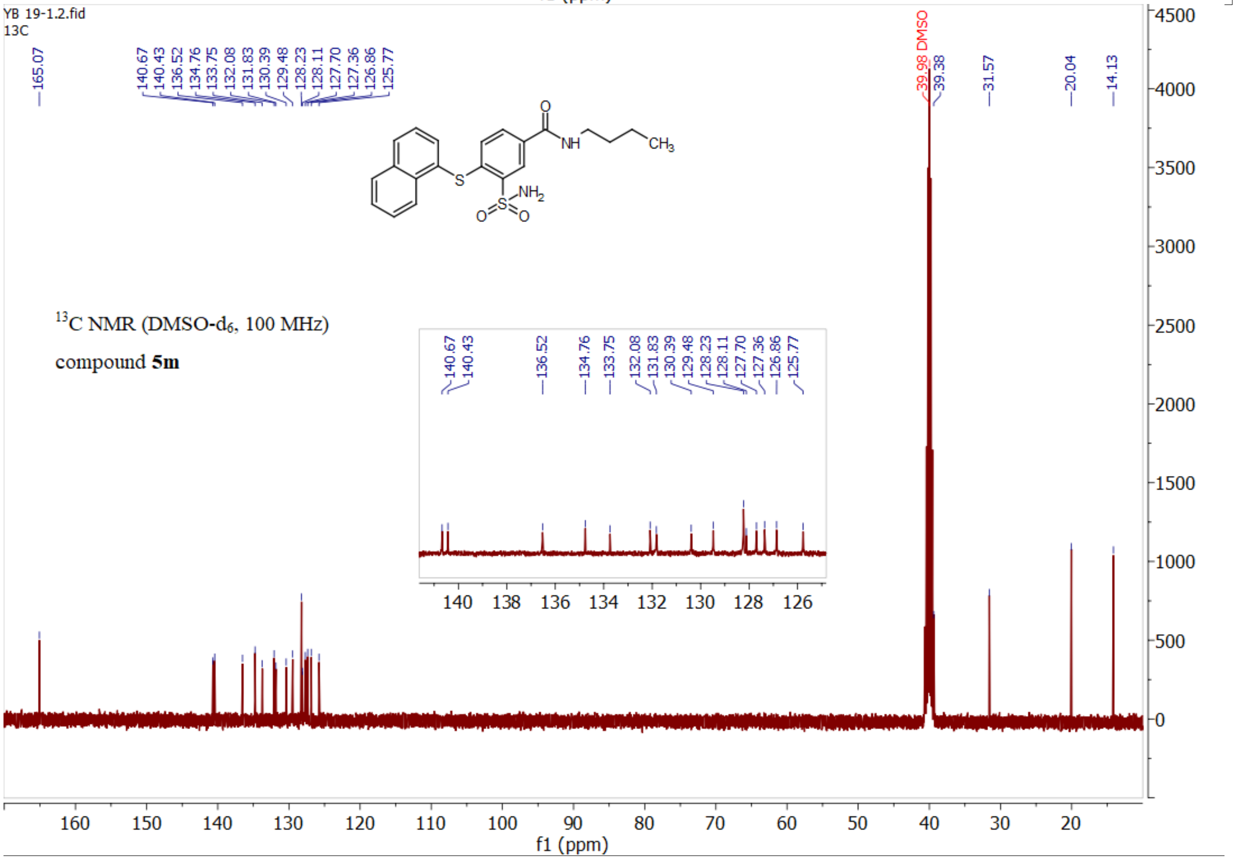
YB 19-1.2.fid
13C
^{13}C NMR (DMSO-d6, 100 MHz)
compound 5m
165.07
140.67
140.43
136.52
134.76
133.75
132.08
131.83
130.39
129.48
128.23
128.11
127.70
127.36
126.86
125.77
39.98 DMSO
39.38
31.57
20.04
14.13
f1 (ppm)

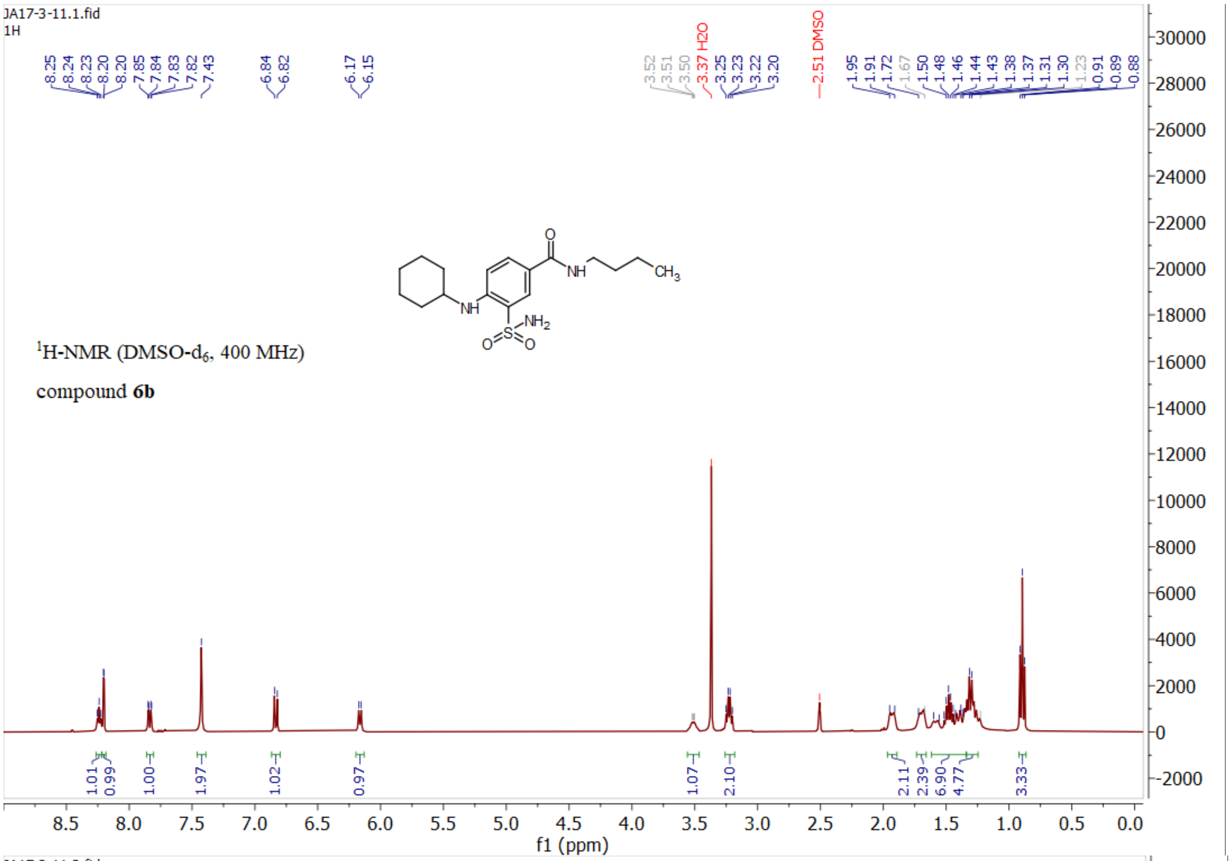
JA17-3-11.1.fid
1H
8.25
8.24
8.23
8.20
8.20
7.85
7.84
7.83
7.82
7.43
6.84
6.82
6.17
6.15
3.52
3.51
3.50
3.37 H2O
3.25
3.23
3.22
3.20
2.51 DMSO
1.95
1.91
1.72
1.67
1.50
1.48
1.46
1.44
1.43
1.38
1.37
1.31
1.30
1.23
0.91
0.89
0.88
O
NH
CH3
NH
NH2
O
S
O
1H-NMR (DMSO-d6, 400 MHz)
compound 6b
1.01
0.99
1.00
1.97
1.02
0.97
1.07
2.10
2.11
2.39
6.90
4.77
3.33
f1 (ppm)

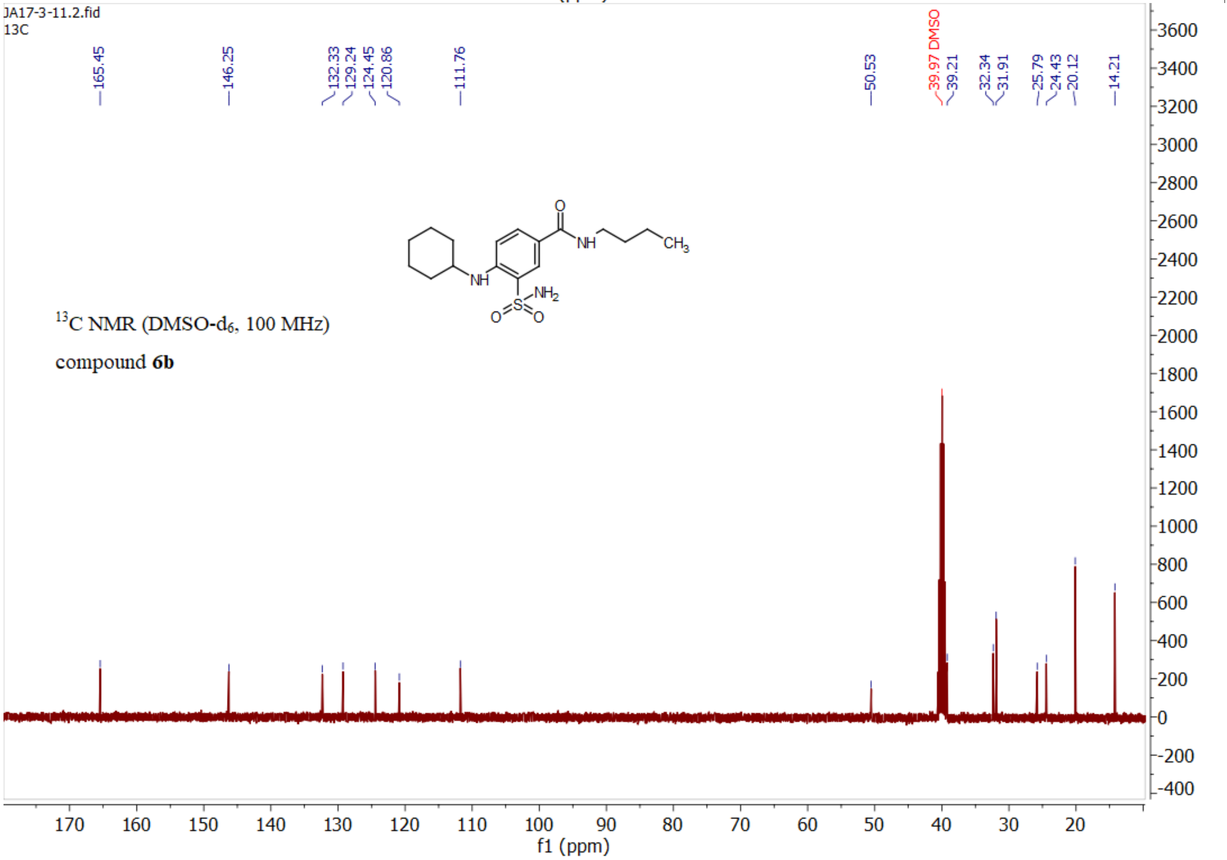
JA17-3-11.2.fid
13C
165.45
146.25
132.33
129.24
124.45
120.86
111.76
50.53
39.97 DMSO
39.21
32.34
31.91
25.79
24.43
20.12
14.21
O
NH
CH3
NH
NH2
O
S
O
13C NMR (DMSO-d6, 100 MHz)
compound 6b
f1 (ppm)

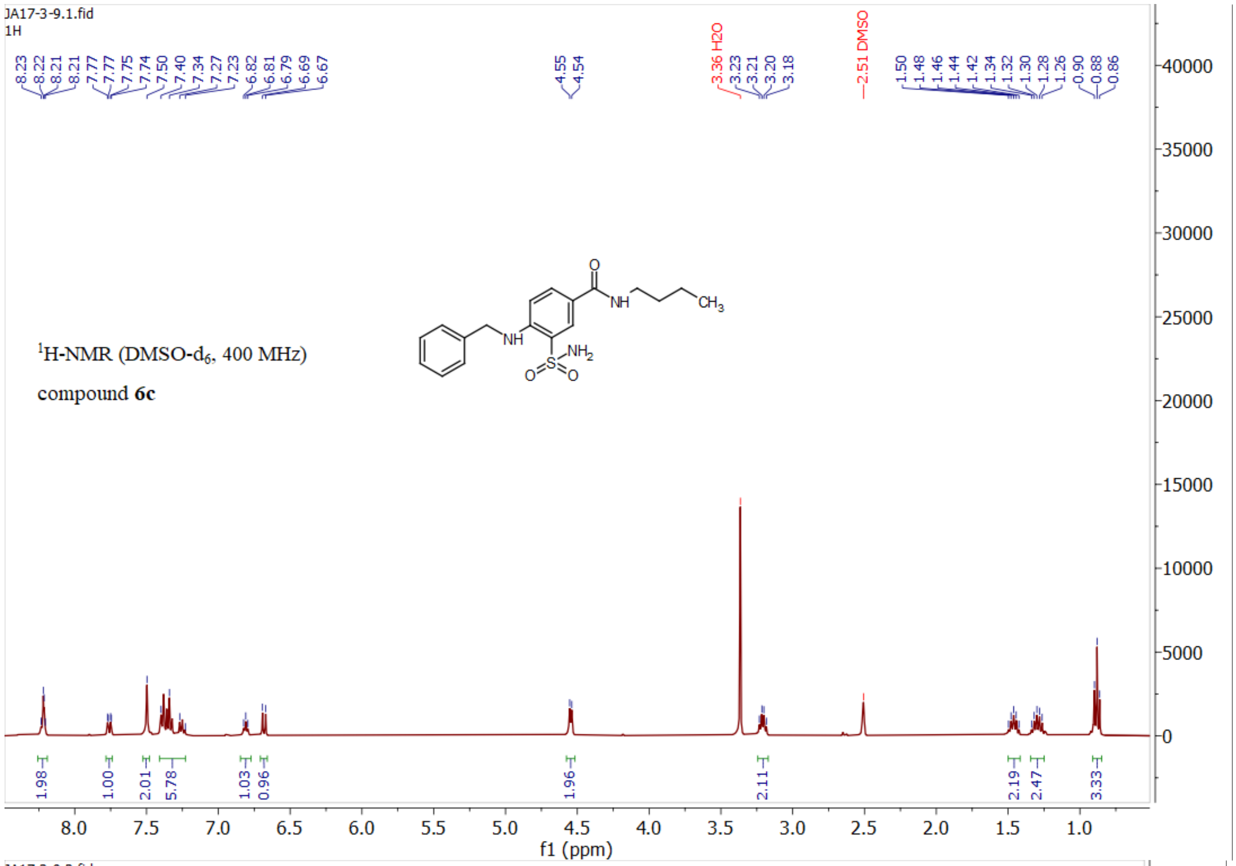
JA17-3-9.1.fid
1H
8.23 8.22 8.21 8.21 7.77 7.77 7.75 7.74 7.50 7.40 7.34 7.27 7.23 6.82 6.81 6.79 6.69 6.67
4.55 4.54
3.36 H2O
3.23 3.21 3.20 3.18
2.51 DMSO
1.50 1.48 1.46 1.44 1.42 1.34 1.32 1.30 1.28 1.26 0.90 0.88 0.86
1H-NMR (DMSO-d6, 400 MHz)
compound 6c
1.98 1.00 2.01 5.78 1.03 0.96 1.96 2.11 2.19 2.47 3.33
f1 (ppm)

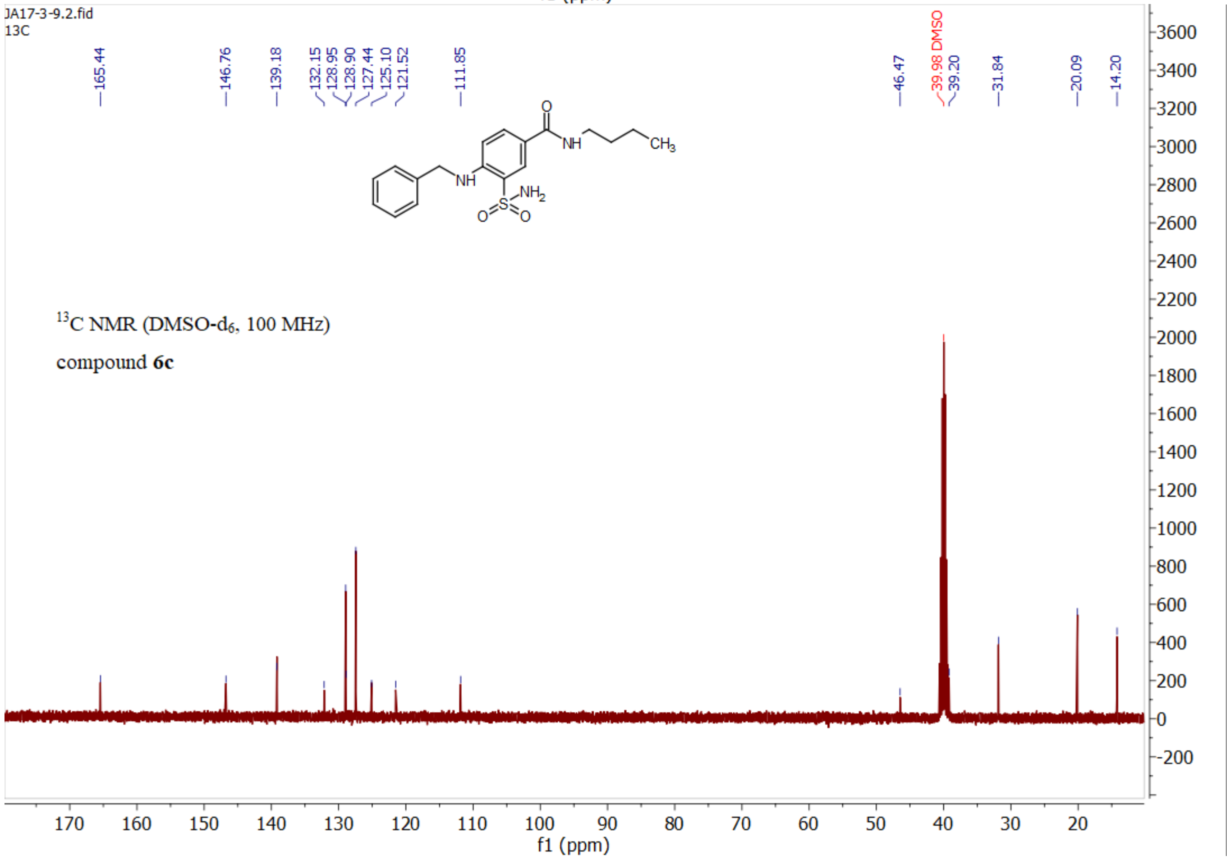
JA17-3-9.2.fid
13C
165.44
146.76
139.18
132.15 128.95 128.90 127.44 125.10 121.52
111.85
46.47
39.98 DMSO
39.20
31.84
20.09
14.20
13C NMR (DMSO-d6, 100 MHz)
compound 6c
f1 (ppm)

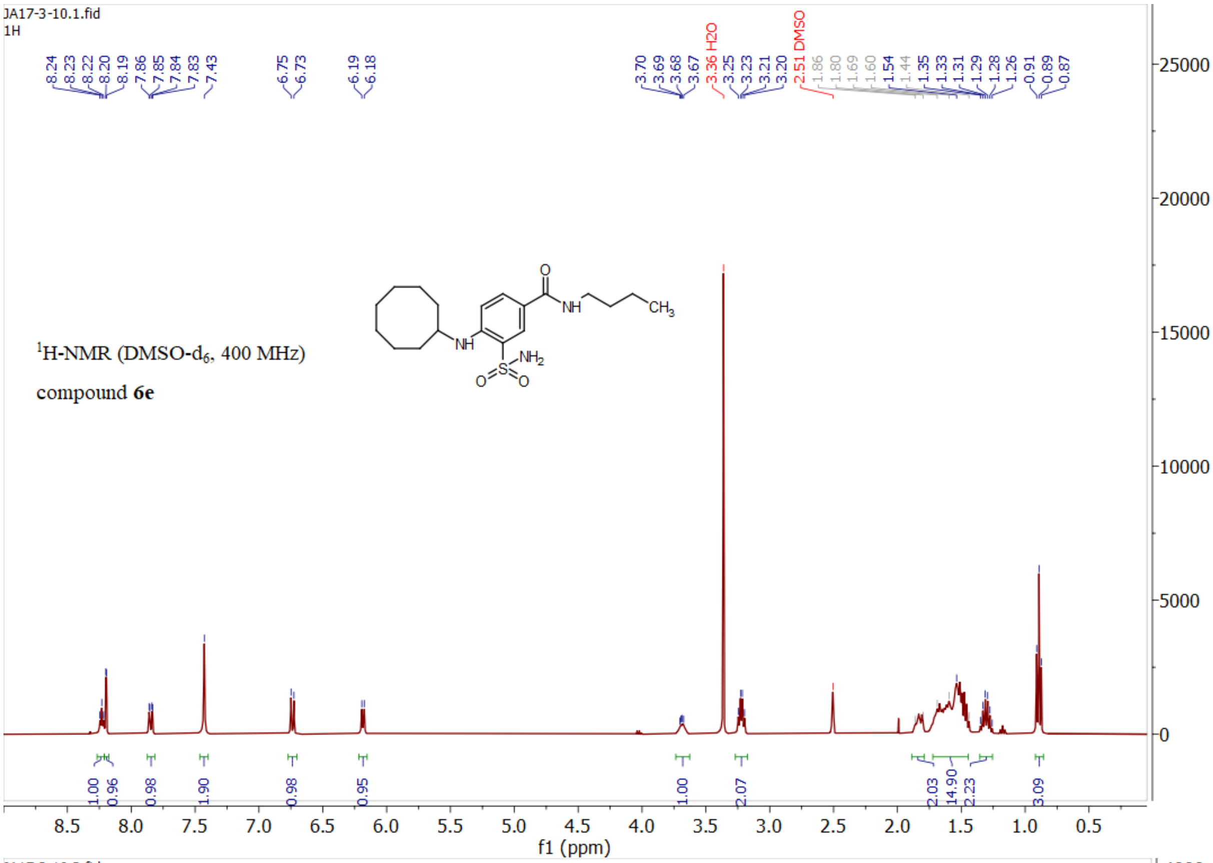
JA17-3-10.1.fid
1H
^{1}H-NMR (DMSO-d$_6$, 400 MHz)
compound **6e**
3.36 H2O
2.51 DMSO
f1 (ppm)

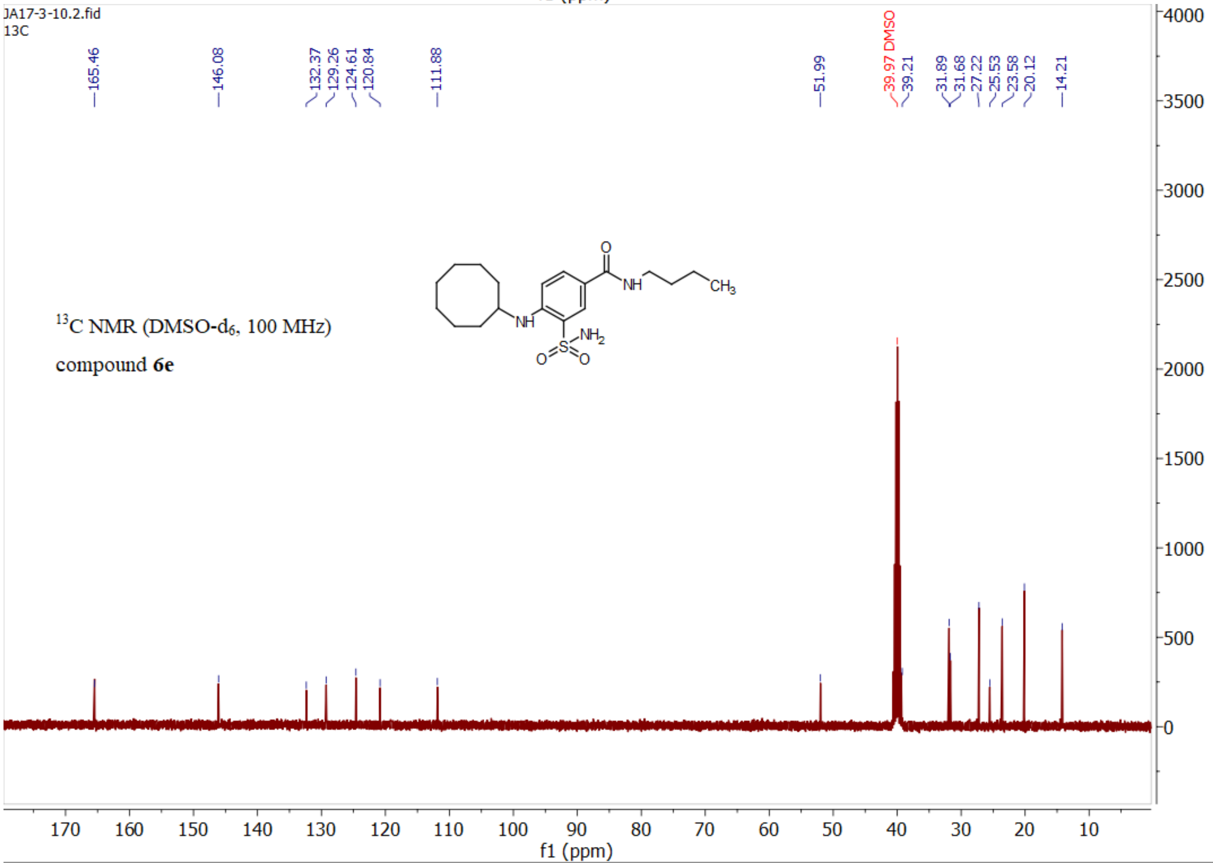
JA17-3-10.2.fid
13C
^{13}C NMR (DMSO-d$_6$, 100 MHz)
compound **6e**
165.46
146.08
132.37
129.26
124.61
120.84
111.88
51.99
39.97 DMSO
39.21
31.89
31.68
27.22
25.53
23.58
20.12
14.21
f1 (ppm)

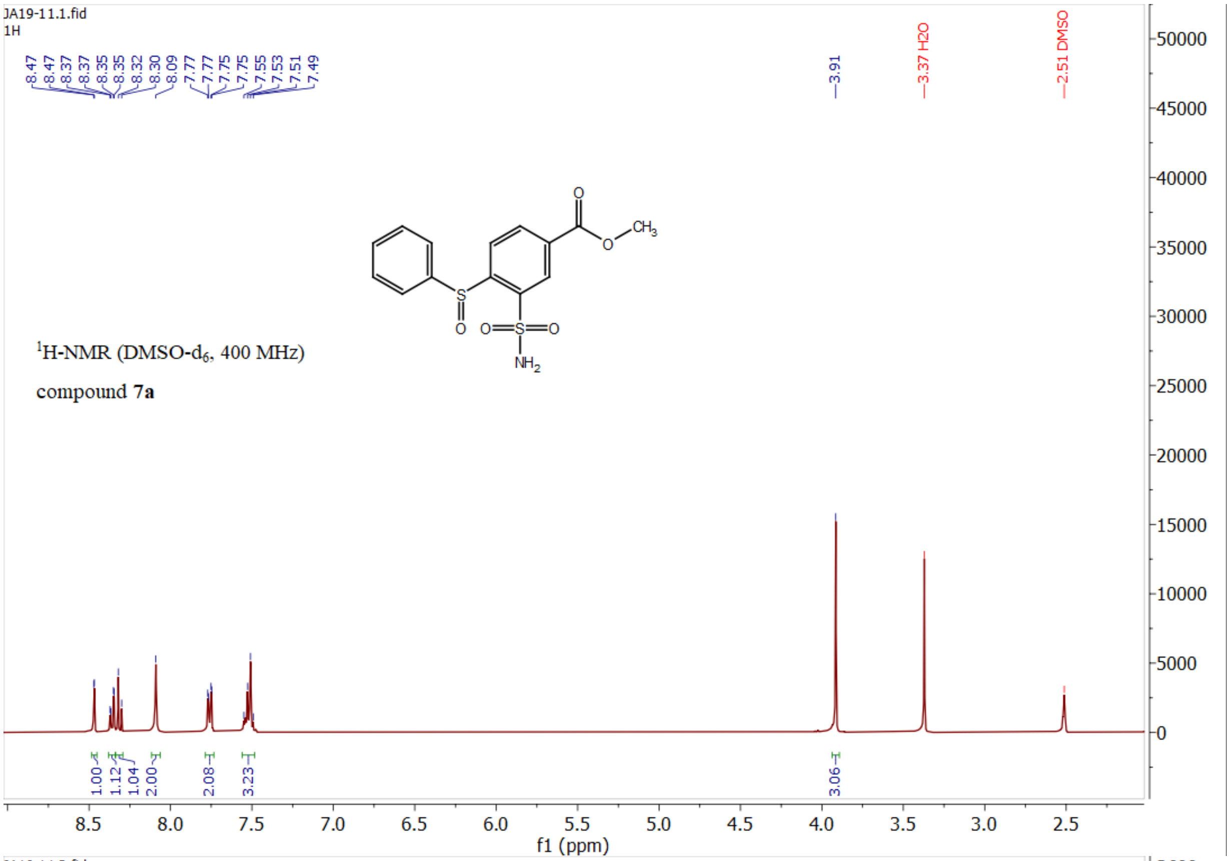
JA19-11.1.fid
1H
8.47 8.47 8.37 8.37 8.35 8.35 8.32 8.30 8.09 7.77 7.77 7.75 7.75 7.55 7.53 7.51 7.49
3.91
3.37 H2O
2.51 DMSO
^{1}H-NMR (DMSO-d$_6$, 400 MHz)
compound **7a**
1.00 1.12 1.04 2.00 2.08 3.23 3.06
f1 (ppm)

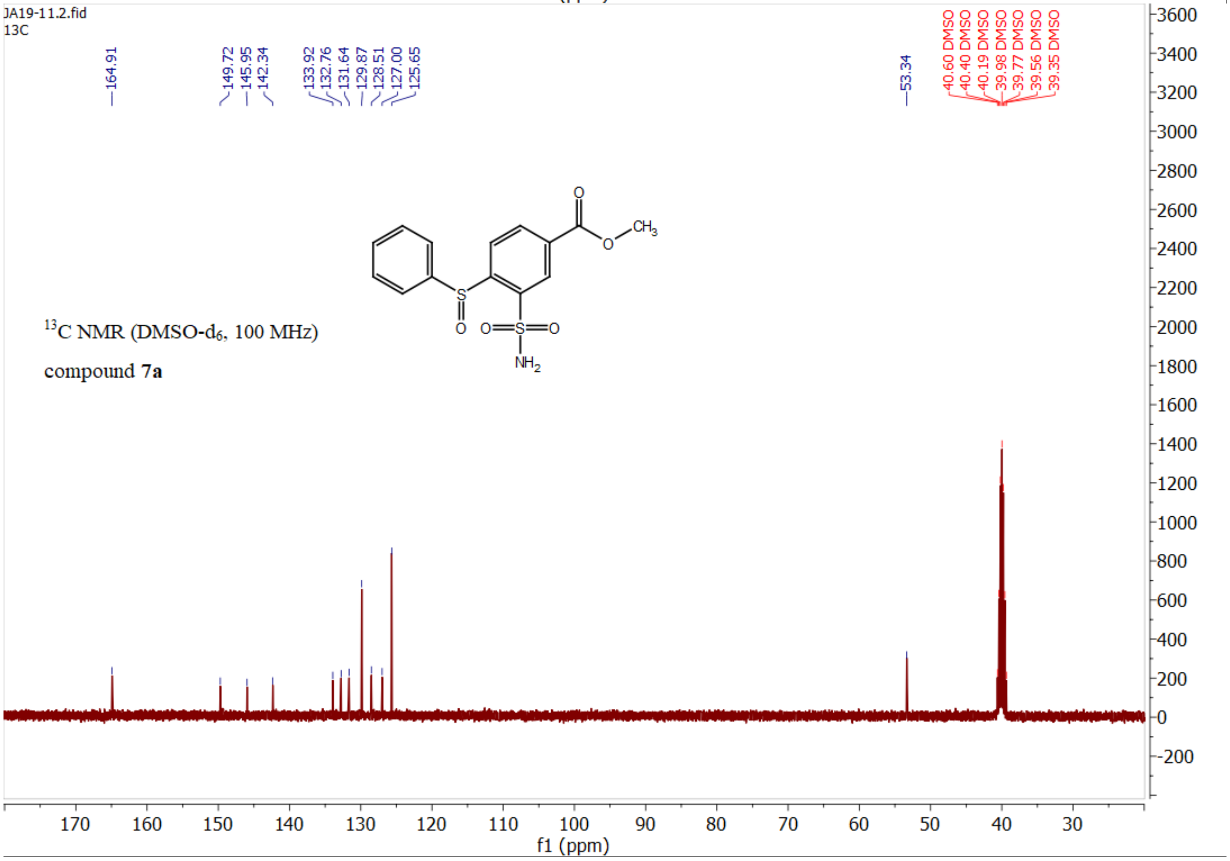
JA19-11.2.fid
13C
164.91
149.72 145.95 142.34
133.92 132.76 131.64 129.87 128.51 127.00 125.65
53.34
40.60 DMSO 40.40 DMSO 40.19 DMSO 39.98 DMSO 39.77 DMSO 39.56 DMSO 39.35 DMSO
^{13}C NMR (DMSO-d$_6$, 100 MHz)
compound **7a**
f1 (ppm)

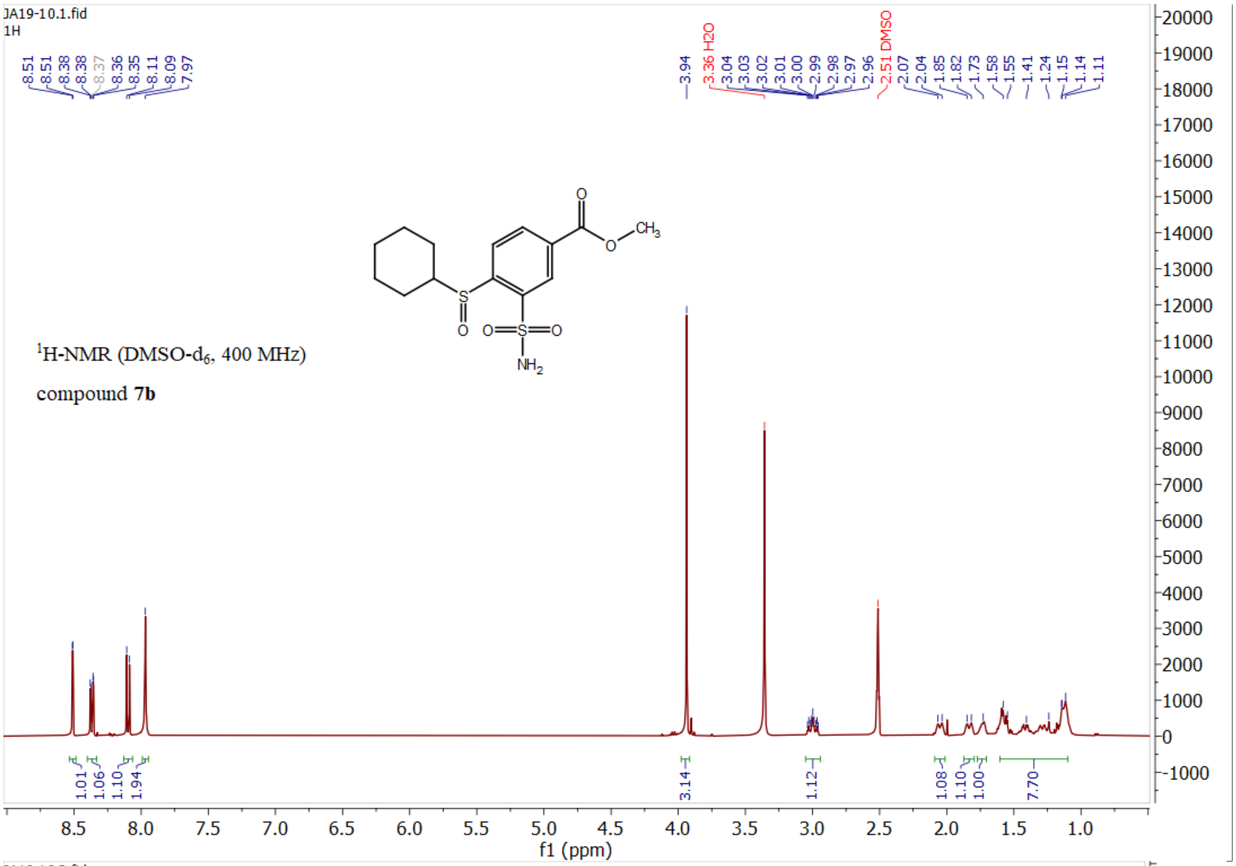

^{1}H-NMR (DMSO-d_6, 400 MHz)

compound **7b**

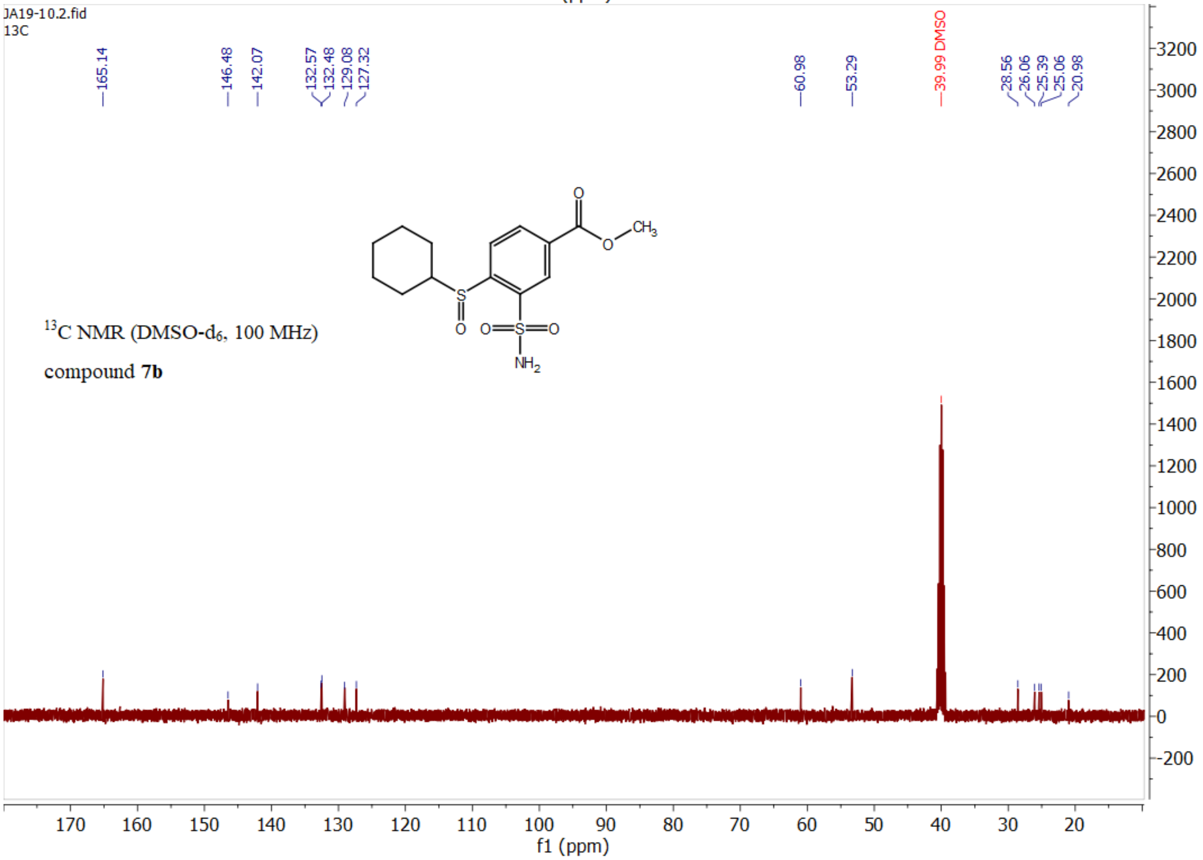

^{13}C NMR (DMSO-d_6, 100 MHz)

compound **7b**

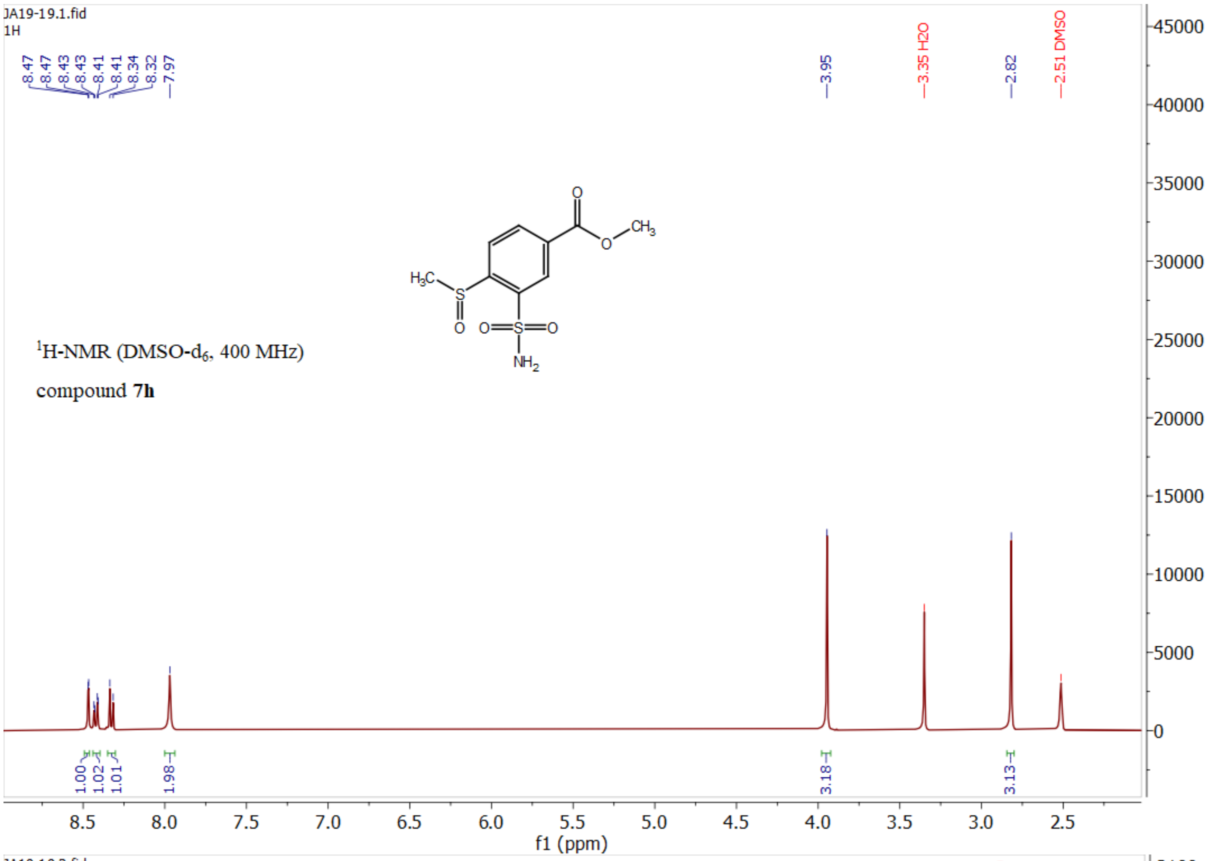

JA19-19.1.fid
1H
8.47
8.47
8.43
8.43
8.41
8.41
8.34
8.32
7.97
3.95
3.35 H2O
2.82
2.51 DMSO
¹H-NMR (DMSO-d₆, 400 MHz)
compound 7h
H₃C
S
O
O
O
CH₃
O=S=O
NH₂
1.00
1.02
1.01
1.98
3.18
3.13
f1 (ppm)

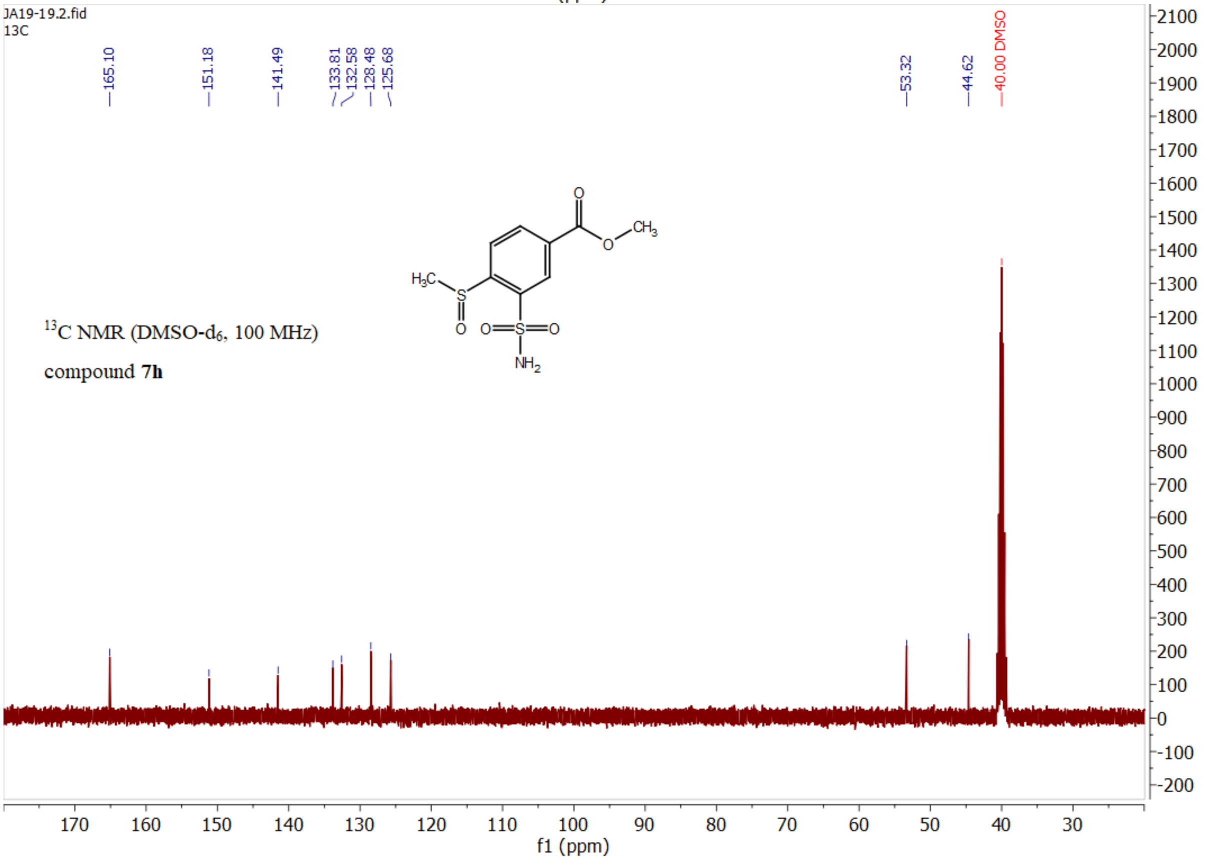

JA19-19.2.fid
13C
165.10
151.18
141.49
133.81
132.58
128.48
125.68
53.32
44.62
40.00 DMSO
¹³C NMR (DMSO-d₆, 100 MHz)
compound 7h
H₃C
S
O
O
O
CH₃
O=S=O
NH₂
f1 (ppm)

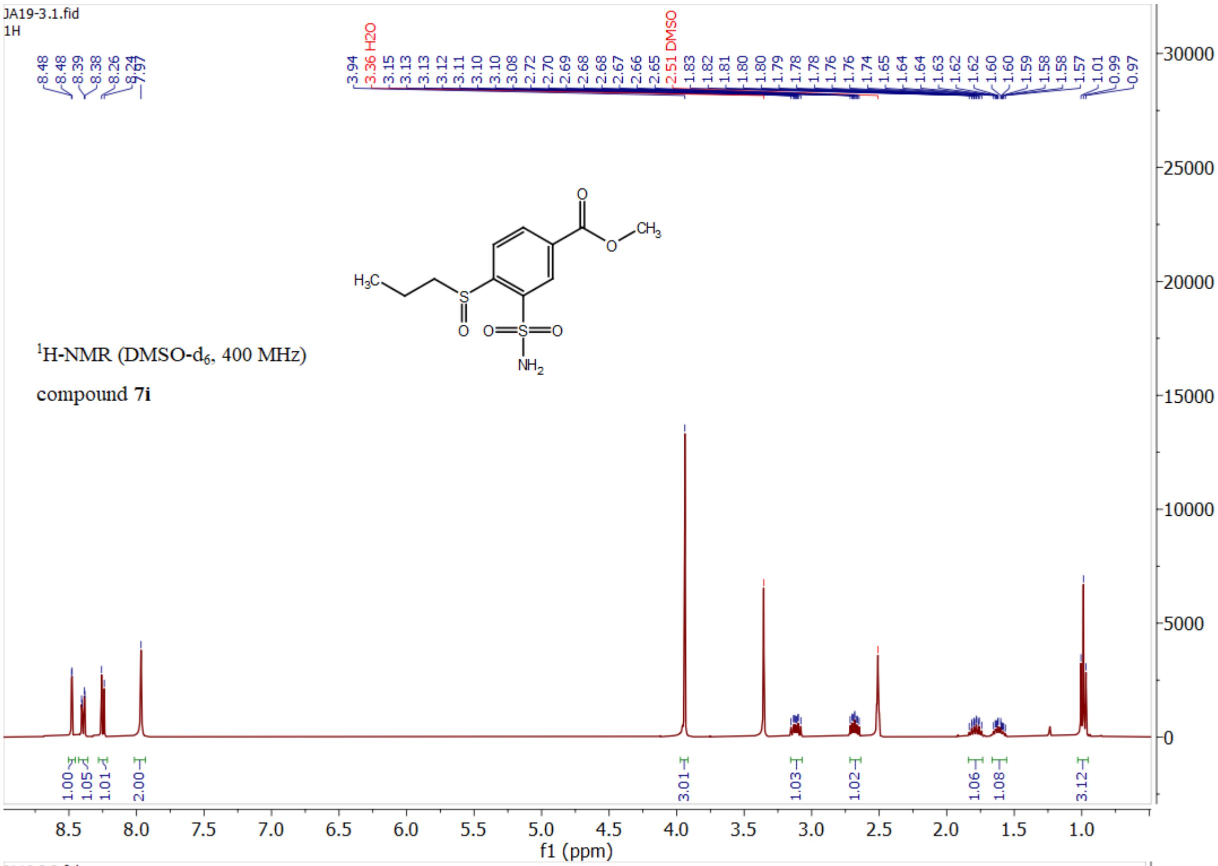
JA19-3.1.fid
1H
8.48 8.48 8.39 8.38 8.26 8.24 7.97
3.94 3.36 H2O 3.15 3.13 3.13 3.12 3.11 3.10 3.10 3.08 2.72 2.70 2.69 2.68 2.68 2.67 2.66 2.65 2.51 DMSO 1.83 1.82 1.81 1.80 1.80 1.79 1.78 1.78 1.76 1.76 1.74 1.65 1.64 1.64 1.63 1.62 1.62 1.60 1.60 1.59 1.58 1.58 1.57 1.01 0.99 0.97
H3C
O
CH3
S
O
O=S=O
NH2
1H-NMR (DMSO-d6, 400 MHz)
compound 7i
1.00 1.05 1.01 2.00 3.01 1.03 1.02 1.06 1.08 3.12
f1 (ppm)

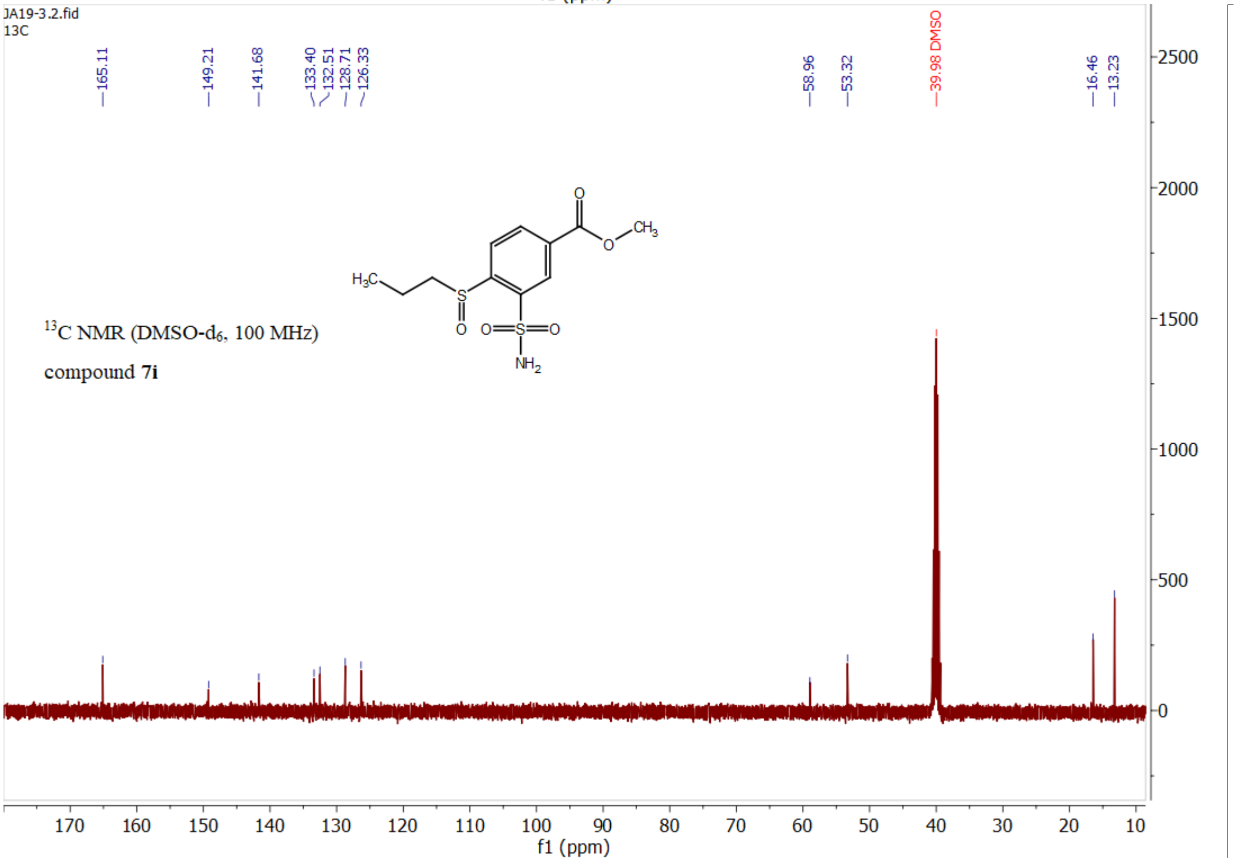
JA19-3.2.fid
13C
165.11 149.21 141.68 133.40 132.51 128.71 126.33 58.96 53.32 39.98 DMSO 16.46 13.23
H3C
O
CH3
S
O
O=S=O
NH2
13C NMR (DMSO-d6, 100 MHz)
compound 7i
f1 (ppm)

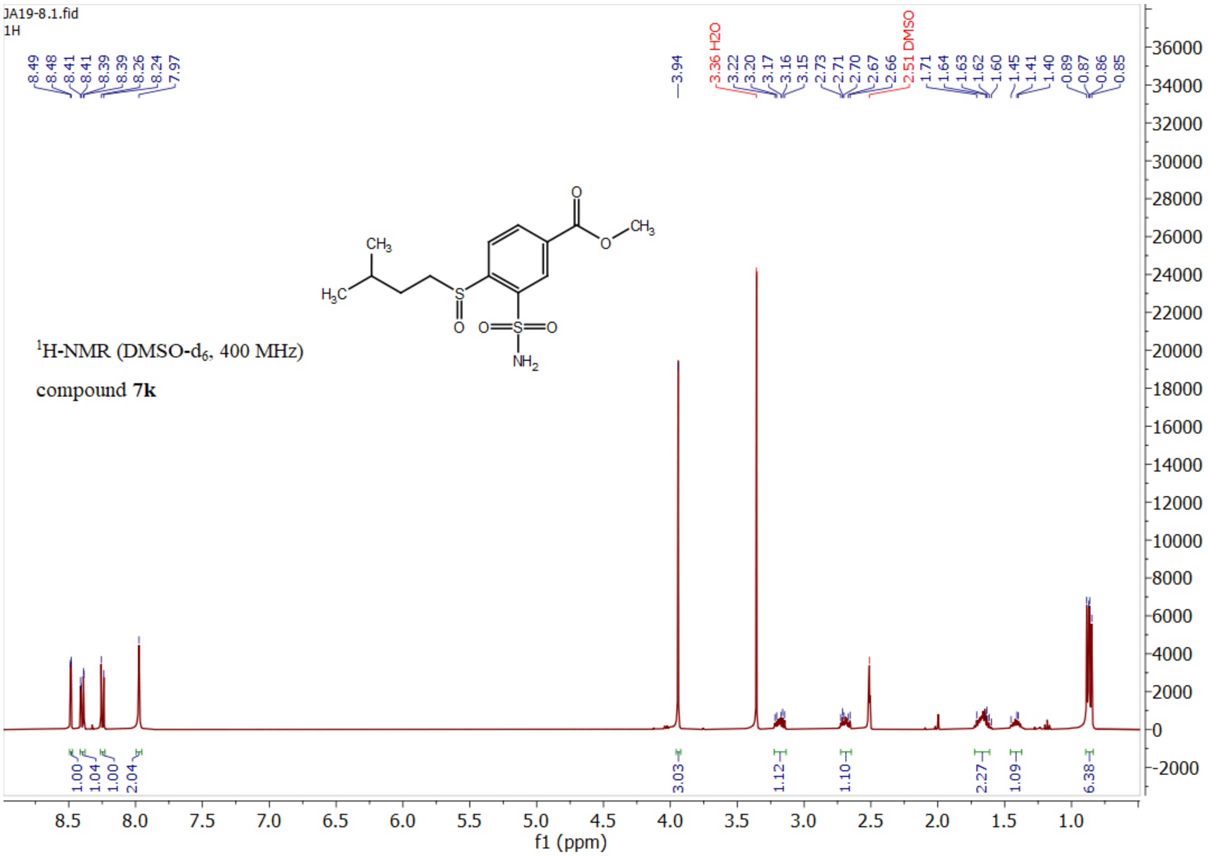
JA19-8.1.fid
1H
8.49
8.48
8.41
8.41
8.39
8.39
8.26
8.24
7.97
3.94
3.36 H2O
3.22
3.20
3.17
3.16
3.15
2.73
2.71
2.70
2.67
2.66
2.51 DMSO
1.71
1.64
1.63
1.62
1.60
1.45
1.41
1.40
0.89
0.87
0.86
0.85
1H-NMR (DMSO-d6, 400 MHz)
compound 7k
1.00
1.04
1.00
2.04
3.03
1.12
1.10
2.27
1.09
6.38
f1 (ppm)

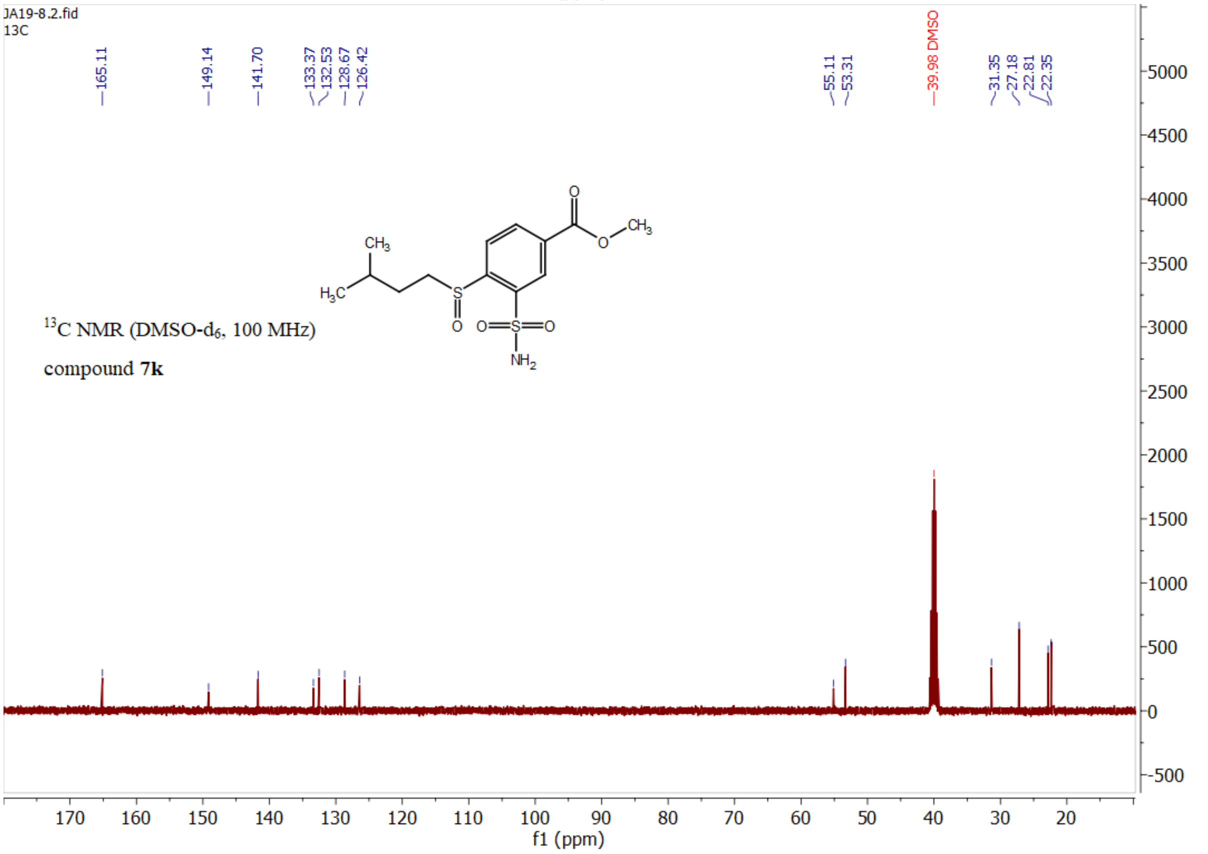
JA19-8.2.fid
13C
165.11
149.14
141.70
133.37
132.53
128.67
126.42
55.11
53.31
39.98 DMSO
31.35
27.18
22.81
22.35
13C NMR (DMSO-d6, 100 MHz)
compound 7k
f1 (ppm)

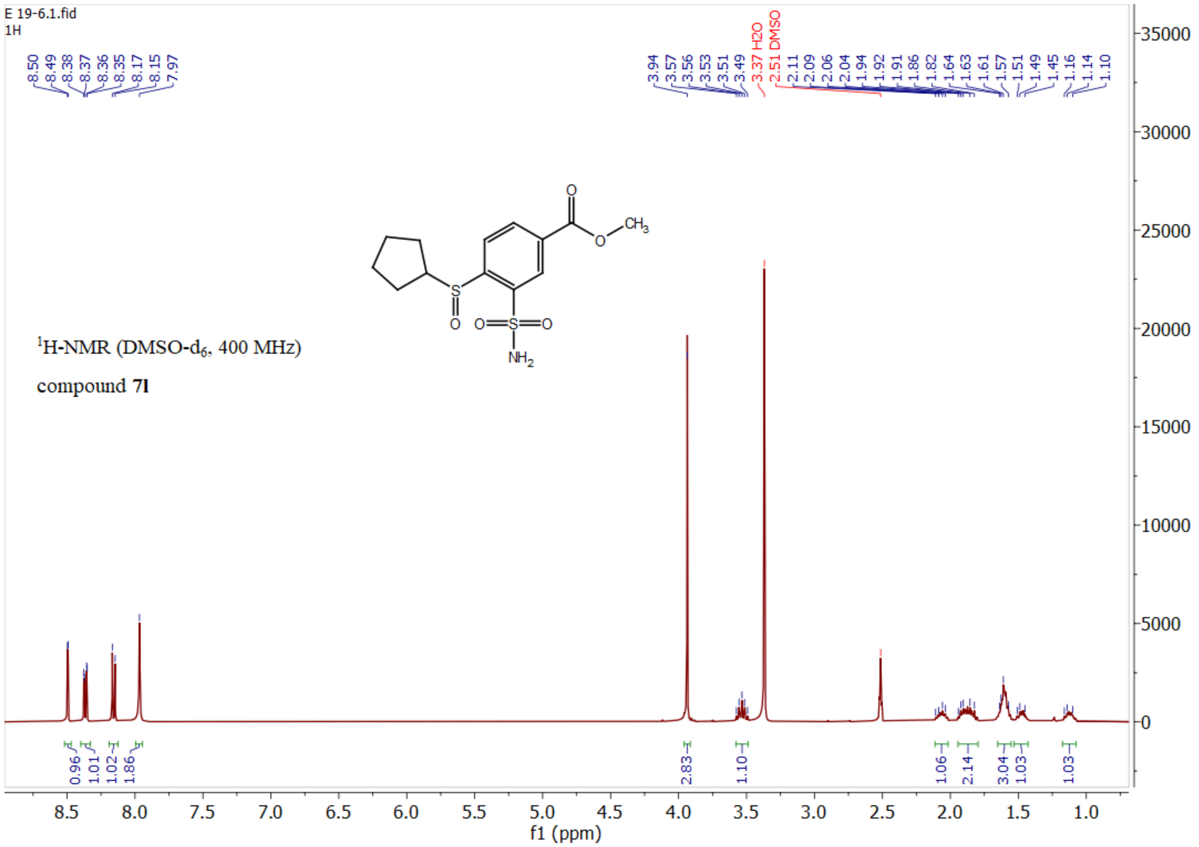
E 19-6.1.fid
1H
8.50
8.49
8.38
8.37
8.36
8.35
8.17
8.15
7.97
3.94
3.57
3.56
3.53
3.51
3.49
3.37 H2O
2.51 DMSO
2.11
2.09
2.06
2.04
1.94
1.92
1.91
1.86
1.82
1.64
1.63
1.61
1.57
1.51
1.49
1.45
1.16
1.14
1.10
O
CH3
S
O
O=S=O
NH2
^{1}H-NMR (DMSO-d$_6$, 400 MHz)
compound **7l**
0.96
1.01
1.02
1.86
2.83
1.10
1.06
2.14
3.04
1.03
1.03
f1 (ppm)

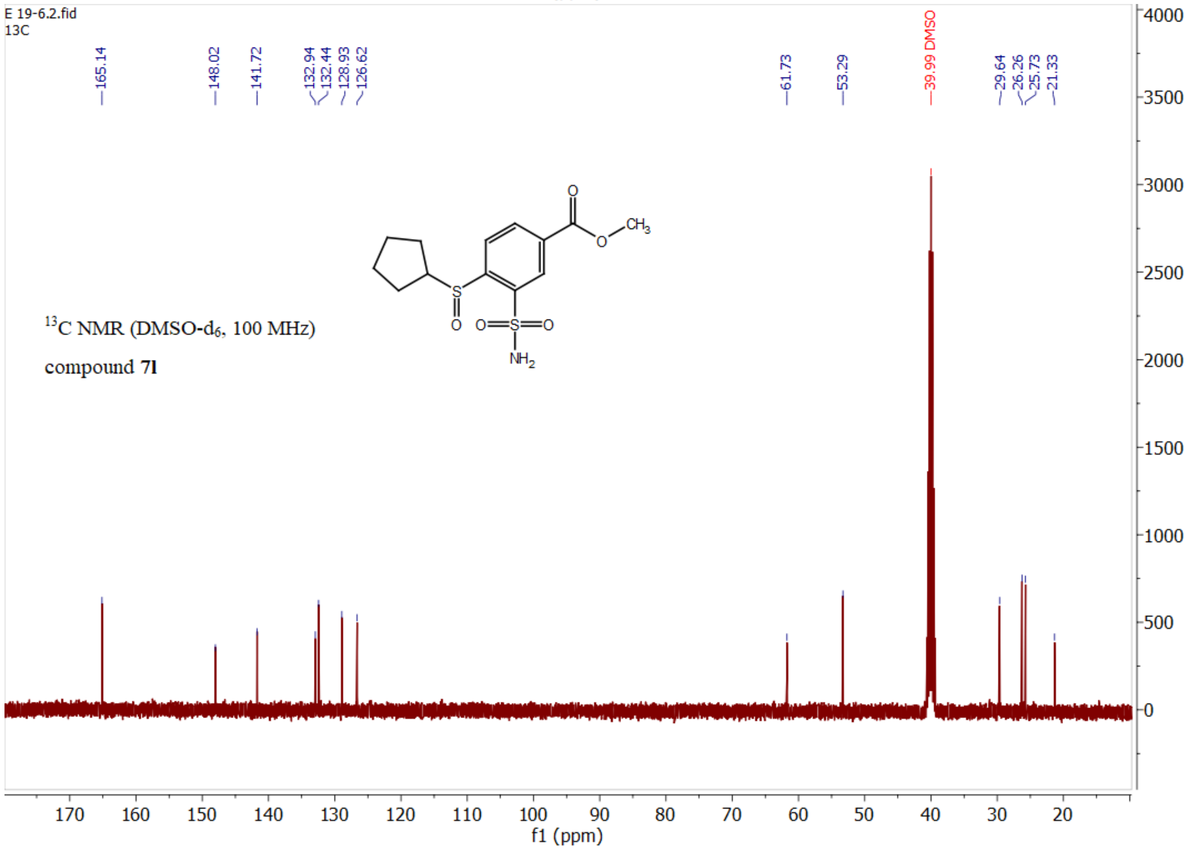
E 19-6.2.fid
13C
165.14
148.02
141.72
132.94
132.44
128.93
126.62
61.73
53.29
39.99 DMSO
29.64
26.26
25.73
21.33
O
CH3
S
O
O=S=O
NH2
^{13}C NMR (DMSO-d$_6$, 100 MHz)
compound **7l**
f1 (ppm)

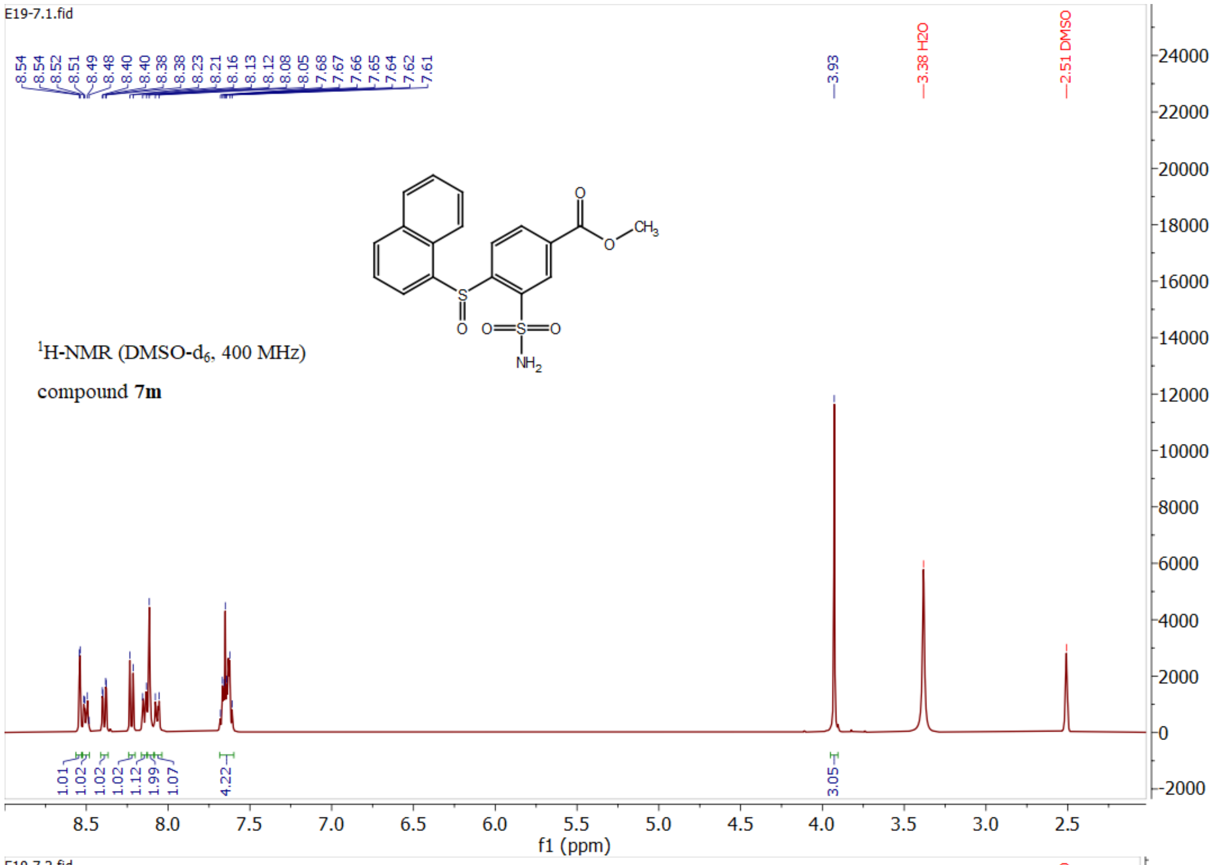
E19-7.1.fid
8.54 8.54 8.52 8.51 8.49 8.48 8.40 8.40 8.38 8.38 8.38 8.23 8.21 8.16 8.13 8.12 8.08 8.05 7.68 7.67 7.66 7.65 7.64 7.62 7.61
3.93
3.38 H2O
2.51 DMSO
¹H-NMR (DMSO-d6, 400 MHz)
compound 7m
1.01 1.02 1.02 1.02 1.12 1.99 1.07 4.22 3.05
f1 (ppm)

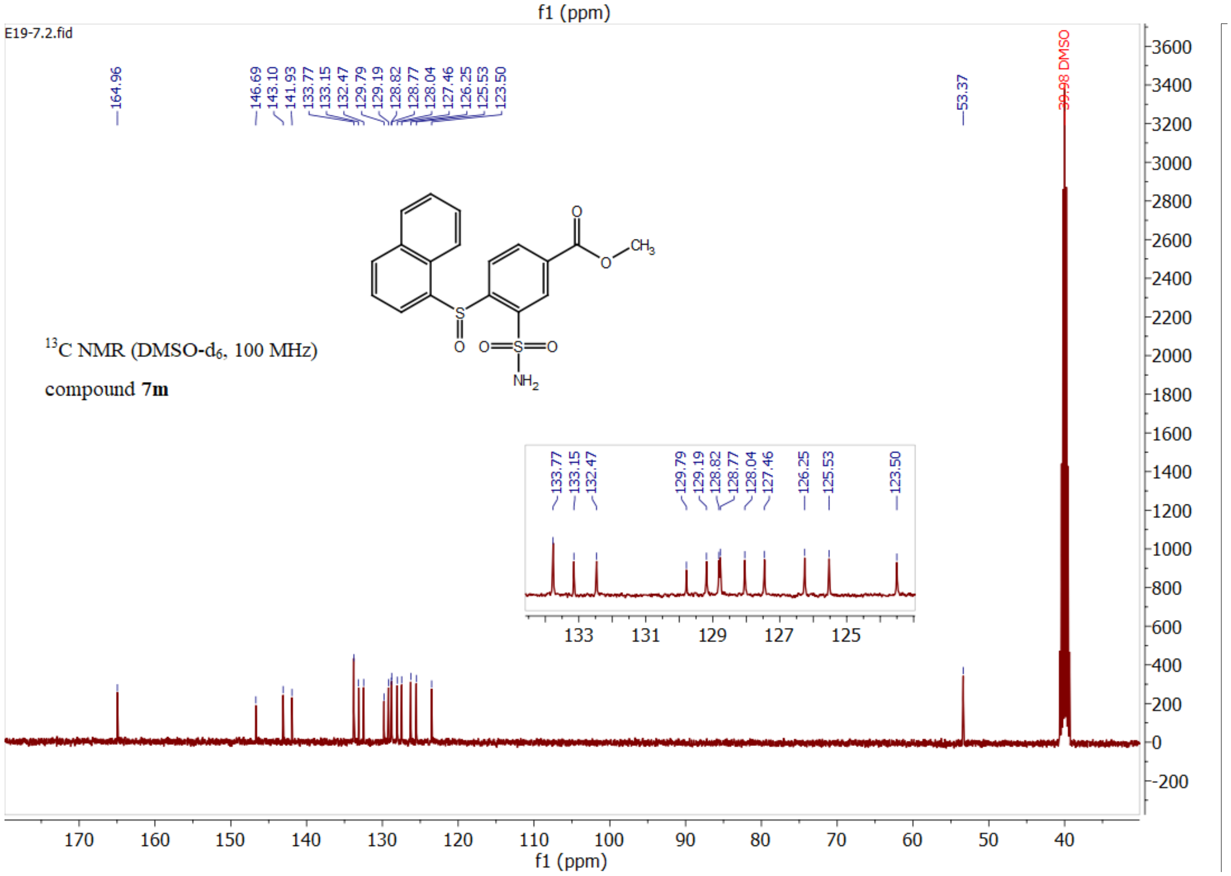
E19-7.2.fid
164.96
146.69 143.10 141.93 133.77 133.15 132.47 129.79 129.19 128.82 128.77 128.04 127.46 126.25 125.53 123.50
53.37
39.98 DMSO
¹³C NMR (DMSO-d6, 100 MHz)
compound 7m
f1 (ppm)

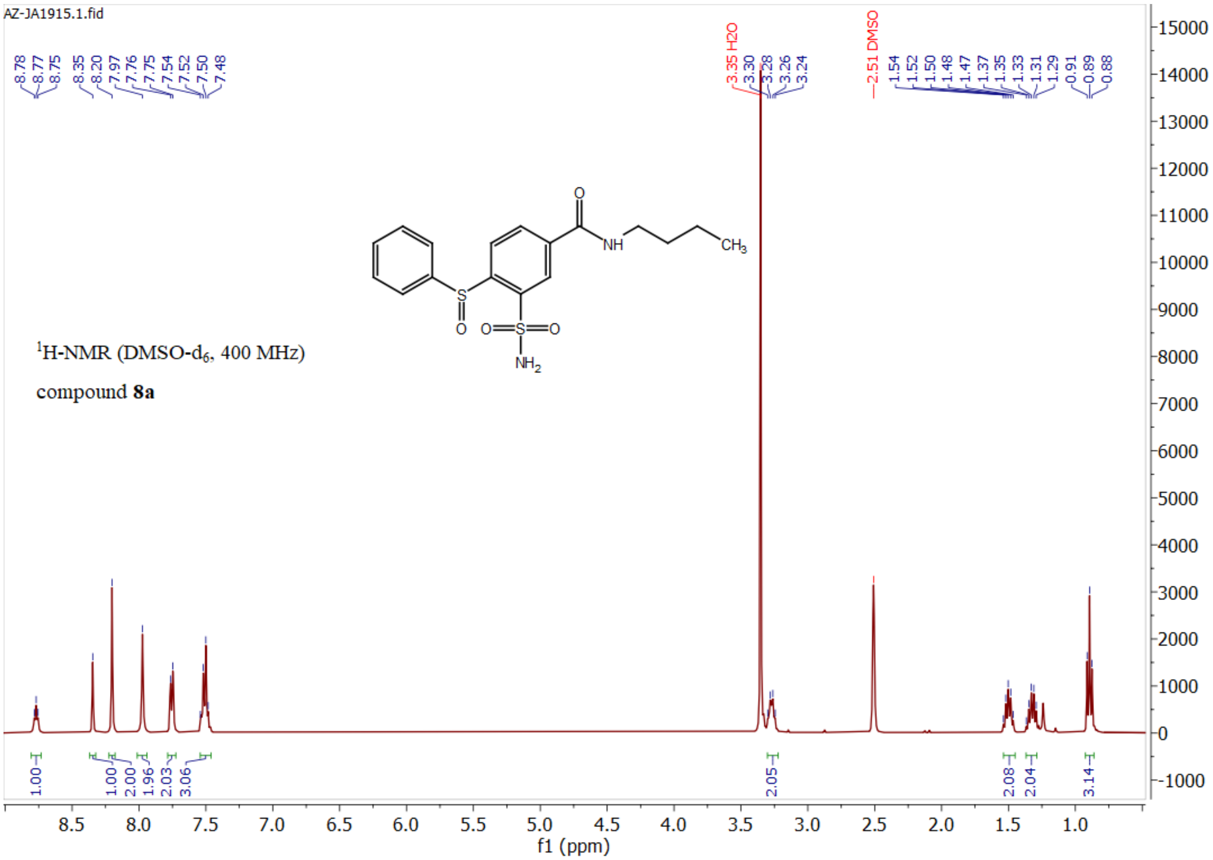

AZ-JA1915.1.fid
8.78
8.77
8.75
8.35
8.20
7.97
7.76
7.75
7.54
7.52
7.50
7.48
3.35 H2O
3.30
3.28
3.26
3.24
2.51 DMSO
1.54
1.52
1.50
1.48
1.47
1.37
1.35
1.33
1.31
1.29
0.91
0.89
0.88
NH
CH3
O
S
O=S=O
NH2
1H-NMR (DMSO-d6, 400 MHz)
compound 8a
1.00
2.00
1.96
2.03
3.06
2.05
2.08
2.04
3.14
f1 (ppm)

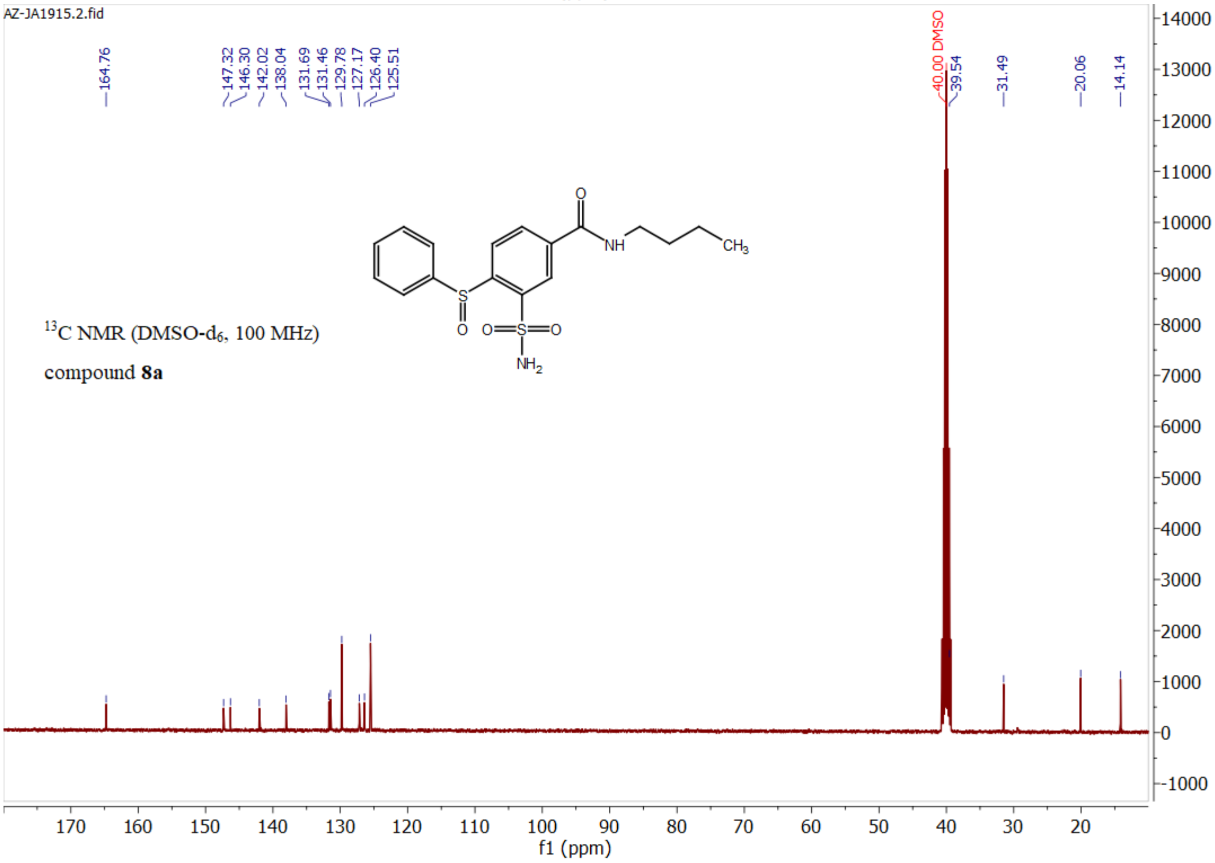

AZ-JA1915.2.fid
164.76
147.32
146.30
142.02
138.04
131.69
131.46
129.78
127.17
126.40
125.51
40.00 DMSO
39.54
31.49
20.06
14.14
NH
CH3
O
S
O=S=O
NH2
13C NMR (DMSO-d6, 100 MHz)
compound 8a
f1 (ppm)

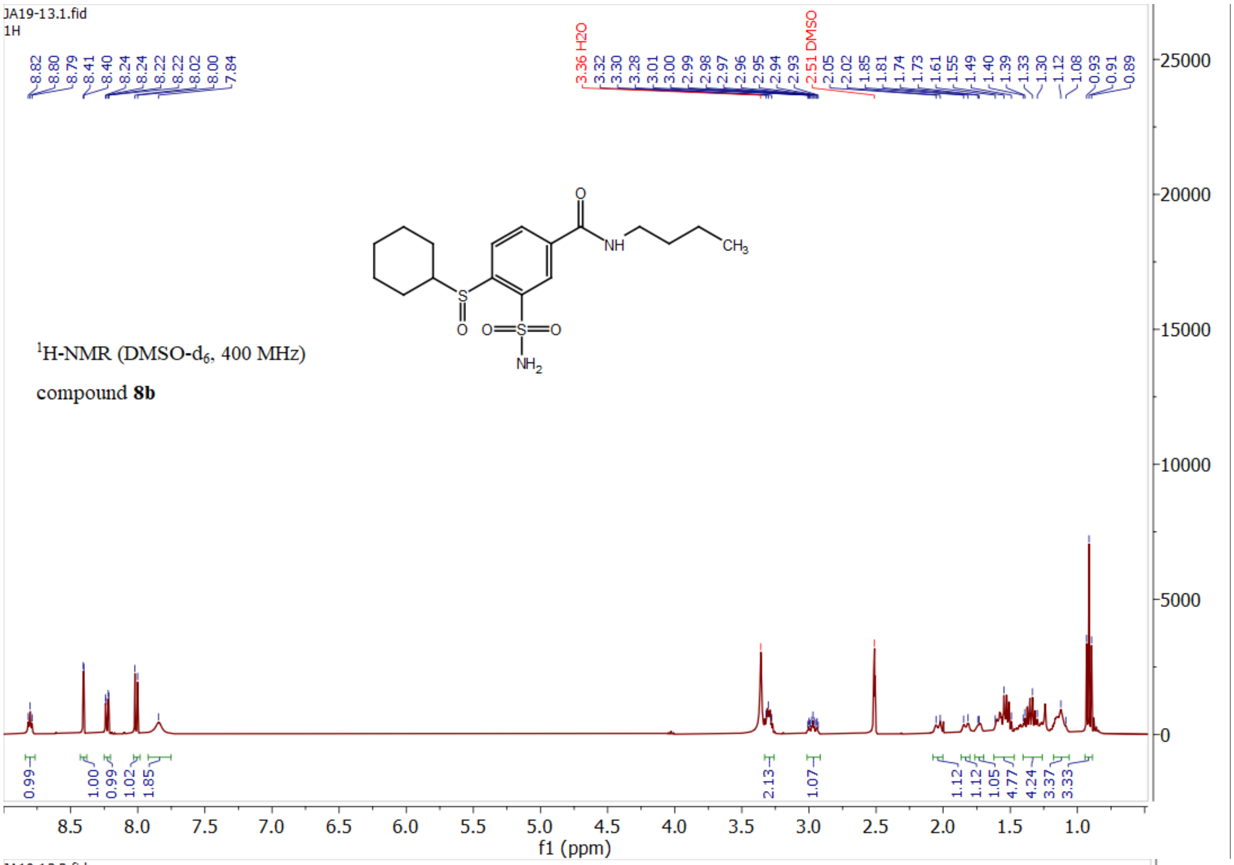

JA19-13.1.fid
1H
8.82 8.80 8.79 8.41 8.40 8.24 8.24 8.22 8.22 8.02 8.00 7.84
3.36 H2O 3.32 3.30 3.28 3.01 3.00 2.99 2.98 2.97 2.96 2.95 2.94 2.93 2.51 DMSO 2.05 2.02 1.85 1.81 1.74 1.73 1.61 1.55 1.49 1.40 1.39 1.33 1.30 1.12 1.08 0.93 0.91 0.89
^{1}H-NMR (DMSO-d$_6$, 400 MHz)
compound **8b**
0.99 1.00 0.99 1.02 1.85 2.13 1.07 1.12 1.12 1.05 4.77 4.24 3.37 3.33
f1 (ppm)

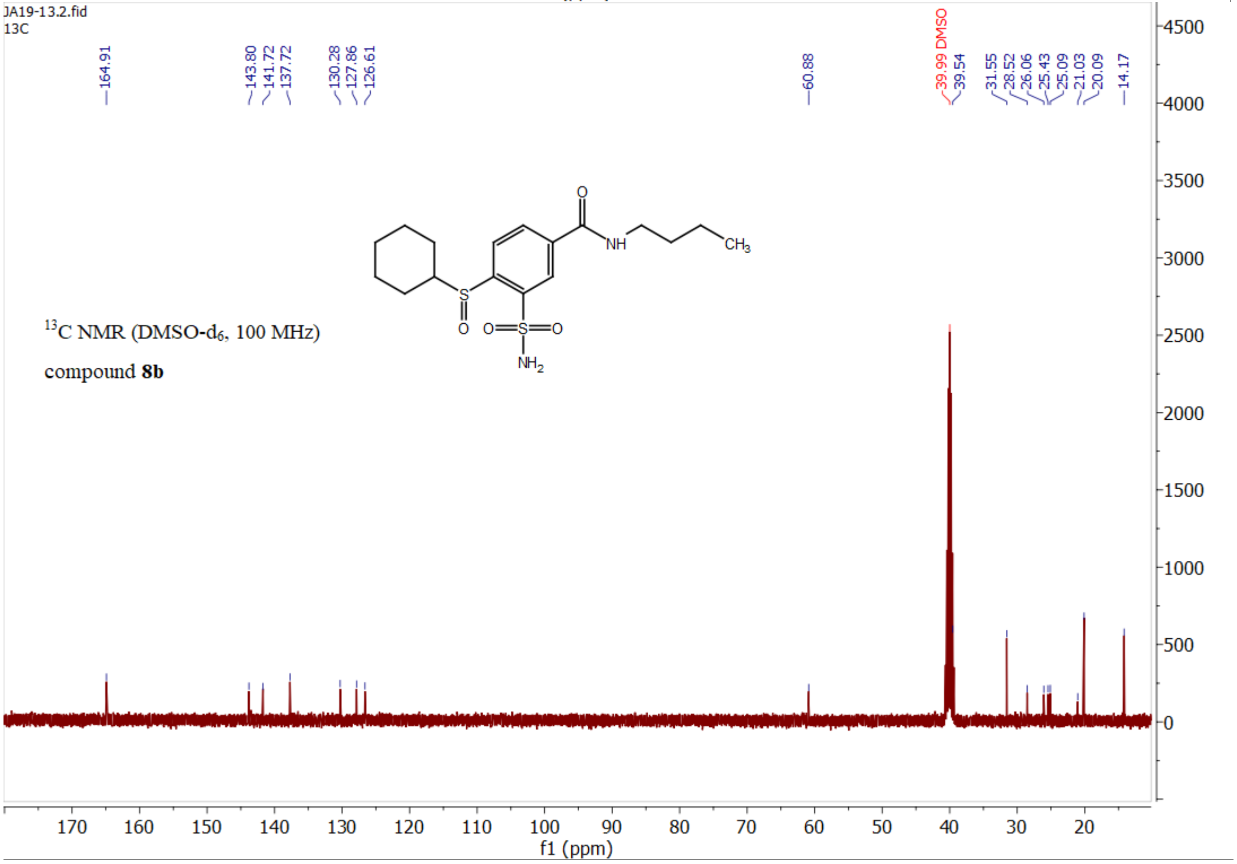

JA19-13.2.fid
13C
164.91 143.80 141.72 137.72 130.28 127.86 126.61 60.88 39.99 DMSO 39.54 31.55 28.52 26.06 25.43 25.09 21.03 20.09 14.17
^{13}C NMR (DMSO-d$_6$, 100 MHz)
compound **8b**
f1 (ppm)

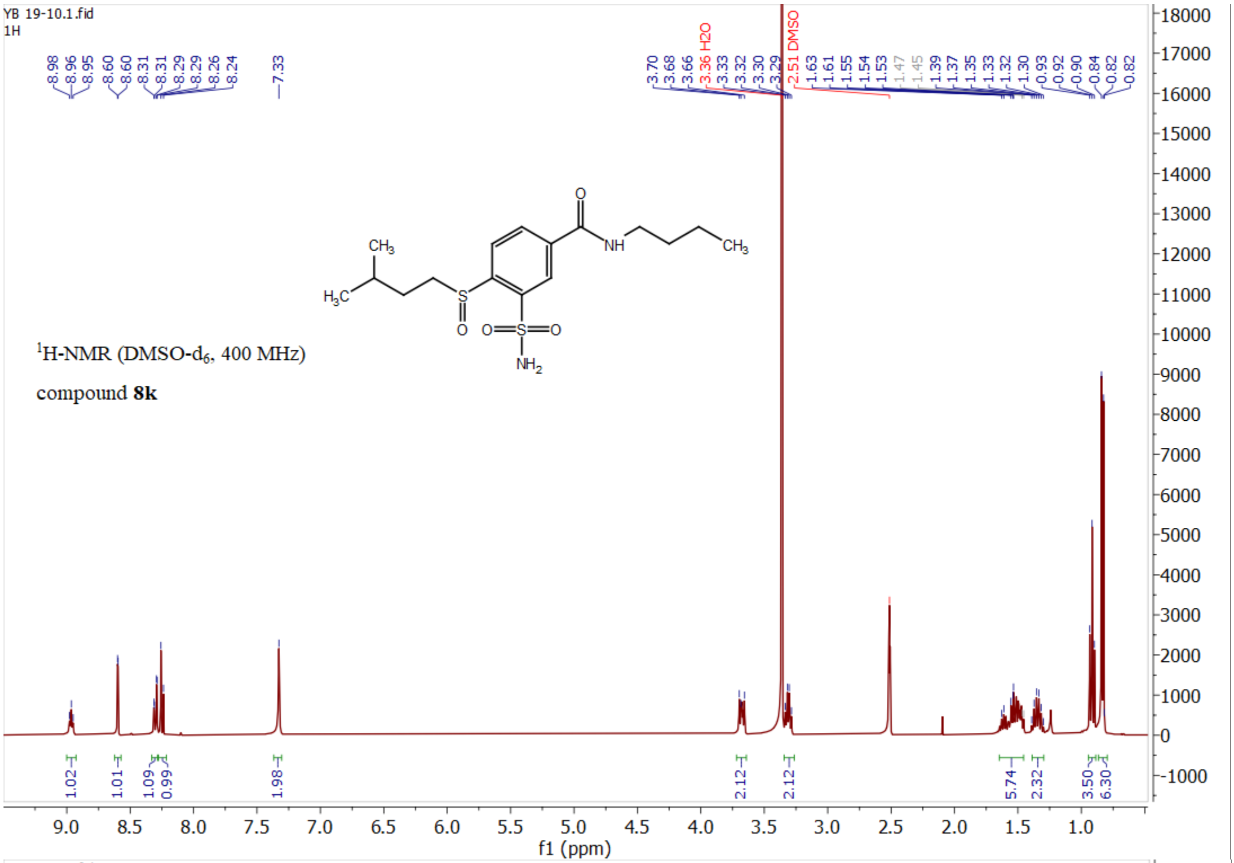
YB 19-10.1.fid
1H
^{1}H-NMR (DMSO-d$_6$, 400 MHz)
compound **8k**
3.36 H2O
2.51 DMSO
f1 (ppm)

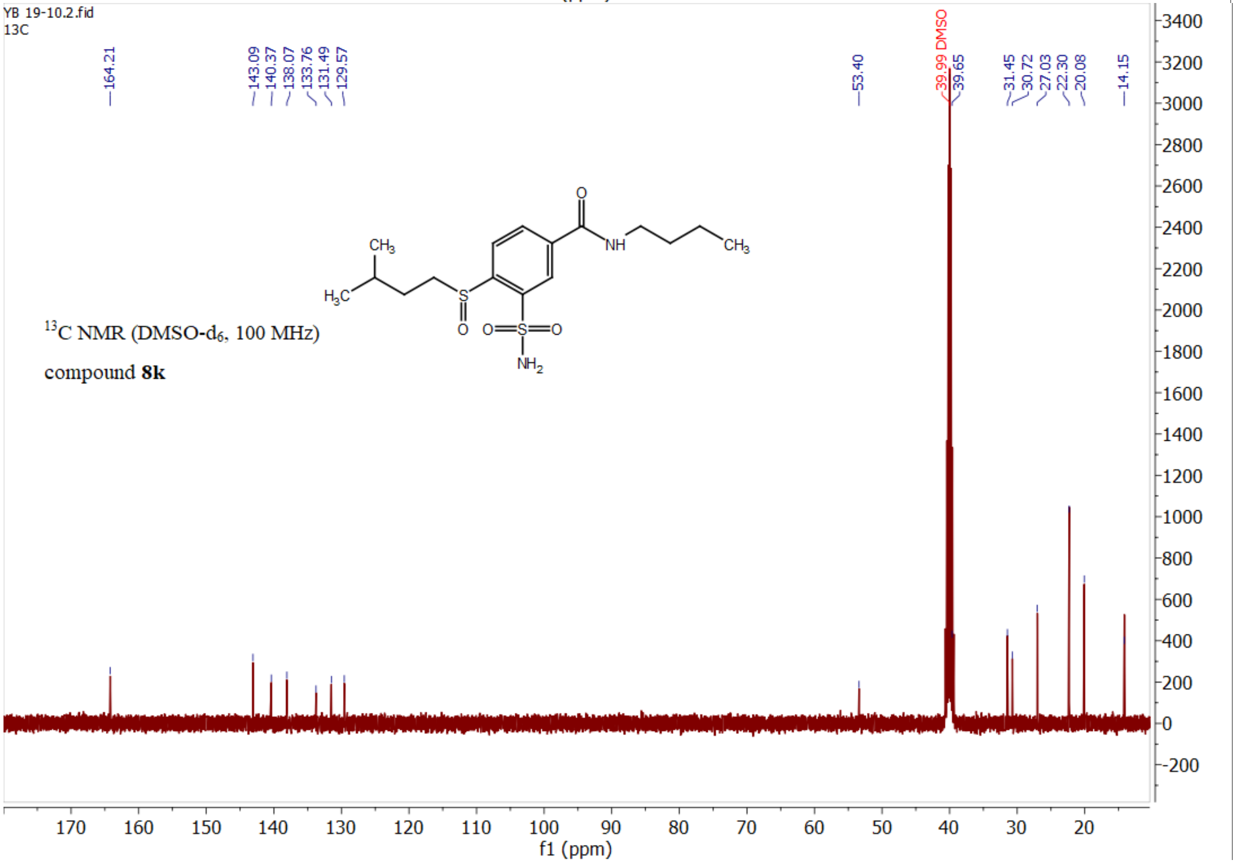
YB 19-10.2.fid
13C
^{13}C NMR (DMSO-d$_6$, 100 MHz)
compound **8k**
164.21
143.09
140.37
138.07
133.76
131.49
129.57
53.40
39.99 DMSO
39.65
31.45
30.72
27.03
22.30
20.08
14.15
f1 (ppm)

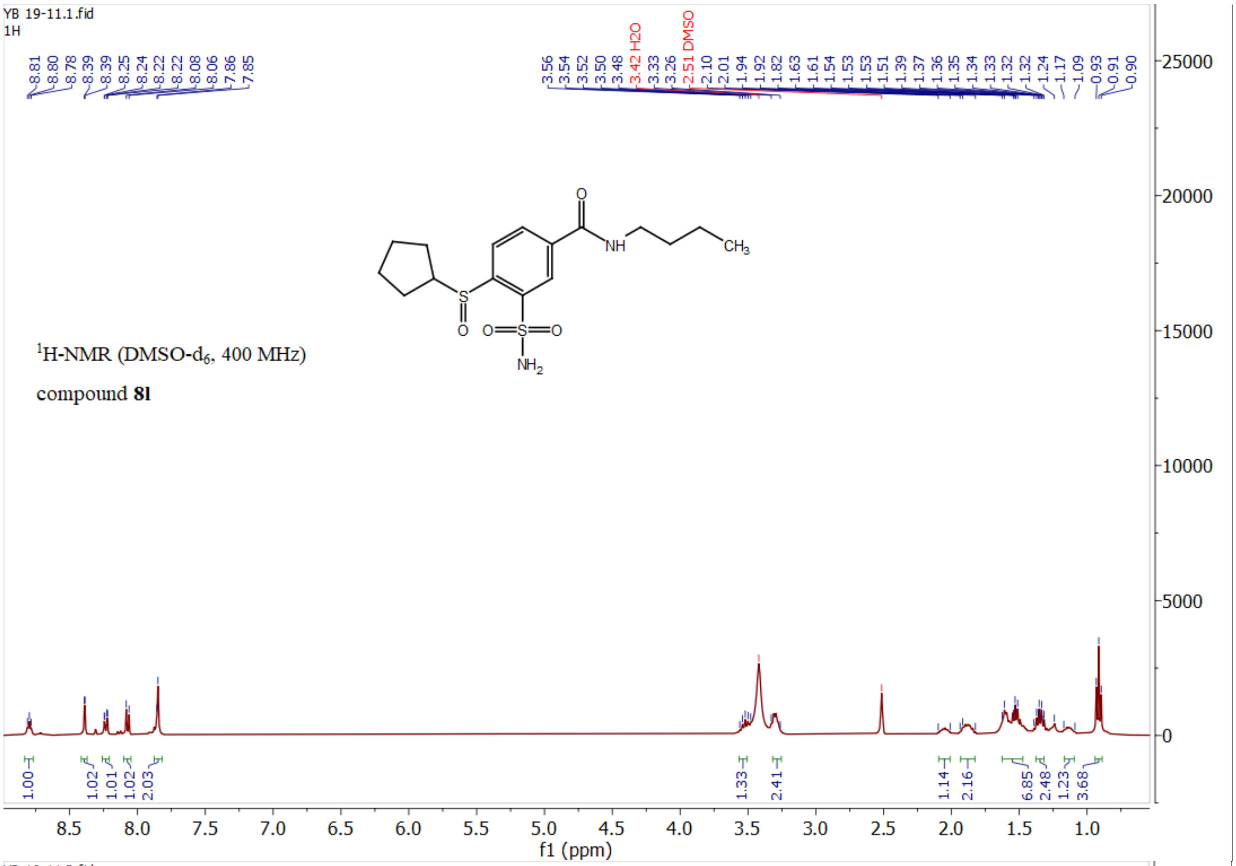

YB 19-11.1.fid
1H
8.81 8.80 8.78 8.39 8.39 8.25 8.24 8.22 8.22 8.08 8.06 7.86 7.85
3.56 3.54 3.52 3.50 3.48 3.42 H2O 3.33 3.26 2.51 DMSO 2.10 2.01 1.94 1.92 1.82 1.63 1.61 1.54 1.53 1.53 1.51 1.39 1.37 1.36 1.35 1.34 1.33 1.32 1.32 1.24 1.17 1.09 0.93 0.91 0.90
O
NH
CH3
S
O
O=S=O
NH2
^{1}H-NMR (DMSO-d$_6$, 400 MHz)
compound **8l**
1.00 1.02 1.01 1.02 2.03 1.33 2.41 1.14 2.16 6.85 2.48 1.23 3.68
25000 20000 15000 10000 5000 0
8.5 8.0 7.5 7.0 6.5 6.0 5.5 5.0 4.5 4.0 3.5 3.0 2.5 2.0 1.5 1.0
f1 (ppm)

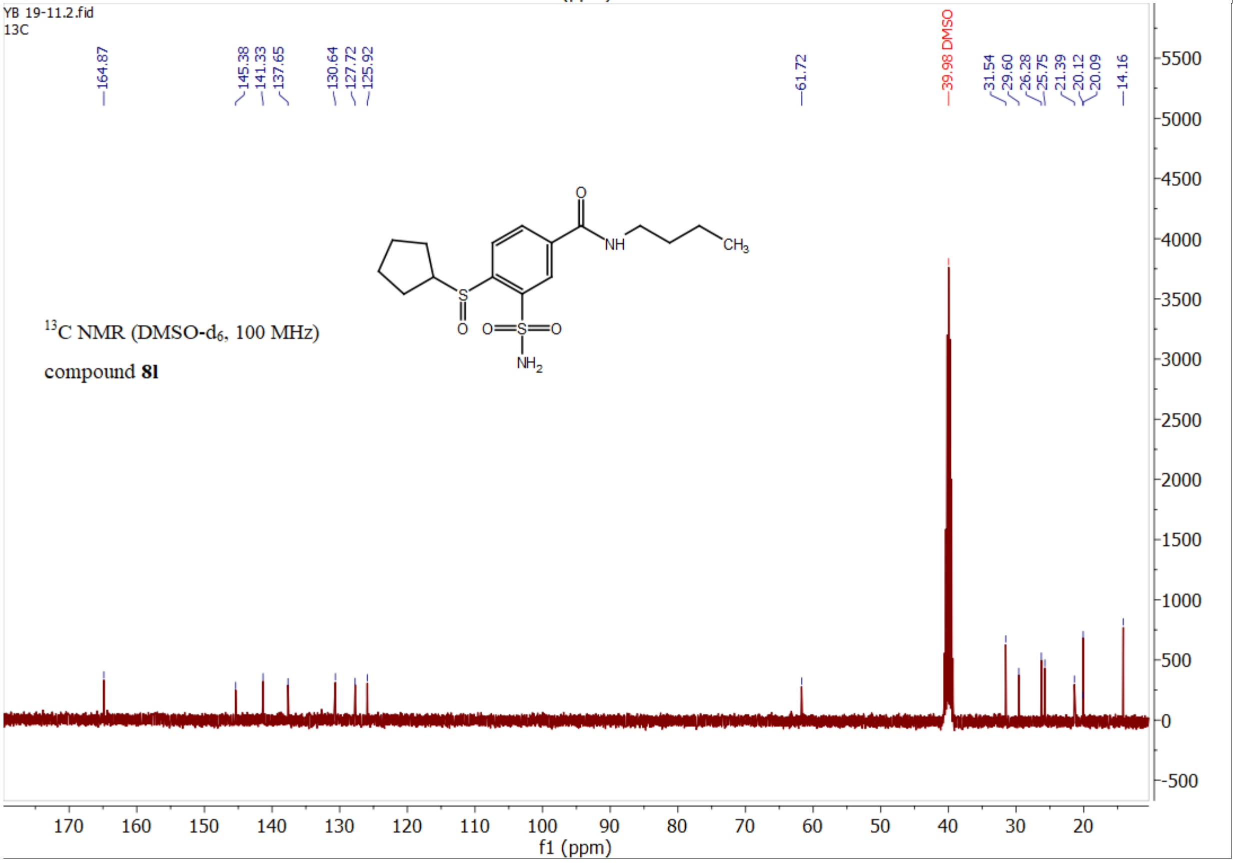

YB 19-11.2.fid
13C
164.87
145.38 141.33 137.65
130.64 127.72 125.92
61.72
39.98 DMSO
31.54 29.60 26.28 25.75 21.39 20.12 20.09
14.16
O
NH
CH3
S
O
O=S=O
NH2
^{13}C NMR (DMSO-d$_6$, 100 MHz)
compound **8l**
5500 5000 4500 4000 3500 3000 2500 2000 1500 1000 500 0 -500
170 160 150 140 130 120 110 100 90 80 70 60 50 40 30 20
f1 (ppm)

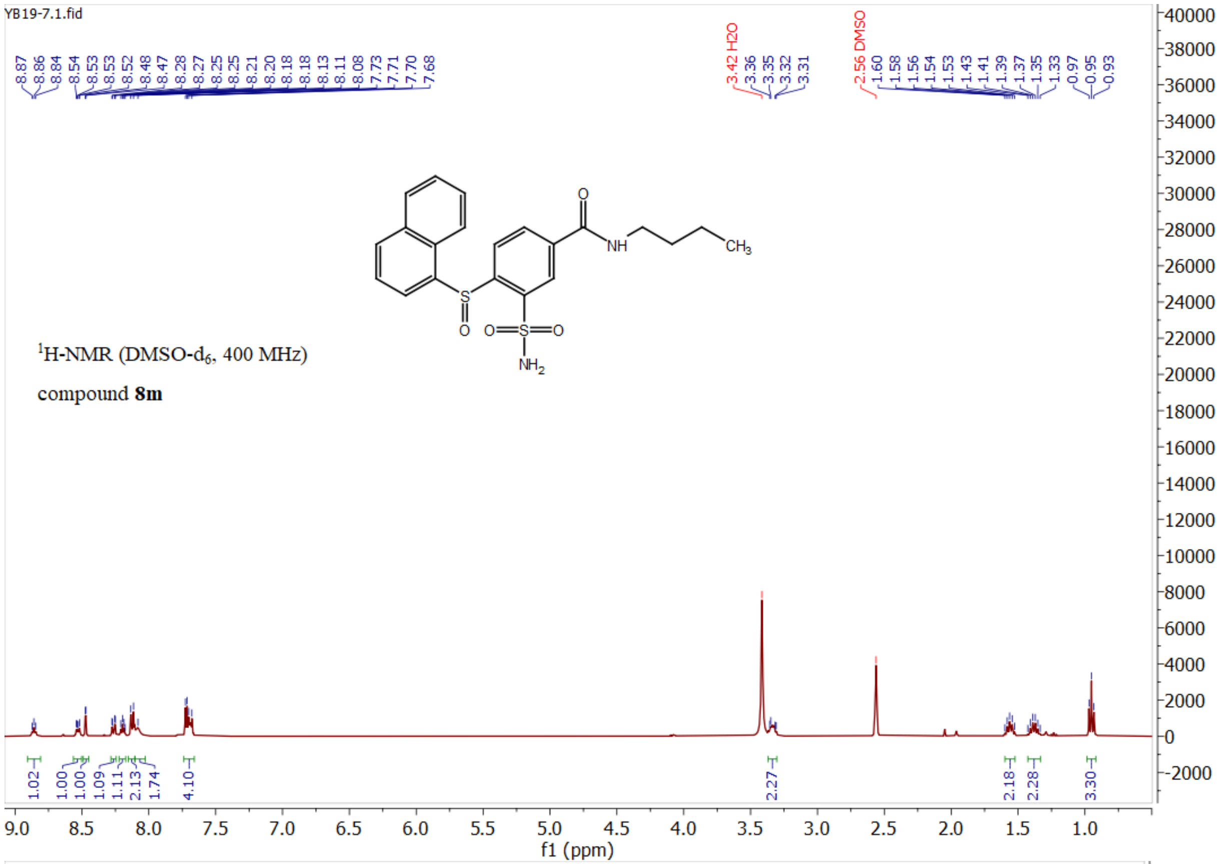
YB19-7.1.fid
1H-NMR (DMSO-d6, 400 MHz)
compound 8m
3.42 H2O
2.56 DMSO
f1 (ppm)

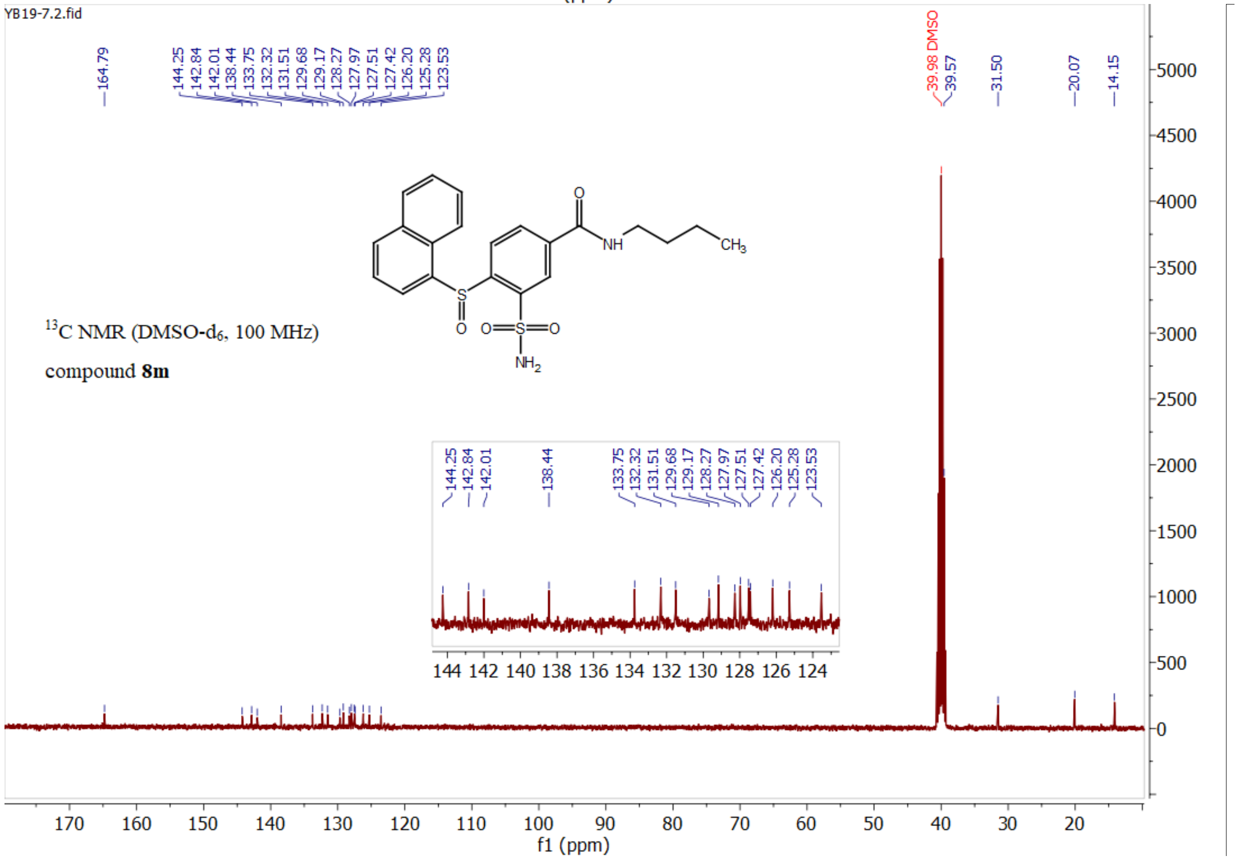
YB19-7.2.fid
13C NMR (DMSO-d6, 100 MHz)
compound 8m
39.98 DMSO
f1 (ppm)

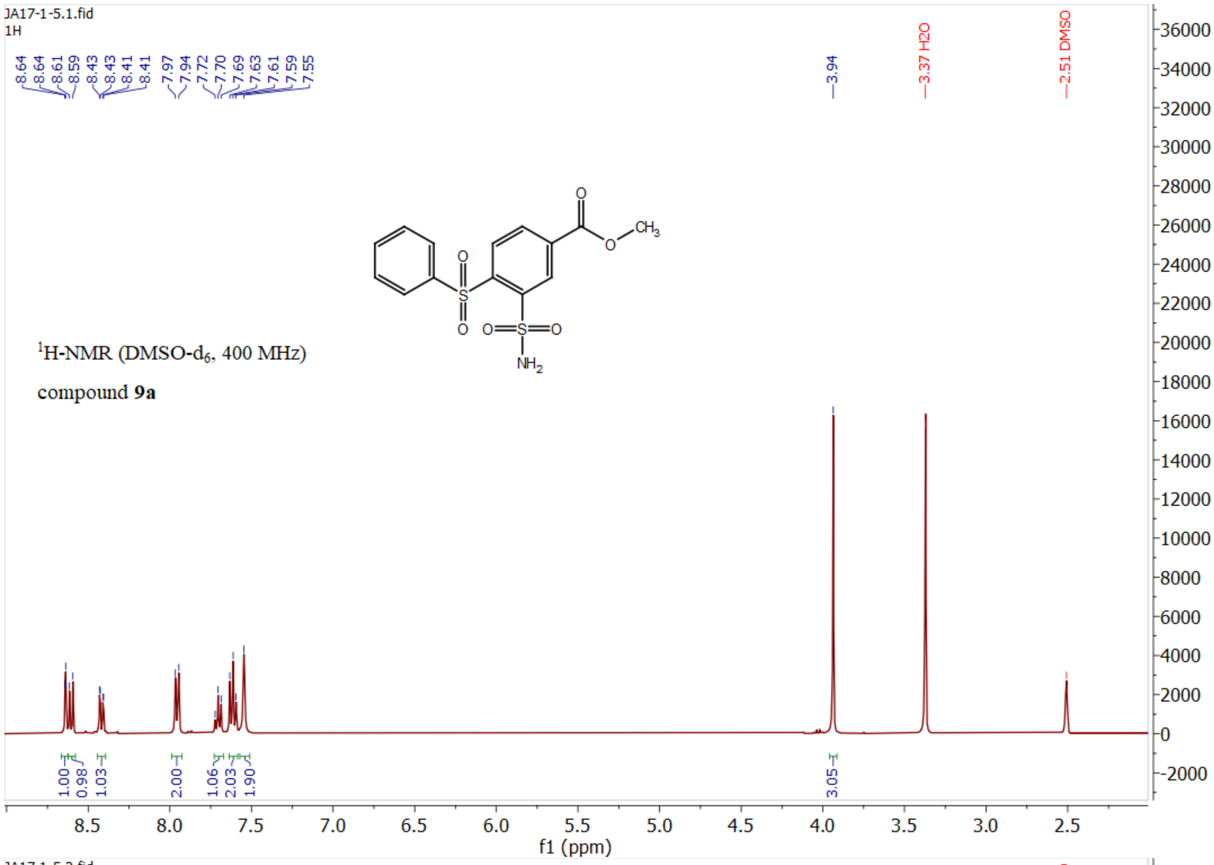
JA17-1-5.1.fid
1H
8.64 8.64 8.61 8.59 8.43 8.43 8.41 8.41 7.97 7.94 7.72 7.70 7.69 7.63 7.61 7.59 7.55
3.94
3.37 H2O
2.51 DMSO
^{1}H-NMR (DMSO-d$_6$, 400 MHz)
compound **9a**
1.00 0.98 1.03 2.00 1.06 2.03 1.90 3.05
f1 (ppm)

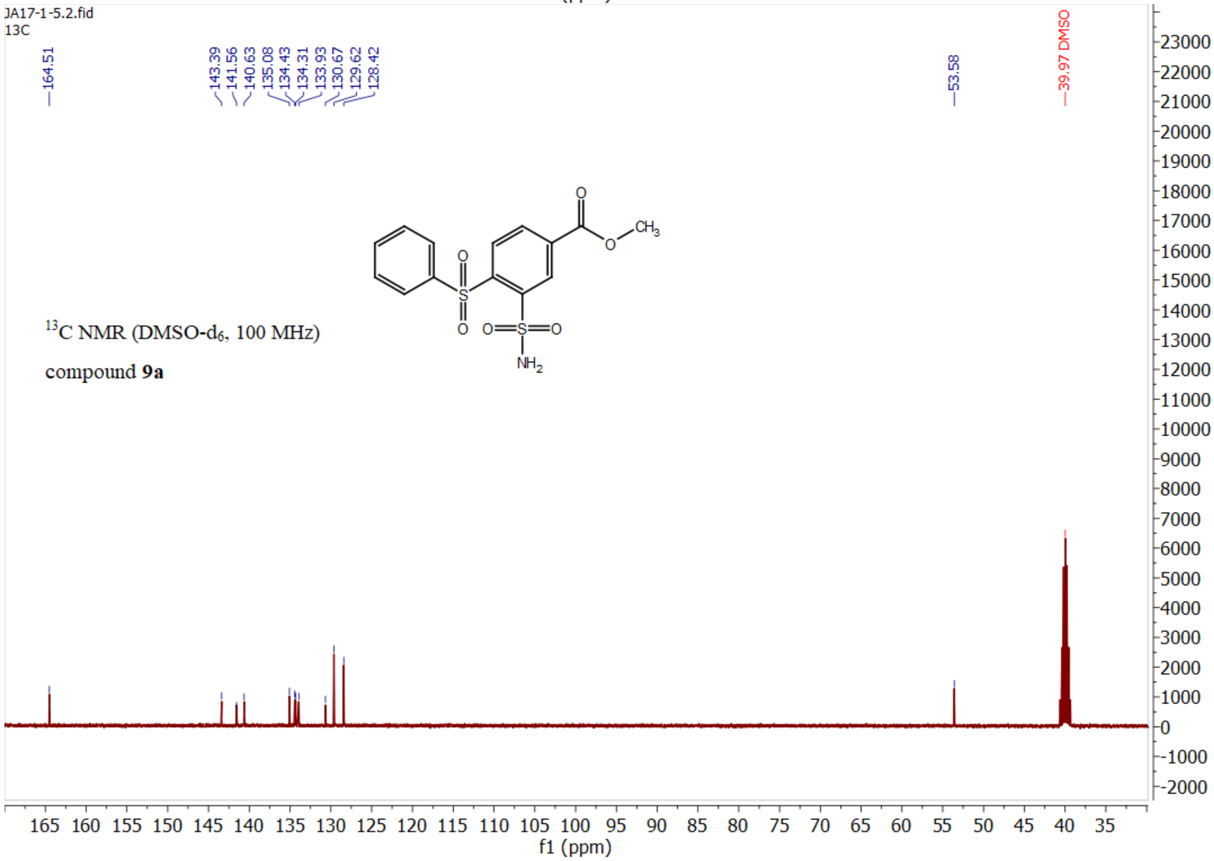
JA17-1-5.2.fid
13C
164.51
143.39 141.56 140.63 135.08 134.43 134.31 133.93 130.67 129.62 128.42
53.58
39.97 DMSO
^{13}C NMR (DMSO-d$_6$, 100 MHz)
compound **9a**
f1 (ppm)

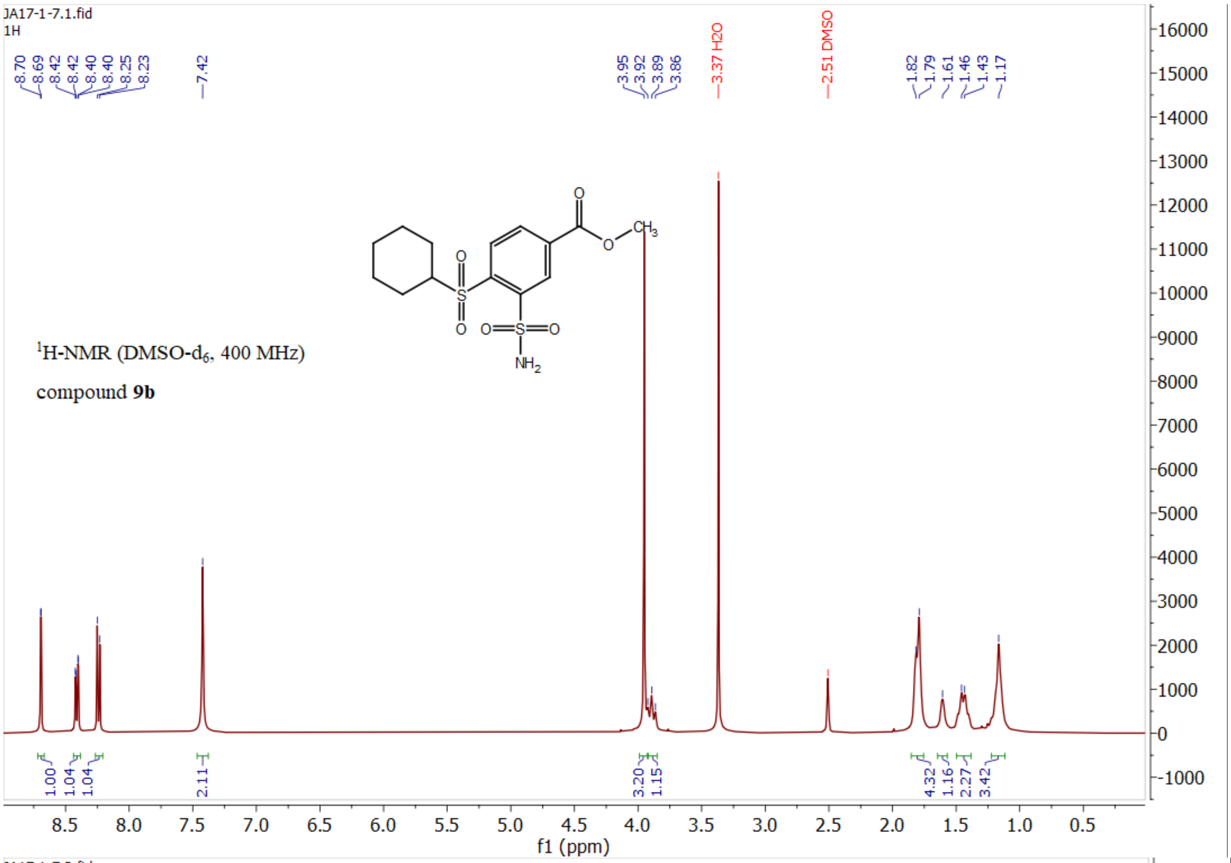
JA17-1-7.1.fid
1H
8.70
8.69
8.42
8.42
8.40
8.40
8.25
8.23
7.42
3.95
3.92
3.89
3.86
3.37 H2O
2.51 DMSO
1.82
1.79
1.61
1.46
1.43
1.17
1H-NMR (DMSO-d6, 400 MHz)
compound 9b
1.00
1.04
1.04
2.11
3.20
1.15
4.32
1.16
2.27
3.42
f1 (ppm)

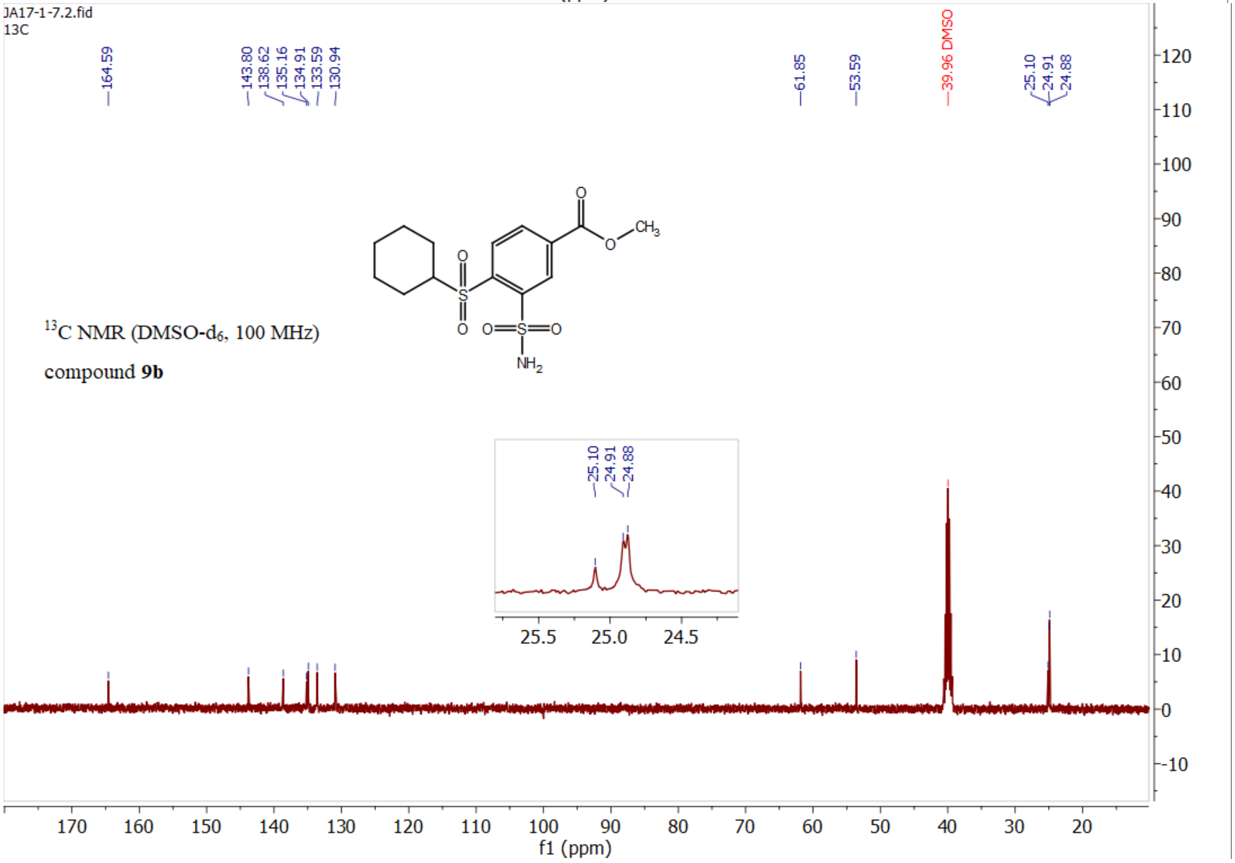
JA17-1-7.2.fid
13C
164.59
143.80
138.62
135.16
134.91
133.59
130.94
61.85
53.59
39.96 DMSO
25.10
24.91
24.88
13C NMR (DMSO-d6, 100 MHz)
compound 9b
f1 (ppm)

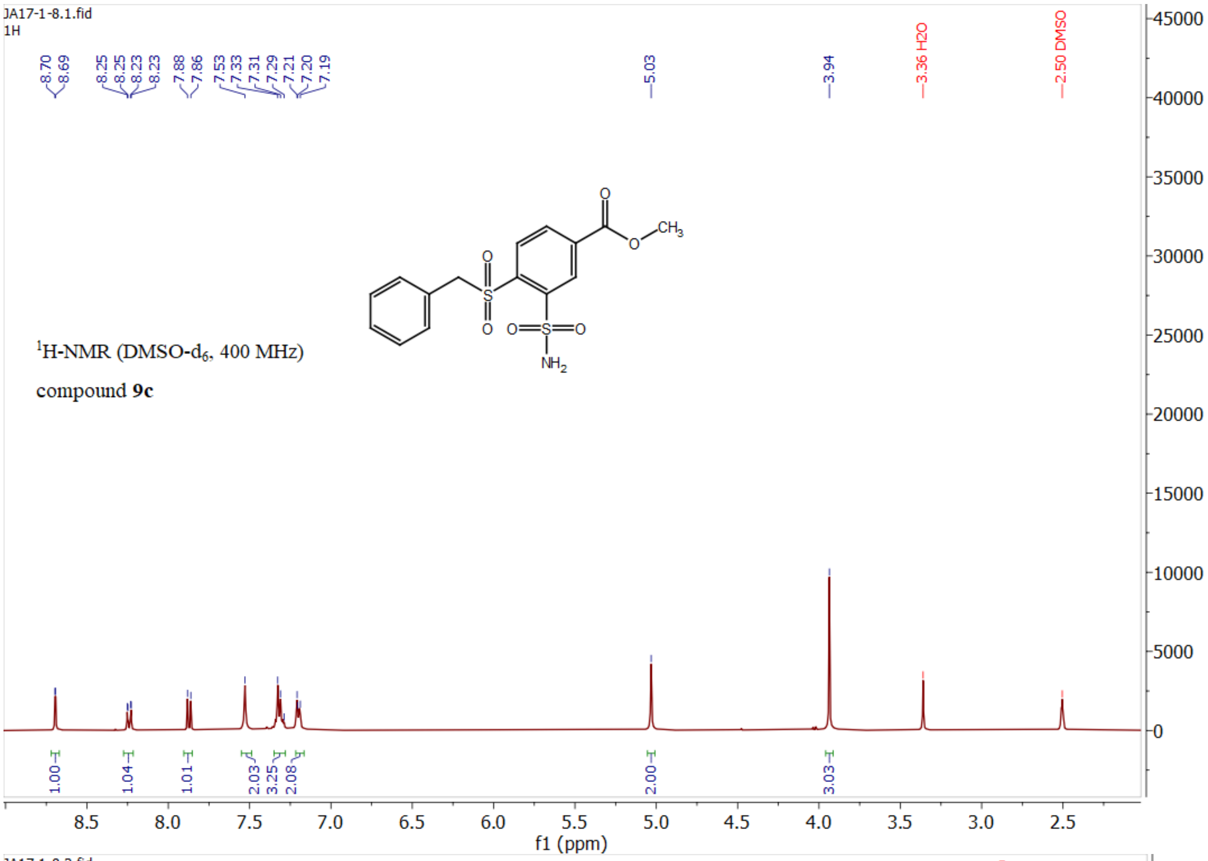
JA17-1-8.1.fid
1H
8.70
8.69
8.25
8.25
8.23
8.23
7.88
7.86
7.53
7.33
7.31
7.29
7.21
7.20
7.19
5.03
3.94
3.36 H2O
2.50 DMSO
O
CH3
O
O
S
O
O
S
O
NH2
^{1}H-NMR (DMSO-d_6, 400 MHz)
compound **9c**
1.00
1.04
1.01
2.03
3.25
2.08
2.00
3.03
f1 (ppm)

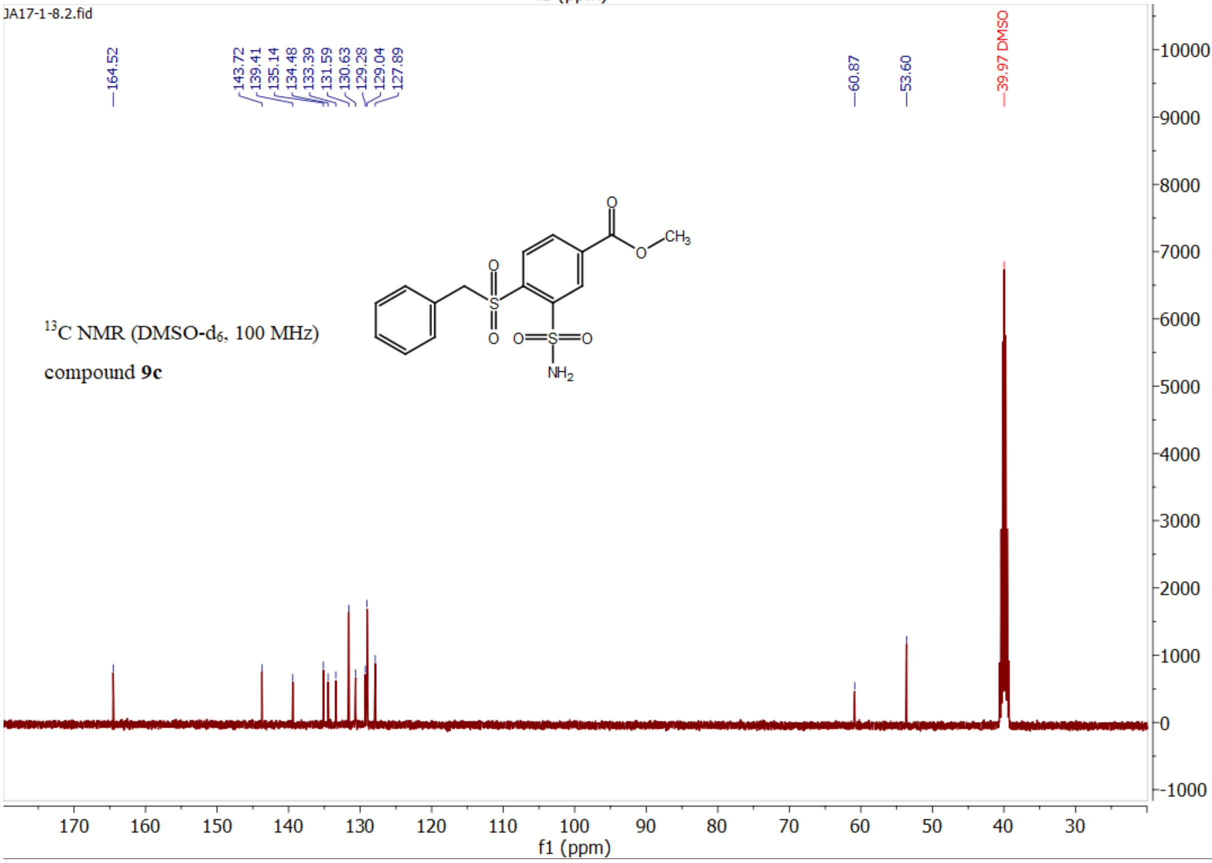
JA17-1-8.2.fid
164.52
143.72
139.41
135.14
134.48
133.39
131.59
130.63
129.28
129.04
127.89
60.87
53.60
39.97 DMSO
^{13}C NMR (DMSO-d_6, 100 MHz)
compound **9c**
f1 (ppm)

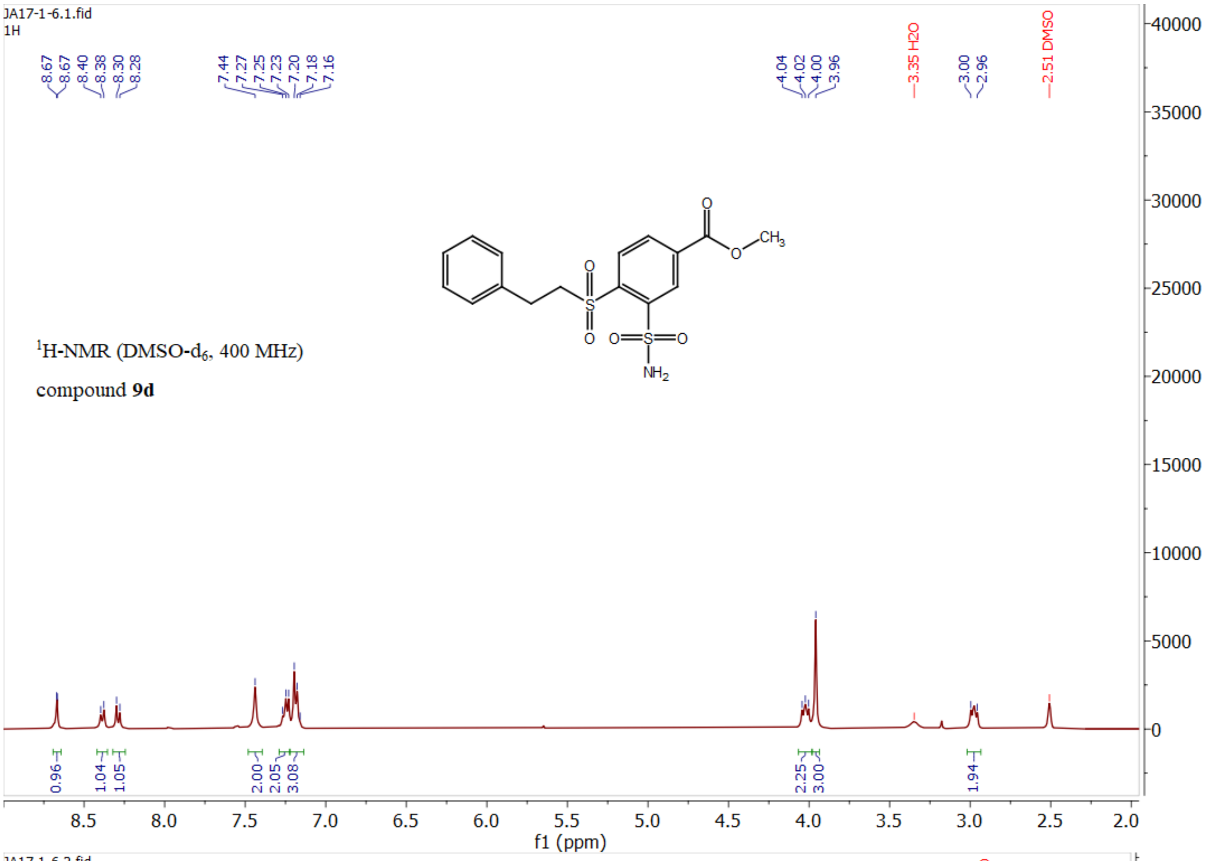
JA17-1-6.1.fid
1H
8.67
8.67
8.40
8.38
8.30
8.28
7.44
7.27
7.25
7.23
7.20
7.18
7.16
4.04
4.02
4.00
3.96
3.35 H2O
3.00
2.96
2.51 DMSO
^{1}H-NMR (DMSO-d$_6$, 400 MHz)
compound **9d**
0.96
1.04
1.05
2.00
2.05
3.08
2.25
3.00
1.94
f1 (ppm)

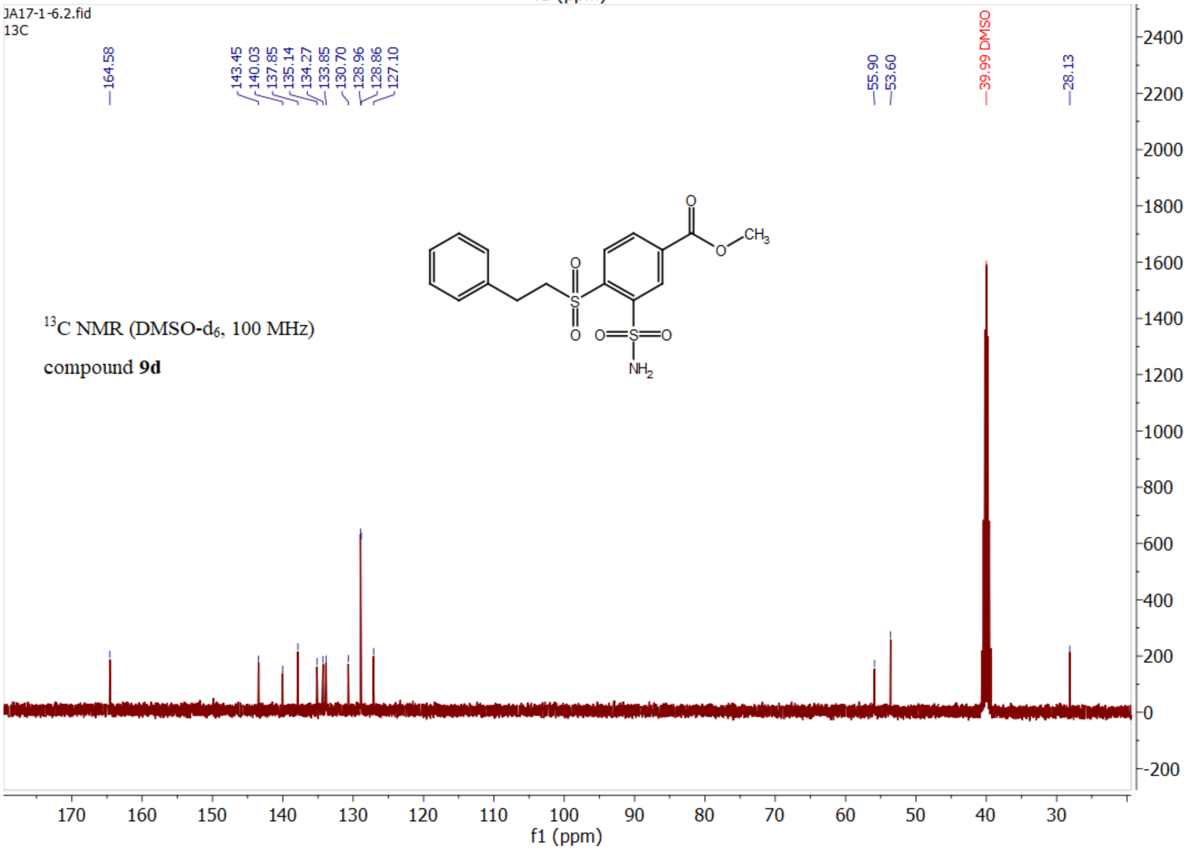
JA17-1-6.2.fid
13C
164.58
143.45
140.03
137.85
135.14
134.27
133.85
130.70
128.96
128.86
127.10
55.90
53.60
39.99 DMSO
28.13
^{13}C NMR (DMSO-d$_6$, 100 MHz)
compound **9d**
f1 (ppm)

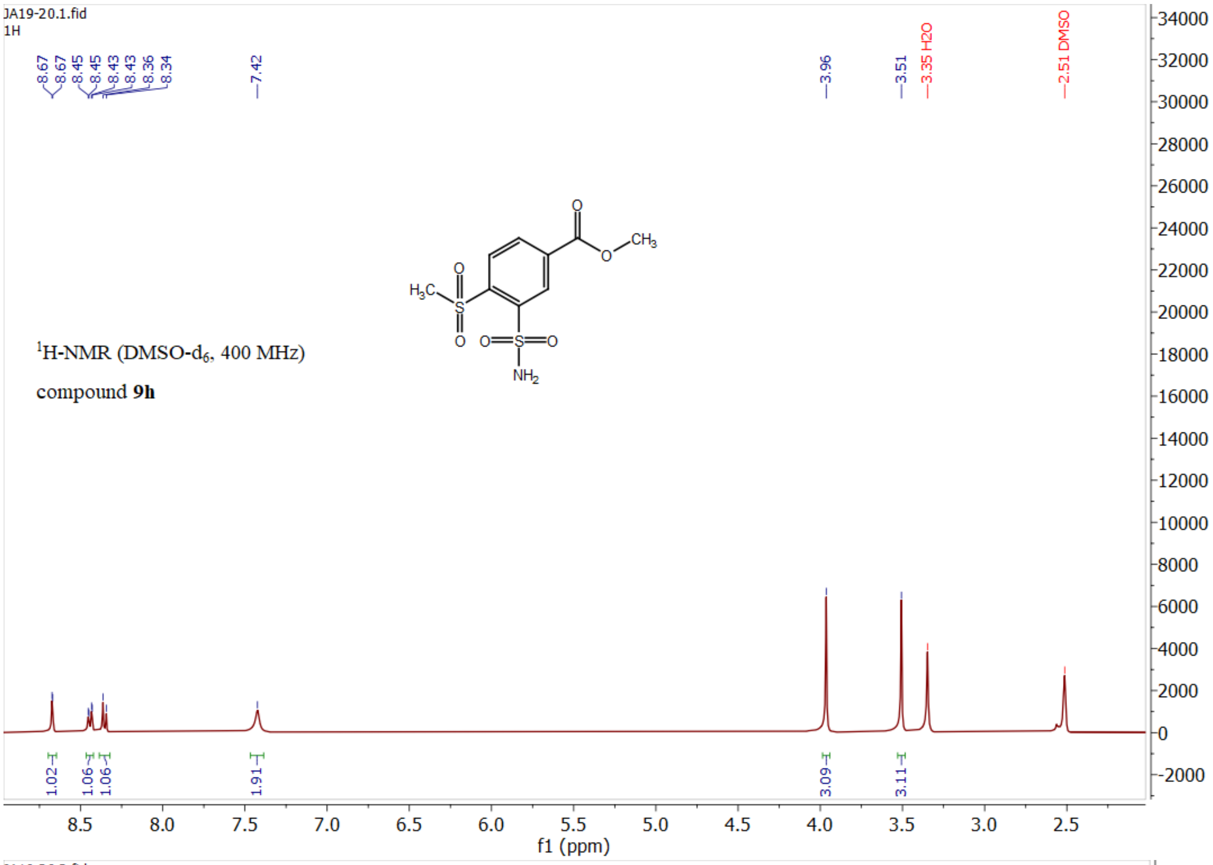
JA19-20.1.fid
1H
8.67
8.67
8.45
8.45
8.43
8.43
8.36
8.34
7.42
3.96
3.51
3.35 H2O
2.51 DMSO
1H-NMR (DMSO-d6, 400 MHz)
compound 9h
1.02
1.06
1.06
1.91
3.09
3.11
f1 (ppm)

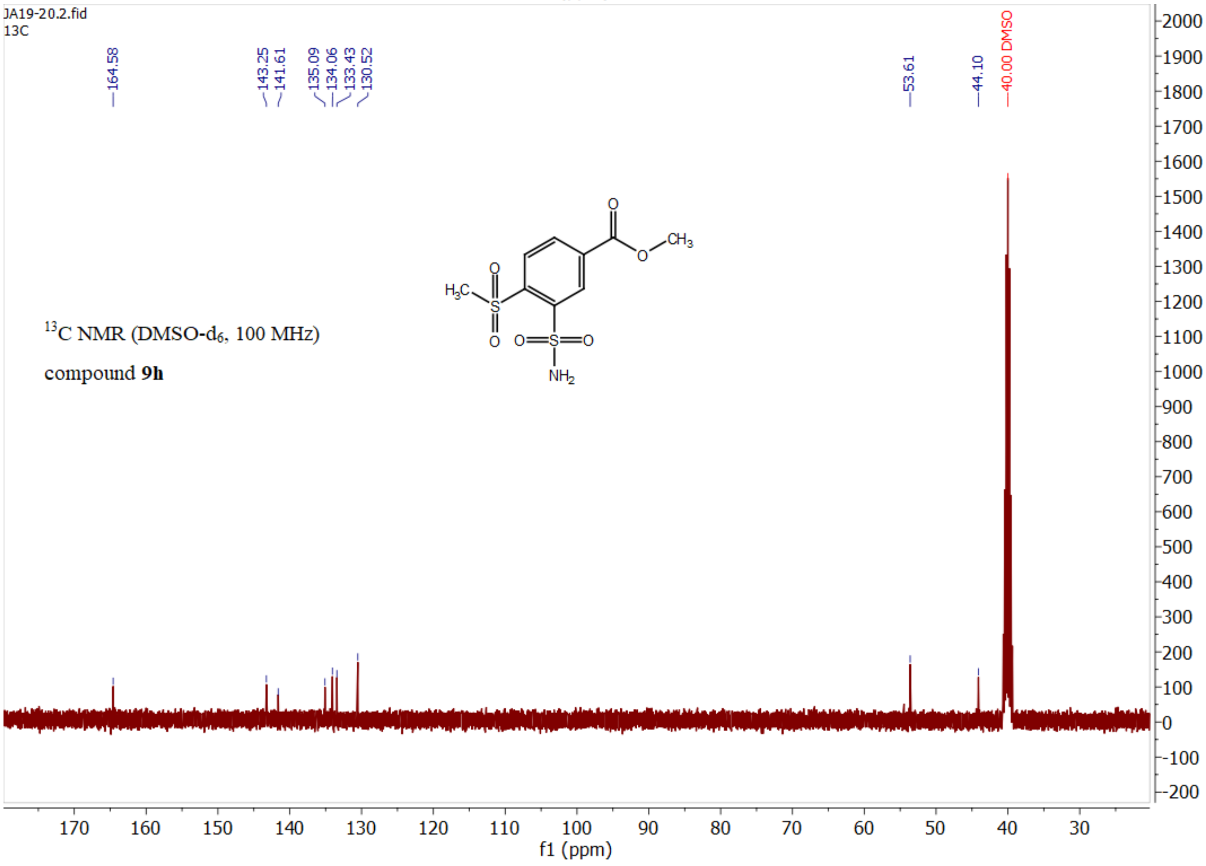
JA19-20.2.fid
13C
164.58
143.25
141.61
135.09
134.06
133.43
130.52
53.61
44.10
40.00 DMSO
13C NMR (DMSO-d6, 100 MHz)
compound 9h
f1 (ppm)

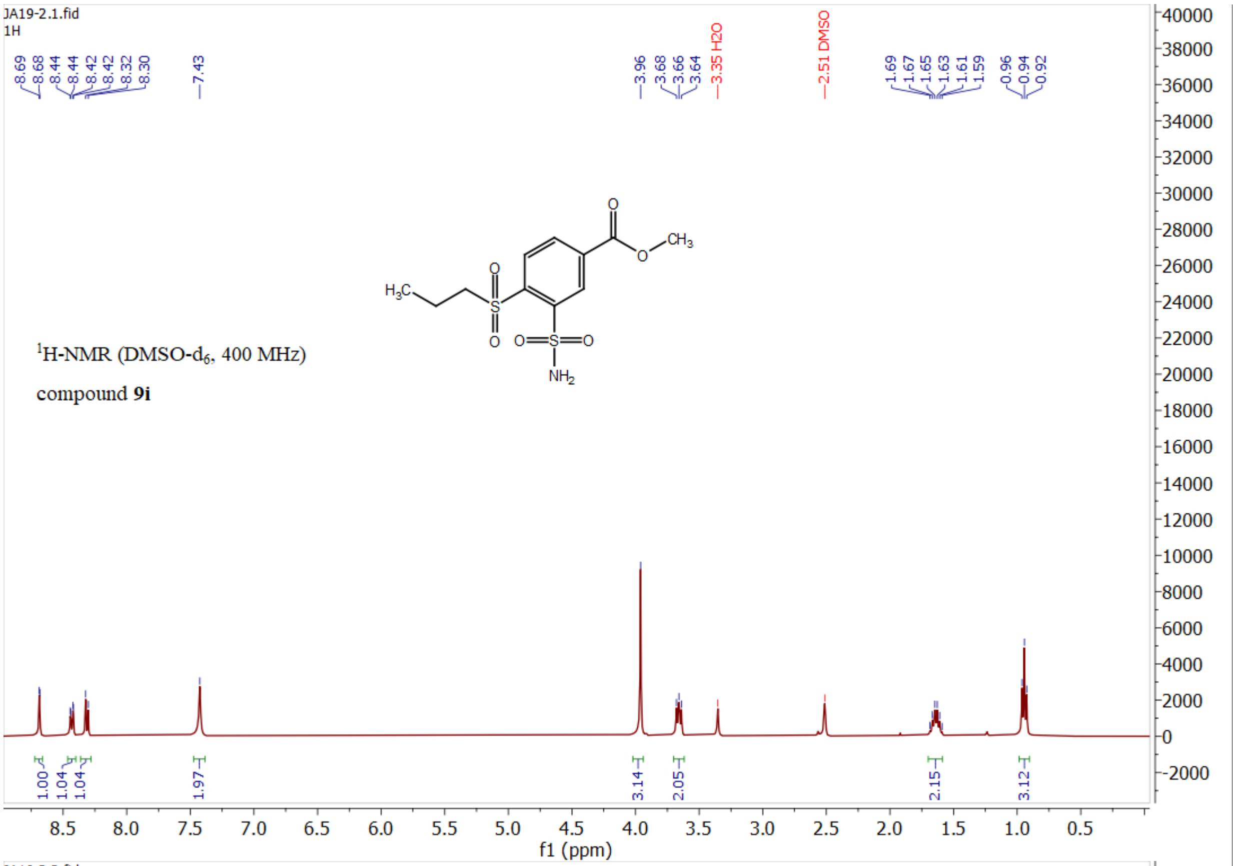
JA19-2.1.fid
1H
8.69
8.68
8.44
8.44
8.42
8.42
8.32
8.30
7.43
3.96
3.68
3.66
3.64
3.35 H2O
2.51 DMSO
1.69
1.67
1.65
1.63
1.61
1.59
0.96
0.94
0.92
1H-NMR (DMSO-d6, 400 MHz)
compound 9i
1.00
1.04
1.04
1.97
3.14
2.05
2.15
3.12
f1 (ppm)

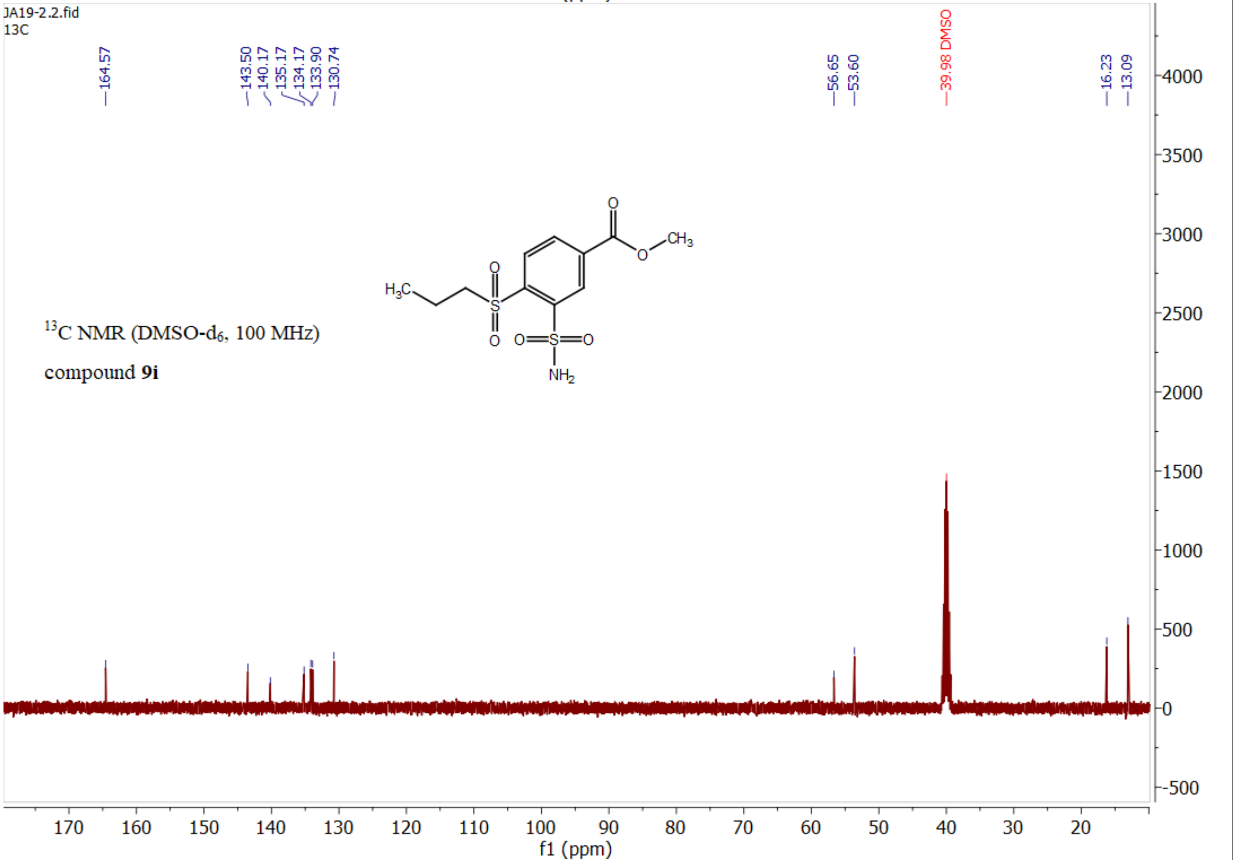
JA19-2.2.fid
13C
164.57
143.50
140.17
135.17
134.17
133.90
130.74
56.65
53.60
39.98 DMSO
16.23
13.09
13C NMR (DMSO-d6, 100 MHz)
compound 9i
f1 (ppm)

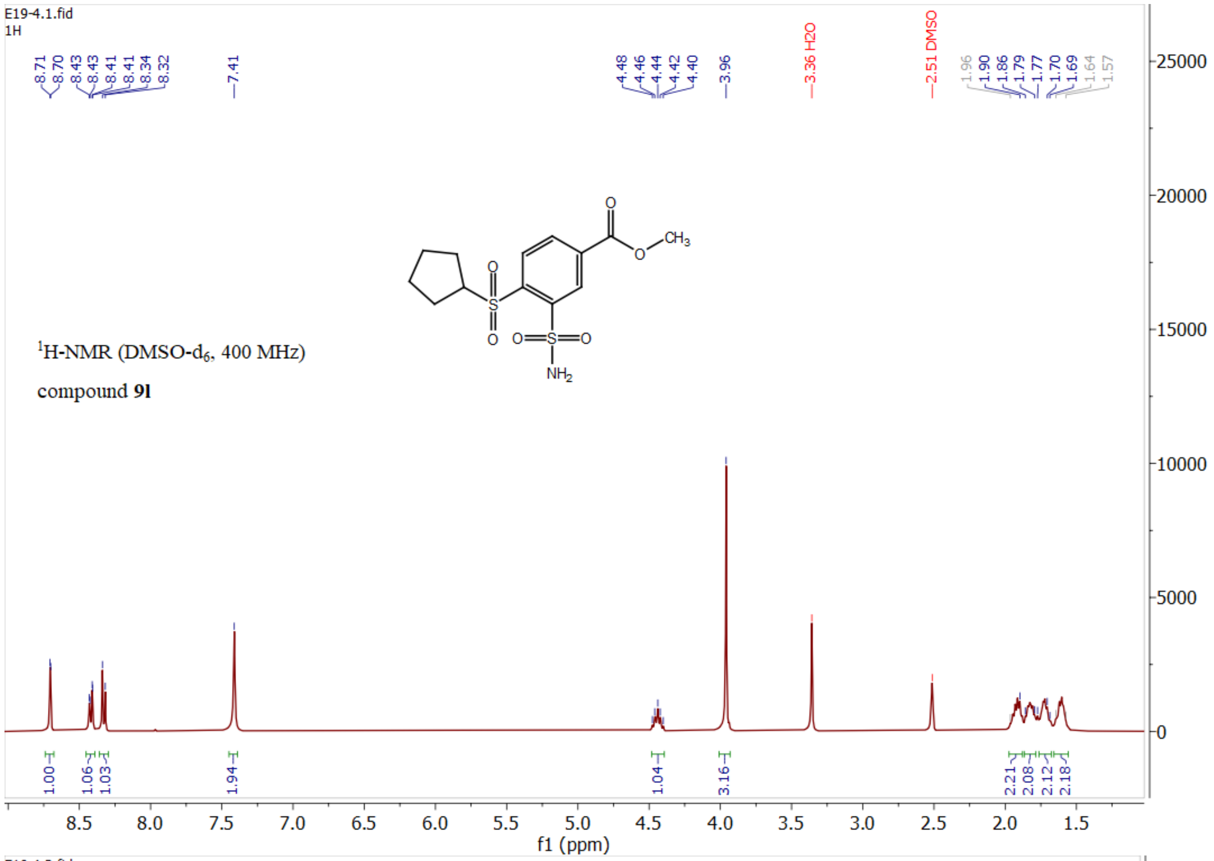
E19-4.1.fid
1H
8.71
8.70
8.43
8.43
8.41
8.41
8.34
8.32
7.41
4.48
4.46
4.44
4.42
4.40
3.96
3.36 H2O
2.51 DMSO
1.96
1.90
1.86
1.79
1.77
1.70
1.69
1.64
1.57
^{1}H-NMR (DMSO-d$_6$, 400 MHz)
compound **9l**
1.00
1.06
1.03
1.94
1.04
3.16
2.21
2.08
2.12
2.18
f1 (ppm)

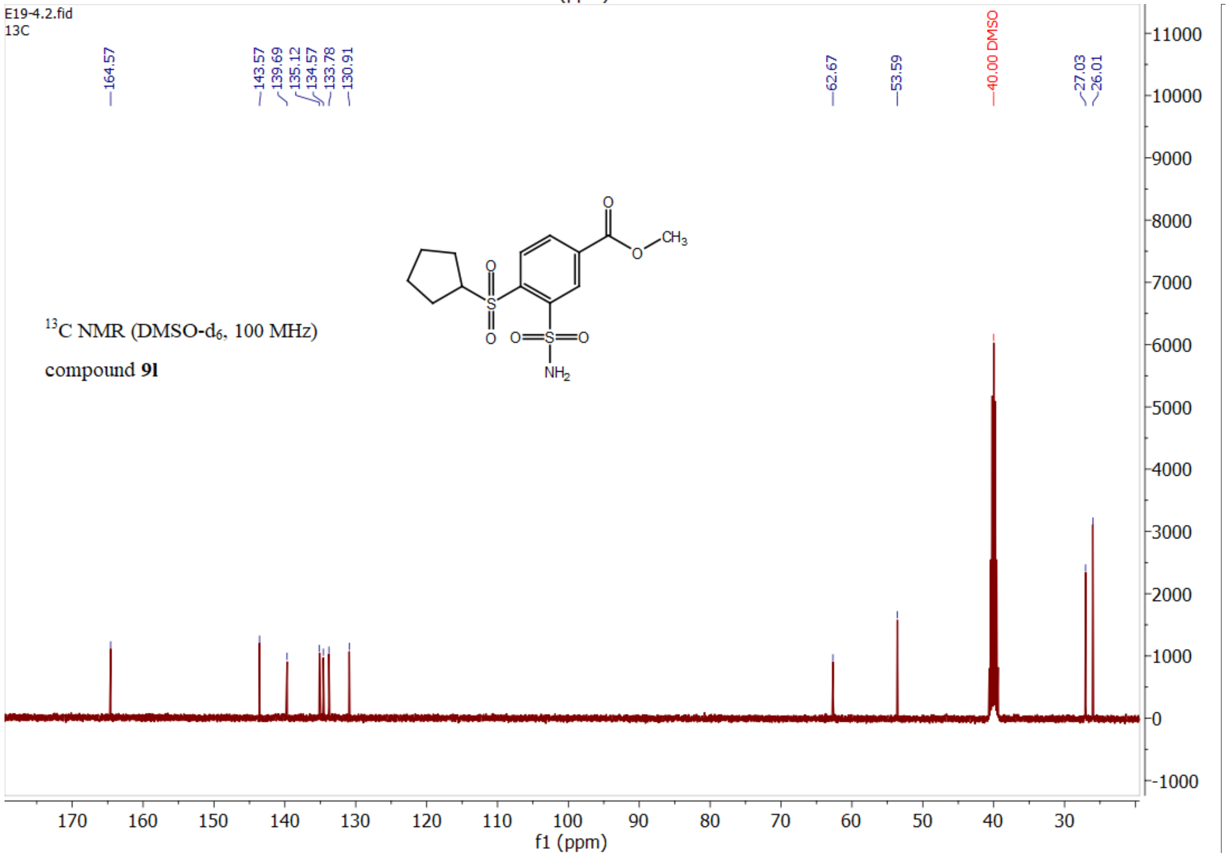
E19-4.2.fid
13C
164.57
143.57
139.69
135.12
134.57
133.78
130.91
62.67
53.59
40.00 DMSO
27.03
26.01
^{13}C NMR (DMSO-d$_6$, 100 MHz)
compound **9l**
f1 (ppm)

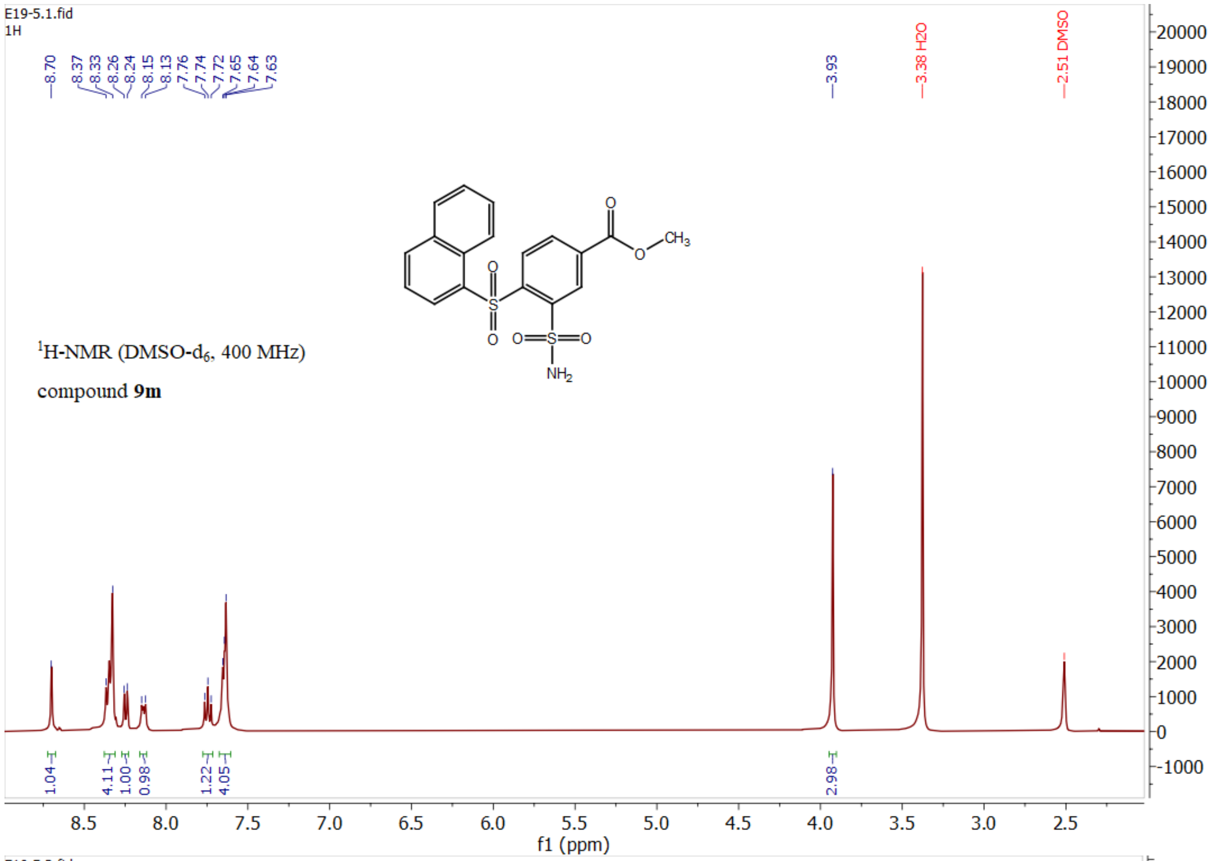
E19-5.1.fid
1H
8.70
8.37
8.33
8.26
8.24
8.15
8.13
7.76
7.74
7.72
7.65
7.64
7.63
3.93
3.38 H2O
2.51 DMSO
1H-NMR (DMSO-d6, 400 MHz)
compound 9m
1.04
4.11
1.00
0.98
1.22
4.05
2.98
f1 (ppm)

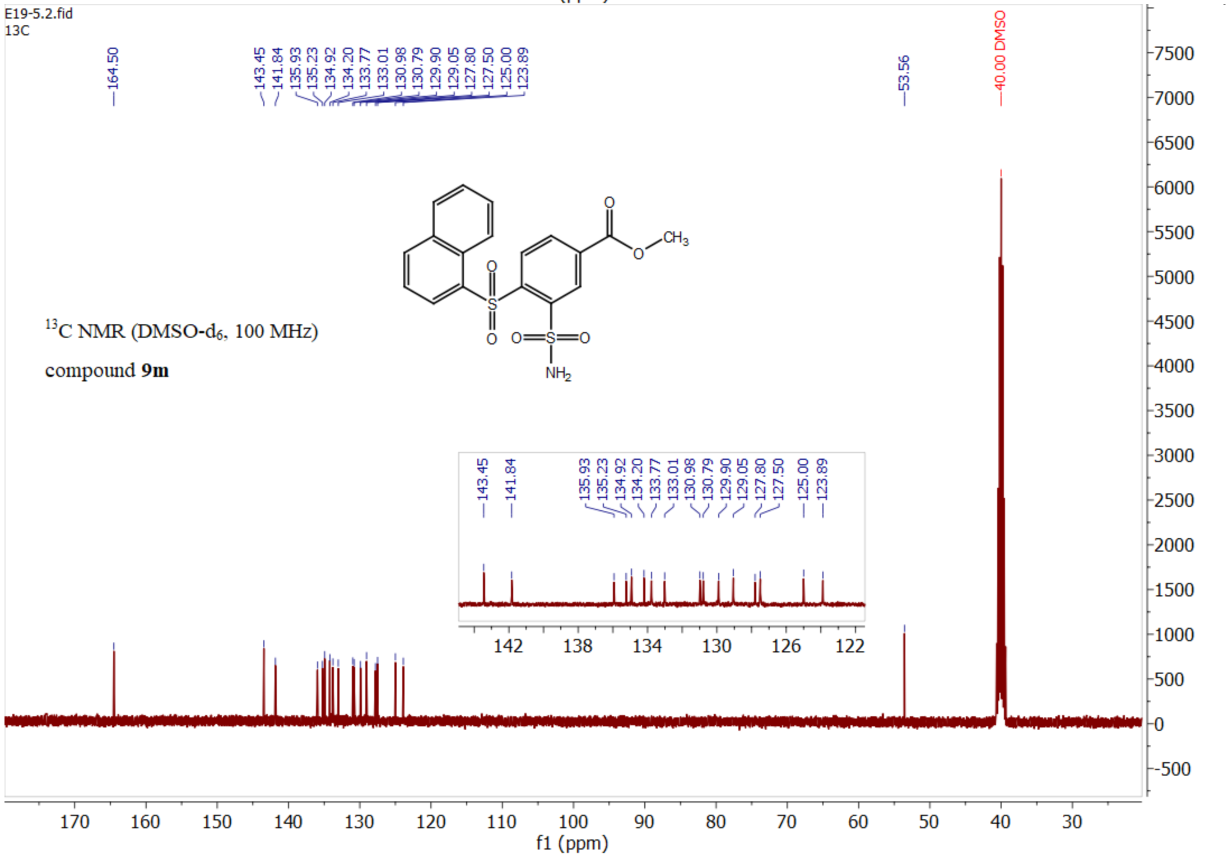
E19-5.2.fid
13C
164.50
143.45
141.84
135.93
135.23
134.92
134.20
133.77
133.01
130.98
130.79
129.90
129.05
127.80
127.50
125.00
123.89
53.56
40.00 DMSO
13C NMR (DMSO-d6, 100 MHz)
compound 9m
f1 (ppm)

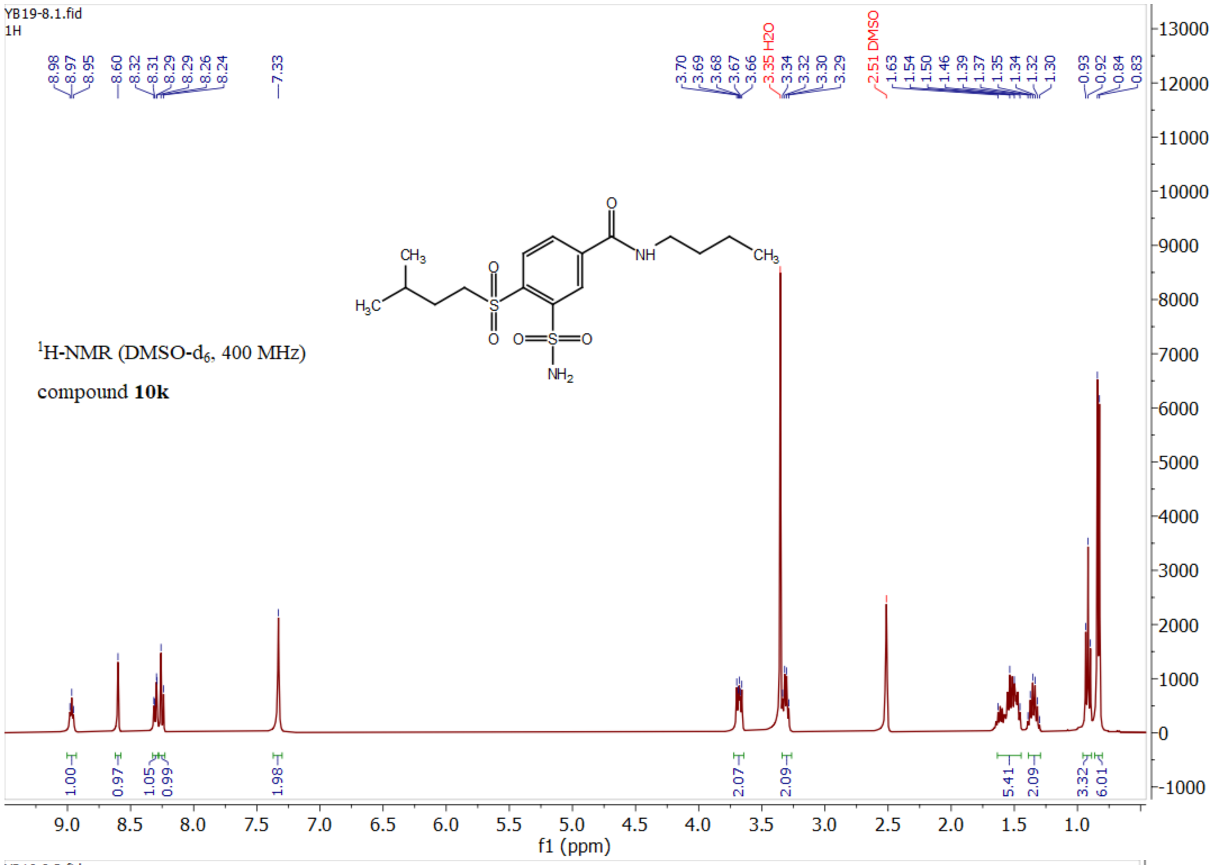
YB19-8.1.fid
1H
^{1}H-NMR (DMSO-d_6, 400 MHz)
compound **10k**
8.98
8.97
8.95
8.60
8.32
8.31
8.29
8.29
8.26
8.24
7.33
3.70
3.69
3.68
3.67
3.66
3.35 H2O
3.34
3.32
3.30
3.29
2.51 DMSO
1.63
1.54
1.50
1.46
1.39
1.37
1.35
1.34
1.32
1.30
0.93
0.92
0.84
0.83
1.00
0.97
1.05
0.99
1.98
2.07
2.09
5.41
2.09
3.32
6.01
f1 (ppm)

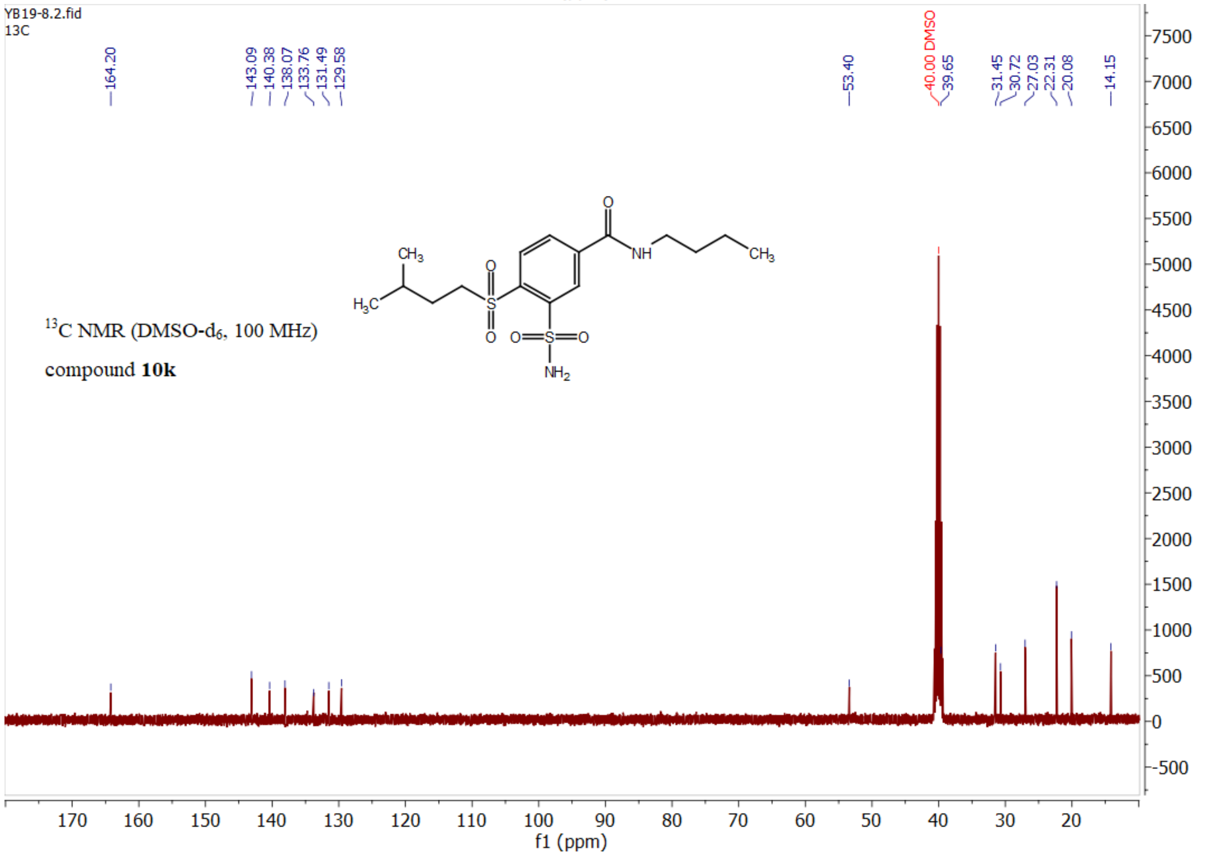
YB19-8.2.fid
13C
^{13}C NMR (DMSO-d_6, 100 MHz)
compound **10k**
164.20
143.09
140.38
138.07
133.76
131.49
129.58
53.40
40.00 DMSO
39.65
31.45
30.72
27.03
22.31
20.08
14.15
f1 (ppm)

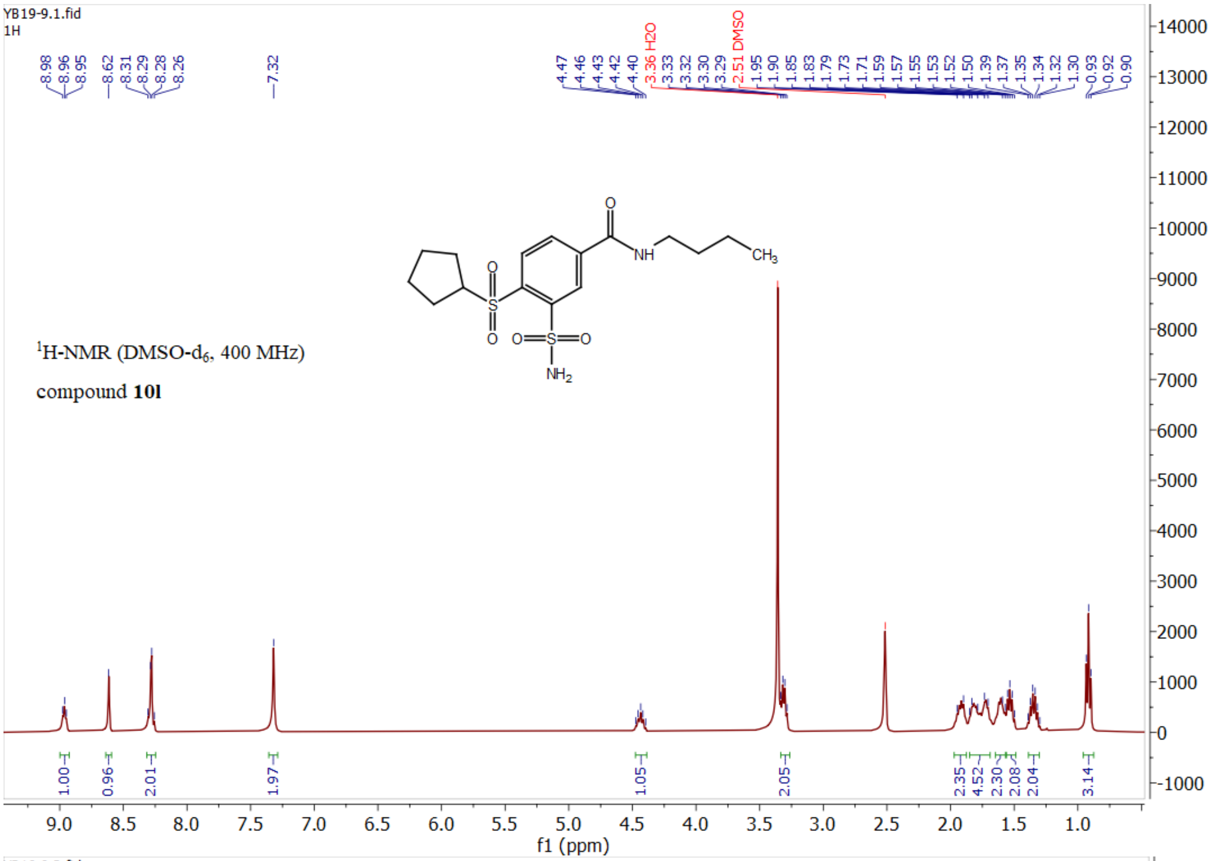
YB19-9.1.fid
1H
^{1}H-NMR (DMSO-d6, 400 MHz)
compound 10l
f1 (ppm)

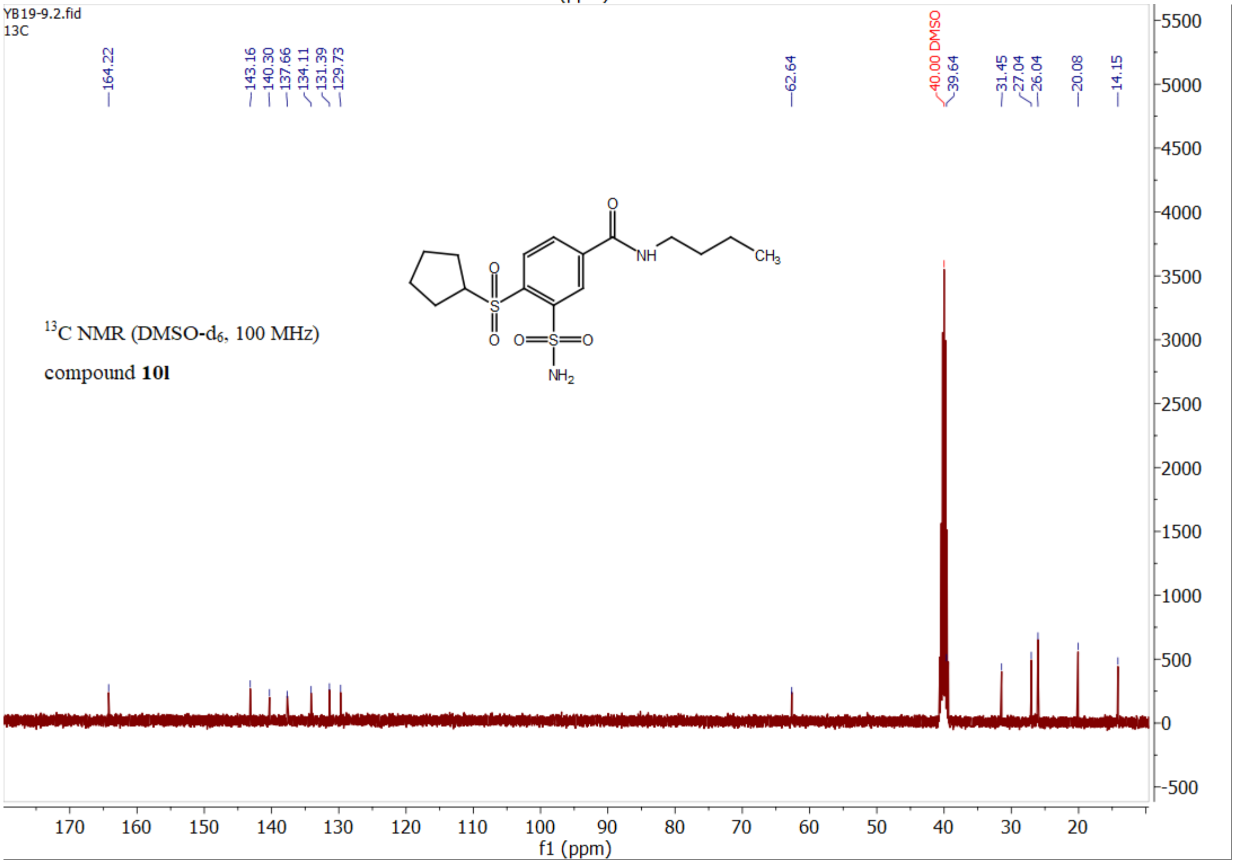
YB19-9.2.fid
13C
^{13}C NMR (DMSO-d6, 100 MHz)
compound 10l
f1 (ppm)

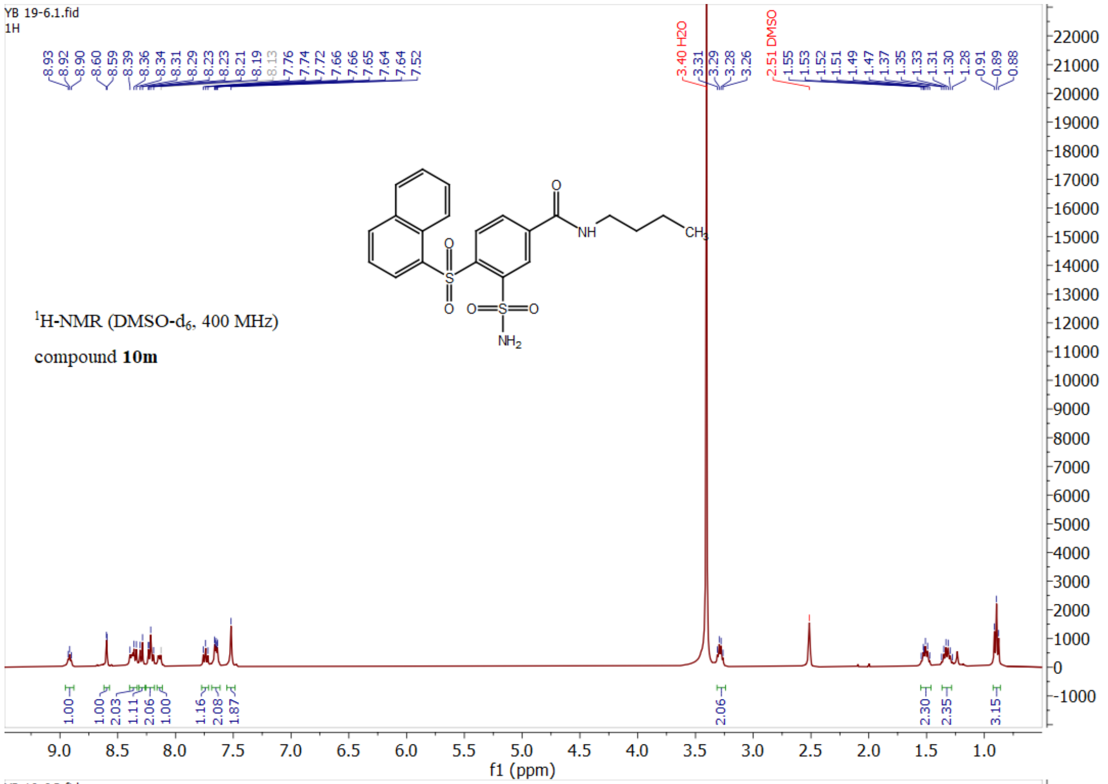

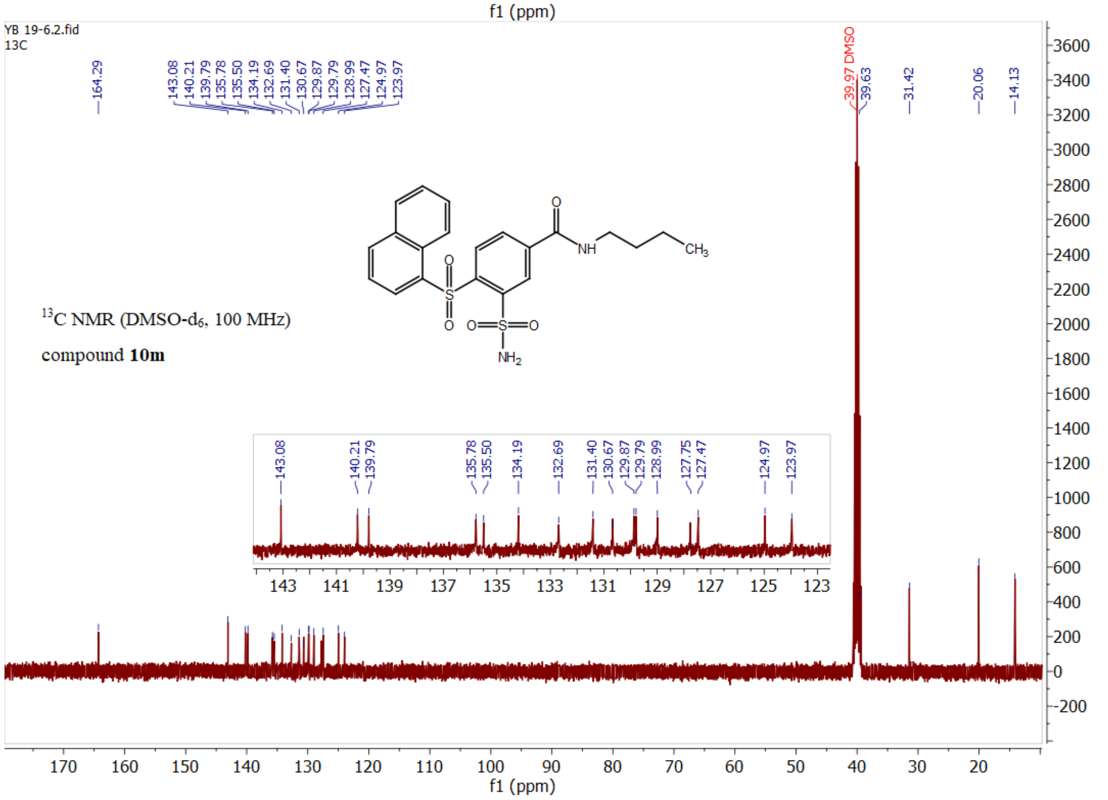

Figure S7. ^{1}H and ^{13}C NMR spectra of synthesized compounds. ^{1}H NMR (DMSO and H_2O) and ^{13}C (DMSO-d$_6$) solvent residual peaks are presented in the graphs. For descriptions of the remaining compounds, see the Experimental section.

HPLC chromatograms of representative compounds

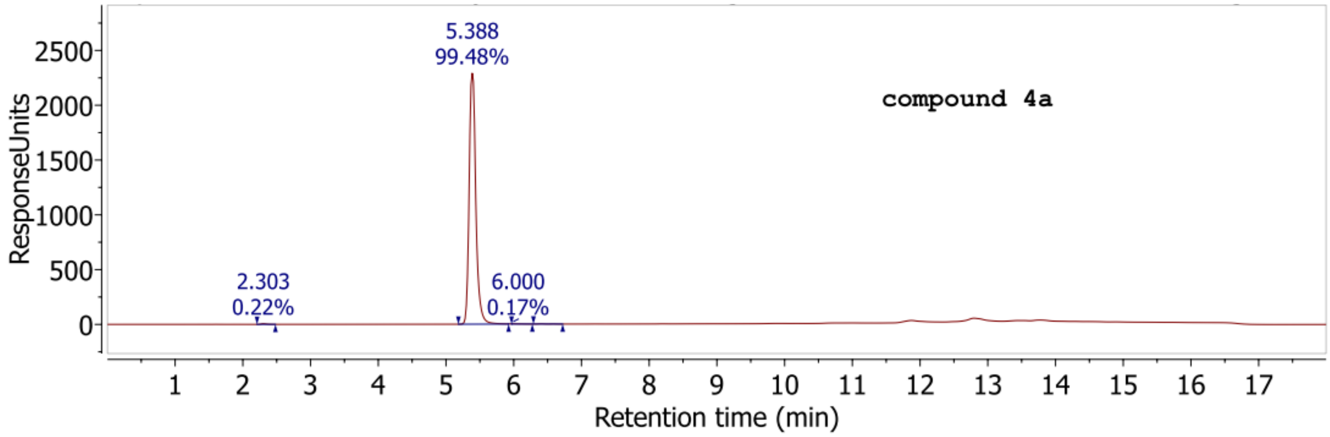

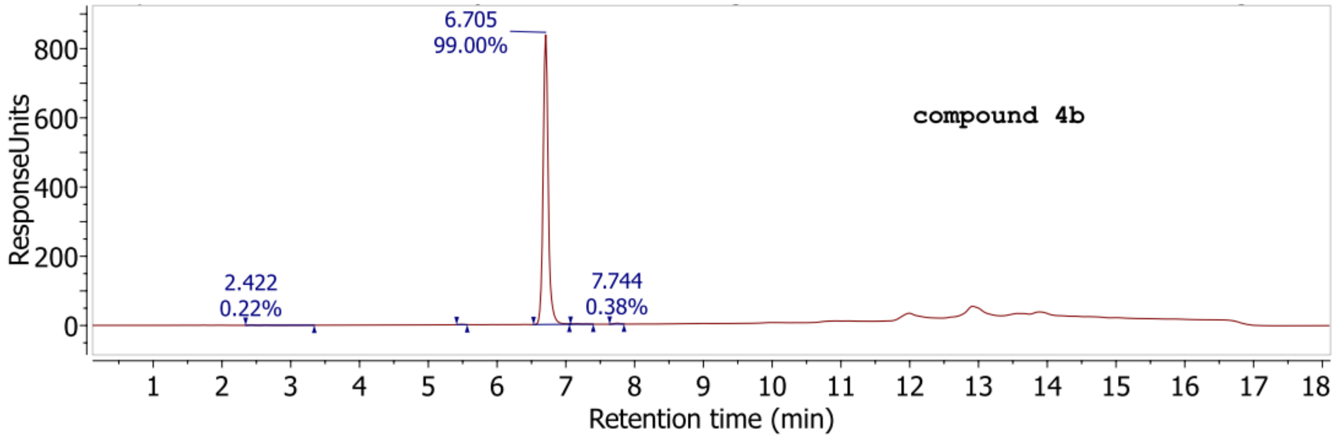

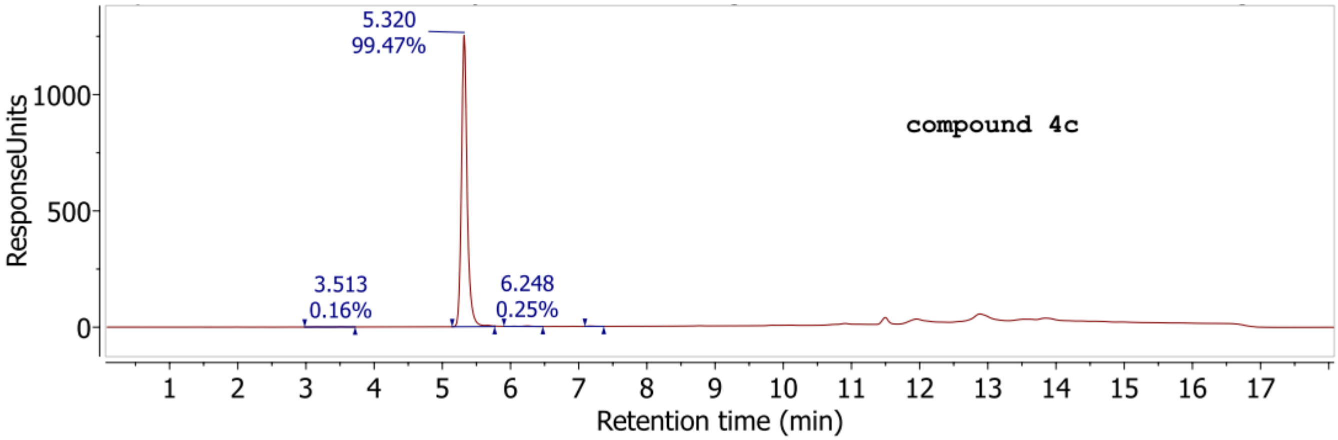

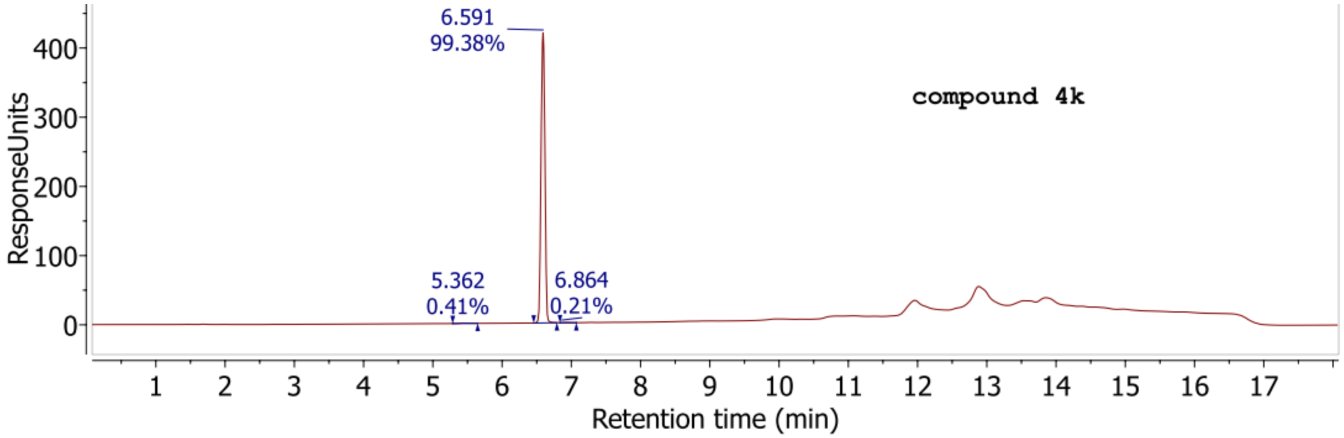
6.591
99.38%
5.362
0.41%
6.864
0.21%
compound 4k
ResponseUnits
Retention time (min)

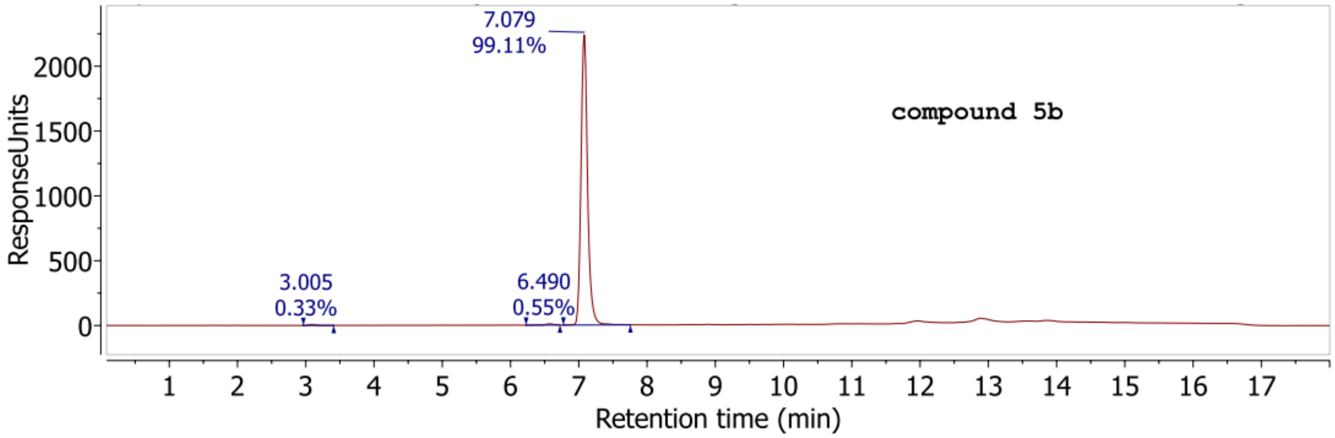
7.079
99.11%
3.005
0.33%
6.490
0.55%
compound 5b
ResponseUnits
Retention time (min)

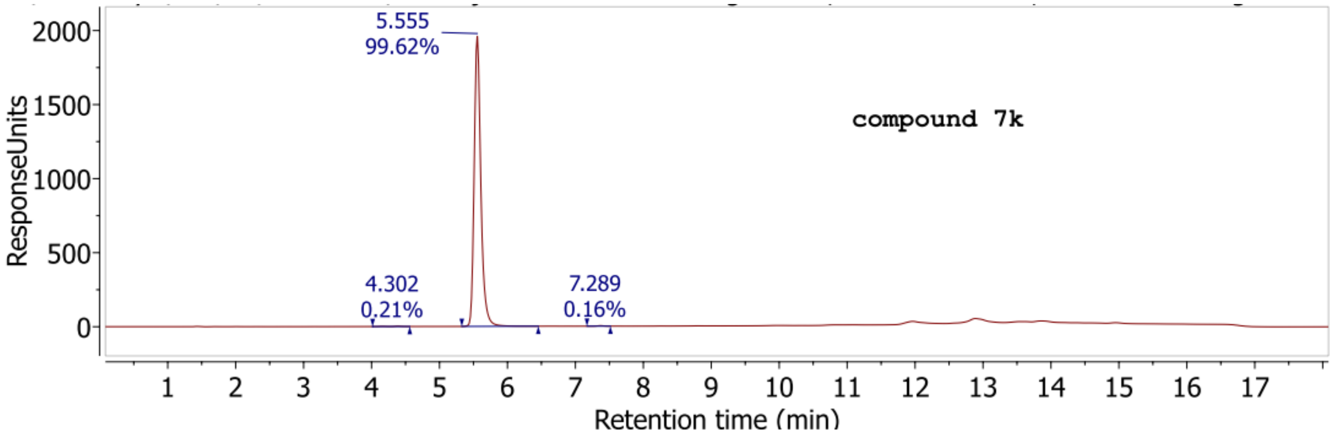
5.555
99.62%
4.302
0.21%
7.289
0.16%
compound 7k
ResponseUnits
Retention time (min)

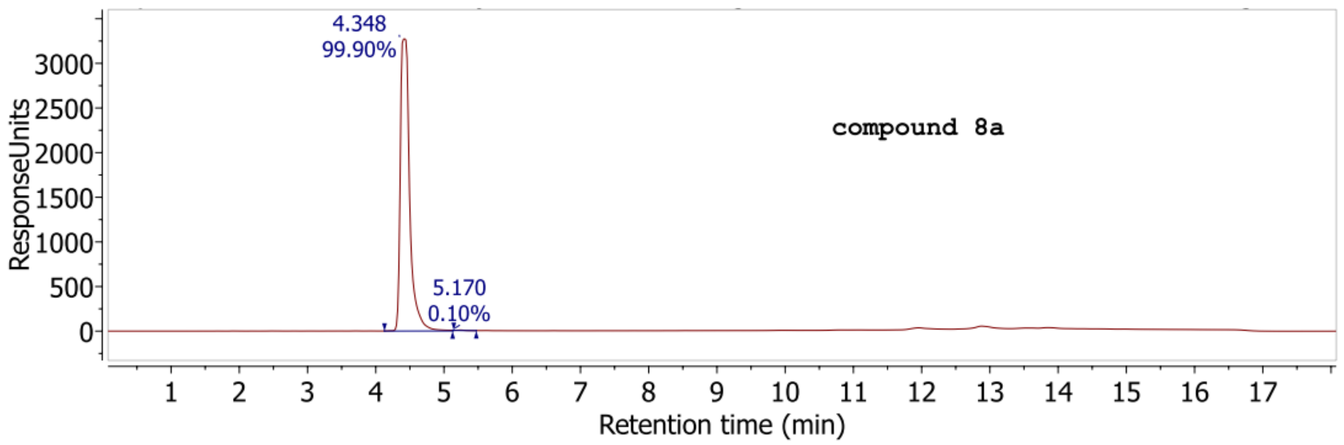

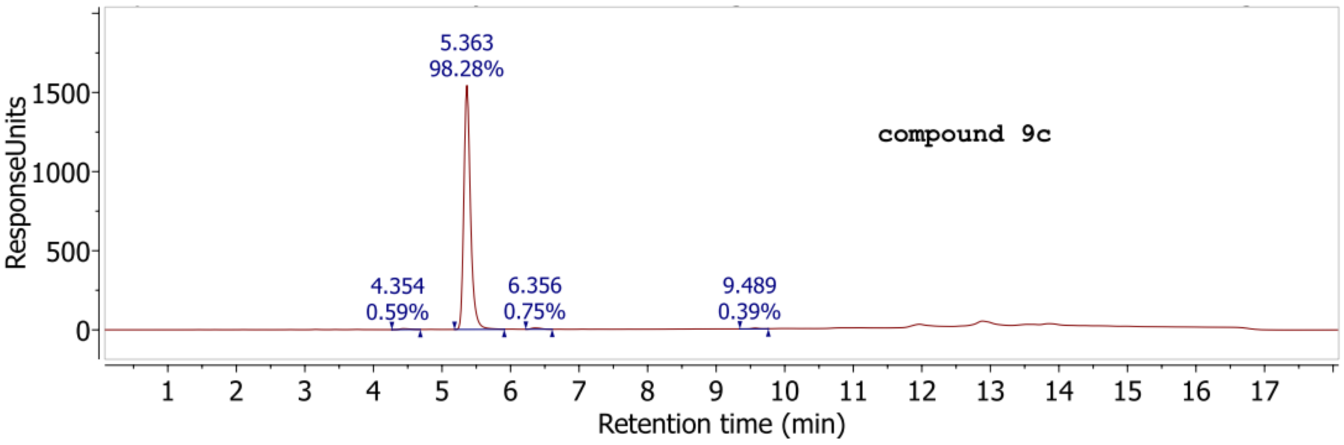

Figure S8. HPLC chromatograms for purity determination of selected compounds **4a**, **4b**, **4c**, **4k**, **5b**, **7k**, **8a**, and **9c**. UV detection was recorded at 254 nm.

ESI-MS spectra of representative compounds

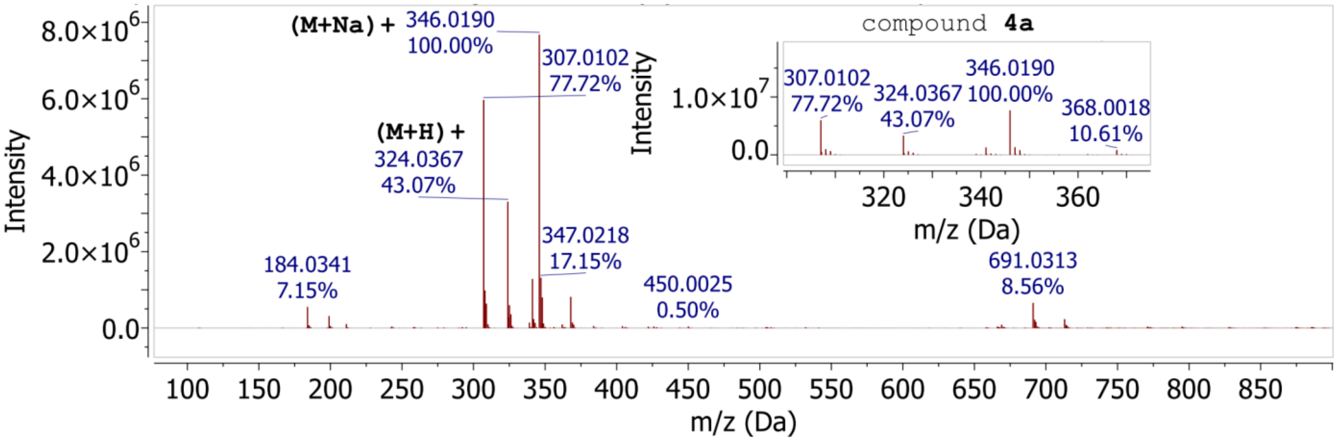

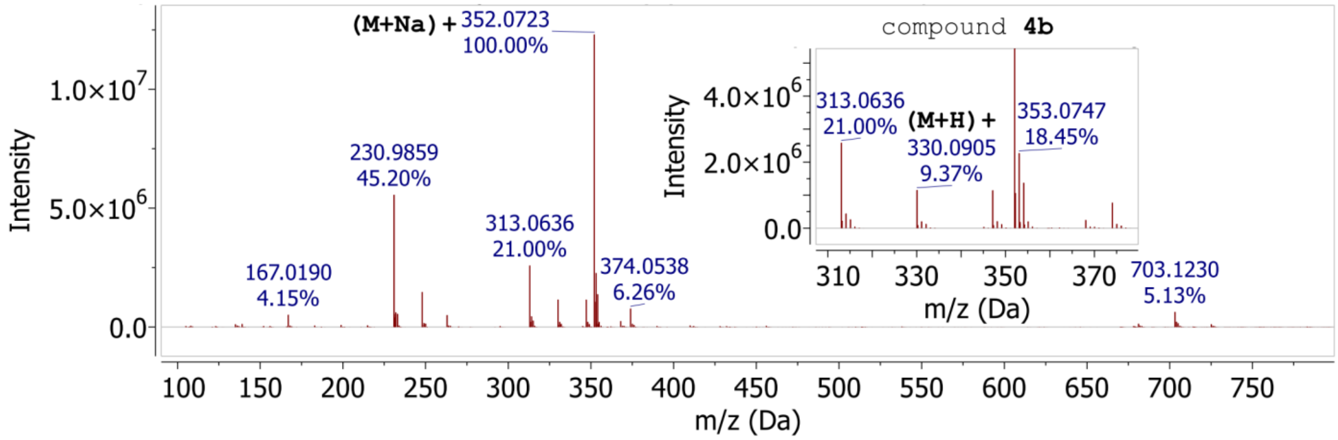

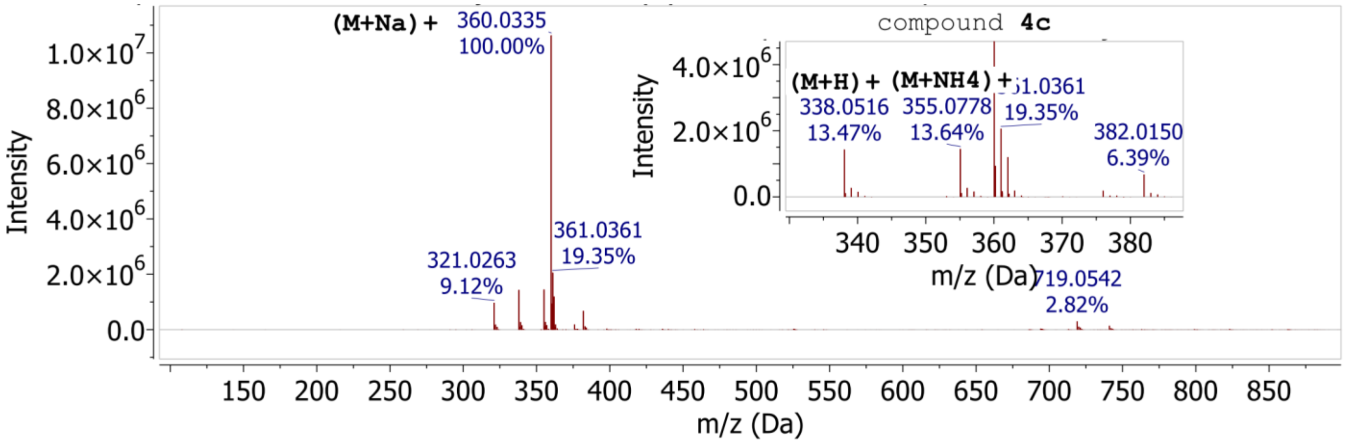

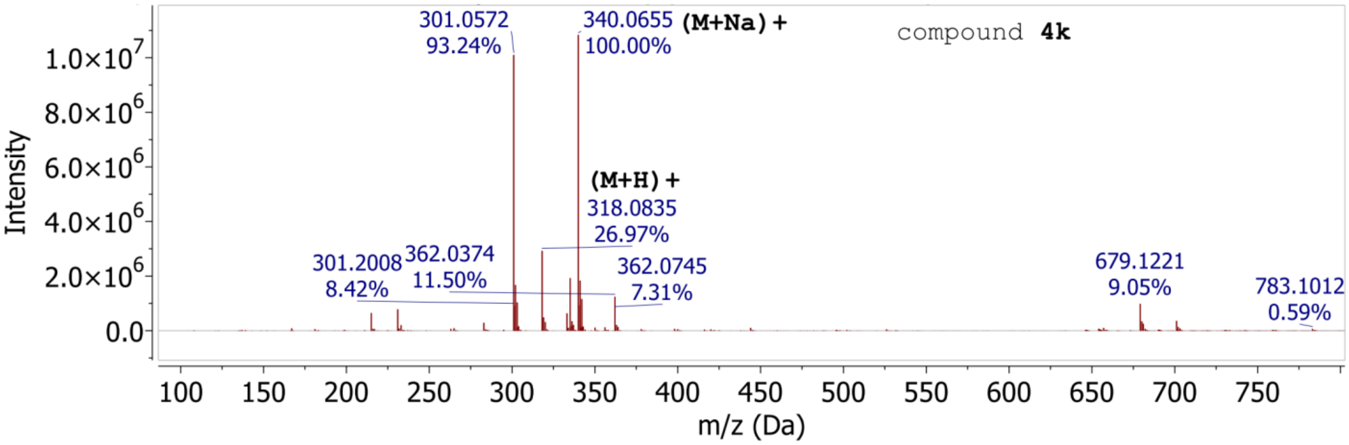
compound 4k
(M+Na)+
340.0655
100.00%
301.0572
93.24%
(M+H)+
318.0835
26.97%
301.2008
8.42%
362.0374
11.50%
362.0745
7.31%
679.1221
9.05%
783.1012
0.59%
Intensity
m/z (Da)

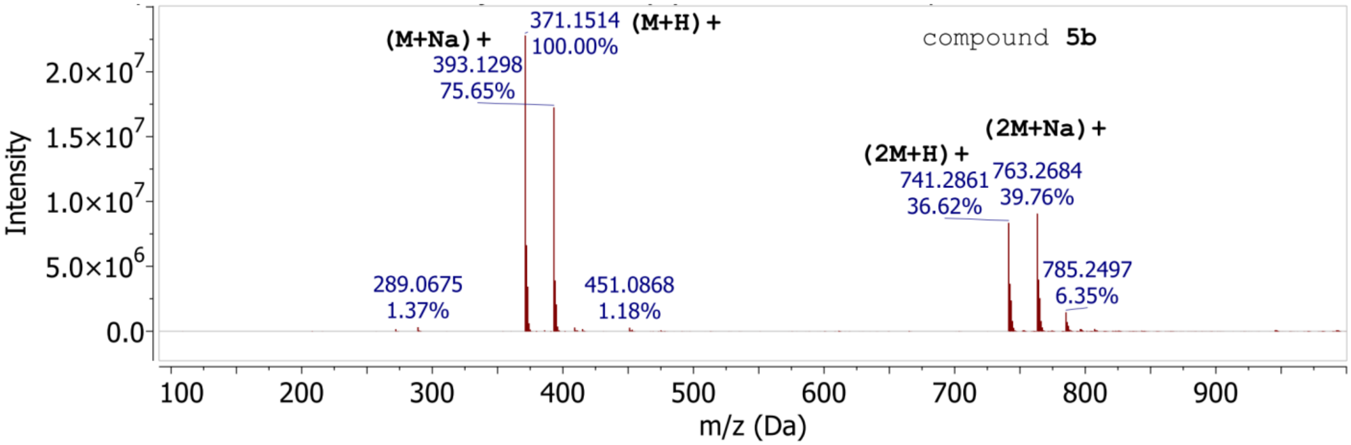
compound 5b
371.1514
100.00%
(M+H)+
(M+Na)+
393.1298
75.65%
(2M+H)+
741.2861
36.62%
(2M+Na)+
763.2684
39.76%
785.2497
6.35%
289.0675
1.37%
451.0868
1.18%
Intensity
m/z (Da)

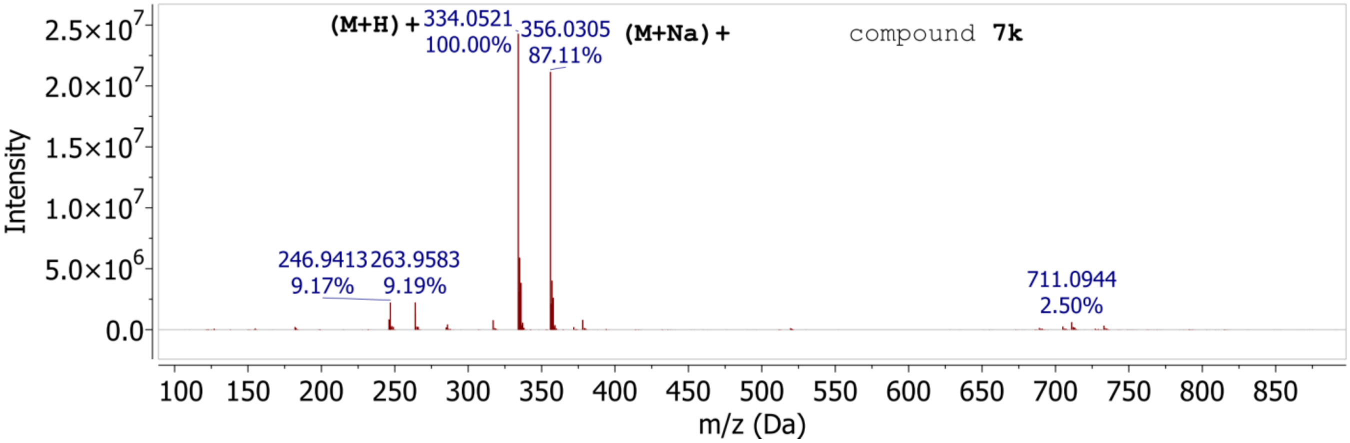
compound 7k
(M+H)+
334.0521
100.00%
356.0305
87.11%
(M+Na)+
246.9413
9.17%
263.9583
9.19%
711.0944
2.50%
Intensity
m/z (Da)

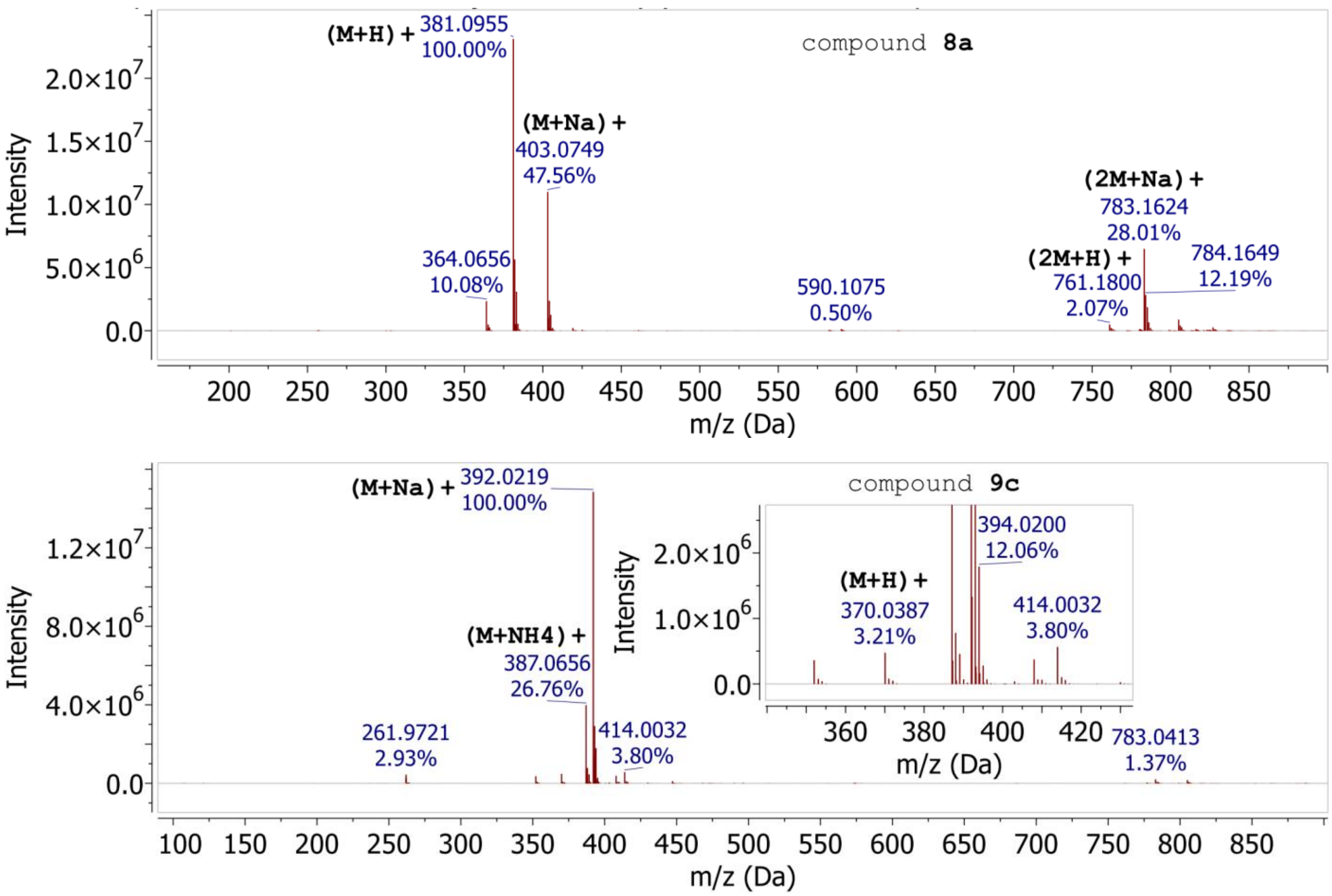

Figure S9. ESI-MS spectra of selected compounds **4a**, **4b**, **4c**, **4k**, **5b**, **7k**, **8a**, and **9c**.
